# Toward a Standardized Test of Fearful Temperament in Primates: A Sensitive Alternative to the Human Intruder Task for Laboratory-Housed Rhesus Macaques (*Macaca mulatta*)

**DOI:** 10.3389/fpsyg.2019.01051

**Published:** 2019-05-14

**Authors:** Emily J. Bethell, Lauren C. Cassidy, Ralf R. Brockhausen, Dana Pfefferle

**Affiliations:** ^1^Centre for Research in Brain and Behaviour, School of Natural Sciences and Psychology, Liverpool John Moores University, Liverpool, United Kingdom; ^2^Biological Anthropology Research Centre, School of Natural Sciences and Psychology, Liverpool John Moores University, Liverpool, United Kingdom; ^3^Welfare and Cognition Group, Cognitive Neuroscience Laboratory, German Primate Center–Leibniz Institute for Primate Research, Göttingen, Germany; ^4^Behavior and Cognition, University of Göttingen, Göttingen, Germany; ^5^Leibniz-Science Campus Primate Cognition, German Primate Center–University of Göttingen, Göttingen, Germany

**Keywords:** Human Intruder Test, behavioral inhibition, response-slowing, welfare, rhesus macaque, dysregulated fear, refinement, 3Rs

## Abstract

Standardized and sensitive tests to assess differences in temperament among primates housed in captivity are essential for monitoring welfare and improving science outcomes through reduced noise in data. Fearful temperament in primates has traditionally been assessed using the Human Intruder Test (HIT) in which duration of bodily freeze in response to approach by an unknown human is measured. The HIT is susceptible to variation between facilities in execution, interpretation of data and could be stressful for animals with more fearful temperaments. We tested the applicability of a touch-screen task with putatively negative stimuli as a more standardizable and sensitive tool for measuring fearful temperament in laboratory primates. Seventeen adult male rhesus macaques were assessed for fearfulness using the HIT. They were then tested on a touch-screen task designed to measure two behavioral indices of fearfulness: behavioral inhibition and response-slowing. We predicted monkeys assessed as having more fearful temperament in the HIT, would show the greatest degree of behavioral inhibition and response-slowing to negative pictures in the touch-screen task. In Study 1, monkeys were rewarded with juice for touching gray squares on the screen (control trials). On test trials a picture of an unknown male conspecific face with direct-gaze (signaling threat) was shown. Monkeys were less likely to touch direct-gaze faces than control trials, indicating behavioral inhibition to threat. Behavioral inhibition was greatest amongst monkeys scored with most fearful temperament in the HIT. This primary result indicates the touch-screen task may be sensitive to a more subtle form of the bodily freeze behavior measured using the HIT. In Study 2, we tested whether these findings generalized to other classes of putatively negative stimuli; monkeys were shown pictures of the human intruder and objects associated with veterinary and husbandry procedures, interspersed with control trials (gray squares). There was no evidence of behavioral inhibition in Study 2. There was some evidence for response-slowing, which was greater for pictures of objects than pictures of the human intruder, and occurred independently of fearfulness in the HIT. We propose touch-screen tasks provide a more standardized and sensitive approach for assessing fearful temperament in laboratory primates.

## Introduction

Reliable methods to assess individual differences in how animals respond to stress in the laboratory environment are essential for improved scientific outcomes, animal welfare, and worker safety and satisfaction (Buchanan-Smith, 2006; Prescott et al., 2017). Primates are widely used research models in the study of the neuroscience of fear and anxiety, with most work being conducted with *Macaca* spp. (Walsh et al., 1995; Carlsson et al., 2004; Coleman and Pierre, 2014). Macaques exhibit marked individual differences in temperament and stress responsivity (Kalin et al., 1998; Gosling and John, 1999; Gottlieb and Capitanio, 2013; Capitanio et al., 2017), which has consequences for noise in experimental data. The development and refinement of methods to measure temperamental differences in coping response to daily stressors is required for standardizing scientific methods across research groups and improving quality of science through better ability to identify and control for latent confounds that may impact scientific research outcomes (Würbel, 2002; Buchanan-Smith, 2006; Prescott et al., 2017). In particular, variation between individuals in fearful temperament in primates is important to identify as it can negatively impact both scientific outcomes and individual welfare (Jennings et al., 2009).

Fearfulness in the laboratory is assessed in terms of defensive behaviors in the presence of threatening stimuli that vary in level of threat, e.g., distance and opportunity for escape (Blanchard et al., 2011). In humans and laboratory-housed non-human primates fearfulness has primarily been studied using bodily freeze in response to threat (e.g., stranger approach in human children: Ainsworth and Bell, 1970; Buss et al., 2004; human intruder tests in non-human primates: Kalin et al., 1998) and fear-conditioning (e.g., fear-potentiated startle reflex: Lang et al., 2000). During the stranger approach test, typically used with human children, a child is left alone in an observation room until a stranger enters and approaches the child. Duration of time the child spends in a frozen posture (generally defined as maintaining a tense body posture with no movement or vocalization for at least 2 s: Buss et al., 2004) is recorded. Children who freeze for long durations in the presence of the stranger are considered to be behaviorally inhibited (Ainsworth and Bell, 1970), which is a measure of fearfulness and a known risk factor for development of affective disorders in later childhood and into adulthood (Lewis-Morrarty et al., 2015; Van Hulle et al., 2017).

An analog of the stranger approach test which has been adapted for use with non-human primates is the Human Intruder Test (HIT; Kalin and Shelton, 1989). The HIT has been most widely applied with macaques (Coleman and Pierre, 2014; Capitanio et al., 2017). As with the stranger approach test, the HIT is a formalized enactment of a regular occurrence – a person entering the room. Briefly, a monkey is isolated in a room away from its social group (or mother in the case of infants). Then, a human ‘intruder,’ often wearing a face mask to conceal their identity, enters the room and stands in profile making no eye contact with the monkey. This is considered to be an ambiguous cue since gaze aversion is a signal of subordination in macaques (Maestripieri, 1997). While the intruder stands in profile macaques tend to freeze [generally defined as maintaining a tense body posture with no movement or vocalization for at least 3 s in non-human primates (Kalin et al., 2004)]. Freezing at this point is an adaptive response allowing assessment of the situation while reducing the likelihood of detection (Blanchard et al., 2011). Enhanced freezing in the profile condition has been associated with increased right frontal lobe activity (Kalin and Shelton, 2003) which, in humans, is associated with greater negative emotion processing and reactivity to negative stimuli (Adolphs et al., 1996). After a predetermined amount of time, the intruder orients frontally to stare at the monkey [a signal of dominance and threat in *Macaca* spp. and most other non-human primates (Maestripieri, 1997)] for an equal amount of time. When stared at, monkeys typically exhibit a range of aggressive-defensive and fear behaviors, reflecting appropriate fight or flight responses (Kalin and Shelton, 2003). The intruder may then approach closer to the monkey and repeat the two orientations (Gottlieb and Capitanio, 2013). Monkeys who display high levels of freezing during the frontal (and any repeated) orientations of the HIT are considered to exhibit the most fearful temperament (Kalin, 2003; Kalin and Shelton, 2003; Buss et al., 2004).

Methods used to conduct the HIT vary between facilities, as does the focus on, and treatment of, behavioral data. For example, the human intruder may stand in each orientation for 1 min (Gottlieb and Capitanio, 2013) or 10 min (Kalin and Shelton, 1989). There may be two orientations performed at each of two distances (Capitanio, 2011) or just one distance (Corcoran et al., 2012), or additional orientations such as standing with the back to the monkey at a single distance (Coleman et al., 2017). Finally, research groups take distinct theoretical and statistical approaches to treatment of behavioral data (e.g., Gottlieb and Capitanio, 2013 did not include freeze behavior in their factor analysis while Corcoran et al., 2012 found a significant effect of intruder on freezing behavior specifically), and interpretation in terms of fearful temperament (Kalin, 2003; Kalin and Shelton, 2003; Buss et al., 2004) or anxious temperament (Rogers et al., 2008; Corcoran et al., 2012; Gottlieb and Capitanio, 2013; Coleman and Pierre, 2014). The ARRIVE guidelines (Kilkenny et al., 2010) for improving bioscience reporting advise to test the imprecision associated with results of studies, but this can be challenging when faced with variability in methods. Furthermore, some studies have demonstrated inconsistency in results obtained from the same individuals when assessed for temperament using different methods. Carter et al. (2012) for example, found that individual scores for boldness in response to predator stimuli in baboons differed from boldness measured in the same individuals as time to approach novel food.

Refined methods for assessing negative emotional states, so that methods can more easily be standardized across research facilities, are more sensitive, less intrusive and less time-consuming, would improve quality of scientific and animal welfare (Russell et al., 1959; Coleman and Novak, 2017). Standardization, reduced intrusiveness and time savings can be achieved through development of automated systems that reduce experimenter bias. This is particularly so in settings where animals are already familiar with automated systems, such as cognitive research laboratories (Calapai et al., 2017; Berger et al., 2018) and some zoos (Clark, 2017; Cronin et al., 2018). Sensitivity can be enhanced by refining methods so that testing occurs at threshold. During the HIT, it has been proposed that most animals exhibit full-body freeze behavior during the far-profile condition, an adaptive response to avoid detection (Kalin and Shelton, 1989; Coleman and Pierre, 2014). A refined method would therefore be sensitive to subtle pre-cursor indicators of fearful response that arise only in animals with high level – or dysregulated – emotion. If such indicators can be characterized these methods would provide a clear refinement to the methodology of identifying fearful individuals (Kilkenny et al., 2010; NC3Rs, 2015; Prescott et al., 2017), additionally because it reduces stress to the others.

A number of computer-based paradigms exist to measure fear-related response to affective stimuli in humans. We argue these paradigms can be adapted for use with non-human primates. Tasks typically measure reaction-time to detect targets that appear on a screen on which threat cues are also presented (Fox et al., 2001; Algom et al., 2004; Mogg et al., 2008; Frings et al., 2010; Hommer et al., 2014; Aylward et al., 2017; Mkrtchian et al., 2017). Within the literature there is a general consensus that response-slowing, or total inhibition of response, to threatening (as opposed to non-threatening) stimuli is associated with negative affect. For example, Fox et al. (2001) developed an exogenous cueing attention bias task in which participants were required to respond to a target that appeared at locations on a screen either congruent or incongruent to locations of previously displayed negative, neutral or positive words. Respondents were slower to respond to targets following negative words than to respond to targets following neutral or positive words. The authors proposed the response-slowing may have been attributable to a subtle cognitive freeze response to the negative words. Recently, reinforcement learning models (e.g., Pavlovian response), which are widely used with non-human primates in research settings, have also been applied to measure behavioral inhibition in people with mood disorders (Mkrtchian et al., 2017). The latter study revealed that individuals categorized as having a mood disorder were more likely to withhold responses on a go/no-go reinforcement learning task than people with no such diagnosis.

Based on the human cognitive literature, Bethell et al. (2016) proposed a ‘response-slowing task’ as an alternative method to the HIT, for measuring dysregulated fear in rhesus macaques (*M. mulatta)*. This touch-screen task presented pictures of conspecific faces which were considered to be potentially threatening (a neutral face with direct-gaze) or to have low threat value (a neutral face in profile). The stimuli were compiled to capture elements of both the HIT and the types of picture used in human cognitive studies. Rhesus macaques were first trained to touch a neutral gray square that appeared on a touch-screen, in order to gain a small food reward. They were then tested on their response times to touch the same gray squares when a conspecific face with direct-gaze or averted gaze appeared in the center of the square. Stimuli were presented one at a time on the touch-screen during two conditions: during a period of enrichment and in the days following a presumably stressful veterinary examination. Rhesus macaques had slower responses to direct-gaze faces (threat) relative to gray square controls following the veterinary examination, but there was no effect for the averted-gaze faces (low threat). The authors interpreted these results as possible evidence of stress-related response-slowing to threat, and proposed the task may provide a less intrusive and more sensitive alternative to the HIT in laboratory primates. More recently, researchers at Lincoln Park Zoo tested the utility of the response-slowing task for measuring the effect of a public air display (in which loud low-flying jets passed overhead on three consecutive days) on fearfulness in three species of primate: Japanese macaques (*M. fuscata*), chimpanzees *(Pan troglodytes*) and gorillas (*Gorilla gorilla gorilla*). Japanese macaques (but not the chimpanzees or gorillas) exhibited enhanced response slowing to direct-gaze faces in weeks when the air display occurred compared to weeks in which no display occurred (Cronin et al., 2018).

In order to serve as an alternative and less intrusive method to the HIT, the response-slowing task requires validation to establish the extent to which slowing and total inhibition of responses to emotional stimuli presented on a touch-screen are associated with freeze response in the HIT. Here, we present data from adult male rhesus macaques who took part in both the HIT and a touch-screen response-slowing task, adapted from Bethell et al. (2016). The rhesus macaques first took part in a HIT test during which they were assessed for fearful temperament measured as duration of freezing and fearful retreat. In our study, the human intruder was a technical staff member (with whom the monkeys had limited experience) wearing a human mask to conceal their identity. Following the HIT, we conducted two studies to assess the extent to which fearful temperament assessed in the HIT was predictive of behavioral inhibition during a touch-screen task. In Study 1, test stimuli were direct-gaze faces of male conspecifics (which we would expect monkeys to perceive as threatening). We measured the proportion of trials on which monkeys touched the faces for a reward or withheld responses (behavioral inhibition). On trials where a response was made, we measured speed to touch the face. We predicted that if the touch-screen task is sensitive to mechanisms that underlie freezing behavior in the HIT, then monkeys who show more freezing and retreat behavior during the HIT, will show greatest inhibition of response, and response-slowing, to direct-gaze faces.

In Study 2, the same cohort of monkeys took part in two response-slowing tasks during which we showed non-social putatively negative stimuli. Stimuli were images of a person wearing the same mask worn by the intruder in the HIT, and familiar objects associated with veterinarian and husbandry procedures which we presumed would have negative associations for the monkeys. We predicted that macaques who show more freezing during the HIT would be more sensitive to these non-social negative stimuli and again show greater inhibition of response, and response-slowing than non-fearful monkeys.

## Materials and Methods

### Animals and Housing

Seventeen male rhesus macaques (age range of 4–12 years: Table 1) living in isosexual groups of 2–4 at the Cognitive Neuroscience Laboratory, German Primate Centre (DPZ) took part in this research. Monkeys were housed in indoor rooms providing an enriched environment (including a multitude of toys and wooden structures, natural as well as artificial light, and space exceeding all applicable German and European regulations, Berger et al., 2018). Indoor rooms were temperature-controlled and connected via a tunnel to rooms with one side (made of wire-mesh and glass louwers) toward the outside of the building and which were at ambient outdoor temperature and lighting, but protected from precipitation. On test days the monkeys had free access to water for at least 4 h (typically much more: for definitions of access to water see Pfefferle et al., 2018) and received monkey chow *ad libitum*. On non-test days the monkeys had free access to water and received monkey chow *ad libitum*, supplemented with dried fruits, fresh fruits and vegetables. The health of the monkeys was monitored daily by the animal care staff, DPZ veterinarians, and the laboratory researchers who are all highly experienced with these animals.

**Table 1:** Animal identity and age at taking part in the HIT, arranged by descending HIT freeze-fear score, with performance data for Study 1 (faces) and Study 2 (mask and object).

Animal info.		HIT	Study 1: face			Study 2: mask			Study 2: object		
ID	Age tested on HIT (years)	Freeze-fear score	Control (mean prob. to touch)	Test (mean prob. to touch)	RT diff. score (mean, ms)	Control (mean prob. to touch)	Test (mean prob. to touch)	RT diff. score (mean, ms)	Control (mean prob. to touch)	Test (mean prob. to touch)	RT diff. score (mean, ms)
Der	6.12	479.86	1.00	0.56	89.99	0.90	0.89	-48.52	0.86	0.78	77.86
Kas	11.02	479.60	0.96	0.94	-6.41	1.00	0.96	-0.84	0.95	0.89	-8.50
Cas	7.26	430.33	0.94	0.67	19.69	1.00	1.00	13.32	1.00	1.00	52.46
Nor	6.20	366.55	0.97	0.94	23.56	1.00	1.00	-8.30	1.00	1.00	163.38
Lou	6.29	357.09	0.90	0.67	126.03	0.85	0.62	173.30	0.81	0.59	562.84
Pac	6.02	309.77	0.99	1.00	15.17	0.98	0.97	13.99	0.96	1.00	80.48
Osk^1^	11.63	291.94	–	–	2.54	–	–	-11.86	–	–	17.91
Elm	6.04	290.10	0.96	1.00	22.22	0.65	1.00	-27.04	0.62	0.94	87.73
Rio	5.69	275.34	0.95	0.94	110.81	0.93	1.00	44.49	0.93	0.83	103.12
Hum^1^	11.29	257.60	–	–	14.94	–	–	16.71	–	–	23.79
Han	6.36	256.87	1.00	1.00	28.37	0.97	1.00	20.91	0.89	0.82	29.52
Zaz^2^	6.13	242.81	–	–	–	0.79	0.74	5.89	0.74	0.40	327.85
Bex^2^	5.74	240.04	–	–	–	0.89	0.89	400.13	0.74	0.61	701.77
Rod	4.22	238.94	0.99	0.89	55.20	1.00	0.88	87.83	1.00	0.89	106.21
Cor^3^	10.80	230.84	–	–	–	1.00	0.94	78.45	1.00	1.00	36.72
Gro	6.95	169.93	0.97	0.94	138.77	0.62	0.94	-105.96	0.93	1.00	352.26
Fla^4^	8.19	138.60	1.00	1.00	33.80	–	–	–	–	-	-

*Summary data for ages at testing in Study 1 and Study 2 are reported in the text, both studies occurring within a 21-month window after the HIT. Mean reaction time (RT) difference scores were calculated as (RT test – RT control). ^*1*^Hum and Osk were tested using 1,000 ms target duration for Study 1 and Study 2 hence their ‘go/no-go’ data were not comparable with that from the 3,000 ms duration. They were retained in the analysis of RT difference scores. ^*2*^Bex and Zaz were excluded from analyses for Study 1 as they were temporarily housed separately for veterinary purposes. Emotional state has previously been shown to influence responses during this task (Bethell et al., 2016). ^*3*^Cor was not available for testing within the timeframe of Study 1. ^*4*^Fla did not reach the performance criterion for Study 2 (mean probability to touch on control trials: 0.45 and 0.47 for mask and object test sessions, respectively).*

### Testing Compartment

Each indoor room was connected to a testing compartment (ca. 80^∗^75^∗^90 cm), with wire mesh walls in an adjacent room, accessible through a sliding door where monkeys regularly took part in touch-screen tasks, were fed treats by care staff, and where they could be separated from the group for veterinary inspection. All animals were used to entering this compartment on a daily basis and many worked daily on touch-screen tasks there. The HIT, and subsequently the response-slowing task (Study 1 and Study 2), were conducted in this area. For all individuals the HIT preceded the response-slowing task (mean lag = 22 weeks; range = 1–89 weeks). Monkeys were separated from social group members and encouraged to enter their respective testing compartment with small fruit rewards (grape, raisin or banana) prior to each testing session. For the HIT, social group members and those animals in adjacent rooms were moved out of sight (to their outdoor enclosure) to prevent exposure to the experiment.

### Human Intruder Test (HIT)

All monkeys completed the HIT prior to cognitive testing, as part of a pre-existing welfare protocol to assess temperament in animals at the facility. A monkey was first separated in its testing compartment and allowed to settle for 5 min. The HIT progressed in four stages: far-profile, far-frontal, near-profile, and near-frontal (after: Gottlieb and Capitanio, 2013; Capitanio et al., 2017). Initially a male adult human (wearing a human mask to conceal his identity), entered the room, approached a point marked 1 m of the front of the compartment and stood in profile to the compartment for 1 min (far-profile condition). After 1 min the experimenter turned to face and look at the monkey for 1 min (far-frontal). Both orientations (profile and frontal) were then repeated at another point 0.3 m from the front of the compartment (near-profile and near-frontal). All stages of the HIT were recorded on a Panasonic (HC-W580) video camera and video was later coded for analysis of behavioral response using the program BORIS v.6.0.2 (Friard and Gamba, 2016). Only one monkey from a pair was tested on any 1 day to minimize disruption for each pair. We identified two non-mutually exclusive behavioral categories of interest from previous studies that used the HIT (Kalin et al., 1998; Gottlieb and Capitanio, 2013) and literature on defensive distance to threat (Blanchard et al., 2011): freeze response (>2 s in a frozen posture) and fearful retreat (defined here as time spent at the back of the compartment). All monkeys were naïve to the HIT at the start of this study.

### Cognitive Task

#### Stimuli and Apparatus

**FIGURE 1:**
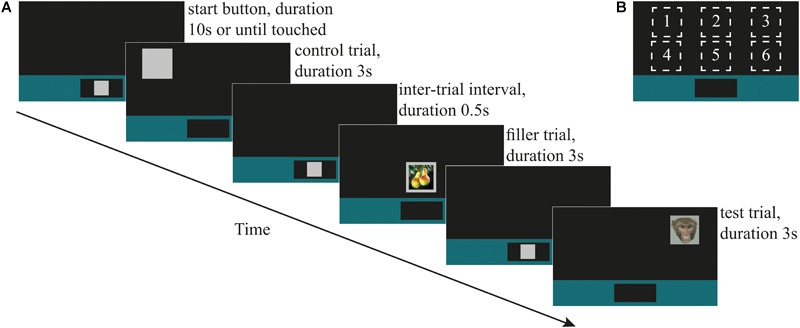
**(A)** An illustrative timeline showing (in chronological order) the ‘start button,’ always displayed at lower center of the screen; control stimulus (during testing; also used as the training stimulus during training), shown at screen location 1; the 0.5 s inter-trial interval (ITI) until the onset of the next start button; filler stimulus ‘fruit,’ shown at screen location 5; ITI and start button; direct-gaze face (test stimulus in Study 1), shown at screen location 3. **(B)** The six locations on the screen at which stimuli were shown.

The response-slowing task was adapted from Bethell et al. (2016). There were four types of stimuli: ‘training,’ ‘filler,’ ‘control,’ and ‘test’ (Figure 1A). Training stimuli consisted of a 70% luminance gray square with a side length of 78 mm on the screen. We created two additional categories of training stimuli by superimposing pictures that we presumed to have positive or neutral emotional valence for the monkeys onto the training stimulus. Images were of fruits (*n* = 18; ‘fruit’) and unknown conspecific infants (*n* = 18 ‘infant’; collected by DP at the Cayo Santiago Field Station of the Caribbean Primate Research Centre, Puerto Rico). Nine ‘fruit’ and 11 ‘infant’ stimuli were also presented during testing as filler stimuli to maintain monkeys’ interest in the task. The control stimulus during testing was the gray square that had been used during training.

Test stimuli were pictures that we presumed to have negative emotional valence for the monkeys. In Study 1 (faces), there was one category of test stimulus: ‘direct-gaze face’ for which pictures of unknown conspecific adult male faces with direct gaze were used (18 identities, photographs taken by DP at the Cayo Santiago Field Station). Pictures were trimmed so that only the monkey’s head was visible. These trimmed photos were superimposed onto the center of the control stimulus (leaving a 6 mm gray border).

In Study 2 (mask and objects), two categories of test stimulus were created. The first category ‘mask’ consisted of a picture of the mask previously seen during the HIT. The second category ‘objects’ comprised color photographs of veterinary and husbandry objects (one picture each of a glove, a broom and a net).

Stimuli were presented on an e**X**perimental **B**ehavioral **I**nstrument (XBI: Calapai et al., 2017; Berger et al., 2018) using MWorks^1^ software. The built-in drinker spout of the XBI was placed 250 mm in front of the center of the integrated 15″ (300 mm × 225 mm) touch-screen monitor. All monkeys had previously worked with the XBI in previous studies.

#### Procedure

All training and test sessions followed a block design and consisted of a maximum of 120 trials. Prior to testing, monkeys were trained to respond to the control and filler stimuli presented at six locations on the touch-screen (Figure 1B). Monkeys began a trial by touching a 70% luminescence gray square (‘start button’) that appeared within a colored bar at the bottom of the screen for up to 10 s. Once touched, the start button disappeared and one of the three categories of training stimulus (control, fruit, or infant) appeared in one of the six screen locations. The stimulus remained on the screen for 3 s^∗^ regardless of whether the monkey touched it. (^∗^During the training phase, 15 monkeys worked consistently with a stimulus duration of 3 s and generally performed up to 120 trials within approximately 120 min. We used a 1 s stimulus duration for two monkeys who indicated poor motivation to work consistently during this training phase.) If the monkey touched the stimulus, as visual feedback, the gray part of it decreased to 35% luminescence (i.e., darkening), a secondary reinforcing tone was played via a speaker behind the apparatus and the monkey was rewarded with automatic delivery of a 0.25 ml fluid reward via the drinker spout. Fluid reward was a mix of 70% plain water and 30% flavored water (active O_2_, Adelholzener) or grape juice (kept consistent for each monkey). Primary and secondary reinforcers were delivered on a 100% fixed reinforcement ratio during training and testing phases. All responses were recorded automatically by the MWorks program. There was a fixed 500 ms inter-trial interval between the stimulus offset and the onset of the start button for the next trial. If the monkey touched the screen during the interval the timer reset so that the onset of the next trial occurred 500 ms after the monkey stopped touching the screen. Each category of stimulus was presented an equal number of times at each of the six screen locations. Criterion for learning the task was 80% touches on control trials.

Once a monkey had reached criterion for training, he began testing on the next available day. A test session always began with a warm-up block (*n* = 6 trials) in which the monkey had to first respond by correctly touching on three control (C) trials, followed by a control and a filler (F; i.e., pictures of infants) trial (which could occur in either order), and followed by a final control trial (i.e., C, C, C, F + C, C). A trial occurred once at each of the six screen locations, with order of location randomized for each testing session. The test block comprised 108 trials (control *n* = 72, test *n* = 18, filler *n* = 18) in which trials occurred in a pseudo-randomized order with the instruction that the 18 test (T) trials were always preceded by at least one, and no more than three, consecutive control trials (i.e., ‘F, C, T…’ and ‘F, C, C, C, T…’). On completing the test block, the test session ended with a cool-down block (*n* = 6 trials) containing one control trial, followed by one control and one filler (infant) trial in randomized order, and ending with three control trials (C, F + C, C, C, C). Again, trials occurred at random, once at each of the six screen locations. In Study 1, (faces) each monkey took part in one daily session. In Study 2 (mask and objects), each monkey took part in one daily test session with mask stimuli, and one daily test session with object stimuli, counterbalanced between monkeys for order in which they did these. Criterion for including data from a given monkey in the analysis was at least 65% touches on control trials in a daily test session.

### Data Preparation

Behavioral data for duration of freeze and time spent at the back of the testing compartment were recorded as total duration for each behavioral category summed across the four 1-min stages of the HIT: far/near × profile/frontal, (after Capitanio et al., 2017). Correlational analysis revealed a positive correlation between freeze and fearful retreat (*r*_1,14_ = 0.54, *P* = 0.05) and so we combined scores to create a single measure we labeled ‘freeze-fear score,’ and which could have a maximum score of 480 s summed across the two non-mutually exclusive categories.

Data from the touch-screen task were treated following common protocol in the human literature (e.g., Mogg et al., 2008; Holmes et al., 2009). Only control and test trial data from the test block were used in the analysis. Data were in two forms: binomial go/no-go response and latency to respond on ‘go’ trials. We removed all responses faster than 300 ms (deemed to have occurred too quickly to reflect meaningful response to the stimuli: see also Bethell et al., 2016). To reduce the influence of outliers, for ‘go’ trials we trimmed the data at 2 SD above the mean per individual. To estimate response-slowing effects on test trials, we calculated a reaction time (RT) difference score for each monkey by subtracting the mean RT on control trials from the mean RT on test trials, controlling for location on the screen. Positive values therefore indicate slower responses on test trials compared with controls and negative values indicate faster responses on test trials compared with controls. The full data set can be accessed at Supplementary Data Sheet S1.

### Statistical Analysis

Statistical analyses were conducted in R v. 3.4.3 (R Core Team, 2017). In both Study 1 and Study 2, we fitted linear mixed models including all possible random slopes using the package ‘lme4’ version 1.1-15 (Bates et al., 2015). Subject identity was entered as a random factor in all models. To keep type I error rates at the nominal level of 0.05, we added all possible random slopes estimates [i.e., for all factors with cases representing all individuals at most levels (Schielzeth and Forstmeier, 2009; Barr et al., 2013)]. We did not include the correlations between random slopes and intercepts into the models, because of increased computation time and since it is known that neglecting them does not compromise type I error rates (Barr et al., 2013). Prior to fitting the models, we checked all predictor variables for correlations above 0.4 (which could result in collinearity), checked all response and predictor variables for their distribution and transformed data to obtain more normal distributions when necessary. We scaled all covariates using a z-transformation to a mean of zero and a standard deviation of one. Scaled covariates provide more comparable estimates and are easier to interpret with regards to interactions (Aiken and West, 1991; Schielzeth, 2010). For plotting purposes we present untransformed values, and model lines obtained from these, to aid interpretation.

#### Study 1 (Direct-Gaze Faces)

In Study 1, we fitted two linear mixed models to test the hypothesis that more fearful monkeys (as assessed using the HIT) would show (a) enhanced behavioral inhibition of response and (b) response-slowing to negative stimuli (conspecific face with direct-gaze) during the touch-screen task. We therefore entered our key predictor variables stimulus type (test, control) and freeze-fear score as an interaction term. We controlled for the potential effects of two factors: stimulus location on the screen (six locations) and XBI apparatus number (five machines); and two covariates: age and trial number. Trial number was square root transformed following distribution checks. We fitted this generalized linear mixed model (including all possible random slopes and subject as random factor) using the ‘glmer’ function with binomial error structure and logit link function (McCullagh and Nelder, 1996). Since the model did not converge when using the default optimizer ‘Nelder-Mead’ and the default number of iterations, we used the argument ‘control’ to specify the optimizer to ‘bobyqa’ and increased the number of iterations to 100,000. We visually inspected the distribution of the random effects for normality, finding no indication of influential cases. Additionally, we assessed model stability using a loop that excludes data points one by one from the data set, and comparing the model estimates derived with those obtained from the full model, again there was no indication of influential cases. To rule out collinearity, we determined Variance Inflation Factors (VIF: Tabachnick and Fidell, 2001; Quinn and Keough, 2002; Field, 2005; Zuur et al., 2010) using the function ‘vif’ of the R-package car (Fox and Weisberg, 2011) applied to a standard linear model with all response and predictor variables, but excluding the interactions and random effects (maximum VIF = 1.40).

For those trials in which the monkeys did make a response, we ran a second generalized linear mixed model testing for a relationship between freeze-fear score from the HIT and the reaction time (RT) difference score. In this model, we controlled for the potential effects of the factors stimulus location on the screen (six locations) and XBI apparatus (five machines), and the covariates age and temporal sequence of stimulus presentations (an adjusted form of trial number since information about unique trial number was lost after removing ‘no-go’ trials and calculating RT difference scores). Following distribution checks, we square root transformed the variable temporal sequence of stimulus presentations. Data were analyzed fitting a general linear mixed model (including all possible random slopes and subject as random factor) using the ‘lmer’ function with Gaussian error structure and identity link function (Baayen, 2008). We checked whether the assumptions of normally distributed and homogeneous residuals were fulfilled by visually inspecting qq-plots and plots of residuals against fitted values. We visually inspected the distribution of random effects for normality and checked for model stability by excluding subjects one at a time and comparing the model estimates derived for these subsets of the data with those derived for the full data set. We found no evidence for influential cases. Variance Inflation Factors (VIF: Tabachnick and Fidell, 2001; Quinn and Keough, 2002; Field, 2005; Zuur et al., 2010) were derived using the function ‘vif’ of the R-package car (Fox and Weisberg, 2011) applied to a standard linear model excluding the interactions and random effects (maximum VIF = 1.46).

#### Study 2 (Mask and Object)

As in Study 1, we ran two models to test the sensitivity of the touch-screen task to fearfulness, this time to a human mask and objects associated with veterinary and husbandry procedures. In the first model, we tested whether the probability to touch a stimulus on the touch-screen (‘go/no-go’) is explained by freeze-fear score and stimulus type (test, control) entered as an interaction term, and freeze-fear score and stimulus category (mask, object) entered as a second interaction term. Control variables were the factors order of test session (mask test session first or object test session first), previous exposure to the test stimuli (in cases where sessions were terminated early for extraneous reasons and rerun later: yes/no), location on the screen (six locations) and XBI apparatus (five machines), and the covariates age and trial number. Following distribution checks we square root transformed the response variable and trial number. We fitted this generalized linear mixed model (including all possible random slopes and subject as random factor) using the ‘glmer’ function with binomial error structure and logit link function (McCullagh and Nelder, 1996). Since the model did not converge when using the default optimizer ‘Nelder-Mead’ and the default number of iterations, we used the argument ‘control’ to specify the optimizer to ‘bobyqa’ and increasing the number of iterations to 1,000,000. We checked the assumptions of the model by visually inspecting the distribution of the random effects for normality, assessed model stability (excluding data points at a time and comparing the model estimates of that reduced model with those of the full model), and tested for issues of collinearity (maximum VIF = 1.78). None of these tests indicated an issue.

In the second model, we tested for a possible effect of freeze-fear score and stimulus category (mask, object) as an interaction term on RT difference score. We fitted a general linear mixed model (including all possible random slopes and subject as random factor) using the ‘lmer’ function with Gaussian error structure and identity link function (Baayen, 2008). Control variables were the factors order of test session (mask session first or object session first), previous exposure to the test stimuli (in cases where sessions were terminated early for extraneous reasons and rerun later: yes/no), location on the screen (six locations) and XBI apparatus number (five machines), and the covariates age and temporal sequence of stimulus presentations (an adjusted form of trial number since information about unique trial number was lost after removing ‘no-go’ trials and calculating RT difference scores). Following distribution checks we square root transformed the response variable as well as the control variable temporal sequence of stimulus presentations. Visual inspection of qq-plots and plots of residuals against fitted values indicated four outliers. We checked the outliers but since no reason could be identified for why those four cases appeared different, and model stability checks indicated no influential cases, we retained the outliers in the model (model results with these four cases removed revealed no meaningful change in the analysis output or interpretation). Variance Inflation Factors gave no indication of collinearity (largest VIF = 1.78).

In Studies 1 and 2, following model checks, we tested the significance of the full model (comprising all predictor variables, random slopes and random effects) against its null model (comprising the intercept, control variables, random slopes and random effects) using a likelihood ratio test (function ‘anova’ with the argument test set to ‘Chisq’; Dobson, 2002; Forstmeier and Schielzeth, 2011). To allow for a likelihood ratio test we fitted the models using Maximum Likelihood (rather than Restricted Maximum Likelihood: Bolker et al., 2009). Where a model comparison result revealed a significant or marginally significantly better fit of the full model, we used the ‘drop’ function to assess whether any interactions were significant and therefore to be retained or removed in the final model (Barr et al., 2013).

### Ethics Statement

This study complied with institutional guidelines on Animal Care and Use of the German Primate Center and was conducted in accordance with national and international guidelines on the use of primates in research including German Animal Protection Law, the European Union Directive 2010/63/EU on the Protection of Animals used for Scientific Purposes and the Society for Neuroscience Policies on the Use of Animals and Humans in Neuroscience Research. The animals used in this study were all highly accustomed to settings and scenarios experienced in the current study. All animals in this study were also participating in studies that require a governmental permit due to being animal experiments (the permit was issued by the responsible regional government office, Niedersaechsisches Landesamt fuer Verbraucherschutz und Lebensmittelsicherheit, LAVES, under the permit number 3392 42502-04-13/1100). Additionally, the study was approved by Liverpool John Moores University Ethical Review Panel under permit number EB/20-184.

## Results

### HIT

Seventeen monkeys completed the HIT (mean age at time of HIT: 7.51 years, range 4.22–11.63 years; Table 1), producing a total of 68 min of video for coding. All monkeys exhibited freeze response and fearful retreat during the first stage of the HIT (far-profile), and all monkeys also exhibited fearful retreat in the near stage (both near-profile and near-frontal). Four monkeys exhibited freeze response in the near stage (both near-profile and near-frontal). Mean freeze-fear score across all four stages of the HIT was 297 s (range 139–480).

### Study 1: Fearful Temperament Predicts Behavioral Inhibition (but Not Response Slowing) to Direct-Gaze Faces

For touch-screen Study 1 (direct-gaze faces), 16 monkeys initially took part, completing 1598 test and control trials (test *n* = 285). All monkeys reached the 65% performance criterion for consideration for inclusion in the analysis. We discarded warm-up and warm-down trials (9.95% of the data, *n* = 159 trials) and responses faster than 300 ms (0.38% of the data, *n* = 6 control trials) resulting in 1,433 trials. For go/no-go responses we then removed data from the two monkeys who had worked on the 1 second trial duration (Hum and Osk: 11.26% of the data, *n* = 180 trials), and two monkeys who had been temporarily singly housed for veterinary purposes during the study period (Bex and Zaz: 11.14% of the data, *n* = 178 trials; Table 1). This resulted in 1075 ‘go/no-go’ trials (test *n* = 216) from 12 monkeys (mean age on first day of Study 1: 7.81 years, range 5.96–12.18) for inclusion in the analysis. Mean probabilities to respond on control and test trials were 0.97 ± 0.01 and 0.88 ± 0.04, respectively. For the analysis of latency to respond on ‘go’ trials, we included data from the two monkeys who worked on the 1 s schedule, and discarded all non-responses (6.51% of the data, *n* = 103 trials), and all trials with RTs above 2 SD of individual means (4.57% of the data, *n* = 73 trials). We calculated the RT difference scores from the remaining 1,257 ‘go’ responses (matched for stimulus identity and location on the screen), resulting in 204 RT difference scores from 14 monkeys (mean age on first day of testing in Study 1: 8.53, range 5.96–12.18) for analysis. Mean RT difference score was 48.19 ± 12.83 ms.

**FIGURE 2:**
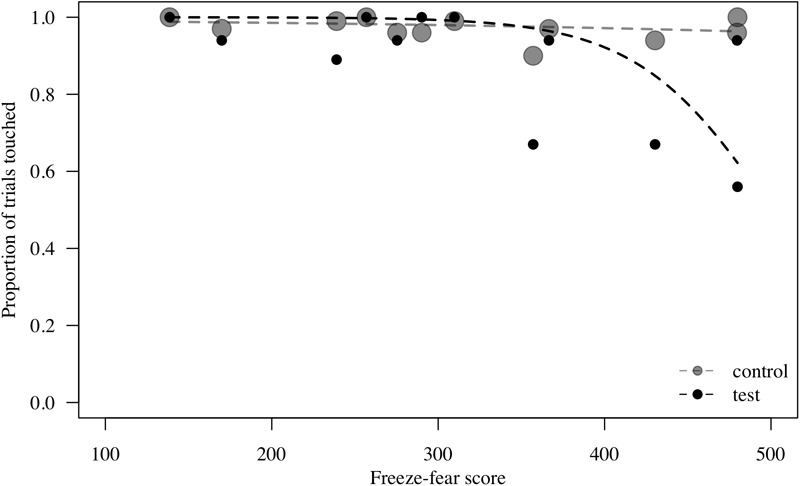
Probability to touch test (direct-gaze face) and control trials combined, plotted against freeze-fear score for *n* = 12 monkeys in Study 1. Individual means are shown. Circle size indicates number of trials. Lines represent model estimates.

**FIGURE 3:**
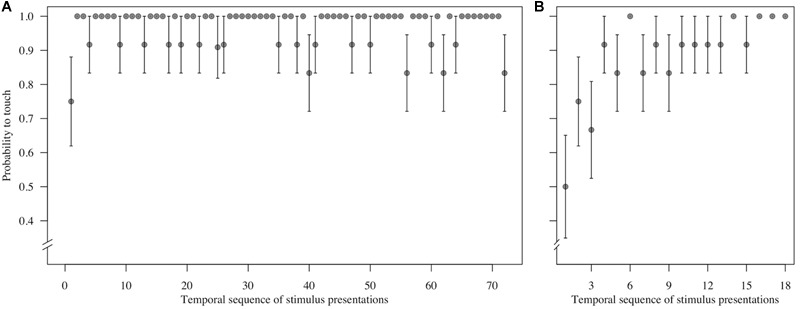
Mean ± SE probability to touch **(A)** control trials (*n* = 72 trials per monkey) and **(B)** test (direct-gaze face) trials (*n* = 18 trials per monkey) in the temporal sequence in which they were shown during the test block for *n* = 12 monkeys in Study 1. Group means are shown.

To assess behavioral inhibition of response, go/no-go data were entered in the model as the binary response variable. The full model (containing the interaction term between stimulus type and freeze-fear score and the four control variables) was significantly different compared to the null model (excluding the interaction term between stimulus type and freeze-fear score, but retaining the intercept and the four control variables: likelihood ratio test, LRT: χ^2^= 14.68, df = 3, *P* = 0.002). The model revealed a significant interaction between stimulus type and freeze-fear score (LRT: χ^2^= 4.32, df = 1, *P* = 0.038: Table 2). Monkeys were significantly less likely to touch direct-gaze faces than controls overall (*z* = -3.10, *P* = 0.002) and this effect was greatest for monkeys with higher freeze-fear scores (z = -2.22, *P* = 0.078; Figure 2). For the four control variables (stimulus location on the screen, XBI apparatus number, age and trial number) we found a significant and positive relationship for trial number (LRT: χ^2^= 4.02, df = 1, *P* = 0.045) whereby monkeys responded on proportionally more trials as the session progressed, an effect that visual inspection of the data revealed was driven by the change in probability of responding during the early presentations of the test trials (Figure 3). There were also significant effects of XBI number (LRT: χ^2^= 10.12, df = 3, *P* = 0.018) and stimulus location (LRT: χ^2^= 17.83, df = 5, *P* = 0.003) on probability to touch stimuli. The latter result appeared to be driven by a tendency to make more responses to stimuli presented in the lower half of the screen (nearer the start button) than the upper part of the screen, with no indication of visual field effects (Table 3).

**Table 2:** Results of the generalized linear mixed model for Study 1 (direct-gaze faces) examining the interaction between test predictors stimulus type and freeze-fear score on the probability of monkeys to touch stimuli presented on the touch-screen.

Predictor variable	Estimate	*SE*	*Z*	*p*	95% CI_lower_	95% CI_upper_	χ^2^	df	*P*
Intercept	4.56	0.83	5.44	<0.001	3.14	6.49			
**Test predictor**									
Stimulus type × freeze-fear score	-1.07	0.48	-2.22	0.026	-1.94	-0.07	4.32	1	0.038
Stimulus type									
Stimulus type (test)^1^	-1.28	0.41	-3.10	0.001	-2.07	-0.19			
Freeze-fear score^a^	-0.65	0.37	-1.76	0.078	-1.44	0.06			
**Control predictors**									
Age^b^	-0.45	0.37	-1.23	0.22	-1.20	0.18	1.97	1	0.161
Target location^2^							17.83	5	0.003
Target location(2)^3^	-0.68	0.49	-1.40	0.162	-1.68	0.39			
Target location(3)^3^	-0.49	0.50	-0.99	0.321	-1.50	-0.47			
Target location(4)^3^	1.60	0.82	1.95	0.051	0.15	3.59			
Target location(5)^3^	-0.36	0.54	-0.68	0.497	-1.47	0.83			
Target location(6)^3^	1.60	1.13	1.41	0.157	-0.15	5.08			
Trial number^c^	0.82	0.44	1.87	0.061	0.02	1.82	4.02	1	0.045
XBI apparatus^4^							10.12	4	0.018
XBI apparatus(2)^5^	-2.34	0.88	-2.65	0.008	-4.40	-0.78			
XBI apparatus(3)^5^	-0.71	0.87	-0.82	0.413	-2.65	0.92			
XBI apparatus(4)^5^	0.57	0.87	0.66	0.511	-1.22	2.40			

*Results for the control predictor variables are also shown. ^*1*^Stimulus type was dummy coded with stimulus type gray (control) being the reference category. ^*2*^Likelihood ratio test comparing the full with the reduced model lacking target location. ^*3*^Target location was dummy coded with location 1 being the reference category. ^*4*^Likelihood ratio test comparing the full with the reduced model lacking XBI apparatus. ^*5*^XBI apparatus was dummy coded with apparatus 1 being the reference category. ^*a*^z-transformed, mean and range of the original values were 316.09 and 138.60–479.86, respectively. ^*b*^z-transformed, mean and range of the original values were 7.81 and 5.96–12.18 years, respectively. ^*c*^Square-root and z-transformed, mean and range of the original values were 263.38 and 1–990, respectively.*

**Table 3:** Probability to touch test (direct-gaze face) and control (gray square) trials at each of the six screen locations in Study 1.

Stimulus	Target position on screen (probability to touch)		
	**1**	**2**	**3**
Face	0.89	0.78	0.81
Gray	0.97	0.96	0.96
	**4**	**5**	**6**
Face	0.97	0.83	1.0
Gray	0.99	0.96	0.98

For RT difference scores in Study 1 the full model did not explain the data better than the null model (LRT: χ^2^= 2.42, df = 1, *P* = 0.120). We ran no further analyses.

### Study 2: Fearful Temperament Does Not Predict Behavioral Inhibition or Response Slowing to a Mask or Objects

For touch-screen Study 2 (mask and objects) 17 monkeys initially took part, of which 16 monkeys responded on control trials above the 65% performance criterion (Table 1). The 16 monkeys completed a total of 3,536 test and control trials (test *n* = 631). We discarded warm-up and warm-down trials (10.01% of the data, *n* = 354 trials) and responses faster than 300 ms (0.48% of data, *n* = 4 test and *n* = 13 control trials) resulting in 3,165 trials. For go/no-go responses we then removed data from the two monkeys who had worked with the 1 s trial duration (Hum and Osk: 13.09% of the data, *n* = 463 trials) resulting in 2,702 trials (test *n* = 537 trials) from 14 monkeys (mean age on first day of Study 2: 7.17 years, range 5.05–11.21 years) for analysis. Mean proportion of responses on control and test trials during the mask study were 0.90 ± 0.03 and 0.92 ± 0.03, respectively, and in the object study were 0.89 ± 0.03 and 0.84 ± 0.05. For the analysis of latency to respond on ‘go’ trials, we retained the two monkeys working on the 1 s schedule and discarded all non-responses (8.99% of data, *n* = 318 trials), and RTs above 2 SD of individual means (3.91% of the data, *n* = 138 trials). We calculated the RT difference scores from the remaining 2767 ‘go’ responses, resulting in 521 difference scores (mask: *n* = 283, object: *n* = 238) from 16 monkeys (mean age on first day of Study 2: 8.02 years, range 5.05–11.95 years) for analysis. Mean RT difference scores for the mask and object studies were 40.78 ± 28.51 and 169.71 ± 52.23, respectively.

For the ‘go/no-go’ data the full vs. null model comparison was non-significant (LRT: χ^2^ = 7.46, df = 5, *P* = 0.188) and we ran no further analyses.

**FIGURE 4:**
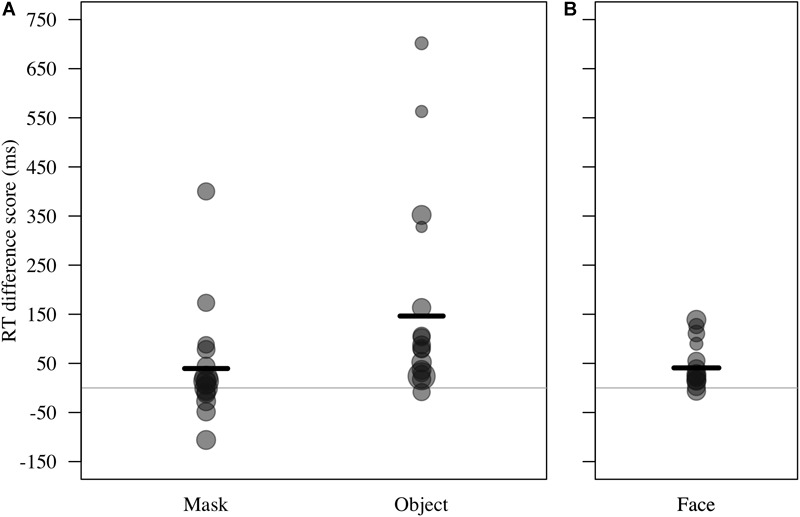
**(A)** Reaction time difference score (RT test trial – RT control trial, in ms) for responses to masks and objects by *n* = 16 monkeys in Study 2. Individual means and model estimate shown. Circle size indicates number of trials. **(B)** RT difference scores for responses to faces (*n* = 12 monkeys in Study 1) are shown with model estimate for comparison purposes.

**FIGURE 5:**
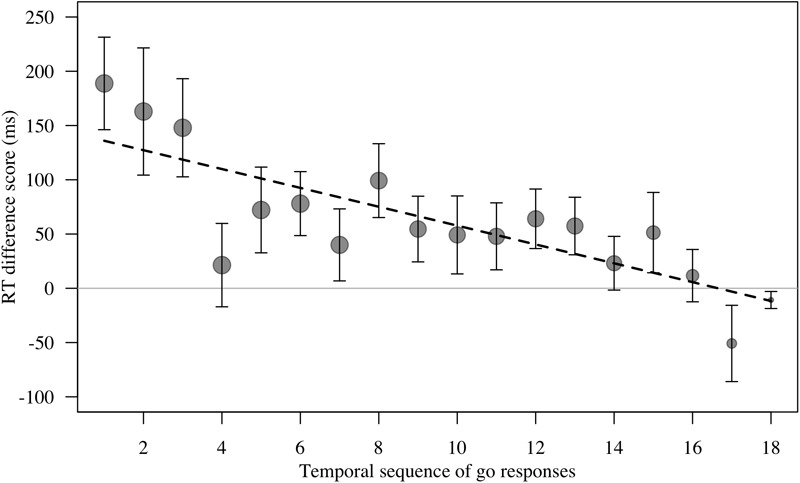
Mean ± SE reaction time difference score (RT test trial – RT control trial, in ms) for responses to masks and objects (combined) by *n* = 16 monkeys in Study 2. There were *n* = 18 test trials per stimulus category per monkey. Data are plotted as temporal sequence of ‘go’ responses made. Group means are shown. Circle size indicates number of trials. Smaller circles for trial to the right reflect the fact that many monkeys did not touch every test trial. Line represents model estimate.

For RT difference score, the comparison between the full and the null model revealed a significant deviation (LRT: χ^2^= 8.17, df = 3, *P* = 0.043; Table 4). The interaction term was not significant (χ^2^= 1.88, df = 1, *P* = 0.170), so we removed it from the model. The final model indicated a significant effect of test type on RT difference score (LRT: χ^2^= 6.10, df = 1, *P* = 0.014), with monkeys showing greater response-slowing to objects than to mask stimuli (estimate = -1.72, *t* = -2.73; Figure 4). We also found a significant change in RT difference scores as the sessions progressed (LRT: χ^2^= 6.49, df = 1, *P* = 0.011; Table 4), with monkeys showing greatest response-slowing during the first three presentations of the test stimuli, which decreased over time (estimate = -0.48, *t* = -2.77; Figure 5). There was a marginally significant effect of age on RT difference scores (LRT: χ^2^= 3.05, df = 1, *P* = 0.081; Table 4), revealing a trend for response-slowing being greater amongst younger animals. Results of the same analysis excluding the four outliers (reported in the “Materials and Methods” section) pointed in a similar direction.

**Table 4:** Results of the general linear mixed model for the Study 2 (mask and object) examining the effect of the test predictors test type and freeze-fear score on the reaction time difference score.

Predictor variable	Estimate	*SE*	*T*	95% CI_lower_	95% CI_upper_	χ^2^	df	*P*
Intercept	26.93	1.62	16.62	23.64	30.35			
**Test predictors**								
Test type^1^						6.10	1	0.014
Test type (Mask)^2^	-1.72	0.63	-2.73	-3.04	-0.40			
Freeze-fear score^a^	-0.25	0.46	-0.54	-1.22	0.71	0.28	1	0.594
**Control predictors**								
Previous exposure^3^						0.08	1	0.781
Previous exposure (yes)^4^	0.14	0.51	0.28	-0.87	1.17			
Test order^5^						0.18	1	0.671
Test order(2nd)^6^	0.57	1.33	0.43	-2.25	3.30			
Age^b^	-1.20	0.65	-1.86	-2.52	0.16	3.05	1	0.081
Target location^7^						6.96	5	0.224
Target_location(2)^8^	0.72	0.47	1.55	-0.21	1.65			
Target_location(3)^8^	1.17	0.49	2.40	0.16	2.15			
Target_location(4)^8^	0.47	0.49	0.97	-0.51	1.47			
Target_location(5)^8^	0.24	0.50	0.48	-0.80	1.26			
Target_location(6)^8^	0.84	0.46	1.80	-0.08	1.79			
Temporal sequence of stimulus presentation^c^	-0.48	0.17	-2.77	-0.87	-0.13	6.49	1	0.011
XBI apparatus^9^						2.53	4	0.640
XBI apparatus(2)^10^	1.86	1.49	1.25	-1.25	4.97			
XBI apparatus(3)^10^	0.37	1.44	0.25	-2.59	3.42			
XBI apparatus(4)^10^	1.87	2.45	0.76	-3.31	6.94			
XBI apparatus(5)^10^	1.81	1.57	1.15	-1.51	4.99			

*^*1*^Likelihood ratio test comparing the full with the null model lacking test type. ^*2*^Test type was dummy coded with test type object being the reference category. ^*3*^Likelihood ratio test comparing the full with the reduced model lacking the predictor exposure. ^*4*^Exposed was dummy coded with the level not being exposed before being the reference category. ^*5*^Likelihood ratio test comparing the full with the reduced model lacking the predictor experiment order. ^*6*^Experiment order was dummy coded with the level being shown first being the reference category. ^*7*^Likelihood ratio test comparing the full with the reduced model lacking the predictor target location. ^*8*^Target location was dummy coded with location 1 being the reference category. ^*9*^Likelihood ratio test comparing the full with the reduced model lacking the predictor XBI apparatus. ^*10*^XBI apparatus was dummy coded with apparatus 1 being the reference category. ^*a*^z-transformed, mean and range of the original values were 308.52 and 169.93–479.86, respectively. ^*b*^z-transformed, mean and range of the original values were 8.02 and 5.05–11.95 years, respectively. ^*c*^Square-root and z-transformed, mean and range of the original values were 8.09 and 1–18, respectively.*

## Discussion

Developing standardized and sensitive tools that improve scientific outcomes while positively impacting animal welfare is a key 3Rs outcome. We proposed that touch-screen tasks sensitive to fearful temperament in humans, and presumably ethically non-problematic given their widespread use in human cognitive psychological research, could be adapted to assess fearful temperament in another primate species. We tested this hypothesis in male rhesus macaques, by comparing behavioral inhibition and response-slowing on a touch-screen task with ratings of fearful temperament obtained using a traditional method: the HIT. In the HIT, monkeys who showed the greatest degree of whole body freezing and fearful retreat behavior were assessed as having the most fearful temperament. In the present study, monkeys who were most fearful in the HIT also showed the greatest degree of behavioral inhibition (measured as withholding of response) during the touch-screen task when presented with pictures of an unknown conspecific with direct-gaze. The relationship between fearful temperament and behavioral inhibition of response was specific to conspecific faces with direct-gaze as we found no evidence for behavioral inhibition when pictures of a human mask or objects associated with veterinary and husbandry procedures were shown. To our knowledge this is the first study to directly assess the extent to which fearful temperament (as assessed in the HIT) is associated with behavioral inhibition of response during a touch-screen task. We propose that this touch-screen task, in the laboratory context, offers a more standardized and sensitive method for assessing fearful temperament than the traditional HIT.

Our finding for behavioral inhibition of response to direct-gaze faces by monkeys with more fearful temperament in Study 1 supports a large body of literature demonstrating a priority of processing effect for conspecific faces (Adolphs et al., 1996; Parr, 2011). Behavioral inhibition of response occurred to the direct-gaze faces, but not to the mask or objects, or the presumably neutral control stimulus (gray square). This finding suggests that behavioral inhibition to direct-gaze faces was driven by the negative emotional content of the face stimuli, and that the touch-screen task is sensitive to inhibition of response to threatening social stimuli. Direct eye-contact by an unknown male is a highly salient signal of dominance and social threat in rhesus macaques (Maestripieri, 1997). The lack of behavioral inhibition to presentations of the human mask suggests that behavioral inhibition is elicited by conspecific faces specifically, and not faces in general.

Our finding for response-slowing only to object stimuli in Study 2 highlights the need to establish the appropriate level analysis for any given species, captive setting and set of stimuli. Monkeys were slower to touch pictures of objects associated with husbandry and veterinary procedures relative to gray squares, and relative to a picture of the mask used during the HIT. This suggests that objects may have greater negative valence for the monkeys than the mask. It is also possible that this finding is an artifact of the use of a single picture of the mask leading to speeded habituation masking any initial inhibition or response-slowing.

There was evidence for habituation to negative stimuli over repeated trials in both studies. Behavioral inhibition of response to direct-gaze conspecific faces occurred most often during the first three presentations in Study 1, as did response-slowing for objects (and mask) in Study 2. Habituation to emotional cues is known to occur when stimuli are presented multiple times within a test session (Denny et al., 2014) and can present issues for analysis of data, especially when effects are small (Nanhoe-Mahabier et al., 2012). We included trial number as a control variable and random slope in our models to control for potential habituation effects of repeated presentations of test trials. We recommend maintaining this information in analyses by retaining some aspect of time or trial number as a control variable. An issue we are currently looking at in a separate study is the extent to which habituation across the course of a session masks patterns of affective responding, a consideration for experimental design in both the human and animal literatures (Nanhoe-Mahabier et al., 2012).

A direction for future research is to establish the sensitivity of the touch-screen task to state and trait affect, and to identify how the influence of the two on performance may be teased apart. Both Bethell et al. (2016) and Cronin et al. (2018) found response-slowing to direct-gaze faces in *Macaca* spp. during presumably negative emotional states (following a veterinary check, and during a noisy public event in a zoo), compared to presumably neutral emotional states in the absence of any known stressors. The current study differs methodologically from Cronin et al. (2018) who tested Japanese macaques in their social group and Bethell et al. (2016), who tested singly housed rhesus macaques. Our study population were temporarily separated from their group members for testing, a procedure which is a daily occurrence for experimental purposes and to which the monkeys had been habituated over time. It is not known to what extent this separation may still result in elevated stress for some individuals. Since the focus of the current study was not to test for changes in affective state, we did not manipulate, measure or control for current emotional state. Given the significant relationship between freeze-fear score and behavioral inhibition to direct-gaze faces, it is unlikely that state affect masked trait fearfulness in Study 1. It may even have enhanced it. Studies with humans indicate that behavioral inhibition on go/no-go tasks is greatest amongst clinical populations when patients are under stress (Mkrtchian et al., 2017). For example, participants diagnosed with negative mood disorders more accurately inhibit responding on a cognitive task when threatened with unpredictable shock, suggesting state stress enhances behavioral inhibition (Mkrtchian et al., 2017). Aylward et al. (2017), however, failed to find an effect of threat of unpredictable shock on behavioral inhibition in a non-clinical sample, indicating that behavioral inhibition under stress identifies clinical, but not sub-clinical, populations. We therefore might expect not to see behavioral inhibition in a (presumably) sub-clinical group of monkeys under baseline conditions. Anecdotally, the two monkeys (Bex and Zaz) who had been temporarily moved into singly housing prior to the start of Study 1 (and were therefore excluded from the analysis for Study 1) touched only one direct-gaze face between them (although both responded above criterion on control trials). Both had mid-range scores for fearful temperament, and a good level of performance during Study 2 when they were socially housed. It is therefore possible this result arose from a negative affective state associated with single housing or the events leading up to it, an interpretation in line with the findings of Bethell et al. (2016). Encouragingly both animals continued working throughout Study 1, as did all other monkeys, avoiding problems of a self-selecting sample. Throughout both studies only one monkey failed to reach criterion on control trials during testing (Fla, Study 2). Since this individual had the lowest freeze-fear score of all animals tested, and a high response rate during Study 1, it does not appear that this poor performance in Study 2 is due to temperament, but possibly due to transient extraneous factors not accounted for here. With trained animals in the laboratory context, the task proposed here has a high completion rate and is unlikely to be restricted to a self-selecting sample. Self-selection may be a consideration in other context such as zoos (e.g., Cronin et al., 2018).

Another future direction for research is to establish sensitivity of responses on the touch-screen task to physiological arousal. Monkeys who found the test stimuli most negative may also have experienced the greatest increases in arousal. Arousal has a non-linear effect on response speed, leading to both speeding and slowing of response depending on where on the arousal curve an individual is (Mendl, 1999). In human psychological research, stimulus valence effects are greatest when stimuli are shown in blocks containing stimuli of just one valence type, where cumulative carry-over effects on physiological arousal occur with repeated presentations (Bradley et al., 1996; Frings et al., 2010). These cumulative effects can be lost when stimuli of different valence are shown within the same block (Algom et al., 2004). We attempted to minimize cumulative arousal effects in the current study by including filler trials in which infants and fruits were shown, although we did not collect physiological data to validate any effect on arousal. Presenting stimuli in a block design with a single-valence shown in each block may reveal response-slowing where the effect to be detected is small and dependent on cumulative arousal.

Additional factors that may have influenced performance on the touch-screen task include mismatching effects (e.g., touch a negative stimulus to gain a positive reward, which has been shown to exacerbate the impact of transient emotion states on task performance (Raoult et al., 2017), and influence of attentional processes (Bar-Haim et al., 2007). For example, Aylward et al. (2017) found faster responses by human participants taking part in a computer task to fearful faces compared to happy faces, which was independent of affective state (threat of electric shock). The authors attributed this speeding of response to attentional capture by negative stimuli. Fox et al. (2001) were able to rule out attentional capture effects for response speed to targets following negative stimuli in their exogenous cueing task by adjusting the relative location of the negative stimuli and neutral targets. Adjusting the temporal and spatial synchrony of cues and targets can therefore help elucidate the relative contribution of different mechanisms underlying responses to cues presented during computer tasks. Furthermore, with the current cohort we did not have data available to allow us to account for latent variables such as relatedness, genotype, early rearing environment, age of removal from the mother, and social rank, all of which reflect the unique history of gene–environmental interactions for each animal (Würbel, 2002). These shape the development of fearful temperament and influence responses to social and negative stimuli in cognitive tasks (Jones et al., 1992; Lang et al., 2000; Buss et al., 2004; Rogers et al., 2008; Corcoran et al., 2012; Gottlieb and Capitanio, 2013; Coleman and Pierre, 2014).

The current study sheds light on previous work. Bethell et al. (2016) presented direct-gaze conspecific face stimuli to adult male *M. mulatta* for 60 s or until touched. In that study it was not possible to distinguish whether monkeys learned that touching a stimulus made it disappear, leading to faster responses in animals who found the stimuli aversive, but not too aversive to touch. In the current study, we inhibited learning that touching a stimulus made it disappear by shortening and fixing the stimulus presentation time to 3 or 1 s. A touch response in the current study therefore should more accurately reflect early emotional response to the stimulus independent of later executive processes that may have subsequently influenced decision to respond in Bethell et al. (2016).

There is a growing call for new technologies and automated systems for standardizing and refining methods across facilities (Buchanan-Smith, 2006; Prescott et al., 2017; Berger et al., 2018). We used a touch-screen apparatus with which monkeys were familiar having used it on a daily basis (Calapai et al., 2017) that was programmed with open source software^2^ to automatically collect data for two objective measures of response (proportion of touches and speed to touch). Familiarity with apparatus and reinforcement contingencies associated with working for rewards in laboratory settings should reduce stress compared with the more ambiguous context of the HIT. The use of pictorial stimuli (which can be manipulated in terms of emotional intensity and to incorporate facility-specific imagery) provides opportunity for increased sensitivity compared to the HIT. For example, in a stranger approach test with 3-year-old children, Buss et al. (2004) found that only children who exhibited fearful response in the mildest of four fearful contexts (stranger approach in which the child could move away and seek comfort from the mother) also showed increased salivary cortisol and were therefore characterized as having dysregulated fear. Here, conspecific faces with direct gaze met this criterion for three of the monkeys who showed highest levels of freeze and fear response during the HIT. Whether the touch-screen task could be adapted for use with younger monkeys (some groups conduct the HIT as standard at ∼107 days old: Capitanio, 2011) is unclear. Ultimately, the reliable analysis of individual differences in expression and regulation of emotion such as fearful temperament will require testing of multiple potential underlying mechanisms across multiple contexts.

In summary, the touch-screen task presented here with male rhesus macaques was sensitive to behavioral inhibition of response to negative conspecific faces, and slowing of responses to pictures of objects associated with husbandry and veterinary procedures. Behavioral inhibition to direct-gaze conspecific faces on the touch-screen task was greatest in monkeys who showed most freezing and fearful behaviors during the HIT, suggesting that the task, when run using highly salient negative picture stimuli, is sensitive to fearful temperament. It is possible the behavioral inhibition reflects an early and subtle pre-cursor component of the freeze response evident in the HIT. Altogether, we conclude that relatively simple touch-screen tasks like that presented here show promise for developing standardized and sensitive tests of temperament, negative affect and underlying mechanisms. Further work is needed to assess the reproducibility and generalizability of these findings across species and contexts.

## Ethics Statement

This study complied with institutional guidelines on Animal Care and Use of the German Primate Center and was conducted in accordance with national and international guidelines on the use of primates in research including German Animal Protection Law, the European Union Directive 2010/63/EU on the Protection of Animals used for Scientific Purposes and the Society for Neuroscience Policies on the Use of Animals and Humans in Neuroscience Research. The animals used in this study were all highly accustomed to settings and scenarios experienced in the current study. All animals in this study were also participating in studies that require a governmental permit due to being animal experiments (the permit was issued by the responsible regional government office, Niedersaechsisches Landesamt fuer Verbraucherschutz und Lebensmittelsicherheit, LAVES, under the permit number 3392 42502-04-13/1100). These fall into the category of mild to moderate, according to the severity categorization of Annex VIII of the European Union’s directive 2010/63/EU on the protection of animals used for scientific purposes (see also Pfefferle et al., 2018). Additionally, the study was approved by Liverpool John Moores University Ethical Review Panel under permit number EB/20-184.

## Author Contributions

EB, DP, and RB contributed to the conception and design of the study. LC, DP, and RB collected the data. DP and EB performed statistical analyses. Together all authors interpreted the data. EB wrote the first draft of the manuscript with DP writing sections of it. All authors contributed to the manuscript revision, read and approved the submitted version.

## Conflict of Interest Statement

The authors declare that the research was conducted in the absence of any commercial or financial relationships that could be construed as a potential conflict of interest.

## Footnotes

1 http://mworks-project.org

2 https://mworks.github.io/
