# Supplementary material for: Toward a Standardized Test of Fearful Temperament in Primates: A Sensitive Alternative to the Human Intruder Task for Laboratory-Housed Rhesus Macaques (Macaca mulatta)
Source: Front Psychol. 2019 May 14;10:1051. doi: 10.3389/fpsyg.2019.01051 (PMC6527799; doi:10.3389/fpsyg.2019.01051)
Supplement: Supplementary file 1 [file Data_Sheet_1.pdf]

Supplementary Data Sheet 1. Raw data for Study 1 and Study 2.

row	ID	Age	XBI#	Order	Study	Exp	Trial#	Postn	Stimulus	RT	HIT_FF_Score
1	bex	6.18	7	1	2	mask	1	1	control	781	240.042
2	bex	6.18	7	1	2	mask	2	5	control	747	240.042
3	bex	6.18	7	1	2	mask	3	3	control	516	240.042
5	bex	6.18	7	1	2	mask	5	4	control	489	240.042
6	bex	6.18	7	1	2	mask	6	4	control	581	240.042
7	bex	6.18	7	1	2	mask	7	4	control	538	240.042
8	bex	6.18	7	1	2	mask	8	4	control	603	240.042
9	bex	6.18	7	1	2	mask	9	6	control	568	240.042
11	bex	6.18	7	1	2	mask	11	1	control	598	240.042
12	bex	6.18	7	1	2	mask	12	1	control	528	240.042
13	bex	6.18	7	1	2	mask	13	4	control	469	240.042
14	bex	6.18	7	1	2	mask	14	1	mask_man	NA	240.042
15	bex	6.18	7	1	2	mask	15	4	control	2167	240.042
16	bex	6.18	7	1	2	mask	16	3	control	557	240.042
18	bex	6.18	7	1	2	mask	18	3	control	486	240.042
19	bex	6.18	7	1	2	mask	19	4	mask_man	1149	240.042
20	bex	6.18	7	1	2	mask	20	1	control	526	240.042
21	bex	6.18	7	1	2	mask	21	5	control	594	240.042
22	bex	6.18	7	1	2	mask	22	5	control	434	240.042
24	bex	6.18	7	1	2	mask	24	6	control	530	240.042
25	bex	6.18	7	1	2	mask	25	4	control	456	240.042
27	bex	6.18	7	1	2	mask	27	5	control	621	240.042
28	bex	6.18	7	1	2	mask	28	5	control	492	240.042
30	bex	6.18	7	1	2	mask	30	4	control	478	240.042
31	bex	6.18	7	1	2	mask	31	3	control	471	240.042
32	bex	6.18	7	1	2	mask	32	1	mask_man	926	240.042
33	bex	6.18	7	1	2	mask	33	2	control	439	240.042
34	bex	6.18	7	1	2	mask	34	6	control	374	240.042
35	bex	6.18	7	1	2	mask	35	1	control	480	240.042
37	bex	6.18	7	1	2	mask	37	3	control	421	240.042
38	bex	6.18	7	1	2	mask	38	3	mask_man	918	240.042
39	bex	6.18	7	1	2	mask	39	1	control	520	240.042
40	bex	6.18	7	1	2	mask	40	3	control	388	240.042
41	bex	6.18	7	1	2	mask	41	4	mask_man	877	240.042
42	bex	6.18	7	1	2	mask	42	6	control	501	240.042
43	bex	6.18	7	1	2	mask	43	2	control	441	240.042
45	bex	6.18	7	1	2	mask	45	2	control	567	240.042
46	bex	6.18	7	1	2	mask	46	5	mask_man	794	240.042
47	bex	6.18	7	1	2	mask	47	2	control	490	240.042
48	bex	6.18	7	1	2	mask	48	2	control	596	240.042
49	bex	6.18	7	1	2	mask	49	6	mask_man	1227	240.042
50	bex	6.18	7	1	2	mask	50	5	control	1327	240.042
52	bex	6.18	7	1	2	mask	52	2	control	473	240.042
53	bex	6.18	7	1	2	mask	53	2	control	469	240.042
54	bex	6.18	7	1	2	mask	54	2	control	448	240.042
55	bex	6.18	7	1	2	mask	55	5	mask_man	1020	240.042
56	bex	6.18	7	1	2	mask	56	5	control	574	240.042
57	bex	6.18	7	1	2	mask	57	3	control	556	240.042

58	bex	6.18	7	1	2	mask	58	3	mask_man	953	240.042
59	bex	6.18	7	1	2	mask	59	5	control	389	240.042
60	bex	6.18	7	1	2	mask	60	1	control	NA	240.042
61	bex	6.18	7	1	2	mask	61	4	control	807	240.042
62	bex	6.18	7	1	2	mask	62	4	mask_man	2069	240.042
63	bex	6.18	7	1	2	mask	63	6	control	501	240.042
64	bex	6.18	7	1	2	mask	64	2	control	452	240.042
65	bex	6.18	7	1	2	mask	65	2	mask_man	648	240.042
66	bex	6.18	7	1	2	mask	66	5	control	534	240.042
67	bex	6.18	7	1	2	mask	67	3	control	508	240.042
68	bex	6.18	7	1	2	mask	68	1	control	468	240.042
70	bex	6.18	7	1	2	mask	70	1	control	419	240.042
72	bex	6.18	7	1	2	mask	72	4	control	531	240.042
74	bex	6.18	7	1	2	mask	74	6	mask_man	1223	240.042
76	bex	6.18	7	1	2	mask	76	4	control	1192	240.042
77	bex	6.18	7	1	2	mask	77	3	control	559	240.042
79	bex	6.18	7	1	2	mask	79	1	control	500	240.042
80	bex	6.18	7	1	2	mask	80	2	mask_man	999	240.042
81	bex	6.18	7	1	2	mask	81	3	control	557	240.042
83	bex	6.18	7	1	2	mask	83	5	control	950	240.042
84	bex	6.18	7	1	2	mask	84	4	control	NA	240.042
85	bex	6.18	7	1	2	mask	85	5	mask_man	820	240.042
88	bex	6.18	7	1	2	mask	88	6	control	608	240.042
90	bex	6.18	7	1	2	mask	90	3	control	499	240.042
91	bex	6.18	7	1	2	mask	91	5	control	377	240.042
94	bex	6.18	7	1	2	mask	94	6	control	835	240.042
96	bex	6.18	7	1	2	mask	96	6	control	NA	240.042
97	bex	6.18	7	1	2	mask	97	4	control	2072	240.042
98	bex	6.18	7	1	2	mask	98	4	control	488	240.042
99	bex	6.18	7	1	2	mask	99	6	mask_man	NA	240.042
100	bex	6.18	7	1	2	mask	100	5	control	517	240.042
104	bex	6.18	7	1	2	mask	104	6	control	2455	240.042
105	bex	6.18	7	1	2	mask	105	6	control	NA	240.042
106	bex	6.18	7	1	2	mask	106	1	control	582	240.042
107	bex	6.18	7	1	2	mask	107	3	mask_man	1034	240.042
111	bex	6.18	7	1	2	mask	111	2	control	831	240.042
116	bex	6.18	7	1	2	mask	116	6	control	NA	240.042
117	bex	6.18	7	1	2	mask	117	1	control	NA	240.042
119	bex	6.18	7	1	2	mask	119	6	control	NA	240.042
120	bex	6.18	7	1	2	mask	120	2	mask_man	934	240.042
124	bex	6.18	7	1	2	mask	124	2	control	NA	240.042
125	bex	6.18	7	1	2	mask	125	3	control	597	240.042
127	bex	6.18	7	1	2	mask	127	2	control	634	240.042
128	bex	6.18	7	1	2	mask	128	1	mask_man	2226	240.042
129	bex	6.18	7	1	2	mask	129	1	control	1435	240.042
130	bex	6.18	7	1	2	mask	130	3	control	497	240.042
131	bex	6.18	7	1	2	mask	131	5	control	562	240.042
133	bex	6.18	7	1	2	mask	133	2	control	504	240.042
134	bex	6.18	7	1	2	mask	134	1	control	NA	240.042
136	bex	6.18	7	1	2	mask	136	1	control	NA	240.042

137	bex	6.18	7	1	2	mask	137	2	control	NA	240.042
138	bex	6.18	7	1	2	mask	138	1	control	558	240.042
139	bex	6.18	7	1	2	object	1	2	control	474	240.042
140	bex	6.18	7	1	2	object	2	2	control	584	240.042
141	bex	6.18	7	1	2	object	3	3	control	456	240.042
148	bex	6.18	7	1	2	object	10	6	control	465	240.042
149	bex	6.18	7	1	2	object	11	1	control	470	240.042
150	bex	6.18	7	1	2	object	12	4	control	433	240.042
151	bex	6.18	7	1	2	object	13	3	control	486	240.042
154	bex	6.18	7	1	2	object	16	6	control	435	240.042
155	bex	6.18	7	1	2	object	17	4	net	NA	240.042
156	bex	6.18	7	1	2	object	18	3	control	522	240.042
157	bex	6.18	7	1	2	object	19	6	control	486	240.042
159	bex	6.18	7	1	2	object	21	6	control	380	240.042
160	bex	6.18	7	1	2	object	22	1	control	472	240.042
161	bex	6.18	7	1	2	object	23	2	control	466	240.042
163	bex	6.18	7	1	2	object	25	6	control	452	240.042
164	bex	6.18	7	1	2	object	26	2	control	432	240.042
165	bex	6.18	7	1	2	object	27	3	control	475	240.042
166	bex	6.18	7	1	2	object	28	1	net	1215	240.042
167	bex	6.18	7	1	2	object	29	6	control	NA	240.042
168	bex	6.18	7	1	2	object	30	1	control	518	240.042
169	bex	6.18	7	1	2	object	31	1	control	571	240.042
171	bex	6.18	7	1	2	object	33	5	control	739	240.042
172	bex	6.18	7	1	2	object	34	2	control	535	240.042
173	bex	6.18	7	1	2	object	35	2	control	521	240.042
175	bex	6.18	7	1	2	object	37	4	control	568	240.042
176	bex	6.18	7	1	2	object	38	6	glove	NA	240.042
177	bex	6.18	7	1	2	object	39	2	control	503	240.042
178	bex	6.18	7	1	2	object	40	4	control	520	240.042
179	bex	6.18	7	1	2	object	41	6	broom	1713	240.042
180	bex	6.18	7	1	2	object	42	2	control	483	240.042
181	bex	6.18	7	1	2	object	43	3	control	623	240.042
184	bex	6.18	7	1	2	object	46	5	control	492	240.042
186	bex	6.18	7	1	2	object	48	5	control	671	240.042
188	bex	6.18	7	1	2	object	50	5	control	468	240.042
189	bex	6.18	7	1	2	object	51	5	control	448	240.042
191	bex	6.18	7	1	2	object	53	3	control	492	240.042
194	bex	6.18	7	1	2	object	56	1	control	513	240.042
196	bex	6.18	7	1	2	object	58	4	control	585	240.042
198	bex	6.18	7	1	2	object	60	5	control	388	240.042
199	bex	6.18	7	1	2	object	61	3	control	486	240.042
200	bex	6.18	7	1	2	object	62	4	control	769	240.042
202	bex	6.18	7	1	2	object	64	5	control	418	240.042
205	bex	6.18	7	1	2	object	67	1	glove	2233	240.042
209	bex	6.18	7	1	2	object	71	6	control	NA	240.042
211	bex	6.18	7	1	2	object	73	3	control	479	240.042
212	bex	6.18	7	1	2	object	74	2	net	NA	240.042
213	bex	6.18	7	1	2	object	75	3	control	506	240.042
216	bex	6.18	7	1	2	object	78	1	control	823	240.042

217	bex	6.18	7	1	2	object	79	4	broom	1819	240.042
218	bex	6.18	7	1	2	object	80	2	control	674	240.042
219	bex	6.18	7	1	2	object	81	6	control	453	240.042
220	bex	6.18	7	1	2	object	82	6	control	488	240.042
222	bex	6.18	7	1	2	object	84	2	control	532	240.042
223	bex	6.18	7	1	2	object	85	3	control	662	240.042
224	bex	6.18	7	1	2	object	86	4	control	538	240.042
225	bex	6.18	7	1	2	object	87	5	net	NA	240.042
228	bex	6.18	7	1	2	object	90	5	control	2503	240.042
229	bex	6.18	7	1	2	object	91	3	net	2650	240.042
231	bex	6.18	7	1	2	object	93	4	control	1362	240.042
232	bex	6.18	7	1	2	object	94	5	control	658	240.042
233	bex	6.18	7	1	2	object	95	5	control	635	240.042
237	bex	6.18	7	1	2	object	99	3	control	NA	240.042
238	bex	6.18	7	1	2	object	100	3	control	628	240.042
239	bex	6.18	7	1	2	object	101	5	broom	1252	240.042
240	bex	6.18	7	1	2	object	102	6	control	505	240.042
241	bex	6.18	7	1	2	object	103	1	control	NA	240.042
242	bex	6.18	7	1	2	object	104	4	control	573	240.042
243	bex	6.18	7	1	2	object	105	6	net	1292	240.042
249	bex	6.18	7	1	2	object	111	1	control	1990	240.042
250	bex	6.18	7	1	2	object	112	1	control	NA	240.042
251	bex	6.18	7	1	2	object	113	1	control	NA	240.042
252	bex	6.18	7	1	2	object	114	2	broom	NA	240.042
253	bex	6.18	7	1	2	object	115	5	control	NA	240.042
258	bex	6.18	7	1	2	object	120	2	control	NA	240.042
259	bex	6.18	7	1	2	object	121	1	control	NA	240.042
260	bex	6.18	7	1	2	object	122	3	broom	NA	240.042
265	bex	6.18	7	1	2	object	127	4	control	NA	240.042
266	bex	6.18	7	1	2	object	128	5	glove	793	240.042
274	bex	6.18	7	1	2	object	136	6	control	918	240.042
275	bex	6.18	7	1	2	object	137	2	control	748	240.042
277	bex	6.18	7	1	2	object	139	6	control	490	240.042
278	bex	6.18	7	1	2	object	140	4	glove	996	240.042
279	bex	6.18	7	1	2	object	141	1	control	574	240.042
302	bex	6.18	7	1	2	object	164	5	control	NA	240.042
303	bex	6.18	7	1	2	object	165	3	control	NA	240.042
305	bex	6.18	7	1	2	object	167	1	broom	NA	240.042
313	bex	6.18	7	1	2	object	175	2	control	NA	240.042
343	bex	6.18	7	1	2	object	205	1	control	NA	240.042
344	bex	6.18	7	1	2	object	206	4	control	NA	240.042
356	bex	6.18	7	1	2	object	218	4	control	NA	240.042
357	bex	6.18	7	1	2	object	219	2	control	NA	240.042
358	bex	6.18	7	1	2	object	220	2	glove	2129	240.042
370	bex	6.18	7	1	2	object	232	6	control	NA	240.042
385	bex	6.18	7	1	2	object	247	4	control	NA	240.042
386	bex	6.18	7	1	2	object	248	3	glove	1102	240.042
387	bex	6.18	7	1	2	object	249	2	control	590	240.042
389	bex	6.18	7	1	2	object	251	3	control	472	240.042
445	bex	6.18	7	1	2	object	307	2	control	NA	240.042

477	bex	6.18	7	1	2	object	339	1	control	NA	240.042
478	bex	6.18	7	1	2	object	340	6	control	637	240.042
479	cas	7.28	1	2	2	object	1	1	control	585	430.331
482	cas	7.28	1	2	2	object	4	1	control	532	430.331
483	cas	7.28	1	2	2	object	5	2	control	542	430.331
487	cas	7.28	1	2	2	object	9	6	control	655	430.331
488	cas	7.28	1	2	2	object	10	4	control	602	430.331
489	cas	7.28	1	2	2	object	11	2	control	444	430.331
492	cas	7.28	1	2	2	object	14	4	control	678	430.331
496	cas	7.28	1	2	2	object	18	2	control	515	430.331
502	cas	7.28	1	2	2	object	24	2	control	580	430.331
504	cas	7.28	1	2	2	object	26	3	control	529	430.331
507	cas	7.28	1	2	2	object	29	4	control	700	430.331
509	cas	7.28	1	2	2	object	31	1	control	471	430.331
510	cas	7.28	1	2	2	object	32	2	control	494	430.331
511	cas	7.28	1	2	2	object	33	1	control	488	430.331
512	cas	7.28	1	2	2	object	34	2	broom	659	430.331
517	cas	7.28	1	2	2	object	39	4	control	585	430.331
518	cas	7.28	1	2	2	object	40	5	control	450	430.331
519	cas	7.28	1	2	2	object	41	4	control	520	430.331
524	cas	7.28	1	2	2	object	46	1	control	544	430.331
525	cas	7.28	1	2	2	object	47	5	control	412	430.331
526	cas	7.28	1	2	2	object	48	4	control	558	430.331
528	cas	7.28	1	2	2	object	50	4	control	466	430.331
529	cas	7.28	1	2	2	object	51	6	glove	526	430.331
530	cas	7.28	1	2	2	object	52	6	control	523	430.331
534	cas	7.28	1	2	2	object	56	3	control	637	430.331
535	cas	7.28	1	2	2	object	57	5	control	458	430.331
536	cas	7.28	1	2	2	object	58	5	net	737	430.331
537	cas	7.28	1	2	2	object	59	4	control	488	430.331
538	cas	7.28	1	2	2	object	60	3	control	517	430.331
539	cas	7.28	1	2	2	object	61	6	control	447	430.331
544	cas	7.28	1	2	2	object	66	3	control	566	430.331
545	cas	7.28	1	2	2	object	67	4	control	392	430.331
547	cas	7.28	1	2	2	object	69	1	control	692	430.331
551	cas	7.28	1	2	2	object	73	5	glove	674	430.331
552	cas	7.28	1	2	2	object	74	1	control	545	430.331
553	cas	7.28	1	2	2	object	75	3	net	668	430.331
555	cas	7.28	1	2	2	object	77	5	control	575	430.331
557	cas	7.28	1	2	2	object	79	3	control	528	430.331
561	cas	7.28	1	2	2	object	83	2	control	458	430.331
563	cas	7.28	1	2	2	object	85	2	control	477	430.331
564	cas	7.28	1	2	2	object	86	4	glove	429	430.331
565	cas	7.28	1	2	2	object	87	3	control	1157	430.331
570	cas	7.28	1	2	2	object	92	6	control	603	430.331
571	cas	7.28	1	2	2	object	93	4	broom	519	430.331
572	cas	7.28	1	2	2	object	94	6	control	434	430.331
578	cas	7.28	1	2	2	object	100	1	control	498	430.331
579	cas	7.28	1	2	2	object	101	2	control	454	430.331
580	cas	7.28	1	2	2	object	102	6	broom	587	430.331

585	cas	7.28	1	2	2	object	107	1	control	512	430.331
587	cas	7.28	1	2	2	object	109	5	control	465	430.331
589	cas	7.28	1	2	2	object	111	6	control	435	430.331
590	cas	7.28	1	2	2	object	112	2	net	546	430.331
591	cas	7.28	1	2	2	object	113	2	control	529	430.331
601	cas	7.28	1	2	2	object	123	6	control	564	430.331
602	cas	7.28	1	2	2	object	124	4	control	553	430.331
608	cas	7.28	1	2	2	object	130	2	control	2701	430.331
609	cas	7.28	1	2	2	object	131	1	glove	565	430.331
615	cas	7.28	1	2	2	object	137	6	control	496	430.331
616	cas	7.28	1	2	2	object	138	3	control	504	430.331
617	cas	7.28	1	2	2	object	139	2	control	445	430.331
619	cas	7.28	1	2	2	object	141	3	control	455	430.331
620	cas	7.28	1	2	2	object	142	3	control	495	430.331
624	cas	7.28	1	2	2	object	146	3	control	519	430.331
626	cas	7.28	1	2	2	object	148	6	control	429	430.331
627	cas	7.28	1	2	2	object	149	5	control	514	430.331
628	cas	7.28	1	2	2	object	150	3	glove	505	430.331
629	cas	7.28	1	2	2	object	151	4	control	530	430.331
630	cas	7.28	1	2	2	object	152	1	control	416	430.331
631	cas	7.28	1	2	2	object	153	2	control	407	430.331
632	cas	7.28	1	2	2	object	154	1	net	605	430.331
633	cas	7.28	1	2	2	object	155	5	control	483	430.331
639	cas	7.28	1	2	2	object	161	2	control	708	430.331
641	cas	7.28	1	2	2	object	163	5	control	534	430.331
642	cas	7.28	1	2	2	object	164	3	broom	610	430.331
643	cas	7.28	1	2	2	object	165	6	control	394	430.331
644	cas	7.28	1	2	2	object	166	1	control	468	430.331
645	cas	7.28	1	2	2	object	167	5	broom	418	430.331
650	cas	7.28	1	2	2	object	172	5	control	403	430.331
651	cas	7.28	1	2	2	object	173	1	control	641	430.331
654	cas	7.28	1	2	2	object	176	5	control	496	430.331
655	cas	7.28	1	2	2	object	177	6	net	555	430.331
656	cas	7.28	1	2	2	object	178	4	control	429	430.331
657	cas	7.28	1	2	2	object	179	3	control	506	430.331
658	cas	7.28	1	2	2	object	180	4	control	463	430.331
659	cas	7.28	1	2	2	object	181	4	net	502	430.331
660	cas	7.28	1	2	2	object	182	1	control	491	430.331
662	cas	7.28	1	2	2	object	184	6	control	476	430.331
664	cas	7.28	1	2	2	object	186	5	control	366	430.331
669	cas	7.28	1	2	2	object	191	1	broom	498	430.331
671	cas	7.28	1	2	2	object	193	1	control	627	430.331
672	cas	7.28	1	2	2	object	194	6	control	481	430.331
673	cas	7.28	1	2	2	object	195	2	glove	476	430.331
674	cas	7.28	1	2	2	object	196	6	control	618	430.331
677	cas	7.28	1	2	2	object	199	3	control	565	430.331
678	cas	7.28	1	2	2	object	200	5	control	366	430.331
680	cas	7.28	1	2	2	object	202	3	control	489	430.331
681	cas	7.28	1	2	2	object	203	3	control	429	430.331
685	cas	7.28	1	2	2	object	207	4	control	386	430.331

688	cas	7.28	1	2	2	object	210	4	control	415	430.331
690	cas	7.28	1	2	2	object	212	5	control	428	430.331
691	cas	7.28	1	2	2	mask	1	5	control	606	430.331
692	cas	7.28	1	2	2	mask	2	3	control	403	430.331
695	cas	7.28	1	2	2	mask	5	3	control	561	430.331
698	cas	7.28	1	2	2	mask	8	4	control	609	430.331
699	cas	7.28	1	2	2	mask	9	2	control	426	430.331
700	cas	7.28	1	2	2	mask	10	4	control	441	430.331
702	cas	7.28	1	2	2	mask	12	4	control	688	430.331
703	cas	7.28	1	2	2	mask	13	5	mask_man	513	430.331
704	cas	7.28	1	2	2	mask	14	4	control	493	430.331
705	cas	7.28	1	2	2	mask	15	2	control	485	430.331
709	cas	7.28	1	2	2	mask	19	1	control	645	430.331
710	cas	7.28	1	2	2	mask	20	1	control	503	430.331
712	cas	7.28	1	2	2	mask	22	3	control	559	430.331
714	cas	7.28	1	2	2	mask	24	3	control	419	430.331
715	cas	7.28	1	2	2	mask	25	6	mask_man	440	430.331
716	cas	7.28	1	2	2	mask	26	1	control	465	430.331
717	cas	7.28	1	2	2	mask	27	5	control	494	430.331
718	cas	7.28	1	2	2	mask	28	5	control	375	430.331
719	cas	7.28	1	2	2	mask	29	5	mask_man	458	430.331
722	cas	7.28	1	2	2	mask	32	3	control	992	430.331
724	cas	7.28	1	2	2	mask	34	6	control	484	430.331
726	cas	7.28	1	2	2	mask	36	3	control	385	430.331
730	cas	7.28	1	2	2	mask	40	3	control	474	430.331
732	cas	7.28	1	2	2	mask	42	4	control	418	430.331
733	cas	7.28	1	2	2	mask	43	2	mask_man	510	430.331
734	cas	7.28	1	2	2	mask	44	6	control	526	430.331
735	cas	7.28	1	2	2	mask	45	2	control	528	430.331
740	cas	7.28	1	2	2	mask	50	1	control	500	430.331
742	cas	7.28	1	2	2	mask	52	5	control	452	430.331
744	cas	7.28	1	2	2	mask	54	6	control	591	430.331
747	cas	7.28	1	2	2	mask	57	2	mask_man	407	430.331
748	cas	7.28	1	2	2	mask	58	1	control	443	430.331
749	cas	7.28	1	2	2	mask	59	4	control	410	430.331
752	cas	7.28	1	2	2	mask	62	1	mask_man	471	430.331
753	cas	7.28	1	2	2	mask	63	2	control	634	430.331
754	cas	7.28	1	2	2	mask	64	6	control	492	430.331
756	cas	7.28	1	2	2	mask	66	2	control	392	430.331
757	cas	7.28	1	2	2	mask	67	3	mask_man	643	430.331
758	cas	7.28	1	2	2	mask	68	3	control	401	430.331
759	cas	7.28	1	2	2	mask	69	6	control	380	430.331
760	cas	7.28	1	2	2	mask	70	4	control	448	430.331
767	cas	7.28	1	2	2	mask	77	2	mask_man	458	430.331
768	cas	7.28	1	2	2	mask	78	3	control	427	430.331
769	cas	7.28	1	2	2	mask	79	3	control	413	430.331
776	cas	7.28	1	2	2	mask	86	5	control	376	430.331
778	cas	7.28	1	2	2	mask	88	2	control	518	430.331
779	cas	7.28	1	2	2	mask	89	2	control	444	430.331
780	cas	7.28	1	2	2	mask	90	6	control	405	430.331

782	cas	7.28	1	2	2	mask	92	1	control	421	430.331
783	cas	7.28	1	2	2	mask	93	5	control	429	430.331
784	cas	7.28	1	2	2	mask	94	2	control	476	430.331
796	cas	7.28	1	2	2	mask	106	2	control	616	430.331
802	cas	7.28	1	2	2	mask	112	4	control	376	430.331
803	cas	7.28	1	2	2	mask	113	5	control	383	430.331
804	cas	7.28	1	2	2	mask	114	4	mask_man	445	430.331
805	cas	7.28	1	2	2	mask	115	1	control	461	430.331
806	cas	7.28	1	2	2	mask	116	5	control	459	430.331
809	cas	7.28	1	2	2	mask	119	6	control	433	430.331
810	cas	7.28	1	2	2	mask	120	1	mask_man	534	430.331
814	cas	7.28	1	2	2	mask	124	5	control	1801	430.331
816	cas	7.28	1	2	2	mask	126	5	control	394	430.331
817	cas	7.28	1	2	2	mask	127	4	mask_man	421	430.331
834	cas	7.28	1	2	2	mask	144	6	control	620	430.331
835	cas	7.28	1	2	2	mask	145	4	control	452	430.331
836	cas	7.28	1	2	2	mask	146	3	mask_man	516	430.331
837	cas	7.28	1	2	2	mask	147	1	control	436	430.331
838	cas	7.28	1	2	2	mask	148	3	control	499	430.331
839	cas	7.28	1	2	2	mask	149	6	mask_man	391	430.331
840	cas	7.28	1	2	2	mask	150	1	control	420	430.331
841	cas	7.28	1	2	2	mask	151	3	control	457	430.331
842	cas	7.28	1	2	2	mask	152	4	mask_man	713	430.331
843	cas	7.28	1	2	2	mask	153	6	control	417	430.331
847	cas	7.28	1	2	2	mask	157	1	mask_man	429	430.331
849	cas	7.28	1	2	2	mask	159	2	control	605	430.331
859	cas	7.28	1	2	2	mask	169	5	control	383	430.331
861	cas	7.28	1	2	2	mask	171	3	control	473	430.331
862	cas	7.28	1	2	2	mask	172	5	mask_man	391	430.331
863	cas	7.28	1	2	2	mask	173	5	control	383	430.331
864	cas	7.28	1	2	2	mask	174	2	control	398	430.331
867	cas	7.28	1	2	2	mask	177	4	control	493	430.331
869	cas	7.28	1	2	2	mask	179	6	control	420	430.331
870	cas	7.28	1	2	2	mask	180	4	control	417	430.331
872	cas	7.28	1	2	2	mask	182	4	control	410	430.331
873	cas	7.28	1	2	2	mask	183	5	control	441	430.331
875	cas	7.28	1	2	2	mask	185	3	control	596	430.331
876	cas	7.28	1	2	2	mask	186	2	control	408	430.331
877	cas	7.28	1	2	2	mask	187	1	control	448	430.331
881	cas	7.28	1	2	2	mask	191	1	control	724	430.331
882	cas	7.28	1	2	2	mask	192	1	control	413	430.331
883	cas	7.28	1	2	2	mask	193	6	control	455	430.331
884	cas	7.28	1	2	2	mask	194	6	mask_man	429	430.331
885	cas	7.28	1	2	2	mask	195	4	control	385	430.331
886	cas	7.28	1	2	2	mask	196	6	control	491	430.331
887	cas	7.28	1	2	2	mask	197	2	control	502	430.331
888	cas	7.28	1	2	2	mask	198	3	mask_man	466	430.331
889	cas	7.28	1	2	2	mask	199	6	control	378	430.331
890	cas	7.28	1	2	2	mask	200	1	control	614	430.331
894	cas	7.28	1	2	2	mask	204	3	control	642	430.331

895	cas	7.28	1	2	2	mask	205	6	control	404	430.331
896	cas	7.28	1	2	2	mask	206	1	control	434	430.331
897	cor	10.81	2	2	2	object	1	2	control	472	230.844
909	cor	10.81	2	2	2	object	13	2	control	421	230.844
910	cor	10.81	2	2	2	object	14	5	control	486	230.844
911	cor	10.81	2	2	2	object	15	5	control	645	230.844
913	cor	10.81	2	2	2	object	17	3	control	494	230.844
933	cor	10.81	2	2	2	object	37	4	control	497	230.844
934	cor	10.81	2	2	2	object	38	2	net	519	230.844
940	cor	10.81	2	2	2	object	44	3	control	401	230.844
950	cor	10.81	2	2	2	object	54	6	control	376	230.844
961	cor	10.81	2	2	2	object	65	6	control	465	230.844
962	cor	10.81	2	2	2	object	66	1	glove	503	230.844
963	cor	10.81	2	2	2	object	67	1	control	525	230.844
964	cor	10.81	2	2	2	object	68	6	control	381	230.844
965	cor	10.81	2	2	2	object	69	5	broom	444	230.844
966	cor	10.81	2	2	2	object	70	2	control	483	230.844
973	cor	10.81	2	2	2	object	77	6	control	396	230.844
974	cor	10.81	2	2	2	object	78	6	broom	483	230.844
975	cor	10.81	2	2	2	object	79	1	control	475	230.844
977	cor	10.81	2	2	2	object	81	4	control	396	230.844
988	cor	10.81	2	2	2	object	92	2	control	526	230.844
989	cor	10.81	2	2	2	object	93	3	control	515	230.844
990	cor	10.81	2	2	2	object	94	6	net	424	230.844
991	cor	10.81	2	2	2	object	95	5	control	414	230.844
992	cor	10.81	2	2	2	object	96	1	control	464	230.844
993	cor	10.81	2	2	2	object	97	4	control	410	230.844
995	cor	10.81	2	2	2	object	99	1	control	457	230.844
996	cor	10.81	2	2	2	object	100	4	control	396	230.844
997	cor	10.81	2	2	2	object	101	6	control	375	230.844
1032	cor	10.81	2	2	2	object	136	3	control	485	230.844
1033	cor	10.81	2	2	2	object	137	2	control	383	230.844
1034	cor	10.81	2	2	2	object	138	1	control	455	230.844
1041	cor	10.81	2	2	2	object	145	6	control	373	230.844
1043	cor	10.81	2	2	2	object	147	4	control	388	230.844
1044	cor	10.81	2	2	2	object	148	4	control	381	230.844
1046	cor	10.81	2	2	2	object	150	2	control	416	230.844
1048	cor	10.81	2	2	2	object	152	1	control	452	230.844
1049	cor	10.81	2	2	2	object	153	5	glove	474	230.844
1050	cor	10.81	2	2	2	object	154	6	control	380	230.844
1054	cor	10.81	2	2	2	object	158	3	control	713	230.844
1055	cor	10.81	2	2	2	object	159	2	control	451	230.844
1056	cor	10.81	2	2	2	object	160	2	broom	437	230.844
1057	cor	10.81	2	2	2	object	161	1	control	472	230.844
1058	cor	10.81	2	2	2	object	162	6	glove	461	230.844
1069	cor	10.81	2	2	2	object	173	2	control	380	230.844
1070	cor	10.81	2	2	2	object	174	4	control	439	230.844
1071	cor	10.81	2	2	2	object	175	4	broom	436	230.844
1072	cor	10.81	2	2	2	object	176	4	control	399	230.844
1073	cor	10.81	2	2	2	object	177	6	control	437	230.844

1074	cor	10.81	2	2	2	object	178	5	control	407	230.844
1075	cor	10.81	2	2	2	object	179	3	broom	431	230.844
1076	cor	10.81	2	2	2	object	180	2	control	401	230.844
1077	cor	10.81	2	2	2	object	181	1	control	407	230.844
1078	cor	10.81	2	2	2	object	182	3	control	388	230.844
1079	cor	10.81	2	2	2	object	183	1	net	421	230.844
1092	cor	10.81	2	2	2	object	196	2	control	399	230.844
1093	cor	10.81	2	2	2	object	197	4	glove	418	230.844
1106	cor	10.81	2	2	2	object	210	3	control	495	230.844
1107	cor	10.81	2	2	2	object	211	4	control	414	230.844
1108	cor	10.81	2	2	2	object	212	2	control	389	230.844
1114	cor	10.81	2	2	2	object	218	5	control	455	230.844
1116	cor	10.81	2	2	2	object	220	3	control	464	230.844
1117	cor	10.81	2	2	2	object	221	5	control	430	230.844
1119	cor	10.81	2	2	2	object	223	4	control	368	230.844
1131	cor	10.81	2	2	2	object	235	6	control	403	230.844
1132	cor	10.81	2	2	2	object	236	3	control	421	230.844
1141	cor	10.81	2	2	2	object	245	5	control	350	230.844
1143	cor	10.81	2	2	2	object	247	5	control	443	230.844
1145	cor	10.81	2	2	2	object	249	5	control	420	230.844
1155	cor	10.81	2	2	2	object	259	1	control	546	230.844
1157	cor	10.81	2	2	2	object	261	4	control	370	230.844
1158	cor	10.81	2	2	2	object	262	1	control	423	230.844
1166	cor	10.81	2	2	2	object	270	5	control	465	230.844
1167	cor	10.81	2	2	2	object	271	5	net	393	230.844
1168	cor	10.81	2	2	2	object	272	1	control	423	230.844
1169	cor	10.81	2	2	2	object	273	6	control	348	230.844
1170	cor	10.81	2	2	2	object	274	3	glove	554	230.844
1171	cor	10.81	2	2	2	object	275	3	control	431	230.844
1172	cor	10.81	2	2	2	object	276	3	control	539	230.844
1173	cor	10.81	2	2	2	object	277	2	glove	417	230.844
1194	cor	10.81	2	2	2	object	298	5	control	318	230.844
1196	cor	10.81	2	2	2	object	300	1	control	544	230.844
1197	cor	10.81	2	2	2	object	301	6	control	408	230.844
1198	cor	10.81	2	2	2	object	302	5	control	407	230.844
1199	cor	10.81	2	2	2	object	303	4	net	546	230.844
1200	cor	10.81	2	2	2	object	304	4	control	412	230.844
1201	cor	10.81	2	2	2	object	305	2	control	405	230.844
1202	cor	10.81	2	2	2	object	306	1	broom	575	230.844
1203	cor	10.81	2	2	2	object	307	3	control	417	230.844
1204	cor	10.81	2	2	2	object	308	2	control	398	230.844
1205	cor	10.81	2	2	2	object	309	6	control	394	230.844
1207	cor	10.81	2	2	2	object	311	2	control	399	230.844
1208	cor	10.81	2	2	2	object	312	3	net	446	230.844
1209	cor	10.81	2	2	2	object	313	3	control	396	230.844
1211	cor	10.81	2	2	2	object	315	4	control	373	230.844
1213	cor	10.81	2	2	2	object	317	1	control	465	230.844
1214	cor	10.81	2	2	2	object	318	6	control	364	230.844
1215	cor	10.81	2	2	2	object	319	5	control	420	230.844
1216	cor	10.81	2	2	2	object	320	2	control	511	230.844

1218	cor	10.81	2	2	2	mask	2	4	control	356	230.844
1226	cor	10.81	2	2	2	mask	10	1	control	444	230.844
1240	cor	10.81	2	2	2	mask	24	2	control	417	230.844
1241	cor	10.81	2	2	2	mask	25	1	control	468	230.844
1243	cor	10.81	2	2	2	mask	27	2	control	467	230.844
1268	cor	10.81	2	2	2	mask	52	4	control	447	230.844
1269	cor	10.81	2	2	2	mask	53	3	mask_man	554	230.844
1270	cor	10.81	2	2	2	mask	54	1	control	424	230.844
1331	cor	10.81	2	2	2	mask	115	3	mask_man	691	230.844
1332	cor	10.81	2	2	2	mask	116	4	control	478	230.844
1333	cor	10.81	2	2	2	mask	117	2	mask_man	543	230.844
1373	cor	10.81	2	2	2	mask	157	3	control	438	230.844
1374	cor	10.81	2	2	2	mask	158	3	control	410	230.844
1408	cor	10.81	2	2	2	mask	192	1	control	516	230.844
1411	cor	10.81	2	2	2	mask	195	5	mask_man	2670	230.844
1412	cor	10.81	2	2	2	mask	196	4	control	374	230.844
1413	cor	10.81	2	2	2	mask	197	5	control	363	230.844
1457	cor	10.81	2	2	2	mask	241	1	mask_man	433	230.844
1480	cor	10.81	2	2	2	mask	264	3	control	527	230.844
1481	cor	10.81	2	2	2	mask	265	1	control	487	230.844
1509	cor	10.81	2	2	2	mask	293	1	control	464	230.844
1510	cor	10.81	2	2	2	mask	294	1	control	529	230.844
1511	cor	10.81	2	2	2	mask	295	2	control	453	230.844
1512	cor	10.81	2	2	2	mask	296	3	mask_man	607	230.844
1513	cor	10.81	2	2	2	mask	297	3	control	523	230.844
1514	cor	10.81	2	2	2	mask	298	4	control	419	230.844
1515	cor	10.81	2	2	2	mask	299	1	control	404	230.844
1517	cor	10.81	2	2	2	mask	301	3	control	469	230.844
1519	cor	10.81	2	2	2	mask	303	1	control	390	230.844
1520	cor	10.81	2	2	2	mask	304	1	control	416	230.844
1522	cor	10.81	2	2	2	mask	306	6	control	427	230.844
1530	cor	10.81	2	2	2	mask	314	1	control	443	230.844
1539	cor	10.81	2	2	2	mask	323	2	mask_man	NA	230.844
1587	cor	10.81	2	2	2	mask	371	6	control	451	230.844
1588	cor	10.81	2	2	2	mask	372	6	control	393	230.844
1589	cor	10.81	2	2	2	mask	373	2	control	437	230.844
1591	cor	10.81	2	2	2	mask	375	4	mask_man	786	230.844
1592	cor	10.81	2	2	2	mask	376	6	control	426	230.844
1593	cor	10.81	2	2	2	mask	377	4	control	377	230.844
1594	cor	10.81	2	2	2	mask	378	5	control	380	230.844
1595	cor	10.81	2	2	2	mask	379	2	mask_man	454	230.844
1596	cor	10.81	2	2	2	mask	380	3	control	382	230.844
1598	cor	10.81	2	2	2	mask	382	5	control	360	230.844
1599	cor	10.81	2	2	2	mask	383	2	control	400	230.844
1606	cor	10.81	2	2	2	mask	390	4	control	410	230.844
1607	cor	10.81	2	2	2	mask	391	6	mask_man	566	230.844
1608	cor	10.81	2	2	2	mask	392	3	control	414	230.844
1610	cor	10.81	2	2	2	mask	394	2	control	399	230.844
1611	cor	10.81	2	2	2	mask	395	2	control	407	230.844
1612	cor	10.81	2	2	2	mask	396	5	control	337	230.844

1613	cor	10.81	2	2	2	mask	397	1	mask_man	472	230.844
1614	cor	10.81	2	2	2	mask	398	6	control	395	230.844
1629	cor	10.81	2	2	2	mask	413	6	control	405	230.844
1630	cor	10.81	2	2	2	mask	414	4	mask_man	401	230.844
1650	cor	10.81	2	2	2	mask	434	5	control	384	230.844
1651	cor	10.81	2	2	2	mask	435	2	control	420	230.844
1652	cor	10.81	2	2	2	mask	436	6	control	422	230.844
1654	cor	10.81	2	2	2	mask	438	4	control	402	230.844
1656	cor	10.81	2	2	2	mask	440	5	control	364	230.844
1657	cor	10.81	2	2	2	mask	441	3	control	402	230.844
1658	cor	10.81	2	2	2	mask	442	3	control	507	230.844
1660	cor	10.81	2	2	2	mask	444	5	control	393	230.844
1662	cor	10.81	2	2	2	mask	446	6	control	410	230.844
1663	cor	10.81	2	2	2	mask	447	2	control	420	230.844
1664	cor	10.81	2	2	2	mask	448	6	mask_man	441	230.844
1666	cor	10.81	2	2	2	mask	450	6	mask_man	399	230.844
1670	cor	10.81	2	2	2	mask	454	4	control	411	230.844
1673	cor	10.81	2	2	2	mask	457	5	control	447	230.844
1674	cor	10.81	2	2	2	mask	458	5	mask_man	362	230.844
1682	cor	10.81	2	2	2	mask	466	1	control	384	230.844
1691	cor	10.81	2	2	2	mask	475	5	mask_man	1882	230.844
1692	cor	10.81	2	2	2	mask	476	3	control	430	230.844
1693	cor	10.81	2	2	2	mask	477	2	control	372	230.844
1695	cor	10.81	2	2	2	mask	479	6	control	422	230.844
1696	cor	10.81	2	2	2	mask	480	3	control	429	230.844
1697	cor	10.81	2	2	2	mask	481	4	control	476	230.844
1699	cor	10.81	2	2	2	mask	483	6	control	386	230.844
1700	cor	10.81	2	2	2	mask	484	1	mask_man	391	230.844
1701	cor	10.81	2	2	2	mask	485	6	control	359	230.844
1706	cor	10.81	2	2	2	mask	490	4	control	432	230.844
1708	cor	10.81	2	2	2	mask	492	3	control	424	230.844
1715	cor	10.81	2	2	2	mask	499	2	control	398	230.844
1716	cor	10.81	2	2	2	mask	500	2	control	394	230.844
1727	cor	10.81	2	2	2	mask	511	4	control	385	230.844
1728	cor	10.81	2	2	2	mask	512	2	control	490	230.844
1730	cor	10.81	2	2	2	mask	514	4	control	385	230.844
1731	cor	10.81	2	2	2	mask	515	2	control	463	230.844
1733	cor	10.81	2	2	2	mask	517	5	control	391	230.844
1734	cor	10.81	2	2	2	mask	518	6	control	376	230.844
1736	cor	10.81	2	2	2	mask	520	1	control	479	230.844
1801	cor	10.81	2	2	2	mask	585	1	control	418	230.844
1802	cor	10.81	2	2	2	mask	586	4	mask_man	443	230.844
1810	cor	10.81	2	2	2	mask	594	5	control	1449	230.844
1811	cor	10.81	2	2	2	mask	595	4	control	363	230.844
1813	cor	10.81	2	2	2	mask	597	5	control	338	230.844
1814	cor	10.81	2	2	2	mask	598	1	control	384	230.844
1815	cor	10.81	2	2	2	mask	599	5	control	368	230.844
1816	der	6.24	5	1	2	mask	1	4	control	535	479.861
1822	der	6.24	5	1	2	mask	7	2	control	528	479.861
1827	der	6.24	5	1	2	mask	12	6	control	619	479.861

1830	der	6.24	5	1	2	mask	15	1	control	846	479.861
1833	der	6.24	5	1	2	mask	18	2	control	978	479.861
1835	der	6.24	5	1	2	mask	20	3	control	1490	479.861
1838	der	6.24	5	1	2	mask	23	4	control	657	479.861
1839	der	6.24	5	1	2	mask	24	1	control	761	479.861
1849	der	6.24	5	1	2	mask	34	2	control	629	479.861
1850	der	6.24	5	1	2	mask	35	6	control	455	479.861
1854	der	6.24	5	1	2	mask	39	4	control	567	479.861
1859	der	6.24	5	1	2	mask	44	5	control	512	479.861
1860	der	6.24	5	1	2	mask	45	3	mask_man	999	479.861
1861	der	6.24	5	1	2	mask	46	6	control	596	479.861
1862	der	6.24	5	1	2	mask	47	4	control	508	479.861
1863	der	6.24	5	1	2	mask	48	6	control	479	479.861
1864	der	6.24	5	1	2	mask	49	1	mask_man	865	479.861
1865	der	6.24	5	1	2	mask	50	2	control	684	479.861
1866	der	6.24	5	1	2	mask	51	6	control	1287	479.861
1867	der	6.24	5	1	2	mask	52	2	mask_man	1235	479.861
1869	der	6.24	5	1	2	mask	54	5	control	532	479.861
1870	der	6.24	5	1	2	mask	55	1	mask_man	NA	479.861
1872	der	6.24	5	1	2	mask	57	5	control	578	479.861
1873	der	6.24	5	1	2	mask	58	4	control	419	479.861
1876	der	6.24	5	1	2	mask	61	1	mask_man	715	479.861
1877	der	6.24	5	1	2	mask	62	2	control	561	479.861
1879	der	6.24	5	1	2	mask	64	5	control	521	479.861
1880	der	6.24	5	1	2	mask	65	1	control	919	479.861
1881	der	6.24	5	1	2	mask	66	4	mask_man	493	479.861
1884	der	6.24	5	1	2	mask	69	6	control	NA	479.861
1885	der	6.24	5	1	2	mask	70	3	control	1979	479.861
1892	der	6.24	5	1	2	mask	77	4	control	694	479.861
1893	der	6.24	5	1	2	mask	78	5	control	505	479.861
1894	der	6.24	5	1	2	mask	79	5	control	548	479.861
1896	der	6.24	5	1	2	mask	81	2	control	1446	479.861
1897	der	6.24	5	1	2	mask	82	5	mask_man	686	479.861
1898	der	6.24	5	1	2	mask	83	6	control	NA	479.861
1900	der	6.24	5	1	2	mask	85	5	control	798	479.861
1901	der	6.24	5	1	2	mask	86	3	control	1609	479.861
1902	der	6.24	5	1	2	mask	87	3	mask_man	651	479.861
1904	der	6.24	5	1	2	mask	89	1	control	951	479.861
1905	der	6.24	5	1	2	mask	90	6	control	495	479.861
1911	der	6.24	5	1	2	mask	96	1	control	2592	479.861
1912	der	6.24	5	1	2	mask	97	1	control	612	479.861
1914	der	6.24	5	1	2	mask	99	4	control	571	479.861
1937	der	6.24	5	1	2	mask	122	1	control	1034	479.861
1938	der	6.24	5	1	2	mask	123	5	control	564	479.861
1939	der	6.24	5	1	2	mask	124	5	mask_man	629	479.861
1940	der	6.24	5	1	2	mask	125	4	control	505	479.861
1944	der	6.24	5	1	2	mask	129	5	control	NA	479.861
1956	der	6.24	5	1	2	mask	141	1	control	NA	479.861
1966	der	6.24	5	1	2	mask	151	5	control	678	479.861
1967	der	6.24	5	1	2	mask	152	3	control	702	479.861

1980	der	6.24	5	1	2	mask	165	3	control	623	479.861
1981	der	6.24	5	1	2	mask	166	4	control	452	479.861
1994	der	6.24	5	1	2	mask	179	1	control	586	479.861
1995	der	6.24	5	1	2	mask	180	4	control	497	479.861
1996	der	6.24	5	1	2	mask	181	1	control	1428	479.861
1997	der	6.24	5	1	2	mask	182	5	mask_man	615	479.861
1998	der	6.24	5	1	2	mask	183	6	control	518	479.861
2019	der	6.24	5	1	2	mask	204	6	control	558	479.861
2021	der	6.24	5	1	2	mask	206	2	control	1419	479.861
2023	der	6.24	5	1	2	mask	208	3	control	NA	479.861
2024	der	6.24	5	1	2	mask	209	6	control	1245	479.861
2025	der	6.24	5	1	2	mask	210	4	mask_man	548	479.861
2026	der	6.24	5	1	2	mask	211	4	control	440	479.861
2027	der	6.24	5	1	2	mask	212	4	mask_man	463	479.861
2028	der	6.24	5	1	2	mask	213	5	control	468	479.861
2029	der	6.24	5	1	2	mask	214	3	control	1450	479.861
2030	der	6.24	5	1	2	mask	215	6	mask_man	543	479.861
2031	der	6.24	5	1	2	mask	216	6	control	474	479.861
2032	der	6.24	5	1	2	mask	217	3	control	542	479.861
2034	der	6.24	5	1	2	mask	219	3	control	462	479.861
2037	der	6.24	5	1	2	mask	222	2	control	669	479.861
2044	der	6.24	5	1	2	mask	229	6	control	625	479.861
2045	der	6.24	5	1	2	mask	230	2	control	650	479.861
2046	der	6.24	5	1	2	mask	231	2	mask_man	851	479.861
2047	der	6.24	5	1	2	mask	232	2	control	510	479.861
2049	der	6.24	5	1	2	mask	234	3	control	2216	479.861
2051	der	6.24	5	1	2	mask	236	2	control	575	479.861
2052	der	6.24	5	1	2	mask	237	3	control	543	479.861
2053	der	6.24	5	1	2	mask	238	4	control	425	479.861
2054	der	6.24	5	1	2	mask	239	6	mask_man	502	479.861
2055	der	6.24	5	1	2	mask	240	2	control	1330	479.861
2056	der	6.24	5	1	2	mask	241	2	mask_man	520	479.861
2057	der	6.24	5	1	2	mask	242	1	control	543	479.861
2059	der	6.24	5	1	2	mask	244	6	mask_man	510	479.861
2069	der	6.24	5	1	2	mask	254	5	control	779	479.861
2071	der	6.24	5	1	2	mask	256	4	control	766	479.861
2073	der	6.24	5	1	2	mask	258	1	control	1627	479.861
2074	der	6.24	5	1	2	mask	259	3	control	NA	479.861
2079	der	6.24	5	1	2	mask	264	1	control	NA	479.861
2085	der	6.24	5	1	2	mask	270	2	control	2552	479.861
2086	der	6.24	5	1	2	mask	271	2	control	521	479.861
2087	der	6.24	5	1	2	mask	272	3	mask_man	NA	479.861
2088	der	6.24	5	1	2	mask	273	6	control	486	479.861
2118	der	6.24	5	1	2	mask	303	5	control	540	479.861
2122	der	6.24	5	1	2	mask	307	2	control	NA	479.861
2133	der	6.24	5	1	2	mask	318	2	control	614	479.861
2134	der	6.24	5	1	2	mask	319	2	control	640	479.861
2135	der	6.24	7	1	2	object	1	4	control	548	479.861
2137	der	6.24	7	1	2	object	3	5	control	NA	479.861
2146	der	6.24	7	1	2	object	12	5	control	555	479.861

2147	der	6.24	7	1	2	object	13	3	control	809	479.861
2149	der	6.24	7	1	2	object	15	5	control	446	479.861
2151	der	6.24	7	1	2	object	17	3	control	686	479.861
2152	der	6.24	7	1	2	object	18	5	control	523	479.861
2157	der	6.24	7	1	2	object	23	6	control	516	479.861
2160	der	6.24	7	1	2	object	26	5	control	578	479.861
2161	der	6.24	7	1	2	object	27	5	control	464	479.861
2163	der	6.24	7	1	2	object	29	1	net	751	479.861
2164	der	6.24	7	1	2	object	30	3	control	812	479.861
2165	der	6.24	7	1	2	object	31	1	control	504	479.861
2167	der	6.24	7	1	2	object	33	1	control	2860	479.861
2169	der	6.24	7	1	2	object	35	6	control	603	479.861
2171	der	6.24	7	1	2	object	37	1	control	NA	479.861
2172	der	6.24	7	1	2	object	38	2	control	1818	479.861
2173	der	6.24	7	1	2	object	39	6	control	1026	479.861
2176	der	6.24	7	1	2	object	42	1	control	552	479.861
2177	der	6.24	7	1	2	object	43	3	net	764	479.861
2178	der	6.24	7	1	2	object	44	2	control	576	479.861
2179	der	6.24	7	1	2	object	45	5	control	508	479.861
2180	der	6.24	7	1	2	object	46	6	broom	2825	479.861
2181	der	6.24	7	1	2	object	47	3	control	618	479.861
2182	der	6.24	7	1	2	object	48	1	control	579	479.861
2185	der	6.24	7	1	2	object	51	5	control	987	479.861
2186	der	6.24	7	1	2	object	52	5	glove	1246	479.861
2187	der	6.24	7	1	2	object	53	5	control	612	479.861
2188	der	6.24	7	1	2	object	54	4	glove	536	479.861
2190	der	6.24	7	1	2	object	56	1	control	621	479.861
2192	der	6.24	7	1	2	object	58	4	control	458	479.861
2193	der	6.24	7	1	2	object	59	5	control	NA	479.861
2194	der	6.24	7	1	2	object	60	3	control	1052	479.861
2195	der	6.24	7	1	2	object	61	4	net	831	479.861
2198	der	6.24	7	1	2	object	64	3	control	2933	479.861
2199	der	6.24	7	1	2	object	65	2	control	1707	479.861
2200	der	6.24	7	1	2	object	66	3	glove	879	479.861
2202	der	6.24	7	1	2	object	68	4	control	557	479.861
2203	der	6.24	7	1	2	object	69	6	control	568	479.861
2204	der	6.24	7	1	2	object	70	2	control	596	479.861
2206	der	6.24	7	1	2	object	72	4	control	1636	479.861
2207	der	6.24	7	1	2	object	73	6	control	605	479.861
2208	der	6.24	7	1	2	object	74	1	control	503	479.861
2211	der	6.24	7	1	2	object	77	4	control	1304	479.861
2212	der	6.24	7	1	2	object	78	1	control	NA	479.861
2213	der	6.24	7	1	2	object	79	5	broom	700	479.861
2216	der	6.24	7	1	2	object	82	3	control	1071	479.861
2218	der	6.24	7	1	2	object	84	1	control	487	479.861
2220	der	6.24	7	1	2	object	86	1	control	NA	479.861
2221	der	6.24	7	1	2	object	87	6	control	633	479.861
2222	der	6.24	7	1	2	object	88	3	broom	1313	479.861
2223	der	6.24	7	1	2	object	89	1	control	2260	479.861
2225	der	6.24	7	1	2	object	91	6	glove	NA	479.861

2226	der	6.24	7	1	2	object	92	5	control	531	479.861
2235	der	6.24	7	1	2	object	101	5	control	731	479.861
2237	der	6.24	7	1	2	object	103	2	control	745	479.861
2242	der	6.24	7	1	2	object	108	1	glove	NA	479.861
2245	der	6.24	7	1	2	object	111	4	control	NA	479.861
2263	der	6.24	7	1	2	object	129	5	control	NA	479.861
2275	der	6.24	7	1	2	object	141	4	control	474	479.861
2277	der	6.24	7	1	2	object	143	6	control	519	479.861
2278	der	6.24	7	1	2	object	144	1	control	495	479.861
2282	der	6.24	7	1	2	object	148	4	control	526	479.861
2283	der	6.24	7	1	2	object	149	2	broom	782	479.861
2295	der	6.24	7	1	2	object	161	2	control	397	479.861
2296	der	6.24	7	1	2	object	162	3	control	629	479.861
2297	der	6.24	7	1	2	object	163	2	control	NA	479.861
2302	der	6.24	7	1	2	object	168	5	net	775	479.861
2303	der	6.24	7	1	2	object	169	2	control	NA	479.861
2306	der	6.24	7	1	2	object	172	2	control	663	479.861
2308	der	6.24	7	1	2	object	174	4	control	530	479.861
2309	der	6.24	7	1	2	object	175	2	control	540	479.861
2314	der	6.24	7	1	2	object	180	6	control	556	479.861
2315	der	6.24	7	1	2	object	181	2	net	778	479.861
2316	der	6.24	7	1	2	object	182	3	control	674	479.861
2317	der	6.24	7	1	2	object	183	2	control	476	479.861
2320	der	6.24	7	1	2	object	186	4	broom	649	479.861
2328	der	6.24	7	1	2	object	194	4	control	442	479.861
2329	der	6.24	7	1	2	object	195	4	control	948	479.861
2333	der	6.24	7	1	2	object	199	6	control	607	479.861
2334	der	6.24	7	1	2	object	200	3	control	1116	479.861
2336	der	6.24	7	1	2	object	202	4	control	575	479.861
2339	der	6.24	7	1	2	object	205	6	net	515	479.861
2343	der	6.24	7	1	2	object	209	2	control	544	479.861
2344	der	6.24	7	1	2	object	210	4	control	1211	479.861
2345	der	6.24	7	1	2	object	211	1	broom	NA	479.861
2348	der	6.24	7	1	2	object	214	3	control	1017	479.861
2349	der	6.24	7	1	2	object	215	6	control	NA	479.861
2350	der	6.24	7	1	2	object	216	5	control	566	479.861
2352	der	6.24	7	1	2	object	218	6	control	524	479.861
2353	der	6.24	7	1	2	object	219	6	control	415	479.861
2354	der	6.24	7	1	2	object	220	3	control	NA	479.861
2359	der	6.24	7	1	2	object	225	5	control	982	479.861
2364	der	6.24	7	1	2	object	230	3	control	511	479.861
2374	der	6.24	7	1	2	object	240	2	glove	NA	479.861
2375	der	6.24	7	1	2	object	241	4	control	NA	479.861
2388	der	6.24	7	1	2	object	254	6	control	560	479.861
2391	der	6.24	7	1	2	object	257	1	control	662	479.861
2392	der	6.24	7	1	2	object	258	2	control	525	479.861
2393	der	6.24	7	1	2	object	259	3	control	585	479.861
2727	elm	6.21	5	1	2	mask	1	2	control	762	290.101
2728	elm	6.21	5	1	2	mask	2	3	control	553	290.101
2729	elm	6.21	5	1	2	mask	3	5	control	614	290.101

2730	elm	6.21	5	1	2	mask	4	6	control	640	290.101
2732	elm	6.21	5	1	2	mask	6	4	control	757	290.101
2738	elm	6.21	5	1	2	mask	12	1	control	2374	290.101
2739	elm	6.21	5	1	2	mask	13	2	control	490	290.101
2740	elm	6.21	5	1	2	mask	14	3	control	632	290.101
2741	elm	6.21	5	1	2	mask	15	2	mask_man	2016	290.101
2742	elm	6.21	5	1	2	mask	16	5	control	608	290.101
2745	elm	6.21	5	1	2	mask	19	3	control	NA	290.101
2746	elm	6.21	5	1	2	mask	20	4	control	570	290.101
2747	elm	6.21	5	1	2	mask	21	2	control	470	290.101
2749	elm	6.21	5	1	2	mask	23	3	control	NA	290.101
2750	elm	6.21	5	1	2	mask	24	6	control	NA	290.101
2751	elm	6.21	5	1	2	mask	25	2	mask_man	1072	290.101
2754	elm	6.21	5	1	2	mask	28	1	control	NA	290.101
2755	elm	6.21	5	1	2	mask	29	6	mask_man	700	290.101
2756	elm	6.21	5	1	2	mask	30	6	control	NA	290.101
2768	elm	6.21	5	1	2	mask	42	6	control	516	290.101
2769	elm	6.21	5	1	2	mask	43	3	control	469	290.101
2770	elm	6.21	5	1	2	mask	44	4	mask_man	597	290.101
2779	elm	6.21	5	1	2	mask	53	2	control	NA	290.101
2792	elm	6.21	5	1	2	mask	66	4	control	707	290.101
2793	elm	6.21	5	1	2	mask	67	4	control	744	290.101
2795	elm	6.21	5	1	2	mask	69	4	control	NA	290.101
2796	elm	6.21	5	1	2	mask	70	2	control	582	290.101
2797	elm	6.21	5	1	2	mask	71	6	mask_man	775	290.101
2798	elm	6.21	5	1	2	mask	72	2	control	599	290.101
2799	elm	6.21	5	1	2	mask	73	3	control	475	290.101
2804	elm	6.21	5	1	2	mask	78	3	mask_man	663	290.101
2805	elm	6.21	5	1	2	mask	79	4	control	NA	290.101
2806	elm	6.21	5	1	2	mask	80	5	mask_man	626	290.101
2807	elm	6.21	5	1	2	mask	81	4	control	1124	290.101
2808	elm	6.21	5	1	2	mask	82	6	control	NA	290.101
2809	elm	6.21	5	1	2	mask	83	5	mask_man	455	290.101
2810	elm	6.21	5	1	2	mask	84	6	control	856	290.101
2811	elm	6.21	5	1	2	mask	85	6	control	NA	290.101
2813	elm	6.21	5	1	2	mask	87	1	control	NA	290.101
2814	elm	6.21	5	1	2	mask	88	3	mask_man	667	290.101
2815	elm	6.21	5	1	2	mask	89	1	control	721	290.101
2817	elm	6.21	5	1	2	mask	91	5	control	NA	290.101
2823	elm	6.21	5	1	2	mask	97	2	control	541	290.101
2824	elm	6.21	5	1	2	mask	98	2	control	501	290.101
2825	elm	6.21	5	1	2	mask	99	5	mask_man	531	290.101
2826	elm	6.21	5	1	2	mask	100	6	control	1190	290.101
2827	elm	6.21	5	1	2	mask	101	1	control	NA	290.101
2828	elm	6.21	5	1	2	mask	102	3	control	2792	290.101
2830	elm	6.21	5	1	2	mask	104	3	control	724	290.101
2831	elm	6.21	5	1	2	mask	105	1	mask_man	762	290.101
2832	elm	6.21	5	1	2	mask	106	3	control	2163	290.101
2833	elm	6.21	5	1	2	mask	107	1	control	NA	290.101
2845	elm	6.21	5	1	2	mask	119	1	control	NA	290.101

2846	elm	6.21	5	1	2	mask	120	2	control	641	290.101
2848	elm	6.21	5	1	2	mask	122	4	control	NA	290.101
2849	elm	6.21	5	1	2	mask	123	2	mask_man	595	290.101
2855	elm	6.21	5	1	2	mask	129	4	control	559	290.101
2856	elm	6.21	5	1	2	mask	130	3	control	NA	290.101
2857	elm	6.21	5	1	2	mask	131	4	control	NA	290.101
2858	elm	6.21	5	1	2	mask	132	4	mask_man	618	290.101
2859	elm	6.21	5	1	2	mask	133	6	control	599	290.101
2860	elm	6.21	5	1	2	mask	134	6	control	597	290.101
2862	elm	6.21	5	1	2	mask	136	4	control	1077	290.101
2863	elm	6.21	5	1	2	mask	137	1	control	771	290.101
2864	elm	6.21	5	1	2	mask	138	5	control	885	290.101
2866	elm	6.21	5	1	2	mask	140	1	control	736	290.101
2867	elm	6.21	5	1	2	mask	141	5	control	566	290.101
2868	elm	6.21	5	1	2	mask	142	5	control	424	290.101
2891	elm	6.21	5	1	2	mask	165	3	mask_man	656	290.101
2892	elm	6.21	5	1	2	mask	166	4	control	NA	290.101
2893	elm	6.21	5	1	2	mask	167	5	control	677	290.101
2895	elm	6.21	5	1	2	mask	169	3	control	675	290.101
2896	elm	6.21	5	1	2	mask	170	4	mask_man	722	290.101
2897	elm	6.21	5	1	2	mask	171	1	control	NA	290.101
2898	elm	6.21	5	1	2	mask	172	3	control	583	290.101
2900	elm	6.21	5	1	2	mask	174	2	control	650	290.101
2902	elm	6.21	5	1	2	mask	176	5	control	NA	290.101
2903	elm	6.21	5	1	2	mask	177	5	control	689	290.101
2904	elm	6.21	5	1	2	mask	178	1	control	NA	290.101
2906	elm	6.21	5	1	2	mask	180	5	control	1114	290.101
2908	elm	6.21	5	1	2	mask	182	5	control	743	290.101
2909	elm	6.21	5	1	2	mask	183	5	control	693	290.101
2910	elm	6.21	5	1	2	mask	184	2	control	616	290.101
2911	elm	6.21	5	1	2	mask	185	6	mask_man	869	290.101
2912	elm	6.21	5	1	2	mask	186	6	control	1013	290.101
2913	elm	6.21	5	1	2	mask	187	6	control	NA	290.101
2914	elm	6.21	5	1	2	mask	188	1	mask_man	708	290.101
2915	elm	6.21	5	1	2	mask	189	1	control	NA	290.101
2917	elm	6.21	5	1	2	mask	191	2	control	663	290.101
2918	elm	6.21	5	1	2	mask	192	2	control	577	290.101
2935	elm	6.21	5	1	2	mask	209	5	control	801	290.101
2936	elm	6.21	5	1	2	mask	210	1	mask_man	635	290.101
2937	elm	6.21	5	1	2	mask	211	6	control	NA	290.101
2938	elm	6.21	5	1	2	mask	212	3	control	2589	290.101
2939	elm	6.21	5	1	2	mask	213	4	control	836	290.101
2941	elm	6.21	5	1	2	mask	215	4	control	842	290.101
2943	elm	6.21	5	1	2	mask	217	6	control	581	290.101
2944	elm	6.21	5	1	2	mask	218	4	control	NA	290.101
2945	elm	6.21	5	1	2	mask	219	1	control	634	290.101
2946	elm	6.21	5	1	2	mask	220	5	control	621	290.101
2947	elm	6.21	7	1	2	object	1	4	control	1014	290.101
2948	elm	6.21	7	1	2	object	2	5	control	650	290.101
2949	elm	6.21	7	1	2	object	3	4	control	940	290.101

2951	elm	6.21	7	1	2	object	5	4	control	NA	290.101
2952	elm	6.21	7	1	2	object	6	1	control	1069	290.101
2953	elm	6.21	7	1	2	object	7	2	control	576	290.101
2954	elm	6.21	7	1	2	object	8	5	control	443	290.101
2958	elm	6.21	7	1	2	object	12	1	control	NA	290.101
2959	elm	6.21	7	1	2	object	13	4	control	791	290.101
2960	elm	6.21	7	1	2	object	14	2	control	504	290.101
2964	elm	6.21	7	1	2	object	18	6	control	NA	290.101
2965	elm	6.21	7	1	2	object	19	3	control	561	290.101
2966	elm	6.21	7	1	2	object	20	2	control	550	290.101
2967	elm	6.21	7	1	2	object	21	6	net	NA	290.101
2968	elm	6.21	7	1	2	object	22	5	control	871	290.101
2969	elm	6.21	7	1	2	object	23	6	glove	1560	290.101
2970	elm	6.21	7	1	2	object	24	2	control	575	290.101
2971	elm	6.21	7	1	2	object	25	3	control	725	290.101
2972	elm	6.21	7	1	2	object	26	1	broom	1060	290.101
2973	elm	6.21	7	1	2	object	27	3	control	570	290.101
2974	elm	6.21	7	1	2	object	28	6	control	NA	290.101
2975	elm	6.21	7	1	2	object	29	6	control	463	290.101
2977	elm	6.21	7	1	2	object	31	5	control	NA	290.101
2979	elm	6.21	7	1	2	object	33	1	control	NA	290.101
2980	elm	6.21	7	1	2	object	34	4	control	NA	290.101
2981	elm	6.21	7	1	2	object	35	5	control	499	290.101
2982	elm	6.21	7	1	2	object	36	5	net	835	290.101
2983	elm	6.21	7	1	2	object	37	6	control	597	290.101
2984	elm	6.21	7	1	2	object	38	1	control	622	290.101
2985	elm	6.21	7	1	2	object	39	3	control	539	290.101
2986	elm	6.21	7	1	2	object	40	1	net	797	290.101
2987	elm	6.21	7	1	2	object	41	5	control	NA	290.101
2989	elm	6.21	7	1	2	object	43	6	control	910	290.101
2990	elm	6.21	7	1	2	object	44	2	control	NA	290.101
2992	elm	6.21	7	1	2	object	46	4	control	NA	290.101
2994	elm	6.21	7	1	2	object	48	1	control	600	290.101
2995	elm	6.21	7	1	2	object	49	2	control	558	290.101
2996	elm	6.21	7	1	2	object	50	6	broom	833	290.101
2998	elm	6.21	7	1	2	object	52	1	control	NA	290.101
2999	elm	6.21	7	1	2	object	53	6	control	714	290.101
3001	elm	6.21	7	1	2	object	55	5	control	574	290.101
3002	elm	6.21	7	1	2	object	56	1	control	867	290.101
3004	elm	6.21	7	1	2	object	58	4	control	NA	290.101
3005	elm	6.21	7	1	2	object	59	4	glove	699	290.101
3006	elm	6.21	7	1	2	object	60	6	control	587	290.101
3007	elm	6.21	7	1	2	object	61	2	net	675	290.101
3008	elm	6.21	7	1	2	object	62	3	control	554	290.101
3009	elm	6.21	7	1	2	object	63	6	control	1038	290.101
3010	elm	6.21	7	1	2	object	64	5	broom	706	290.101
3011	elm	6.21	7	1	2	object	65	4	control	NA	290.101
3012	elm	6.21	7	1	2	object	66	3	control	NA	290.101
3014	elm	6.21	7	1	2	object	68	2	control	NA	290.101
3016	elm	6.21	7	1	2	object	70	5	control	NA	290.101

3017	elm	6.21	7	1	2	object	71	4	control	NA	290.101
3018	elm	6.21	7	1	2	object	72	4	net	793	290.101
3019	elm	6.21	7	1	2	object	73	3	control	593	290.101
3020	elm	6.21	7	1	2	object	74	4	control	678	290.101
3021	elm	6.21	7	1	2	object	75	5	control	521	290.101
3023	elm	6.21	7	1	2	object	77	5	control	496	290.101
3024	elm	6.21	7	1	2	object	78	4	control	NA	290.101
3025	elm	6.21	7	1	2	object	79	1	control	883	290.101
3027	elm	6.21	7	1	2	object	81	6	control	NA	290.101
3028	elm	6.21	7	1	2	object	82	1	glove	794	290.101
3029	elm	6.21	7	1	2	object	83	1	control	703	290.101
3031	elm	6.21	7	1	2	object	85	2	control	722	290.101
3033	elm	6.21	7	1	2	object	87	6	control	793	290.101
3034	elm	6.21	7	1	2	object	88	4	control	822	290.101
3035	elm	6.21	7	1	2	object	89	2	glove	619	290.101
3038	elm	6.21	7	1	2	object	92	3	control	NA	290.101
3039	elm	6.21	7	1	2	object	93	2	control	683	290.101
3040	elm	6.21	7	1	2	object	94	4	control	NA	290.101
3042	elm	6.21	7	1	2	object	96	3	control	666	290.101
3043	elm	6.21	7	1	2	object	97	2	control	591	290.101
3044	elm	6.21	7	1	2	object	98	4	broom	719	290.101
3045	elm	6.21	7	1	2	object	99	1	control	NA	290.101
3046	elm	6.21	7	1	2	object	100	3	control	694	290.101
3048	elm	6.21	7	1	2	object	102	3	control	NA	290.101
3050	elm	6.21	7	1	2	object	104	5	control	NA	290.101
3051	elm	6.21	7	1	2	object	105	3	glove	849	290.101
3056	elm	6.21	7	1	2	object	110	1	control	NA	290.101
3058	elm	6.21	7	1	2	object	112	3	control	NA	290.101
3059	elm	6.21	7	1	2	object	113	4	control	650	290.101
3060	elm	6.21	7	1	2	object	114	2	control	647	290.101
3061	elm	6.21	7	1	2	object	115	2	broom	654	290.101
3062	elm	6.21	7	1	2	object	116	6	control	753	290.101
3063	elm	6.21	7	1	2	object	117	5	control	760	290.101
3064	elm	6.21	7	1	2	object	118	1	control	NA	290.101
3065	elm	6.21	7	1	2	object	119	3	broom	583	290.101
3066	elm	6.21	7	1	2	object	120	1	control	NA	290.101
3067	elm	6.21	7	1	2	object	121	5	glove	644	290.101
3068	elm	6.21	7	1	2	object	122	4	control	510	290.101
3069	elm	6.21	7	1	2	object	123	3	net	1213	290.101
3070	elm	6.21	7	1	2	object	124	6	control	1286	290.101
3075	elm	6.21	7	1	2	object	129	5	control	816	290.101
3077	elm	6.21	7	1	2	object	131	2	control	739	290.101
3079	elm	6.21	7	1	2	object	133	4	control	NA	290.101
3081	elm	6.21	7	1	2	object	135	6	control	746	290.101
3082	elm	6.21	7	1	2	object	136	6	control	2377	290.101
3083	elm	6.21	7	1	2	object	137	1	control	791	290.101
3084	elm	6.21	7	1	2	object	138	3	control	567	290.101
4980	gro	7.68	7	1	2	mask	1	5	control	422	169.929
4981	gro	7.68	7	1	2	mask	2	3	control	461	169.929
4984	gro	7.68	7	1	2	mask	5	3	control	521	169.929

4985	gro	7.68	7	1	2	mask	6	6	control	856	169.929
4989	gro	7.68	7	1	2	mask	10	6	control	939	169.929
4990	gro	7.68	7	1	2	mask	11	6	control	526	169.929
4991	gro	7.68	7	1	2	mask	12	1	mask_man	NA	169.929
4992	gro	7.68	7	1	2	mask	13	6	control	1270	169.929
4993	gro	7.68	7	1	2	mask	14	6	control	NA	169.929
4994	gro	7.68	7	1	2	mask	15	5	mask_man	978	169.929
4995	gro	7.68	7	1	2	mask	16	1	control	NA	169.929
4997	gro	7.68	7	1	2	mask	18	2	control	1865	169.929
4998	gro	7.68	7	1	2	mask	19	1	control	2138	169.929
4999	gro	7.68	7	1	2	mask	20	5	mask_man	812	169.929
5000	gro	7.68	7	1	2	mask	21	6	control	1687	169.929
5002	gro	7.68	7	1	2	mask	23	4	control	591	169.929
5003	gro	7.68	7	1	2	mask	24	6	control	630	169.929
5005	gro	7.68	7	1	2	mask	26	4	control	733	169.929
5006	gro	7.68	7	1	2	mask	27	2	control	683	169.929
5007	gro	7.68	7	1	2	mask	28	6	control	NA	169.929
5008	gro	7.68	7	1	2	mask	29	4	mask_man	861	169.929
5009	gro	7.68	7	1	2	mask	30	1	control	NA	169.929
5010	gro	7.68	7	1	2	mask	31	2	control	1105	169.929
5011	gro	7.68	7	1	2	mask	32	1	mask_man	860	169.929
5012	gro	7.68	7	1	2	mask	33	2	control	NA	169.929
5013	gro	7.68	7	1	2	mask	34	3	mask_man	844	169.929
5014	gro	7.68	7	1	2	mask	35	1	control	NA	169.929
5015	gro	7.68	7	1	2	mask	36	2	control	NA	169.929
5017	gro	7.68	7	1	2	mask	38	5	control	NA	169.929
5018	gro	7.68	7	1	2	mask	39	4	control	NA	169.929
5019	gro	7.68	7	1	2	mask	40	5	control	NA	169.929
5021	gro	7.68	7	1	2	mask	42	6	control	NA	169.929
5022	gro	7.68	7	1	2	mask	43	6	control	NA	169.929
5024	gro	7.68	7	1	2	mask	45	3	control	NA	169.929
5025	gro	7.68	7	1	2	mask	46	6	control	2176	169.929
5026	gro	7.68	7	1	2	mask	47	5	control	NA	169.929
5028	gro	7.68	7	1	2	mask	49	4	control	1078	169.929
5029	gro	7.68	7	1	2	mask	50	4	control	NA	169.929
5030	gro	7.68	7	1	2	mask	51	3	mask_man	1023	169.929
5031	gro	7.68	7	1	2	mask	52	1	control	NA	169.929
5032	gro	7.68	7	1	2	mask	53	6	mask_man	731	169.929
5033	gro	7.68	7	1	2	mask	54	2	control	1321	169.929
5035	gro	7.68	7	1	2	mask	56	6	control	NA	169.929
5036	gro	7.68	7	1	2	mask	57	2	mask_man	805	169.929
5037	gro	7.68	7	1	2	mask	58	2	control	1106	169.929
5038	gro	7.68	7	1	2	mask	59	3	control	NA	169.929
5039	gro	7.68	7	1	2	mask	60	6	mask_man	887	169.929
5041	gro	7.68	7	1	2	mask	62	2	control	855	169.929
5042	gro	7.68	7	1	2	mask	63	1	control	NA	169.929
5044	gro	7.68	7	1	2	mask	65	3	control	NA	169.929
5045	gro	7.68	7	1	2	mask	66	5	control	609	169.929
5046	gro	7.68	7	1	2	mask	67	4	control	NA	169.929
5047	gro	7.68	7	1	2	mask	68	1	mask_man	846	169.929

5050	gro	7.68	7	1	2	mask	71	1	control	2662	169.929
5051	gro	7.68	7	1	2	mask	72	5	control	616	169.929
5053	gro	7.68	7	1	2	mask	74	4	control	2330	169.929
5054	gro	7.68	7	1	2	mask	75	4	control	802	169.929
5055	gro	7.68	7	1	2	mask	76	2	mask_man	859	169.929
5056	gro	7.68	7	1	2	mask	77	1	control	1321	169.929
5057	gro	7.68	7	1	2	mask	78	5	control	2897	169.929
5059	gro	7.68	7	1	2	mask	80	3	control	696	169.929
5061	gro	7.68	7	1	2	mask	82	2	control	2706	169.929
5062	gro	7.68	7	1	2	mask	83	3	control	614	169.929
5063	gro	7.68	7	1	2	mask	84	3	control	1538	169.929
5065	gro	7.68	7	1	2	mask	86	6	control	497	169.929
5067	gro	7.68	7	1	2	mask	88	4	control	826	169.929
5068	gro	7.68	7	1	2	mask	89	5	control	723	169.929
5069	gro	7.68	7	1	2	mask	90	4	mask_man	863	169.929
5070	gro	7.68	7	1	2	mask	91	5	control	719	169.929
5071	gro	7.68	7	1	2	mask	92	5	control	531	169.929
5072	gro	7.68	7	1	2	mask	93	2	control	519	169.929
5073	gro	7.68	7	1	2	mask	94	6	mask_man	1034	169.929
5075	gro	7.68	7	1	2	mask	96	3	control	989	169.929
5078	gro	7.68	7	1	2	mask	99	4	control	889	169.929
5089	gro	7.68	7	1	2	mask	110	4	control	NA	169.929
5091	gro	7.68	7	1	2	mask	112	5	control	1189	169.929
5093	gro	7.68	7	1	2	mask	114	5	control	NA	169.929
5094	gro	7.68	7	1	2	mask	115	3	control	592	169.929
5095	gro	7.68	7	1	2	mask	116	4	control	NA	169.929
5096	gro	7.68	7	1	2	mask	117	3	mask_man	973	169.929
5098	gro	7.68	7	1	2	mask	119	3	control	788	169.929
5100	gro	7.68	7	1	2	mask	121	1	control	NA	169.929
5101	gro	7.68	7	1	2	mask	122	5	mask_man	684	169.929
5104	gro	7.68	7	1	2	mask	125	1	control	727	169.929
5105	gro	7.68	7	1	2	mask	126	4	mask_man	865	169.929
5108	gro	7.68	7	1	2	mask	129	3	control	688	169.929
5109	gro	7.68	7	1	2	mask	130	3	control	NA	169.929
5113	gro	7.68	7	1	2	mask	134	1	control	2044	169.929
5115	gro	7.68	7	1	2	mask	136	3	control	674	169.929
5116	gro	7.68	7	1	2	mask	137	6	control	576	169.929
5117	gro	7.68	7	1	2	mask	138	2	control	719	169.929
5118	gro	7.68	7	1	2	mask	139	2	mask_man	852	169.929
5148	gro	7.68	7	1	2	mask	169	1	control	NA	169.929
5149	gro	7.68	7	1	2	mask	170	2	control	1560	169.929
5150	gro	7.68	7	1	2	mask	171	5	control	600	169.929
5152	gro	7.68	7	1	2	mask	173	5	control	555	169.929
5153	gro	7.68	7	1	2	mask	174	6	control	438	169.929
5155	gro	7.68	7	1	2	mask	176	6	control	427	169.929
5156	gro	7.68	7	1	2	mask	177	3	control	477	169.929
5157	gro	7.68	7	1	2	mask	178	2	control	462	169.929
5158	gro	7.68	7	1	2	object	1	4	control	553	169.929
5159	gro	7.68	7	1	2	object	2	5	control	NA	169.929
5160	gro	7.68	7	1	2	object	3	4	control	631	169.929

5161	gro	7.68	7	1	2	object	4	4	control	NA	169.929
5163	gro	7.68	7	1	2	object	6	1	control	NA	169.929
5164	gro	7.68	7	1	2	object	7	5	control	NA	169.929
5165	gro	7.68	7	1	2	object	8	2	control	NA	169.929
5166	gro	7.68	7	1	2	object	9	5	control	784	169.929
5167	gro	7.68	7	1	2	object	10	2	broom	1486	169.929
5168	gro	7.68	7	1	2	object	11	6	control	587	169.929
5169	gro	7.68	7	1	2	object	12	5	control	1329	169.929
5171	gro	7.68	7	1	2	object	14	4	control	957	169.929
5172	gro	7.68	7	1	2	object	15	6	control	799	169.929
5173	gro	7.68	7	1	2	object	16	6	broom	865	169.929
5177	gro	7.68	7	1	2	object	20	5	control	598	169.929
5178	gro	7.68	7	1	2	object	21	3	control	689	169.929
5179	gro	7.68	7	1	2	object	22	1	broom	1008	169.929
5180	gro	7.68	7	1	2	object	23	3	control	655	169.929
5181	gro	7.68	7	1	2	object	24	1	control	1146	169.929
5182	gro	7.68	7	1	2	object	25	5	control	632	169.929
5184	gro	7.68	7	1	2	object	27	3	control	576	169.929
5185	gro	7.68	7	1	2	object	28	1	control	618	169.929
5186	gro	7.68	7	1	2	object	29	1	control	592	169.929
5187	gro	7.68	7	1	2	object	30	4	net	2896	169.929
5188	gro	7.68	7	1	2	object	31	4	control	645	169.929
5189	gro	7.68	7	1	2	object	32	6	glove	1201	169.929
5190	gro	7.68	7	1	2	object	33	2	control	NA	169.929
5191	gro	7.68	7	1	2	object	34	6	control	1116	169.929
5192	gro	7.68	7	1	2	object	35	3	control	719	169.929
5194	gro	7.68	7	1	2	object	37	1	net	1515	169.929
5195	gro	7.68	7	1	2	object	38	5	control	1688	169.929
5196	gro	7.68	7	1	2	object	39	1	control	NA	169.929
5198	gro	7.68	7	1	2	object	41	4	control	569	169.929
5200	gro	7.68	7	1	2	object	43	1	control	720	169.929
5201	gro	7.68	7	1	2	object	44	1	control	783	169.929
5203	gro	7.68	7	1	2	object	46	2	control	577	169.929
5204	gro	7.68	7	1	2	object	47	6	control	602	169.929
5205	gro	7.68	7	1	2	object	48	5	broom	955	169.929
5206	gro	7.68	7	1	2	object	49	5	control	582	169.929
5211	gro	7.68	7	1	2	object	54	4	control	574	169.929
5212	gro	7.68	7	1	2	object	55	6	control	584	169.929
5214	gro	7.68	7	1	2	object	57	5	control	558	169.929
5215	gro	7.68	7	1	2	object	58	4	control	555	169.929
5216	gro	7.68	7	1	2	object	59	4	broom	868	169.929
5218	gro	7.68	7	1	2	object	61	5	control	NA	169.929
5231	gro	7.68	7	1	2	object	74	4	control	599	169.929
5233	gro	7.68	7	1	2	object	76	2	control	572	169.929
5234	gro	7.68	7	1	2	object	77	3	net	1140	169.929
5235	gro	7.68	7	1	2	object	78	3	control	840	169.929
5236	gro	7.68	7	1	2	object	79	2	control	599	169.929
5237	gro	7.68	7	1	2	object	80	6	control	514	169.929
5238	gro	7.68	7	1	2	object	81	3	broom	971	169.929
5240	gro	7.68	7	1	2	object	83	2	control	544	169.929

5241	gro	7.68	7	1	2	object	84	1	control	609	169.929
5242	gro	7.68	7	1	2	object	85	2	glove	710	169.929
5243	gro	7.68	7	1	2	object	86	1	control	961	169.929
5260	gro	7.68	7	1	2	object	103	6	control	668	169.929
5261	gro	7.68	7	1	2	object	104	4	control	557	169.929
5262	gro	7.68	7	1	2	object	105	6	control	505	169.929
5264	gro	7.68	7	1	2	object	107	6	control	486	169.929
5265	gro	7.68	7	1	2	object	108	3	control	486	169.929
5267	gro	7.68	7	1	2	object	110	4	control	559	169.929
5269	gro	7.68	7	1	2	object	112	2	control	652	169.929
5270	gro	7.68	7	1	2	object	113	4	control	503	169.929
5278	gro	7.68	7	1	2	object	121	1	control	646	169.929
5280	gro	7.68	7	1	2	object	123	3	control	555	169.929
5281	gro	7.68	7	1	2	object	124	2	net	932	169.929
5282	gro	7.68	7	1	2	object	125	3	control	589	169.929
5287	gro	7.68	7	1	2	object	130	3	control	672	169.929
5288	gro	7.68	7	1	2	object	131	6	control	938	169.929
5290	gro	7.68	7	1	2	object	133	2	control	560	169.929
5291	gro	7.68	7	1	2	object	134	1	control	688	169.929
5292	gro	7.68	7	1	2	object	135	3	glove	1025	169.929
5293	gro	7.68	7	1	2	object	136	2	control	531	169.929
5294	gro	7.68	7	1	2	object	137	5	glove	1019	169.929
5295	gro	7.68	7	1	2	object	138	4	control	519	169.929
5296	gro	7.68	7	1	2	object	139	3	control	549	169.929
5297	gro	7.68	7	1	2	object	140	4	control	509	169.929
5298	gro	7.68	7	1	2	object	141	5	net	855	169.929
5299	gro	7.68	7	1	2	object	142	3	control	548	169.929
5300	gro	7.68	7	1	2	object	143	4	glove	770	169.929
5301	gro	7.68	7	1	2	object	144	5	control	489	169.929
5303	gro	7.68	7	1	2	object	146	4	control	535	169.929
5305	gro	7.68	7	1	2	object	148	2	control	518	169.929
5306	gro	7.68	7	1	2	object	149	2	control	870	169.929
5307	gro	7.68	7	1	2	object	150	6	control	498	169.929
5309	gro	7.68	7	1	2	object	152	2	control	563	169.929
5310	gro	7.68	7	1	2	object	153	6	control	513	169.929
5311	gro	7.68	7	1	2	object	154	1	control	527	169.929
5312	gro	7.68	7	1	2	object	155	6	net	910	169.929
5313	gro	7.68	7	1	2	object	156	3	control	543	169.929
5314	gro	7.68	7	1	2	object	157	1	control	601	169.929
5315	gro	7.68	7	1	2	object	158	5	control	2227	169.929
5317	gro	7.68	7	1	2	object	160	5	control	2625	169.929
5318	gro	7.68	7	1	2	object	161	1	glove	825	169.929
5319	gro	7.68	7	1	2	object	162	1	control	568	169.929
5320	gro	7.68	7	1	2	object	163	3	control	666	169.929
5322	gro	7.68	7	1	2	object	165	1	control	626	169.929
5323	gro	7.68	7	1	2	object	166	4	control	876	169.929
5324	gro	7.68	7	1	2	object	167	3	control	625	169.929
5325	han	7.15	7	1	2	mask	1	3	control	582	256.872
5326	han	7.15	7	1	2	mask	2	1	control	528	256.872
5327	han	7.15	7	1	2	mask	3	6	control	459	256.872

5333	han	7.15	7	1	2	mask	9	2	control	368	256.872
5358	han	7.15	7	1	2	mask	34	4	control	597	256.872
5359	han	7.15	7	1	2	mask	35	1	control	487	256.872
5368	han	7.15	7	1	2	mask	44	6	control	422	256.872
5370	han	7.15	7	1	2	mask	46	6	control	454	256.872
5372	han	7.15	7	1	2	mask	48	2	control	455	256.872
5373	han	7.15	7	1	2	mask	49	4	control	474	256.872
5374	han	7.15	7	1	2	mask	50	5	mask_woma	337	256.872
5375	han	7.15	7	1	2	mask	51	4	control	538	256.872
5382	han	7.15	7	1	2	mask	58	2	mask_woma	938	256.872
5383	han	7.15	7	1	2	mask	59	4	control	540	256.872
5440	han	7.15	7	1	2	mask	116	2	control	450	256.872
5441	han	7.15	7	1	2	mask	117	5	control	417	256.872
5449	han	7.15	7	1	2	mask	125	6	mask_woma	348	256.872
5458	han	7.15	7	1	2	mask	134	5	control	429	256.872
5462	han	7.15	7	1	2	mask	138	5	control	471	256.872
5463	han	7.15	7	1	2	mask	139	5	control	470	256.872
5470	han	7.15	7	1	2	mask	146	4	control	727	256.872
5472	han	7.15	7	1	2	mask	148	6	control	449	256.872
5473	han	7.15	7	1	2	mask	149	5	control	351	256.872
5474	han	7.15	7	1	2	mask	150	3	control	504	256.872
5475	han	7.15	7	1	2	mask	151	3	mask_woma	433	256.872
5476	han	7.15	7	1	2	mask	152	4	control	451	256.872
5477	han	7.15	7	1	2	mask	153	4	mask_woma	390	256.872
5478	han	7.15	7	1	2	mask	154	1	control	397	256.872
5480	han	7.15	7	1	2	mask	156	2	control	567	256.872
5481	han	7.15	7	1	2	mask	157	1	control	484	256.872
5482	han	7.15	7	1	2	mask	158	6	control	349	256.872
5484	han	7.15	7	1	2	mask	160	4	mask_woma	455	256.872
5500	han	7.15	7	1	2	mask	176	3	control	619	256.872
5536	han	7.15	7	1	2	mask	212	3	control	1046	256.872
5537	han	7.15	7	1	2	mask	213	3	control	404	256.872
5540	han	7.15	7	1	2	mask	216	3	control	551	256.872
5543	han	7.15	7	1	2	mask	219	4	control	448	256.872
5546	han	7.15	7	1	2	mask	222	1	control	571	256.872
5547	han	7.15	7	1	2	mask	223	1	control	525	256.872
5552	han	7.15	7	1	2	mask	228	5	mask_woma	422	256.872
5553	han	7.15	7	1	2	mask	229	1	control	1055	256.872
5555	han	7.15	7	1	2	mask	231	2	control	399	256.872
5560	han	7.15	7	1	2	mask	236	2	control	601	256.872
5561	han	7.15	7	1	2	mask	237	6	mask_woma	365	256.872
5562	han	7.15	7	1	2	mask	238	5	control	393	256.872
5625	han	7.15	7	1	2	mask	301	4	control	364	256.872
5626	han	7.15	7	1	2	mask	302	1	mask_woma	590	256.872
5632	han	7.15	7	1	2	mask	308	6	control	748	256.872
5633	han	7.15	7	1	2	mask	309	4	control	521	256.872
5634	han	7.15	7	1	2	mask	310	1	control	552	256.872
5636	han	7.15	7	1	2	mask	312	6	control	409	256.872
5639	han	7.15	7	1	2	mask	315	6	control	474	256.872
5640	han	7.15	7	1	2	mask	316	5	control	387	256.872

5657	han	7.15	7	1	2	mask	333	2	control	421	256.872
5658	han	7.15	7	1	2	mask	334	6	control	469	256.872
5677	han	7.15	7	1	2	mask	353	3	control	665	256.872
5679	han	7.15	7	1	2	mask	355	3	control	421	256.872
5681	han	7.15	7	1	2	mask	357	4	control	447	256.872
5692	han	7.15	7	1	2	mask	1	5	control	510	256.872
5693	han	7.15	7	1	2	mask	2	2	control	395	256.872
5696	han	7.15	7	1	2	mask	5	4	control	NA	256.872
5716	han	7.15	7	1	2	mask	25	2	control	859	256.872
5729	han	7.15	7	1	2	mask	38	1	control	687	256.872
5730	han	7.15	7	1	2	mask	39	2	control	521	256.872
5732	han	7.15	7	1	2	mask	41	1	control	535	256.872
5733	han	7.15	7	1	2	mask	42	3	mask_woma	455	256.872
5738	han	7.15	7	1	2	mask	47	6	control	448	256.872
5739	han	7.15	7	1	2	mask	48	6	control	NA	256.872
5744	han	7.15	7	1	2	mask	53	3	control	950	256.872
5745	han	7.15	7	1	2	mask	54	2	mask_woma	663	256.872
5746	han	7.15	7	1	2	mask	55	6	control	434	256.872
5747	han	7.15	7	1	2	mask	56	6	mask_woma	1028	256.872
5748	han	7.15	7	1	2	mask	57	5	control	337	256.872
5750	han	7.15	7	1	2	mask	59	3	control	499	256.872
5751	han	7.15	7	1	2	mask	60	1	control	459	256.872
5754	han	7.15	7	1	2	mask	63	6	control	407	256.872
5761	han	7.15	7	1	2	mask	70	3	control	645	256.872
5762	han	7.15	7	1	2	mask	71	5	control	624	256.872
5764	han	7.15	7	1	2	mask	73	3	control	504	256.872
5766	han	7.15	7	1	2	mask	75	5	mask_woma	336	256.872
5767	han	7.15	7	1	2	mask	76	1	control	447	256.872
5770	han	7.15	7	1	2	mask	79	6	control	787	256.872
5773	han	7.15	7	1	2	mask	82	4	control	386	256.872
5774	han	7.15	7	1	2	mask	83	4	mask_woma	331	256.872
5775	han	7.15	7	1	2	mask	84	6	control	558	256.872
5776	han	7.15	7	1	2	mask	85	1	control	431	256.872
5779	han	7.15	7	1	2	mask	88	4	control	457	256.872
5780	han	7.15	7	1	2	mask	89	1	mask_woma	370	256.872
5781	han	7.15	7	1	2	mask	90	2	control	537	256.872
5783	han	7.15	7	1	2	mask	92	3	control	NA	256.872
5784	han	7.15	7	1	2	mask	93	2	control	442	256.872
5785	han	7.15	7	1	2	mask	94	3	mask_woma	502	256.872
5786	han	7.15	7	1	2	mask	95	1	control	352	256.872
5787	han	7.15	7	1	2	mask	96	4	mask_woma	380	256.872
5788	han	7.15	7	1	2	mask	97	1	control	352	256.872
5789	han	7.15	7	1	2	mask	98	2	control	346	256.872
5791	han	7.15	7	1	2	mask	100	1	control	726	256.872
5792	han	7.15	7	1	2	mask	101	3	mask_woma	1065	256.872
5793	han	7.15	7	1	2	mask	102	6	control	845	256.872
5794	han	7.15	7	1	2	mask	103	3	control	536	256.872
5795	han	7.15	7	1	2	mask	104	6	mask_woma	651	256.872
5796	han	7.15	7	1	2	mask	105	4	control	420	256.872
5797	han	7.15	7	1	2	mask	106	2	control	597	256.872

5798	han	7.15	7	1	2	mask	107	5	mask_woma	426	256.872
5799	han	7.15	7	1	2	mask	108	4	control	686	256.872
5800	han	7.15	7	1	2	mask	109	2	control	496	256.872
5802	han	7.15	7	1	2	mask	111	1	control	501	256.872
5803	han	7.15	7	1	2	mask	112	4	control	529	256.872
5804	han	7.15	7	1	2	mask	113	6	control	318	256.872
5805	han	7.15	7	1	2	mask	114	5	mask_woma	756	256.872
5808	han	7.15	7	1	2	mask	117	4	control	437	256.872
5811	han	7.15	7	1	2	mask	120	4	control	651	256.872
5812	han	7.15	7	1	2	mask	121	5	control	623	256.872
5816	han	7.15	7	1	2	mask	125	3	control	480	256.872
5818	han	7.15	7	1	2	mask	127	2	control	NA	256.872
5819	han	7.15	7	1	2	mask	128	4	control	486	256.872
5856	han	7.15	7	1	2	mask	165	2	control	699	256.872
5857	han	7.15	7	1	2	mask	166	5	control	602	256.872
5863	han	7.15	7	1	2	mask	172	3	control	2231	256.872
5865	han	7.15	7	1	2	mask	174	5	control	389	256.872
5866	han	7.15	7	1	2	mask	175	5	control	342	256.872
5867	han	7.15	7	1	2	mask	176	1	mask_woma	706	256.872
5868	han	7.15	7	1	2	mask	177	1	control	840	256.872
5870	han	7.15	7	1	2	mask	179	1	mask_woma	2765	256.872
5871	han	7.15	7	1	2	mask	180	6	control	355	256.872
5872	han	7.15	7	1	2	mask	181	2	control	356	256.872
5874	han	7.15	7	1	2	mask	183	1	control	369	256.872
5898	han	7.15	7	1	2	mask	207	4	control	365	256.872
5899	han	7.15	7	1	2	mask	208	4	control	313	256.872
5900	han	7.15	7	1	2	mask	209	2	mask_woma	401	256.872
5907	han	7.15	7	1	2	mask	216	4	control	470	256.872
5908	han	7.15	7	1	2	mask	217	5	control	1032	256.872
5909	han	7.15	7	1	2	mask	218	3	control	719	256.872
5924	han	7.15	7	1	2	mask	233	6	control	385	256.872
5925	han	7.15	7	1	2	mask	234	5	control	430	256.872
5926	han	7.15	7	1	2	mask	235	1	control	348	256.872
5939	han	7.15	7	1	2	mask	248	6	control	NA	256.872
5940	han	7.15	7	1	2	mask	249	1	control	334	256.872
5943	han	7.15	7	1	2	mask	252	5	control	570	256.872
5945	han	7.15	7	1	2	mask	254	2	control	630	256.872
5946	han	7.15	7	1	2	mask	255	3	control	402	256.872
5947	han	7.15	7	1	2	mask	256	2	control	347	256.872
5951	han	7.15	7	1	2	mask	260	5	control	586	256.872
5952	han	7.15	7	1	2	mask	261	4	control	323	256.872
5955	han	7.15	7	1	2	mask	264	2	mask_woma	351	256.872
5972	han	7.15	7	1	2	mask	281	6	control	357	256.872
6014	han	7.15	7	1	2	mask	323	3	control	332	256.872
6015	han	7.15	7	1	2	mask	324	3	control	310	256.872
6016	han	7.15	7	1	2	mask	325	4	mask_woma	346	256.872
6024	han	7.15	7	1	2	mask	333	2	control	374	256.872
6047	han	7.15	7	1	2	mask	356	5	control	318	256.872
6049	han	7.15	7	1	2	mask	358	5	control	359	256.872
6050	han	7.15	7	1	2	mask	359	3	control	499	256.872

6051	han	7.15	7	1	2	mask	360	5	control	402	256.872
6053	han	7.15	7	1	2	mask	362	6	control	364	256.872
6068	han	7.15	7	1	2	object	15	2	control	411	256.872
6090	han	7.15	7	1	2	object	37	2	control	381	256.872
6091	han	7.15	7	1	2	object	38	2	control	464	256.872
6125	han	7.15	7	1	2	object	72	3	control	337	256.872
6133	han	7.15	7	1	2	object	80	3	control	500	256.872
6134	han	7.15	7	1	2	object	81	1	control	351	256.872
6148	han	7.15	7	1	2	object	95	1	control	962	256.872
6149	han	7.15	7	1	2	object	96	2	control	794	256.872
6155	han	7.15	7	1	2	object	102	3	control	332	256.872
6157	han	7.15	7	1	2	object	104	4	control	607	256.872
6170	han	7.15	7	1	2	object	117	3	control	NA	256.872
6177	han	7.15	7	1	2	object	124	5	control	NA	256.872
6178	han	7.15	7	1	2	object	125	4	control	499	256.872
6207	han	7.15	7	1	2	object	154	5	control	384	256.872
6211	han	7.15	7	1	2	object	158	4	control	NA	256.872
6212	han	7.15	7	1	2	object	159	2	net	NA	256.872
6214	han	7.15	7	1	2	object	161	6	control	775	256.872
6215	han	7.15	7	1	2	object	162	1	control	694	256.872
6216	han	7.15	7	1	2	object	163	6	broom	NA	256.872
6217	han	7.15	7	1	2	object	164	4	control	422	256.872
6218	han	7.15	7	1	2	object	165	6	control	397	256.872
6268	han	7.15	7	1	2	object	215	1	control	680	256.872
6269	han	7.15	7	1	2	object	216	3	broom	376	256.872
6270	han	7.15	7	1	2	object	217	6	control	424	256.872
6271	han	7.15	7	1	2	object	218	6	control	340	256.872
6272	han	7.15	7	1	2	object	219	3	control	831	256.872
6273	han	7.15	7	1	2	object	220	4	net	371	256.872
6274	han	7.15	7	1	2	object	221	6	control	328	256.872
6277	han	7.15	7	1	2	object	224	5	control	343	256.872
6285	han	7.15	7	1	2	object	232	4	control	445	256.872
6286	han	7.15	7	1	2	object	233	1	control	566	256.872
6287	han	7.15	7	1	2	object	234	1	control	409	256.872
6289	han	7.15	7	1	2	object	236	5	control	398	256.872
6294	han	7.15	7	1	2	object	241	5	glove	NA	256.872
6295	han	7.15	7	1	2	object	242	2	control	420	256.872
6318	han	7.15	7	1	2	object	265	6	glove	558	256.872
6319	han	7.15	7	1	2	object	266	4	control	324	256.872
6320	han	7.15	7	1	2	object	267	4	control	655	256.872
6321	han	7.15	7	1	2	object	268	4	broom	340	256.872
6322	han	7.15	7	1	2	object	269	1	control	378	256.872
6324	han	7.15	7	1	2	object	271	3	control	412	256.872
6326	han	7.15	7	1	2	object	273	2	control	567	256.872
6332	han	7.15	7	1	2	object	279	1	control	476	256.872
6359	han	7.15	7	1	2	object	306	4	control	NA	256.872
6360	han	7.15	7	1	2	object	307	1	net	952	256.872
6361	han	7.15	7	1	2	object	308	3	control	559	256.872
6362	han	7.15	7	1	2	object	309	3	net	557	256.872
6363	han	7.15	7	1	2	object	310	6	control	321	256.872

6369	han	7.15	7	1	2	object	316	4	control	324	256.872
6396	han	7.15	7	1	2	object	343	2	control	404	256.872
6397	han	7.15	7	1	2	object	344	6	control	430	256.872
6400	han	7.15	7	1	2	object	347	5	control	492	256.872
6401	han	7.15	7	1	2	object	348	6	control	506	256.872
6406	han	7.15	7	1	2	object	353	6	control	483	256.872
6407	han	7.15	7	1	2	object	354	5	control	467	256.872
6418	han	7.15	7	1	2	object	365	5	broom	646	256.872
6419	han	7.15	7	1	2	object	366	6	control	396	256.872
6420	han	7.15	7	1	2	object	367	5	control	437	256.872
6421	han	7.15	7	1	2	object	368	2	control	399	256.872
6423	han	7.15	7	1	2	object	370	5	control	338	256.872
6424	han	7.15	7	1	2	object	371	3	control	905	256.872
6425	han	7.15	7	1	2	object	372	1	control	372	256.872
6432	han	7.15	7	1	2	object	379	6	net	363	256.872
6433	han	7.15	7	1	2	object	380	4	control	494	256.872
6434	han	7.15	7	1	2	object	381	6	control	367	256.872
6436	han	7.15	7	1	2	object	383	3	control	413	256.872
6439	han	7.15	7	1	2	object	386	4	glove	747	256.872
6483	han	7.15	7	1	2	object	430	6	control	NA	256.872
6484	han	7.15	7	1	2	object	431	3	control	403	256.872
6522	han	7.15	7	1	2	object	469	2	control	403	256.872
6523	han	7.15	7	1	2	object	470	2	broom	388	256.872
6524	han	7.15	7	1	2	object	471	3	control	332	256.872
6525	han	7.15	7	1	2	object	472	2	control	321	256.872
6527	han	7.15	7	1	2	object	474	2	control	404	256.872
6528	han	7.15	7	1	2	object	475	2	control	335	256.872
6529	han	7.15	7	1	2	object	476	1	broom	446	256.872
6530	han	7.15	7	1	2	object	477	3	control	382	256.872
6537	han	7.15	7	1	2	object	484	3	control	378	256.872
6538	han	7.15	7	1	2	object	485	3	control	424	256.872
6540	han	7.15	7	1	2	object	487	4	control	456	256.872
6542	han	7.15	7	1	2	object	489	2	control	634	256.872
6543	han	7.15	7	1	2	object	490	4	control	493	256.872
6544	han	7.15	7	1	2	object	491	2	control	436	256.872
6546	han	7.15	7	1	2	object	493	1	control	405	256.872
6548	han	7.15	7	1	2	object	495	1	glove	410	256.872
6549	han	7.15	7	1	2	object	496	1	control	499	256.872
6550	han	7.15	7	1	2	object	497	2	control	487	256.872
6552	han	7.15	7	1	2	object	499	5	control	405	256.872
6625	han	7.15	7	1	2	object	572	1	control	NA	256.872
6626	han	7.15	7	1	2	object	573	3	glove	447	256.872
6630	han	7.15	7	1	2	object	577	5	control	371	256.872
6631	han	7.15	7	1	2	object	578	5	control	NA	256.872
6633	han	7.15	7	1	2	object	580	2	glove	405	256.872
6634	han	7.15	7	1	2	object	581	5	control	366	256.872
6636	han	7.15	7	1	2	object	583	2	control	NA	256.872
6680	han	7.15	7	1	2	object	627	3	control	431	256.872
6681	han	7.15	7	1	2	object	628	1	control	380	256.872
6682	han	7.15	7	1	2	object	629	4	control	355	256.872

6683	kas	11.21	7	1	2	mask	1	1	control	450	479.6
6684	kas	11.21	7	1	2	mask	2	4	control	391	479.6
6694	kas	11.21	7	1	2	mask	12	6	control	541	479.6
6710	kas	11.21	7	1	2	mask	28	3	control	599	479.6
6718	kas	11.21	7	1	2	mask	36	5	control	409	479.6
6723	kas	11.21	7	1	2	mask	41	3	control	427	479.6
6749	kas	11.21	7	1	2	mask	67	5	mask_woma	357	479.6
6768	kas	11.21	7	1	2	mask	86	2	control	407	479.6
6814	kas	11.21	7	1	2	mask	132	2	mask_woma	519	479.6
6815	kas	11.21	7	1	2	mask	133	4	control	475	479.6
6817	kas	11.21	7	1	2	mask	135	6	control	684	479.6
6818	kas	11.21	7	1	2	mask	136	1	control	462	479.6
6819	kas	11.21	7	1	2	mask	137	2	control	491	479.6
6820	kas	11.21	7	1	2	mask	138	5	mask_woma	420	479.6
6825	kas	11.21	7	1	2	mask	143	4	control	495	479.6
6839	kas	11.21	7	1	2	mask	157	1	control	644	479.6
6841	kas	11.21	7	1	2	mask	159	5	control	498	479.6
6842	kas	11.21	7	1	2	mask	160	1	control	519	479.6
6843	kas	11.21	7	1	2	mask	161	6	control	445	479.6
6849	kas	11.21	7	1	2	mask	167	6	control	416	479.6
6858	kas	11.21	7	1	2	mask	176	4	control	417	479.6
6859	kas	11.21	7	1	2	mask	177	3	mask_woma	525	479.6
6873	kas	11.21	7	1	2	mask	191	4	control	439	479.6
6880	kas	11.21	7	1	2	mask	198	6	control	465	479.6
6882	kas	11.21	7	1	2	mask	200	1	control	476	479.6
6883	kas	11.21	7	1	2	mask	201	1	control	491	479.6
6884	kas	11.21	7	1	2	mask	202	2	control	470	479.6
6886	kas	11.21	7	1	2	mask	204	5	control	504	479.6
6888	kas	11.21	7	1	2	mask	206	5	control	586	479.6
6889	kas	11.21	7	1	2	mask	207	4	mask_woma	511	479.6
6890	kas	11.21	7	1	2	mask	208	6	control	405	479.6
6916	kas	11.21	7	1	2	mask	234	1	control	485	479.6
6917	kas	11.21	7	1	2	mask	235	2	control	453	479.6
6982	kas	11.21	7	1	2	mask	300	2	control	514	479.6
6983	kas	11.21	7	1	2	mask	301	1	control	543	479.6
6985	kas	11.21	7	1	2	mask	303	3	control	718	479.6
6986	kas	11.21	7	1	2	mask	304	4	control	462	479.6
6987	kas	11.21	7	1	2	mask	305	3	control	451	479.6
6990	kas	11.21	7	1	2	mask	308	6	control	444	479.6
6991	kas	11.21	7	1	2	mask	309	6	control	427	479.6
6992	kas	11.21	7	1	2	mask	310	6	mask_woma	462	479.6
6993	kas	11.21	7	1	2	mask	311	5	control	332	479.6
6994	kas	11.21	7	1	2	mask	312	2	control	502	479.6
6995	kas	11.21	7	1	2	mask	313	2	mask_woma	642	479.6
6996	kas	11.21	7	1	2	mask	314	1	control	511	479.6
6997	kas	11.21	7	1	2	mask	315	2	control	1023	479.6
6998	kas	11.21	7	1	2	mask	316	3	control	476	479.6
7009	kas	11.21	7	1	2	mask	327	3	control	416	479.6
7010	kas	11.21	7	1	2	mask	328	6	control	415	479.6
7027	kas	11.21	7	1	2	mask	345	3	control	406	479.6

7428	kas	11.21	7	1	2	mask	1	2	control	428	479.6
7429	kas	11.21	7	1	2	mask	2	3	control	354	479.6
7430	kas	11.21	7	1	2	mask	3	6	control	415	479.6
7434	kas	11.21	7	1	2	mask	7	5	control	326	479.6
7437	kas	11.21	7	1	2	mask	10	5	control	351	479.6
7443	kas	11.21	7	1	2	mask	16	3	control	700	479.6
7451	kas	11.21	7	1	2	mask	24	3	control	565	479.6
7457	kas	11.21	7	1	2	mask	30	3	control	506	479.6
7471	kas	11.21	7	1	2	mask	44	1	control	517	479.6
7472	kas	11.21	7	1	2	mask	45	1	control	395	479.6
7485	kas	11.21	7	1	2	mask	58	2	mask_woma	428	479.6
7486	kas	11.21	7	1	2	mask	59	4	control	430	479.6
7487	kas	11.21	7	1	2	mask	60	4	control	386	479.6
7535	kas	11.21	7	1	2	mask	108	4	control	514	479.6
7558	kas	11.21	7	1	2	mask	131	2	control	399	479.6
7559	kas	11.21	7	1	2	mask	132	3	control	426	479.6
7560	kas	11.21	7	1	2	mask	133	3	mask_woma	470	479.6
7562	kas	11.21	7	1	2	mask	135	4	control	572	479.6
7563	kas	11.21	7	1	2	mask	136	3	mask_woma	496	479.6
7564	kas	11.21	7	1	2	mask	137	6	control	380	479.6
7597	kas	11.21	7	1	2	mask	170	2	control	399	479.6
7599	kas	11.21	7	1	2	mask	172	2	control	444	479.6
7600	kas	11.21	7	1	2	mask	173	5	control	418	479.6
7601	kas	11.21	7	1	2	mask	174	4	control	416	479.6
7639	kas	11.21	7	1	2	mask	212	5	control	515	479.6
7668	kas	11.21	7	1	2	mask	241	2	control	400	479.6
7670	kas	11.21	7	1	2	mask	243	1	control	445	479.6
7671	kas	11.21	7	1	2	mask	244	2	control	415	479.6
7672	kas	11.21	7	1	2	mask	245	3	mask_woma	548	479.6
7673	kas	11.21	7	1	2	mask	246	4	control	415	479.6
7674	kas	11.21	7	1	2	mask	247	6	control	366	479.6
7675	kas	11.21	7	1	2	mask	248	5	control	434	479.6
7677	kas	11.21	7	1	2	mask	250	4	control	535	479.6
7678	kas	11.21	7	1	2	mask	251	6	control	426	479.6
7679	kas	11.21	7	1	2	mask	252	3	control	453	479.6
7680	kas	11.21	7	1	2	mask	253	2	mask_woma	409	479.6
7727	kas	11.21	7	1	2	mask	300	6	control	487	479.6
7767	kas	11.21	7	1	2	mask	340	4	control	460	479.6
7768	kas	11.21	7	1	2	mask	341	6	control	370	479.6
7770	kas	11.21	7	1	2	mask	343	1	control	437	479.6
7771	kas	11.21	7	1	2	mask	344	1	mask_woma	425	479.6
7772	kas	11.21	7	1	2	mask	345	5	control	380	479.6
7773	kas	11.21	7	1	2	mask	346	1	control	430	479.6
7774	kas	11.21	7	1	2	mask	347	5	mask_woma	359	479.6
7775	kas	11.21	7	1	2	mask	348	3	control	426	479.6
7776	kas	11.21	7	1	2	mask	349	1	control	505	479.6
7777	kas	11.21	7	1	2	mask	350	4	control	390	479.6
7779	kas	11.21	7	1	2	mask	352	5	control	486	479.6
7804	kas	11.21	7	1	2	mask	377	6	mask_woma	488	479.6
7805	kas	11.21	7	1	2	mask	378	1	control	365	479.6

7818	kas	11.21	7	1	2	mask	391	2	control	493	479.6
7835	kas	11.21	7	1	2	mask	408	4	control	519	479.6
7852	kas	11.21	7	1	2	mask	425	6	control	300	479.6
7853	kas	11.21	7	1	2	mask	426	5	control	617	479.6
7854	kas	11.21	7	1	2	mask	427	2	control	396	479.6
7855	kas	11.21	7	1	2	mask	428	5	mask_woma	381	479.6
7856	kas	11.21	7	1	2	mask	429	1	control	519	479.6
7857	kas	11.21	7	1	2	mask	430	3	control	427	479.6
7858	kas	11.21	7	1	2	mask	431	3	control	523	479.6
7860	kas	11.21	7	1	2	mask	433	4	control	440	479.6
7861	kas	11.21	7	1	2	mask	434	5	control	458	479.6
7862	kas	11.21	7	1	2	mask	435	6	control	469	479.6
7864	kas	11.21	7	1	2	mask	437	3	control	488	479.6
7865	kas	11.21	7	1	2	mask	438	2	mask_woma	505	479.6
7866	kas	11.21	7	1	2	mask	439	5	control	400	479.6
7894	kas	11.21	7	1	2	mask	467	1	control	474	479.6
7912	kas	11.21	7	1	2	mask	485	5	control	1341	479.6
7923	kas	11.21	7	1	2	mask	496	1	mask_woma	NA	479.6
7924	kas	11.21	7	1	2	mask	497	3	control	530	479.6
7925	kas	11.21	7	1	2	mask	498	4	mask_woma	443	479.6
7926	kas	11.21	7	1	2	mask	499	6	control	527	479.6
7932	kas	11.21	7	1	2	mask	505	6	control	426	479.6
7933	kas	11.21	7	1	2	mask	506	5	mask_woma	427	479.6
7934	kas	11.21	7	1	2	mask	507	3	control	617	479.6
7936	kas	11.21	7	1	2	mask	509	6	control	434	479.6
7937	kas	11.21	7	1	2	mask	510	5	control	499	479.6
7938	kas	11.21	7	1	2	mask	511	1	control	520	479.6
7941	kas	11.21	7	1	2	mask	514	6	mask_woma	521	479.6
7942	kas	11.21	7	1	2	mask	515	4	control	435	479.6
7943	kas	11.21	7	1	2	mask	516	2	control	387	479.6
7953	kas	11.21	7	1	2	mask	526	5	control	524	479.6
7954	kas	11.21	7	1	2	mask	527	6	control	445	479.6
7955	kas	11.21	7	1	2	mask	528	4	mask_woma	417	479.6
7956	kas	11.21	7	1	2	mask	529	3	control	502	479.6
7957	kas	11.21	7	1	2	mask	530	2	control	610	479.6
7958	kas	11.21	7	1	2	mask	531	1	mask_woma	447	479.6
7959	kas	11.21	7	1	2	mask	532	6	control	427	479.6
7960	kas	11.21	7	1	2	mask	533	2	control	603	479.6
7961	kas	11.21	7	1	2	mask	534	5	control	402	479.6
7962	kas	11.21	7	1	2	mask	535	4	mask_woma	550	479.6
7966	kas	11.21	7	1	2	mask	539	1	control	515	479.6
7987	kas	11.21	7	1	2	mask	560	1	control	479	479.6
7992	kas	11.21	7	1	2	mask	565	2	control	492	479.6
7993	kas	11.21	7	1	2	mask	566	2	control	469	479.6
7994	kas	11.21	7	1	2	mask	567	6	mask_woma	384	479.6
7995	kas	11.21	7	1	2	mask	568	6	control	407	479.6
7997	kas	11.21	7	1	2	mask	570	3	control	438	479.6
8001	kas	11.21	7	1	2	mask	574	1	control	467	479.6
8002	kas	11.21	7	1	2	mask	575	3	control	440	479.6
8003	kas	11.21	7	1	2	mask	576	5	control	399	479.6

8004	kas	11.21	7	1	2	object	1	1	control	418	479.6
8006	kas	11.21	7	1	2	object	3	6	control	460	479.6
8028	kas	11.21	7	1	2	object	25	1	control	490	479.6
8032	kas	11.21	7	1	2	object	29	2	control	524	479.6
8046	kas	11.21	7	1	2	object	43	3	control	450	479.6
8047	kas	11.21	7	1	2	object	44	6	control	354	479.6
8059	kas	11.21	7	1	2	object	56	3	net	597	479.6
8060	kas	11.21	7	1	2	object	57	4	control	510	479.6
8063	kas	11.21	7	1	2	object	60	3	control	524	479.6
8064	kas	11.21	7	1	2	object	61	6	control	437	479.6
8080	kas	11.21	7	1	2	object	77	1	broom	545	479.6
8081	kas	11.21	7	1	2	object	78	6	control	473	479.6
8087	kas	11.21	7	1	2	object	84	2	control	447	479.6
8099	kas	11.21	7	1	2	object	96	1	control	529	479.6
8104	kas	11.21	7	1	2	object	101	1	net	1135	479.6
8105	kas	11.21	7	1	2	object	102	3	control	526	479.6
8108	kas	11.21	7	1	2	object	105	5	control	447	479.6
8112	kas	11.21	7	1	2	object	109	6	control	461	479.6
8115	kas	11.21	7	1	2	object	112	2	control	481	479.6
8118	kas	11.21	7	1	2	object	115	1	control	492	479.6
8140	kas	11.21	7	1	2	object	137	3	control	467	479.6
8141	kas	11.21	7	1	2	object	138	3	control	444	479.6
8150	kas	11.21	7	1	2	object	147	5	control	421	479.6
8151	kas	11.21	7	1	2	object	148	4	glove	419	479.6
8152	kas	11.21	7	1	2	object	149	5	control	443	479.6
8153	kas	11.21	7	1	2	object	150	5	control	450	479.6
8161	kas	11.21	7	1	2	object	158	2	control	422	479.6
8187	kas	11.21	7	1	2	object	184	6	control	425	479.6
8188	kas	11.21	7	1	2	object	185	4	control	476	479.6
8190	kas	11.21	7	1	2	object	187	6	control	411	479.6
8218	kas	11.21	7	1	2	object	215	3	control	467	479.6
8219	kas	11.21	7	1	2	object	216	6	control	415	479.6
8224	kas	11.21	7	1	2	object	221	4	control	449	479.6
8245	kas	11.21	7	1	2	object	242	4	control	471	479.6
8246	kas	11.21	7	1	2	object	243	5	control	439	479.6
8247	kas	11.21	7	1	2	object	244	1	control	527	479.6
8248	kas	11.21	7	1	2	object	245	5	net	445	479.6
8249	kas	11.21	7	1	2	object	246	3	control	467	479.6
8259	kas	11.21	7	1	2	object	256	2	control	866	479.6
8272	kas	11.21	7	1	2	object	269	5	broom	438	479.6
8273	kas	11.21	7	1	2	object	270	6	control	430	479.6
8286	kas	11.21	7	1	2	object	283	5	glove	487	479.6
8287	kas	11.21	7	1	2	object	284	1	control	541	479.6
8288	kas	11.21	7	1	2	object	285	4	control	419	479.6
8318	kas	11.21	7	1	2	object	315	4	broom	479	479.6
8319	kas	11.21	7	1	2	object	316	2	control	653	479.6
8335	kas	11.21	7	1	2	object	332	6	glove	NA	479.6
8336	kas	11.21	7	1	2	object	333	1	control	480	479.6
8337	kas	11.21	7	1	2	object	334	4	control	417	479.6
8338	kas	11.21	7	1	2	object	335	4	control	501	479.6

8340	kas	11.21	7	1	2	object	337	1	control	446	479.6
8364	kas	11.21	7	1	2	object	361	2	net	540	479.6
8365	kas	11.21	7	1	2	object	362	4	control	480	479.6
8366	kas	11.21	7	1	2	object	363	1	control	466	479.6
8467	kas	11.21	7	1	2	object	464	5	control	540	479.6
8469	kas	11.21	7	1	2	object	466	6	control	379	479.6
8470	kas	11.21	7	1	2	object	467	2	control	468	479.6
8471	kas	11.21	7	1	2	object	468	5	control	434	479.6
8473	kas	11.21	7	1	2	object	470	6	control	480	479.6
8475	kas	11.21	7	1	2	object	472	2	control	513	479.6
8476	kas	11.21	7	1	2	object	473	4	control	427	479.6
8488	kas	11.21	7	1	2	object	485	1	control	2666	479.6
8490	kas	11.21	7	1	2	object	487	2	control	485	479.6
8491	kas	11.21	7	1	2	object	488	6	control	456	479.6
8545	kas	11.21	7	1	2	object	542	4	control	645	479.6
8546	kas	11.21	7	1	2	object	543	2	broom	488	479.6
8547	kas	11.21	7	1	2	object	544	1	control	NA	479.6
8549	kas	11.21	7	1	2	object	546	2	control	595	479.6
8550	kas	11.21	7	1	2	object	547	3	control	605	479.6
8555	kas	11.21	7	1	2	object	552	3	broom	1256	479.6
8556	kas	11.21	7	1	2	object	553	5	control	387	479.6
8557	kas	11.21	7	1	2	object	554	5	control	NA	479.6
8558	kas	11.21	7	1	2	object	555	2	glove	NA	479.6
8559	kas	11.21	7	1	2	object	556	3	control	459	479.6
8560	kas	11.21	7	1	2	object	557	5	control	NA	479.6
8561	kas	11.21	7	1	2	object	558	3	glove	573	479.6
8567	kas	11.21	7	1	2	object	564	3	control	470	479.6
8568	kas	11.21	7	1	2	object	565	2	control	478	479.6
8569	kas	11.21	7	1	2	object	566	6	broom	383	479.6
8576	kas	11.21	7	1	2	object	573	3	control	406	479.6
8591	kas	11.21	7	1	2	object	588	3	control	1049	479.6
8593	kas	11.21	7	1	2	object	590	5	control	408	479.6
8594	kas	11.21	7	1	2	object	591	4	control	488	479.6
8596	kas	11.21	7	1	2	object	593	5	control	461	479.6
8635	kas	11.21	7	1	2	object	632	1	glove	408	479.6
8636	kas	11.21	7	1	2	object	633	3	control	578	479.6
8637	kas	11.21	7	1	2	object	634	1	control	449	479.6
8638	kas	11.21	7	1	2	object	635	2	control	411	479.6
8640	kas	11.21	7	1	2	object	637	4	net	422	479.6
8646	kas	11.21	7	1	2	object	643	4	control	661	479.6
8647	kas	11.21	7	1	2	object	644	2	control	473	479.6
8660	kas	11.21	7	1	2	object	657	6	control	377	479.6
8661	kas	11.21	7	1	2	object	658	6	net	424	479.6
8662	kas	11.21	7	1	2	object	659	1	control	488	479.6
8663	kas	11.21	7	1	2	object	660	1	control	465	479.6
8665	kas	11.21	7	1	2	object	662	2	control	456	479.6
8674	kas	11.21	7	1	2	object	671	2	control	550	479.6
8676	kas	11.21	7	1	2	object	673	1	control	530	479.6
8677	kas	11.21	7	1	2	object	674	5	control	436	479.6
8678	kas	11.21	7	1	2	object	675	3	control	443	479.6

8784	lou	6.36	2	2	2	object	106	1	control	1247	357.091
8823	lou	6.36	2	2	2	object	145	4	control	NA	357.091
8910	lou	6.36	2	2	2	object	232	3	control	NA	357.091
8936	lou	6.36	2	2	2	object	258	6	control	671	357.091
9002	lou	6.36	2	2	2	object	324	3	control	790	357.091
9021	lou	6.36	2	2	2	object	343	2	control	881	357.091
9076	lou	6.36	2	2	2	object	398	2	control	539	357.091
9092	lou	6.36	2	2	2	object	414	5	control	557	357.091
9099	lou	6.36	2	2	2	object	421	3	control	526	357.091
9107	lou	6.36	2	2	2	object	429	3	glove	1143	357.091
9108	lou	6.36	2	2	2	object	430	3	control	577	357.091
9110	lou	6.36	2	2	2	object	432	2	control	523	357.091
9116	lou	6.36	2	2	2	object	438	4	control	512	357.091
9118	lou	6.36	2	2	2	object	440	5	control	521	357.091
9142	lou	6.36	2	2	2	object	464	1	control	476	357.091
9144	lou	6.36	2	2	2	object	466	1	control	501	357.091
9157	lou	6.36	2	2	2	object	479	1	net	1859	357.091
9158	lou	6.36	2	2	2	object	480	3	control	NA	357.091
9161	lou	6.36	2	2	2	object	483	1	control	894	357.091
9162	lou	6.36	2	2	2	object	484	5	control	545	357.091
9164	lou	6.36	2	2	2	object	486	6	control	415	357.091
9171	lou	6.36	2	2	2	object	493	5	control	577	357.091
9173	lou	6.36	2	2	2	object	495	4	control	531	357.091
9174	lou	6.36	2	2	2	object	496	3	net	1460	357.091
9175	lou	6.36	2	2	2	object	497	2	control	552	357.091
9176	lou	6.36	2	2	2	object	498	6	control	447	357.091
9178	lou	6.36	2	2	2	object	500	5	control	457	357.091
9181	lou	6.36	2	2	2	object	503	5	glove	1028	357.091
9182	lou	6.36	2	2	2	object	504	2	control	420	357.091
9183	lou	6.36	2	2	2	object	505	6	control	434	357.091
9184	lou	6.36	2	2	2	object	506	1	control	543	357.091
9187	lou	6.36	2	2	2	object	509	4	net	943	357.091
9188	lou	6.36	2	2	2	object	510	2	control	522	357.091
9189	lou	6.36	2	2	2	object	511	4	control	508	357.091
9192	lou	6.36	2	2	2	object	514	6	control	482	357.091
9194	lou	6.36	2	2	2	object	516	4	control	710	357.091
9199	lou	6.36	2	2	2	object	521	3	control	NA	357.091
9201	lou	6.36	2	2	2	object	523	5	control	616	357.091
9205	lou	6.36	2	2	2	object	527	6	control	NA	357.091
9206	lou	6.36	2	2	2	object	528	1	control	981	357.091
9210	lou	6.36	2	2	2	object	532	5	net	922	357.091
9211	lou	6.36	2	2	2	object	533	3	control	923	357.091
9215	lou	6.36	2	2	2	object	537	2	control	713	357.091
9216	lou	6.36	2	2	2	object	538	1	control	651	357.091
9220	lou	6.36	2	2	2	object	542	6	control	640	357.091
9221	lou	6.36	2	2	2	object	543	4	control	NA	357.091
9222	lou	6.36	2	2	2	object	544	4	control	NA	357.091
9228	lou	6.36	2	2	2	object	550	6	net	1063	357.091
9229	lou	6.36	2	2	2	object	551	2	control	NA	357.091
9235	lou	6.36	2	2	2	object	557	5	control	NA	357.091

9236	lou	6.36	2	2	2	object	558	5	broom	1291	357.091
9237	lou	6.36	2	2	2	object	559	6	control	1556	357.091
9241	lou	6.36	2	2	2	object	563	5	control	NA	357.091
9242	lou	6.36	2	2	2	object	564	1	control	782	357.091
9244	lou	6.36	2	2	2	object	566	3	control	NA	357.091
9248	lou	6.36	2	2	2	object	570	6	control	632	357.091
9252	lou	6.36	2	2	2	object	574	4	broom	NA	357.091
9256	lou	6.36	2	2	2	object	578	1	control	651	357.091
9257	lou	6.36	2	2	2	object	579	6	control	543	357.091
9266	lou	6.36	2	2	2	object	588	2	control	675	357.091
9273	lou	6.36	2	2	2	object	595	4	control	582	357.091
9274	lou	6.36	2	2	2	object	596	1	control	579	357.091
9287	lou	6.36	2	2	2	object	609	6	control	464	357.091
9288	lou	6.36	2	2	2	object	610	1	glove	NA	357.091
9289	lou	6.36	2	2	2	object	611	1	control	536	357.091
9292	lou	6.36	2	2	2	object	614	6	glove	NA	357.091
9296	lou	6.36	2	2	2	object	618	3	control	NA	357.091
9301	lou	6.36	2	2	2	object	623	4	control	NA	357.091
9307	lou	6.36	2	2	2	object	629	5	control	NA	357.091
9323	lou	6.36	2	2	2	object	645	5	control	466	357.091
9324	lou	6.36	2	2	2	object	646	4	control	876	357.091
9333	lou	6.36	2	2	2	object	655	5	control	588	357.091
9349	lou	6.36	2	2	2	object	671	2	broom	NA	357.091
9350	lou	6.36	2	2	2	object	672	2	control	513	357.091
9351	lou	6.36	2	2	2	object	673	5	control	480	357.091
9352	lou	6.36	2	2	2	object	674	6	control	457	357.091
9366	lou	6.36	2	2	2	object	688	6	control	463	357.091
9367	lou	6.36	2	2	2	object	689	4	glove	NA	357.091
9373	lou	6.36	2	2	2	object	695	3	control	512	357.091
9383	lou	6.36	2	2	2	object	705	2	control	NA	357.091
9384	lou	6.36	2	2	2	object	706	1	broom	NA	357.091
9385	lou	6.36	2	2	2	object	707	4	control	542	357.091
9390	lou	6.36	2	2	2	object	712	1	control	575	357.091
9401	lou	6.36	2	2	2	object	723	2	net	1785	357.091
9402	lou	6.36	2	2	2	object	724	3	control	487	357.091
9408	lou	6.36	2	2	2	object	730	3	control	547	357.091
9409	lou	6.36	2	2	2	object	731	4	control	932	357.091
9418	lou	6.36	2	2	2	object	740	3	control	492	357.091
9426	lou	6.36	2	2	2	object	748	4	control	588	357.091
9427	lou	6.36	2	2	2	object	749	6	broom	2455	357.091
9428	lou	6.36	2	2	2	object	750	2	control	572	357.091
9437	lou	6.36	2	2	2	object	759	1	control	NA	357.091
9448	lou	6.36	2	2	2	object	770	3	control	607	357.091
9449	lou	6.36	2	2	2	object	771	3	broom	NA	357.091
9459	lou	6.36	2	2	2	object	781	1	control	NA	357.091
9465	lou	6.36	2	2	2	object	787	2	control	426	357.091
9469	lou	6.36	2	2	2	object	791	3	control	1315	357.091
9474	lou	6.36	2	2	2	object	796	2	control	454	357.091
9481	lou	6.36	2	2	2	object	803	2	control	554	357.091
9609	lou	6.36	2	2	2	mask	128	5	control	1414	357.091

9711	lou	6.36	2	2	2	mask	230	6	control	NA	357.091
9771	lou	6.36	2	2	2	mask	290	5	control	797	357.091
9824	lou	6.36	2	2	2	mask	343	4	control	NA	357.091
9825	lou	6.36	2	2	2	mask	344	3	control	659	357.091
9843	lou	6.36	2	2	2	mask	362	1	control	631	357.091
9859	lou	6.36	2	2	2	mask	378	2	control	NA	357.091
9903	lou	6.36	2	2	2	mask	422	4	mask_man	2042	357.091
9905	lou	6.36	2	2	2	mask	424	6	control	436	357.091
9906	lou	6.36	2	2	2	mask	425	5	control	505	357.091
9913	lou	6.36	2	2	2	mask	432	1	mask_man	1986	357.091
9914	lou	6.36	2	2	2	mask	433	1	control	NA	357.091
9938	lou	6.36	2	2	2	mask	457	1	control	566	357.091
9945	lou	6.36	2	2	2	mask	464	1	control	574	357.091
9966	lou	6.36	2	2	2	mask	485	1	control	686	357.091
9967	lou	6.36	2	2	2	mask	486	4	control	447	357.091
9968	lou	6.36	2	2	2	mask	487	2	control	443	357.091
9987	lou	6.36	2	2	2	mask	506	6	mask_man	NA	357.091
9988	lou	6.36	2	2	2	mask	507	2	control	449	357.091
10007	lou	6.36	2	2	2	mask	526	3	control	513	357.091
10017	lou	6.36	2	2	2	mask	536	4	control	NA	357.091
10028	lou	6.36	2	2	2	mask	547	3	mask_man	NA	357.091
10029	lou	6.36	2	2	2	mask	548	3	control	421	357.091
10034	lou	6.36	2	2	2	mask	553	1	mask_man	NA	357.091
10041	lou	6.36	2	2	2	mask	560	2	control	472	357.091
10077	lou	6.36	2	2	2	mask	596	5	control	NA	357.091
10087	lou	6.36	2	2	2	mask	606	3	control	514	357.091
10114	lou	6.36	2	2	2	mask	633	6	control	459	357.091
10133	lou	6.36	2	2	2	mask	652	5	control	1454	357.091
10134	lou	6.36	2	2	2	mask	653	5	mask_man	NA	357.091
10152	lou	6.36	2	2	2	mask	671	4	control	518	357.091
10175	lou	6.36	2	2	2	mask	694	5	control	500	357.091
10177	lou	6.36	2	2	2	mask	696	4	control	591	357.091
10206	lou	6.36	2	2	2	mask	725	6	mask_man	NA	357.091
10236	lou	6.36	2	2	2	mask	755	2	control	452	357.091
10258	lou	6.36	2	2	2	mask	777	3	mask_man	NA	357.091
10259	lou	6.36	2	2	2	mask	778	5	control	449	357.091
10270	lou	6.36	2	2	2	mask	789	4	control	585	357.091
10282	lou	6.36	2	2	2	mask	801	4	control	534	357.091
10283	lou	6.36	2	2	2	mask	802	4	control	813	357.091
10297	lou	6.36	2	2	2	mask	816	6	mask_man	NA	357.091
10298	lou	6.36	2	2	2	mask	817	2	control	486	357.091
10310	lou	6.36	2	2	2	mask	829	5	control	436	357.091
10311	lou	6.36	2	2	2	mask	830	4	control	NA	357.091
10320	lou	6.36	2	2	2	mask	839	3	mask_man	NA	357.091
10330	lou	6.36	2	2	2	mask	849	1	control	NA	357.091
10331	lou	6.36	2	2	2	mask	850	4	control	518	357.091
10341	lou	6.36	2	2	2	mask	860	2	control	435	357.091
10342	lou	6.36	2	2	2	mask	861	5	mask_man	NA	357.091
10353	lou	6.36	2	2	2	mask	872	1	control	453	357.091
10356	lou	6.36	2	2	2	mask	875	3	control	444	357.091

10445	lou	6.36	2	2	2	mask	85	1	control	NA	357.091
10499	lou	6.36	2	2	2	mask	139	6	control	NA	357.091
10510	lou	6.36	2	2	2	mask	150	3	control	553	357.091
10548	lou	6.36	2	2	2	mask	188	6	control	NA	357.091
10599	lou	6.36	2	2	2	mask	239	4	control	NA	357.091
10613	lou	6.36	2	2	2	mask	253	4	control	1050	357.091
10650	lou	6.36	2	2	2	mask	290	4	control	1179	357.091
10762	lou	6.36	2	2	2	mask	402	1	mask_man	NA	357.091
10763	lou	6.36	2	2	2	mask	403	6	control	523	357.091
10858	lou	6.36	2	2	2	mask	498	3	control	672	357.091
10859	lou	6.36	2	2	2	mask	499	2	control	500	357.091
10993	lou	6.36	2	2	2	mask	633	2	control	661	357.091
10994	lou	6.36	2	2	2	mask	634	5	mask_man	NA	357.091
10995	lou	6.36	2	2	2	mask	635	2	control	476	357.091
11016	lou	6.36	2	2	2	mask	656	1	control	654	357.091
11018	lou	6.36	2	2	2	mask	658	4	control	464	357.091
11020	lou	6.36	2	2	2	mask	660	1	control	562	357.091
11021	lou	6.36	2	2	2	mask	661	6	mask_man	1560	357.091
11034	lou	6.36	2	2	2	mask	674	5	control	634	357.091
11041	lou	6.36	2	2	2	mask	681	6	control	461	357.091
11060	lou	6.36	2	2	2	mask	700	4	control	588	357.091
11061	lou	6.36	2	2	2	mask	701	1	control	508	357.091
11062	lou	6.36	2	2	2	mask	702	2	control	709	357.091
11063	lou	6.36	2	2	2	mask	703	1	mask_man	809	357.091
11093	lou	6.36	2	2	2	mask	733	2	control	533	357.091
11101	lou	6.36	2	2	2	mask	741	4	control	437	357.091
11106	lou	6.36	2	2	2	mask	746	5	control	463	357.091
11107	lou	6.36	2	2	2	mask	747	1	control	764	357.091
11140	lou	6.36	2	2	2	mask	780	5	control	532	357.091
11141	lou	6.36	2	2	2	mask	781	1	control	669	357.091
11142	lou	6.36	2	2	2	mask	782	6	mask_man	814	357.091
11143	lou	6.36	2	2	2	mask	783	2	control	567	357.091
11163	lou	6.36	2	2	2	mask	803	6	control	524	357.091
11165	lou	6.36	2	2	2	mask	805	4	control	490	357.091
11183	lou	6.36	2	2	2	mask	823	6	control	437	357.091
11185	lou	6.36	2	2	2	mask	825	2	mask_man	1365	357.091
11186	lou	6.36	2	2	2	mask	826	3	control	463	357.091
11194	lou	6.36	2	2	2	mask	834	2	control	479	357.091
11223	lou	6.36	2	2	2	mask	863	5	mask_man	761	357.091
11240	lou	6.36	2	2	2	mask	880	1	control	790	357.091
11242	lou	6.36	2	2	2	mask	882	4	control	537	357.091
11248	lou	6.36	2	2	2	mask	888	4	mask_man	706	357.091
11254	lou	6.36	2	2	2	mask	894	2	control	656	357.091
11260	lou	6.36	2	2	2	mask	900	5	mask_man	588	357.091
11261	lou	6.36	2	2	2	mask	901	5	control	469	357.091
11281	lou	6.36	2	2	2	mask	921	2	mask_man	666	357.091
11282	lou	6.36	2	2	2	mask	922	2	control	562	357.091
11283	lou	6.36	2	2	2	mask	923	3	control	471	357.091
11295	lou	6.36	2	2	2	mask	935	4	mask_man	605	357.091
11310	lou	6.36	2	2	2	mask	950	6	control	415	357.091

11311	lou	6.36	2	2	2	mask	951	5	control	442	357.091
11312	lou	6.36	2	2	2	mask	952	3	mask_man	652	357.091
11318	lou	6.36	2	2	2	mask	958	5	control	431	357.091
11319	lou	6.36	2	2	2	mask	959	3	control	481	357.091
11336	lou	6.36	2	2	2	mask	976	5	control	1552	357.091
11338	lou	6.36	2	2	2	mask	978	1	control	559	357.091
11353	lou	6.36	2	2	2	mask	993	1	control	586	357.091
11379	lou	6.36	2	2	2	mask	1019	5	control	449	357.091
11380	lou	6.36	2	2	2	mask	1020	3	control	409	357.091
11382	lou	6.36	2	2	2	mask	1022	5	control	436	357.091
11383	lou	6.36	2	2	2	mask	1023	4	control	418	357.091
11384	lou	6.36	2	2	2	mask	1024	5	control	395	357.091
11399	lou	6.36	2	2	2	mask	1039	4	control	421	357.091
11423	lou	6.36	2	2	2	mask	1063	2	control	570	357.091
11428	lou	6.36	2	2	2	mask	1068	5	control	479	357.091
11433	lou	6.36	2	2	2	mask	1073	5	control	498	357.091
11448	lou	6.36	2	2	2	mask	1088	6	mask_man	681	357.091
11450	lou	6.36	2	2	2	mask	1090	2	control	498	357.091
11451	lou	6.36	2	2	2	mask	1091	3	control	440	357.091
11455	lou	6.36	2	2	2	mask	1095	6	control	479	357.091
11460	lou	6.36	2	2	2	mask	1100	6	control	418	357.091
11484	lou	6.36	2	2	2	mask	1124	3	control	583	357.091
11497	lou	6.36	2	2	2	mask	1137	1	control	518	357.091
11506	lou	6.36	2	2	2	mask	1146	2	control	441	357.091
11507	lou	6.36	2	2	2	mask	1147	3	mask_man	809	357.091
11520	lou	6.36	2	2	2	mask	1160	1	control	508	357.091
11521	lou	6.36	2	2	2	mask	1161	6	control	391	357.091
11522	lou	6.36	2	2	2	mask	1162	6	control	376	357.091
11537	lou	6.36	2	2	2	mask	1177	3	control	NA	357.091
11538	lou	6.36	2	2	2	mask	1178	6	control	415	357.091
11539	lou	6.36	2	2	2	mask	1179	1	control	548	357.091
11548	lou	6.36	2	2	2	mask	1188	3	mask_man	523	357.091
11558	lou	6.36	2	2	2	mask	1198	4	control	563	357.091
11559	lou	6.36	2	2	2	mask	1199	4	control	456	357.091
11564	lou	6.36	2	2	2	mask	1204	6	control	436	357.091
11565	lou	6.36	2	2	2	mask	1205	2	mask_man	665	357.091
11570	lou	6.36	2	2	2	mask	1210	3	control	495	357.091
11571	lou	6.36	2	2	2	mask	1211	3	control	469	357.091
11576	lou	6.36	2	2	2	mask	1216	4	control	474	357.091
11577	lou	6.36	2	2	2	mask	1217	4	mask_man	656	357.091
11584	lou	6.36	2	2	2	mask	1224	6	control	487	357.091
11585	lou	6.36	2	2	2	mask	1225	1	mask_man	718	357.091
11593	lou	6.36	2	2	2	mask	1233	1	control	505	357.091
11594	lou	6.36	2	2	2	mask	1234	3	control	427	357.091
11622	lou	6.36	2	2	2	mask	1262	3	control	481	357.091
11624	lou	6.36	2	2	2	mask	1264	4	control	547	357.091
11632	lou	6.36	2	2	2	mask	1272	3	control	461	357.091
11633	lou	6.36	2	2	2	mask	1273	4	control	410	357.091
11646	lou	6.36	2	2	2	mask	1286	1	control	680	357.091
11647	lou	6.36	2	2	2	mask	1287	4	control	545	357.091

12392	pac	6.1	1	2	2	object	39	5	control	647	309.77
12393	pac	6.1	1	2	2	object	40	6	control	717	309.77
12394	pac	6.1	1	2	2	object	41	6	control	570	309.77
12395	pac	6.1	1	2	2	object	42	3	control	491	309.77
12397	pac	6.1	1	2	2	object	44	3	control	483	309.77
12398	pac	6.1	1	2	2	object	45	2	control	424	309.77
12399	pac	6.1	1	2	2	object	46	6	control	517	309.77
12400	pac	6.1	1	2	2	object	47	1	control	513	309.77
12402	pac	6.1	1	2	2	object	49	5	control	428	309.77
12420	pac	6.1	1	2	2	object	67	3	control	493	309.77
12421	pac	6.1	1	2	2	object	68	5	control	435	309.77
12422	pac	6.1	1	2	2	object	69	2	glove	496	309.77
12462	pac	6.1	1	2	2	object	109	6	control	422	309.77
12463	pac	6.1	1	2	2	object	110	3	control	466	309.77
12464	pac	6.1	1	2	2	object	111	1	control	414	309.77
12466	pac	6.1	1	2	2	object	113	6	control	392	309.77
12467	pac	6.1	1	2	2	object	114	6	control	417	309.77
12497	pac	6.1	1	2	2	object	144	1	control	396	309.77
12499	pac	6.1	1	2	2	object	146	2	control	424	309.77
12508	pac	6.1	1	2	2	object	155	3	control	488	309.77
12509	pac	6.1	1	2	2	object	156	4	control	401	309.77
12510	pac	6.1	1	2	2	object	157	2	broom	1249	309.77
12511	pac	6.1	1	2	2	object	158	5	control	385	309.77
12520	pac	6.1	1	2	2	object	167	4	control	443	309.77
12522	pac	6.1	1	2	2	object	169	6	control	575	309.77
12523	pac	6.1	1	2	2	object	170	2	control	1183	309.77
12541	pac	6.1	1	2	2	object	188	4	control	391	309.77
12543	pac	6.1	1	2	2	object	190	5	control	402	309.77
12544	pac	6.1	1	2	2	object	191	6	control	403	309.77
12545	pac	6.1	1	2	2	object	192	3	control	453	309.77
12584	pac	6.1	1	2	2	object	231	5	control	395	309.77
12585	pac	6.1	1	2	2	object	232	4	glove	396	309.77
12586	pac	6.1	1	2	2	object	233	5	control	417	309.77
12587	pac	6.1	1	2	2	object	234	3	control	404	309.77
12588	pac	6.1	1	2	2	object	235	6	broom	840	309.77
12589	pac	6.1	1	2	2	object	236	6	control	359	309.77
12590	pac	6.1	1	2	2	object	237	2	control	409	309.77
12591	pac	6.1	1	2	2	object	238	4	control	408	309.77
12601	pac	6.1	1	2	2	object	248	3	broom	655	309.77
12602	pac	6.1	1	2	2	object	249	6	control	728	309.77
12603	pac	6.1	1	2	2	object	250	1	control	382	309.77
12634	pac	6.1	1	2	2	object	281	3	control	457	309.77
12635	pac	6.1	1	2	2	object	282	1	control	397	309.77
12636	pac	6.1	1	2	2	object	283	3	glove	550	309.77
12637	pac	6.1	1	2	2	object	284	5	control	365	309.77
12638	pac	6.1	1	2	2	object	285	5	glove	389	309.77
12651	pac	6.1	1	2	2	object	298	3	control	443	309.77
12652	pac	6.1	1	2	2	object	299	2	control	380	309.77
12660	pac	6.1	1	2	2	object	307	2	control	397	309.77
12661	pac	6.1	1	2	2	object	308	4	control	381	309.77

12663	pac	6.1	1	2	2	object	310	5	control	380	309.77
12664	pac	6.1	1	2	2	object	311	4	control	401	309.77
12665	pac	6.1	1	2	2	object	312	6	control	396	309.77
12666	pac	6.1	1	2	2	object	313	5	net	1283	309.77
12667	pac	6.1	1	2	2	object	314	6	control	407	309.77
12681	pac	6.1	1	2	2	object	328	6	control	456	309.77
12682	pac	6.1	1	2	2	object	329	1	control	401	309.77
12683	pac	6.1	1	2	2	object	330	4	net	459	309.77
12684	pac	6.1	1	2	2	object	331	3	control	497	309.77
12686	pac	6.1	1	2	2	object	333	4	control	385	309.77
12687	pac	6.1	1	2	2	object	334	3	net	531	309.77
12688	pac	6.1	1	2	2	object	335	2	control	478	309.77
12689	pac	6.1	1	2	2	object	336	1	glove	499	309.77
12701	pac	6.1	1	2	2	object	348	6	control	490	309.77
12702	pac	6.1	1	2	2	object	349	1	control	503	309.77
12703	pac	6.1	1	2	2	object	350	1	control	424	309.77
12705	pac	6.1	1	2	2	object	352	1	control	486	309.77
12707	pac	6.1	1	2	2	object	354	4	control	350	309.77
12708	pac	6.1	1	2	2	object	355	6	glove	402	309.77
12709	pac	6.1	1	2	2	object	356	1	control	624	309.77
12710	pac	6.1	1	2	2	object	357	1	control	398	309.77
12711	pac	6.1	1	2	2	object	358	5	control	341	309.77
12712	pac	6.1	1	2	2	object	359	1	net	490	309.77
12713	pac	6.1	1	2	2	object	360	4	control	399	309.77
12792	pac	6.1	1	2	2	object	439	4	control	362	309.77
12793	pac	6.1	1	2	2	object	440	1	broom	497	309.77
12794	pac	6.1	1	2	2	object	441	2	control	376	309.77
12795	pac	6.1	1	2	2	object	442	3	control	365	309.77
12813	pac	6.1	1	2	2	object	460	5	broom	514	309.77
12814	pac	6.1	1	2	2	object	461	2	control	375	309.77
12815	pac	6.1	1	2	2	object	462	2	net	497	309.77
12816	pac	6.1	1	2	2	object	463	2	control	352	309.77
12826	pac	6.1	1	2	2	object	473	2	control	341	309.77
12827	pac	6.1	1	2	2	object	474	4	control	467	309.77
12828	pac	6.1	1	2	2	object	475	6	net	467	309.77
12829	pac	6.1	1	2	2	object	476	5	control	378	309.77
12830	pac	6.1	1	2	2	object	477	3	control	421	309.77
12841	pac	6.1	1	2	2	object	488	1	control	536	309.77
12842	pac	6.1	1	2	2	object	489	2	control	455	309.77
12856	pac	6.1	1	2	2	object	503	4	broom	732	309.77
12857	pac	6.1	1	2	2	object	504	3	control	370	309.77
12858	pac	6.1	1	2	2	object	505	4	control	376	309.77
12859	pac	6.1	1	2	2	object	506	5	control	359	309.77
12861	pac	6.1	1	2	2	object	508	3	control	362	309.77
12862	pac	6.1	1	2	2	object	509	5	control	356	309.77
12872	pac	6.1	1	2	2	object	519	2	control	398	309.77
12873	pac	6.1	1	2	2	object	520	3	control	661	309.77
12877	pac	6.1	1	2	2	object	524	3	control	356	309.77
12878	pac	6.1	1	2	2	object	525	2	control	424	309.77
12879	pac	6.1	1	2	2	object	526	4	control	412	309.77

12903	pac	6.1	1	2	2	mask	24	3	control	432	309.77
12904	pac	6.1	1	2	2	mask	25	1	control	397	309.77
12905	pac	6.1	1	2	2	mask	26	6	control	373	309.77
13030	pac	6.1	1	2	2	mask	151	4	control	448	309.77
13031	pac	6.1	1	2	2	mask	152	5	control	382	309.77
13032	pac	6.1	1	2	2	mask	153	3	control	416	309.77
13033	pac	6.1	1	2	2	mask	154	5	mask_man	611	309.77
13050	pac	6.1	1	2	2	mask	171	4	control	1120	309.77
13051	pac	6.1	1	2	2	mask	172	3	mask_man	430	309.77
13052	pac	6.1	1	2	2	mask	173	2	control	341	309.77
13091	pac	6.1	1	2	2	mask	212	4	control	378	309.77
13092	pac	6.1	1	2	2	mask	213	3	control	410	309.77
13094	pac	6.1	1	2	2	mask	215	5	control	348	309.77
13101	pac	6.1	1	2	2	mask	222	4	control	455	309.77
13102	pac	6.1	1	2	2	mask	223	1	control	363	309.77
13103	pac	6.1	1	2	2	mask	224	2	mask_man	340	309.77
13104	pac	6.1	1	2	2	mask	225	1	control	345	309.77
13129	pac	6.1	1	2	2	mask	250	6	control	370	309.77
13130	pac	6.1	1	2	2	mask	251	1	control	408	309.77
13136	pac	6.1	1	2	2	mask	257	3	control	501	309.77
13137	pac	6.1	1	2	2	mask	258	5	control	349	309.77
13287	pac	6.1	1	2	2	mask	408	3	mask_man	NA	309.77
13288	pac	6.1	1	2	2	mask	409	2	control	425	309.77
13289	pac	6.1	1	2	2	mask	410	1	control	402	309.77
13290	pac	6.1	1	2	2	mask	411	4	mask_man	455	309.77
13301	pac	6.1	1	2	2	mask	422	3	control	429	309.77
13302	pac	6.1	1	2	2	mask	423	5	control	385	309.77
13303	pac	6.1	1	2	2	mask	424	5	mask_man	412	309.77
13323	pac	6.1	1	2	2	mask	444	1	control	535	309.77
13324	pac	6.1	1	2	2	mask	445	1	mask_man	412	309.77
13358	pac	6.1	1	2	2	mask	479	5	control	610	309.77
13359	pac	6.1	1	2	2	mask	480	5	control	801	309.77
13375	pac	6.1	1	2	2	mask	496	3	control	447	309.77
13377	pac	6.1	1	2	2	mask	498	4	control	355	309.77
13399	pac	6.1	1	2	2	mask	520	5	control	414	309.77
13426	pac	6.1	1	2	2	mask	547	4	control	429	309.77
13427	pac	6.1	1	2	2	mask	548	6	control	523	309.77
13428	pac	6.1	1	2	2	mask	549	1	mask_man	433	309.77
13429	pac	6.1	1	2	2	mask	550	4	control	388	309.77
13430	pac	6.1	1	2	2	mask	551	2	control	342	309.77
13432	pac	6.1	1	2	2	mask	553	3	control	437	309.77
13444	pac	6.1	1	2	2	mask	565	2	control	626	309.77
13446	pac	6.1	1	2	2	mask	567	2	control	382	309.77
13495	pac	6.1	1	2	2	mask	616	1	control	380	309.77
13496	pac	6.1	1	2	2	mask	617	1	control	359	309.77
13497	pac	6.1	1	2	2	mask	618	2	control	357	309.77
13498	pac	6.1	1	2	2	mask	619	6	mask_man	498	309.77
13499	pac	6.1	1	2	2	mask	620	3	control	430	309.77
13501	pac	6.1	1	2	2	mask	622	6	control	352	309.77
13508	pac	6.1	1	2	2	mask	629	6	control	760	309.77

13509	pac	6.1	1	2	2	mask	630	5	control	541	309.77
13540	pac	6.1	1	2	2	mask	661	1	control	711	309.77
13551	pac	6.1	1	2	2	mask	672	5	control	368	309.77
13552	pac	6.1	1	2	2	mask	673	1	control	466	309.77
13554	pac	6.1	1	2	2	mask	675	2	control	367	309.77
13555	pac	6.1	1	2	2	mask	676	6	control	394	309.77
13563	pac	6.1	1	2	2	mask	684	2	mask_man	399	309.77
13564	pac	6.1	1	2	2	mask	685	6	control	331	309.77
13565	pac	6.1	1	2	2	mask	686	6	mask_man	429	309.77
13566	pac	6.1	1	2	2	mask	687	2	control	374	309.77
13567	pac	6.1	1	2	2	mask	688	3	control	447	309.77
13568	pac	6.1	1	2	2	mask	689	3	control	382	309.77
13569	pac	6.1	1	2	2	mask	690	3	mask_man	471	309.77
13570	pac	6.1	1	2	2	mask	691	6	control	389	309.77
13571	pac	6.1	1	2	2	mask	692	2	control	486	309.77
13572	pac	6.1	1	2	2	mask	693	1	mask_man	393	309.77
13599	pac	6.1	1	2	2	mask	720	6	control	395	309.77
13600	pac	6.1	1	2	2	mask	721	6	control	379	309.77
13601	pac	6.1	1	2	2	mask	722	1	control	356	309.77
13602	pac	6.1	1	2	2	mask	723	4	mask_man	374	309.77
13603	pac	6.1	1	2	2	mask	724	4	control	350	309.77
13609	pac	6.1	1	2	2	mask	730	2	control	371	309.77
13611	pac	6.1	1	2	2	mask	2	5	control	349	309.77
13612	pac	6.1	1	2	2	mask	3	4	control	495	309.77
13613	pac	6.1	1	2	2	mask	4	3	control	563	309.77
13673	pac	6.1	1	2	2	mask	64	5	control	380	309.77
13674	pac	6.1	1	2	2	mask	65	3	control	427	309.77
13675	pac	6.1	1	2	2	mask	66	2	control	381	309.77
13682	pac	6.1	1	2	2	mask	73	4	control	2856	309.77
13683	pac	6.1	1	2	2	mask	74	3	control	448	309.77
13685	pac	6.1	1	2	2	mask	76	5	control	382	309.77
13686	pac	6.1	1	2	2	mask	77	5	control	398	309.77
13687	pac	6.1	1	2	2	mask	78	5	mask_man	381	309.77
13688	pac	6.1	1	2	2	mask	79	5	control	360	309.77
13689	pac	6.1	1	2	2	mask	80	3	mask_man	416	309.77
13690	pac	6.1	1	2	2	mask	81	1	control	381	309.77
13718	pac	6.1	1	2	2	mask	109	4	control	1104	309.77
13719	pac	6.1	1	2	2	mask	110	2	control	1040	309.77
13721	pac	6.1	1	2	2	mask	112	6	control	483	309.77
13722	pac	6.1	1	2	2	mask	113	2	control	368	309.77
13723	pac	6.1	1	2	2	mask	114	1	mask_man	377	309.77
13724	pac	6.1	1	2	2	mask	115	3	control	371	309.77
13725	pac	6.1	1	2	2	mask	116	4	mask_man	380	309.77
13733	pac	6.1	1	2	2	mask	124	1	control	454	309.77
13811	pac	6.1	1	2	2	mask	202	4	control	379	309.77
13813	pac	6.1	1	2	2	mask	204	3	control	618	309.77
13814	pac	6.1	1	2	2	mask	205	3	control	420	309.77
13816	pac	6.1	1	2	2	mask	207	2	control	384	309.77
13818	pac	6.1	1	2	2	mask	209	5	control	347	309.77
13819	pac	6.1	1	2	2	mask	210	2	mask_man	424	309.77

13820	pac	6.1	1	2	2	mask	211	2	control	409	309.77
13836	pac	6.1	1	2	2	mask	227	5	control	NA	309.77
13837	pac	6.1	1	2	2	mask	228	6	control	377	309.77
13839	pac	6.1	1	2	2	mask	230	4	control	387	309.77
13840	pac	6.1	1	2	2	mask	231	5	mask_man	386	309.77
13841	pac	6.1	1	2	2	mask	232	1	control	393	309.77
13842	pac	6.1	1	2	2	mask	233	3	control	395	309.77
13843	pac	6.1	1	2	2	mask	234	1	control	457	309.77
13845	pac	6.1	1	2	2	mask	236	4	control	364	309.77
13846	pac	6.1	1	2	2	mask	237	1	control	360	309.77
13847	pac	6.1	1	2	2	mask	238	2	mask_man	402	309.77
13883	pac	6.1	1	2	2	mask	274	6	control	628	309.77
13884	pac	6.1	1	2	2	mask	275	1	mask_man	598	309.77
13885	pac	6.1	1	2	2	mask	276	3	control	2901	309.77
13886	pac	6.1	1	2	2	mask	277	1	control	395	309.77
13887	pac	6.1	1	2	2	mask	278	3	mask_man	417	309.77
13901	pac	6.1	1	2	2	mask	292	1	control	383	309.77
13903	pac	6.1	1	2	2	mask	294	5	control	656	309.77
13905	pac	6.1	1	2	2	mask	296	2	control	469	309.77
13907	pac	6.1	1	2	2	mask	298	6	control	368	309.77
13908	pac	6.1	1	2	2	mask	299	2	control	455	309.77
13939	pac	6.1	1	2	2	mask	330	1	control	362	309.77
13940	pac	6.1	1	2	2	mask	331	6	control	389	309.77
13942	pac	6.1	1	2	2	mask	333	4	control	332	309.77
13943	pac	6.1	1	2	2	mask	334	3	control	368	309.77
13944	pac	6.1	1	2	2	mask	335	2	control	358	309.77
13945	pac	6.1	1	2	2	mask	336	5	mask_man	417	309.77
13946	pac	6.1	1	2	2	mask	337	5	control	341	309.77
13947	pac	6.1	1	2	2	mask	338	6	control	348	309.77
13948	pac	6.1	1	2	2	mask	339	4	mask_man	411	309.77
13963	pac	6.1	1	2	2	mask	354	6	control	497	309.77
13964	pac	6.1	1	2	2	mask	355	5	control	364	309.77
13966	pac	6.1	1	2	2	mask	357	3	control	NA	309.77
13997	pac	6.1	1	2	2	mask	388	6	control	NA	309.77
14008	pac	6.1	1	2	2	mask	399	1	control	366	309.77
14009	pac	6.1	1	2	2	mask	400	4	control	446	309.77
14010	pac	6.1	1	2	2	mask	401	1	control	341	309.77
14046	pac	6.1	1	2	2	mask	437	2	mask_man	606	309.77
14047	pac	6.1	1	2	2	mask	438	5	control	341	309.77
14048	pac	6.1	1	2	2	mask	439	6	mask_man	1994	309.77
14049	pac	6.1	1	2	2	mask	440	5	control	425	309.77
14064	pac	6.1	1	2	2	mask	455	6	control	1518	309.77
14065	pac	6.1	1	2	2	mask	456	4	control	429	309.77
14066	pac	6.1	1	2	2	mask	457	4	mask_man	359	309.77
14067	pac	6.1	1	2	2	mask	458	3	control	388	309.77
14068	pac	6.1	1	2	2	mask	459	6	control	357	309.77
14069	pac	6.1	1	2	2	mask	460	4	control	421	309.77
14070	pac	6.1	1	2	2	mask	461	6	mask_man	442	309.77
14071	pac	6.1	1	2	2	mask	462	6	control	428	309.77
14072	pac	6.1	1	2	2	mask	463	4	control	494	309.77

14077	pac	6.1	1	2	2	mask	468	1	control	875	309.77
14078	pac	6.1	1	2	2	mask	469	3	mask_man	1033	309.77
14079	pac	6.1	1	2	2	mask	470	2	control	415	309.77
14097	pac	6.1	1	2	2	mask	488	3	control	428	309.77
14098	pac	6.1	1	2	2	mask	489	5	control	335	309.77
14100	pac	6.1	1	2	2	mask	491	2	control	564	309.77
14102	pac	6.1	1	2	2	mask	493	5	control	500	309.77
14103	pac	6.1	1	2	2	mask	494	6	control	393	309.77
14104	pac	6.1	1	2	2	mask	495	4	control	364	309.77
14106	pac	6.1	1	2	2	mask	497	1	control	395	309.77
14107	pac	6.1	1	2	2	mask	498	2	control	337	309.77
14112	pac	6.1	1	2	2	mask	503	6	mask_man	480	309.77
14113	pac	6.1	1	2	2	mask	504	3	control	381	309.77
14114	pac	6.1	1	2	2	mask	505	4	control	345	309.77
14115	pac	6.1	1	2	2	mask	506	2	control	477	309.77
14116	pac	6.1	1	2	2	mask	507	1	mask_man	2754	309.77
14117	pac	6.1	1	2	2	mask	508	3	control	407	309.77
14119	pac	6.1	1	2	2	mask	510	6	control	1387	309.77
14121	pac	6.1	1	2	2	mask	512	5	control	491	309.77
14122	pac	6.1	1	2	2	mask	513	5	control	334	309.77
14140	pac	6.1	1	2	2	mask	531	2	control	805	309.77
14141	pac	6.1	1	2	2	mask	532	4	control	332	309.77
14142	rio	5.73	2	2	2	object	1	3	control	606	275.339
14143	rio	5.73	2	2	2	object	2	1	control	NA	275.339
14144	rio	5.73	2	2	2	object	3	3	control	NA	275.339
14146	rio	5.73	2	2	2	object	5	6	control	462	275.339
14147	rio	5.73	2	2	2	object	6	3	control	NA	275.339
14148	rio	5.73	2	2	2	object	7	5	control	938	275.339
14149	rio	5.73	2	2	2	object	8	5	control	432	275.339
14151	rio	5.73	2	2	2	object	10	4	control	536	275.339
14152	rio	5.73	2	2	2	object	11	4	control	547	275.339
14153	rio	5.73	2	2	2	object	12	3	control	476	275.339
14154	rio	5.73	2	2	2	object	13	6	net	1223	275.339
14156	rio	5.73	2	2	2	object	15	5	control	445	275.339
14157	rio	5.73	2	2	2	object	16	3	net	801	275.339
14158	rio	5.73	2	2	2	object	17	2	control	651	275.339
14159	rio	5.73	2	2	2	object	18	5	control	396	275.339
14161	rio	5.73	2	2	2	object	20	1	control	410	275.339
14163	rio	5.73	2	2	2	object	22	2	control	604	275.339
14164	rio	5.73	2	2	2	object	23	3	control	974	275.339
14166	rio	5.73	2	2	2	object	25	1	control	1922	275.339
14167	rio	5.73	2	2	2	object	26	3	control	421	275.339
14168	rio	5.73	2	2	2	object	27	2	control	423	275.339
14169	rio	5.73	2	2	2	object	28	5	net	522	275.339
14170	rio	5.73	2	2	2	object	29	5	control	450	275.339
14171	rio	5.73	2	2	2	object	30	6	control	380	275.339
14172	rio	5.73	2	2	2	object	31	2	glove	NA	275.339
14173	rio	5.73	2	2	2	object	32	1	control	NA	275.339
14176	rio	5.73	2	2	2	object	35	5	control	474	275.339
14177	rio	5.73	2	2	2	object	36	6	glove	NA	275.339

14178	rio	5.73	2	2	2	object	37	4	control	476	275.339
14179	rio	5.73	2	2	2	object	38	1	glove	626	275.339
14181	rio	5.73	2	2	2	object	40	1	control	801	275.339
14182	rio	5.73	2	2	2	object	41	6	control	506	275.339
14183	rio	5.73	2	2	2	object	42	6	broom	555	275.339
14184	rio	5.73	2	2	2	object	43	1	control	638	275.339
14185	rio	5.73	2	2	2	object	44	5	glove	NA	275.339
14187	rio	5.73	2	2	2	object	46	2	control	507	275.339
14188	rio	5.73	2	2	2	object	47	4	control	686	275.339
14189	rio	5.73	2	2	2	object	48	4	control	527	275.339
14190	rio	5.73	2	2	2	object	49	4	net	759	275.339
14191	rio	5.73	2	2	2	object	50	2	control	837	275.339
14193	rio	5.73	2	2	2	object	52	3	control	455	275.339
14194	rio	5.73	2	2	2	object	53	4	control	433	275.339
14196	rio	5.73	2	2	2	object	55	4	control	317	275.339
14197	rio	5.73	2	2	2	object	56	5	control	411	275.339
14199	rio	5.73	2	2	2	object	58	4	control	445	275.339
14201	rio	5.73	2	2	2	object	60	5	control	NA	275.339
14202	rio	5.73	2	2	2	object	61	3	control	645	275.339
14203	rio	5.73	2	2	2	object	62	1	control	503	275.339
14204	rio	5.73	2	2	2	object	63	1	net	580	275.339
14205	rio	5.73	2	2	2	object	64	1	control	598	275.339
14206	rio	5.73	2	2	2	object	65	2	control	487	275.339
14208	rio	5.73	2	2	2	object	67	3	control	431	275.339
14209	rio	5.73	2	2	2	object	68	3	control	421	275.339
14210	rio	5.73	2	2	2	object	69	1	control	NA	275.339
14213	rio	5.73	2	2	2	object	72	6	control	469	275.339
14214	rio	5.73	2	2	2	object	73	2	net	453	275.339
14215	rio	5.73	2	2	2	object	74	2	control	521	275.339
14216	rio	5.73	2	2	2	object	75	3	control	496	275.339
14218	rio	5.73	2	2	2	object	77	6	control	444	275.339
14220	rio	5.73	2	2	2	object	79	6	control	459	275.339
14221	rio	5.73	2	2	2	object	80	2	control	689	275.339
14223	rio	5.73	2	2	2	object	82	4	control	425	275.339
14226	rio	5.73	2	2	2	object	85	5	control	460	275.339
14227	rio	5.73	2	2	2	object	86	1	broom	623	275.339
14228	rio	5.73	2	2	2	object	87	3	control	487	275.339
14230	rio	5.73	2	2	2	object	89	5	control	348	275.339
14232	rio	5.73	2	2	2	object	91	5	control	458	275.339
14233	rio	5.73	2	2	2	object	92	4	control	434	275.339
14235	rio	5.73	2	2	2	object	94	2	control	451	275.339
14236	rio	5.73	2	2	2	object	95	3	control	391	275.339
14237	rio	5.73	2	2	2	object	96	5	broom	326	275.339
14239	rio	5.73	2	2	2	object	98	2	control	393	275.339
14240	rio	5.73	2	2	2	object	99	6	control	381	275.339
14242	rio	5.73	2	2	2	object	101	1	control	509	275.339
14244	rio	5.73	2	2	2	object	103	6	control	386	275.339
14249	rio	5.73	2	2	2	object	108	6	control	442	275.339
14251	rio	5.73	2	2	2	object	110	1	control	435	275.339
14253	rio	5.73	2	2	2	object	112	6	control	465	275.339

14255	rio	5.73	2	2	2	object	114	3	control	556	275.339
14257	rio	5.73	2	2	2	object	116	4	control	608	275.339
14262	rio	5.73	2	2	2	object	121	5	control	970	275.339
14263	rio	5.73	2	2	2	object	122	3	control	502	275.339
14264	rio	5.73	2	2	2	object	123	2	broom	494	275.339
14265	rio	5.73	2	2	2	object	124	6	control	385	275.339
14267	rio	5.73	2	2	2	object	126	1	control	501	275.339
14268	rio	5.73	2	2	2	object	127	4	broom	668	275.339
14269	rio	5.73	2	2	2	object	128	2	control	469	275.339
14272	rio	5.73	2	2	2	object	131	4	glove	979	275.339
14275	rio	5.73	2	2	2	object	134	2	control	562	275.339
14276	rio	5.73	2	2	2	object	135	1	control	451	275.339
14277	rio	5.73	2	2	2	object	136	3	glove	742	275.339
14278	rio	5.73	2	2	2	object	137	6	control	453	275.339
14281	rio	5.73	2	2	2	object	140	6	control	459	275.339
14282	rio	5.73	2	2	2	object	141	4	control	451	275.339
14283	rio	5.73	2	2	2	object	142	3	broom	552	275.339
14284	rio	5.73	2	2	2	object	143	3	control	434	275.339
14286	rio	5.73	2	2	2	object	145	5	control	394	275.339
14287	rio	5.73	2	2	2	object	146	3	control	501	275.339
14288	rio	5.73	2	2	2	object	147	5	control	404	275.339
14289	rio	5.73	2	2	2	object	148	4	control	393	275.339
14290	rio	5.73	2	2	2	mask	1	3	control	812	275.339
14291	rio	5.73	2	2	2	mask	2	4	control	421	275.339
14292	rio	5.73	2	2	2	mask	3	2	control	479	275.339
14294	rio	5.73	2	2	2	mask	5	4	control	436	275.339
14295	rio	5.73	2	2	2	mask	6	5	control	427	275.339
14296	rio	5.73	2	2	2	mask	7	3	control	484	275.339
14297	rio	5.73	2	2	2	mask	8	4	control	383	275.339
14298	rio	5.73	2	2	2	mask	9	3	control	396	275.339
14299	rio	5.73	2	2	2	mask	10	3	mask_man	2454	275.339
14300	rio	5.73	2	2	2	mask	11	6	control	507	275.339
14301	rio	5.73	2	2	2	mask	12	2	mask_man	666	275.339
14303	rio	5.73	2	2	2	mask	14	2	control	559	275.339
14304	rio	5.73	2	2	2	mask	15	6	control	437	275.339
14306	rio	5.73	2	2	2	mask	17	2	control	397	275.339
14307	rio	5.73	2	2	2	mask	18	1	control	475	275.339
14308	rio	5.73	2	2	2	mask	19	2	control	432	275.339
14309	rio	5.73	2	2	2	mask	20	2	mask_man	467	275.339
14310	rio	5.73	2	2	2	mask	21	5	control	337	275.339
14311	rio	5.73	2	2	2	mask	22	1	mask_man	570	275.339
14312	rio	5.73	2	2	2	mask	23	1	control	456	275.339
14313	rio	5.73	2	2	2	mask	24	1	control	476	275.339
14314	rio	5.73	2	2	2	mask	25	3	mask_man	456	275.339
14315	rio	5.73	2	2	2	mask	26	4	control	385	275.339
14317	rio	5.73	2	2	2	mask	28	4	control	394	275.339
14318	rio	5.73	2	2	2	mask	29	6	control	376	275.339
14319	rio	5.73	2	2	2	mask	30	4	control	440	275.339
14321	rio	5.73	2	2	2	mask	32	3	control	414	275.339
14322	rio	5.73	2	2	2	mask	33	4	mask_man	734	275.339

14324	rio	5.73	2	2	2	mask	35	3	control	526	275.339
14325	rio	5.73	2	2	2	mask	36	5	mask_man	450	275.339
14327	rio	5.73	2	2	2	mask	38	1	control	826	275.339
14328	rio	5.73	2	2	2	mask	39	6	control	501	275.339
14329	rio	5.73	2	2	2	mask	40	1	mask_man	596	275.339
14331	rio	5.73	2	2	2	mask	42	2	control	NA	275.339
14332	rio	5.73	2	2	2	mask	43	6	control	784	275.339
14334	rio	5.73	2	2	2	mask	45	2	control	464	275.339
14335	rio	5.73	2	2	2	mask	46	6	control	344	275.339
14336	rio	5.73	2	2	2	mask	47	1	control	599	275.339
14337	rio	5.73	2	2	2	mask	48	1	mask_man	627	275.339
14339	rio	5.73	2	2	2	mask	50	4	control	476	275.339
14340	rio	5.73	2	2	2	mask	51	1	control	598	275.339
14341	rio	5.73	2	2	2	mask	52	3	control	542	275.339
14343	rio	5.73	2	2	2	mask	54	5	mask_man	485	275.339
14344	rio	5.73	2	2	2	mask	55	4	control	383	275.339
14345	rio	5.73	2	2	2	mask	56	4	control	814	275.339
14346	rio	5.73	2	2	2	mask	57	2	control	770	275.339
14348	rio	5.73	2	2	2	mask	59	4	mask_man	607	275.339
14349	rio	5.73	2	2	2	mask	60	3	control	797	275.339
14350	rio	5.73	2	2	2	mask	61	5	control	389	275.339
14351	rio	5.73	2	2	2	mask	62	3	control	431	275.339
14353	rio	5.73	2	2	2	mask	64	3	control	442	275.339
14356	rio	5.73	2	2	2	mask	67	5	control	NA	275.339
14357	rio	5.73	2	2	2	mask	68	4	control	1429	275.339
14358	rio	5.73	2	2	2	mask	69	6	control	429	275.339
14360	rio	5.73	2	2	2	mask	71	6	control	379	275.339
14362	rio	5.73	2	2	2	mask	73	1	control	475	275.339
14363	rio	5.73	2	2	2	mask	74	5	mask_man	621	275.339
14364	rio	5.73	2	2	2	mask	75	3	control	568	275.339
14367	rio	5.73	2	2	2	mask	78	2	control	NA	275.339
14369	rio	5.73	2	2	2	mask	80	5	control	1184	275.339
14370	rio	5.73	2	2	2	mask	81	6	mask_man	468	275.339
14371	rio	5.73	2	2	2	mask	82	5	control	506	275.339
14373	rio	5.73	2	2	2	mask	84	4	control	484	275.339
14374	rio	5.73	2	2	2	mask	85	4	control	1231	275.339
14376	rio	5.73	2	2	2	mask	87	1	control	450	275.339
14377	rio	5.73	2	2	2	mask	88	2	control	507	275.339
14379	rio	5.73	2	2	2	mask	90	1	control	452	275.339
14381	rio	5.73	2	2	2	mask	92	1	control	448	275.339
14382	rio	5.73	2	2	2	mask	93	3	control	519	275.339
14385	rio	5.73	2	2	2	mask	96	6	control	408	275.339
14386	rio	5.73	2	2	2	mask	97	3	control	420	275.339
14387	rio	5.73	2	2	2	mask	98	5	control	490	275.339
14389	rio	5.73	2	2	2	mask	100	5	control	385	275.339
14390	rio	5.73	2	2	2	mask	101	5	control	381	275.339
14391	rio	5.73	2	2	2	mask	102	2	mask_man	1691	275.339
14393	rio	5.73	2	2	2	mask	104	5	control	459	275.339
14396	rio	5.73	2	2	2	mask	107	4	control	NA	275.339
14397	rio	5.73	2	2	2	mask	108	2	control	942	275.339

14398	rio	5.73	2	2	2	mask	109	6	mask_man	480	275.339
14399	rio	5.73	2	2	2	mask	110	5	control	459	275.339
14402	rio	5.73	2	2	2	mask	113	6	mask_man	1629	275.339
14403	rio	5.73	2	2	2	mask	114	3	control	NA	275.339
14407	rio	5.73	2	2	2	mask	118	2	control	479	275.339
14408	rio	5.73	2	2	2	mask	119	6	control	522	275.339
14411	rio	5.73	2	2	2	mask	122	1	control	435	275.339
14412	rio	5.73	2	2	2	mask	123	2	control	404	275.339
14414	rio	5.73	2	2	2	mask	125	5	control	405	275.339
14416	rio	5.73	2	2	2	mask	127	6	control	389	275.339
14417	rio	5.73	2	2	2	mask	128	1	control	NA	275.339
14420	rio	5.73	2	2	2	mask	131	4	mask_man	534	275.339
14421	rio	5.73	2	2	2	mask	132	5	control	449	275.339
14422	rio	5.73	2	2	2	mask	133	6	control	379	275.339
14423	rio	5.73	2	2	2	mask	134	4	control	447	275.339
14425	rio	5.73	2	2	2	mask	136	2	control	470	275.339
14427	rio	5.73	2	2	2	mask	138	3	mask_man	575	275.339
14428	rio	5.73	2	2	2	mask	139	4	control	446	275.339
14430	rio	5.73	2	2	2	mask	141	3	control	NA	275.339
14433	rio	5.73	2	2	2	mask	144	6	control	339	275.339
14434	rio	5.73	2	2	2	mask	145	6	control	379	275.339
14435	rio	5.73	2	2	2	mask	146	5	control	374	275.339
14436	rod	5.05	5	1	2	mask	1	3	control	557	238.938
14437	rod	5.05	5	1	2	mask	2	3	control	546	238.938
14438	rod	5.05	5	1	2	mask	3	6	control	351	238.938
14440	rod	5.05	5	1	2	mask	5	4	control	370	238.938
14441	rod	5.05	5	1	2	mask	6	2	control	436	238.938
14442	rod	5.05	5	1	2	mask	7	4	control	426	238.938
14443	rod	5.05	5	1	2	mask	8	5	control	481	238.938
14444	rod	5.05	5	1	2	mask	9	1	mask_man	NA	238.938
14445	rod	5.05	5	1	2	mask	10	1	control	625	238.938
14446	rod	5.05	5	1	2	mask	11	5	control	1227	238.938
14447	rod	5.05	5	1	2	mask	12	2	control	491	238.938
14448	rod	5.05	5	1	2	mask	13	6	mask_man	1219	238.938
14449	rod	5.05	5	1	2	mask	14	2	control	808	238.938
14450	rod	5.05	5	1	2	mask	15	2	control	642	238.938
14452	rod	5.05	5	1	2	mask	17	6	control	562	238.938
14453	rod	5.05	5	1	2	mask	18	3	control	491	238.938
14454	rod	5.05	5	1	2	mask	19	2	control	443	238.938
14456	rod	5.05	5	1	2	mask	21	3	control	484	238.938
14457	rod	5.05	5	1	2	mask	22	3	mask_man	1459	238.938
14458	rod	5.05	5	1	2	mask	23	2	control	497	238.938
14459	rod	5.05	5	1	2	mask	24	1	mask_man	1052	238.938
14460	rod	5.05	5	1	2	mask	25	2	control	540	238.938
14462	rod	5.05	5	1	2	mask	27	4	control	633	238.938
14463	rod	5.05	5	1	2	mask	28	1	control	426	238.938
14465	rod	5.05	5	1	2	mask	30	5	control	523	238.938
14466	rod	5.05	5	1	2	mask	31	3	control	486	238.938
14467	rod	5.05	5	1	2	mask	32	3	control	744	238.938
14468	rod	5.05	5	1	2	mask	33	2	mask_man	703	238.938

14469	rod	5.05	5	1	2	mask	34	1	control	623	238.938
14470	rod	5.05	5	1	2	mask	35	4	control	364	238.938
14471	rod	5.05	5	1	2	mask	36	3	mask_man	NA	238.938
14472	rod	5.05	5	1	2	mask	37	1	control	437	238.938
14474	rod	5.05	5	1	2	mask	39	1	control	431	238.938
14476	rod	5.05	5	1	2	mask	41	5	control	458	238.938
14477	rod	5.05	5	1	2	mask	42	2	mask_man	676	238.938
14478	rod	5.05	5	1	2	mask	43	5	control	457	238.938
14479	rod	5.05	5	1	2	mask	44	1	control	416	238.938
14480	rod	5.05	5	1	2	mask	45	3	control	493	238.938
14482	rod	5.05	5	1	2	mask	47	3	control	517	238.938
14483	rod	5.05	5	1	2	mask	48	5	control	422	238.938
14485	rod	5.05	5	1	2	mask	50	6	control	425	238.938
14486	rod	5.05	5	1	2	mask	51	5	control	443	238.938
14487	rod	5.05	5	1	2	mask	52	5	control	555	238.938
14488	rod	5.05	5	1	2	mask	53	3	mask_man	549	238.938
14489	rod	5.05	5	1	2	mask	54	5	control	531	238.938
14491	rod	5.05	5	1	2	mask	56	6	control	483	238.938
14492	rod	5.05	5	1	2	mask	57	6	control	523	238.938
14493	rod	5.05	5	1	2	mask	58	3	control	522	238.938
14494	rod	5.05	5	1	2	mask	59	4	mask_man	445	238.938
14495	rod	5.05	5	1	2	mask	60	6	control	466	238.938
14496	rod	5.05	5	1	2	mask	61	6	control	494	238.938
14497	rod	5.05	5	1	2	mask	62	5	mask_man	525	238.938
14498	rod	5.05	5	1	2	mask	63	4	control	420	238.938
14499	rod	5.05	5	1	2	mask	64	5	mask_man	454	238.938
14500	rod	5.05	5	1	2	mask	65	5	control	404	238.938
14503	rod	5.05	5	1	2	mask	68	3	control	467	238.938
14504	rod	5.05	5	1	2	mask	69	2	mask_man	618	238.938
14505	rod	5.05	5	1	2	mask	70	6	control	363	238.938
14506	rod	5.05	5	1	2	mask	71	2	control	692	238.938
14507	rod	5.05	5	1	2	mask	72	3	control	451	238.938
14509	rod	5.05	5	1	2	mask	74	1	control	652	238.938
14510	rod	5.05	5	1	2	mask	75	3	control	513	238.938
14511	rod	5.05	5	1	2	mask	76	4	control	420	238.938
14513	rod	5.05	5	1	2	mask	78	6	control	430	238.938
14514	rod	5.05	5	1	2	mask	79	6	control	529	238.938
14515	rod	5.05	5	1	2	mask	80	4	control	403	238.938
14516	rod	5.05	5	1	2	mask	81	5	mask_man	654	238.938
14518	rod	5.05	5	1	2	mask	83	2	control	411	238.938
14523	rod	5.05	5	1	2	mask	88	6	control	580	238.938
14524	rod	5.05	5	1	2	mask	89	4	control	526	238.938
14526	rod	5.05	5	1	2	mask	91	4	control	453	238.938
14527	rod	5.05	5	1	2	mask	92	1	control	475	238.938
14529	rod	5.05	5	1	2	mask	94	4	control	595	238.938
14531	rod	5.05	5	1	2	mask	96	5	control	486	238.938
14533	rod	5.05	5	1	2	mask	98	1	control	381	238.938
14534	rod	5.05	5	1	2	mask	99	4	mask_man	469	238.938
14535	rod	5.05	5	1	2	mask	100	2	control	521	238.938
14536	rod	5.05	5	1	2	mask	101	2	control	440	238.938

14537	rod	5.05	5	1	2	mask	102	6	mask_man	528	238.938
14538	rod	5.05	5	1	2	mask	103	1	control	447	238.938
14539	rod	5.05	5	1	2	mask	104	2	control	449	238.938
14540	rod	5.05	5	1	2	mask	105	2	control	444	238.938
14541	rod	5.05	5	1	2	mask	106	1	mask_man	526	238.938
14542	rod	5.05	5	1	2	mask	107	1	control	491	238.938
14543	rod	5.05	5	1	2	mask	108	4	control	378	238.938
14545	rod	5.05	5	1	2	mask	110	5	control	379	238.938
14547	rod	5.05	5	1	2	mask	112	4	control	1252	238.938
14549	rod	5.05	5	1	2	mask	114	4	mask_man	538	238.938
14550	rod	5.05	5	1	2	mask	115	3	control	839	238.938
14551	rod	5.05	5	1	2	mask	116	4	control	455	238.938
14552	rod	5.05	5	1	2	mask	117	6	control	581	238.938
14554	rod	5.05	5	1	2	mask	119	1	control	678	238.938
14555	rod	5.05	5	1	2	mask	120	6	mask_man	888	238.938
14556	rod	5.05	5	1	2	mask	121	3	control	636	238.938
14557	rod	5.05	5	1	2	mask	122	6	control	511	238.938
14559	rod	5.05	5	1	2	mask	124	3	control	545	238.938
14560	rod	5.05	5	1	2	mask	125	4	control	481	238.938
14562	rod	5.05	5	1	2	mask	127	6	control	521	238.938
14563	rod	5.05	5	1	2	mask	128	1	control	424	238.938
14564	rod	5.05	5	1	2	mask	129	2	control	519	238.938
14565	rod	5.05	5	1	2	object	1	5	control	534	238.938
14566	rod	5.05	5	1	2	object	2	2	control	432	238.938
14567	rod	5.05	5	1	2	object	3	6	control	437	238.938
14572	rod	5.05	5	1	2	object	8	5	control	495	238.938
14573	rod	5.05	5	1	2	object	9	3	control	930	238.938
14574	rod	5.05	5	1	2	object	10	4	control	422	238.938
14575	rod	5.05	5	1	2	object	11	4	control	475	238.938
14578	rod	5.05	5	1	2	object	14	2	control	471	238.938
14579	rod	5.05	5	1	2	object	15	4	control	636	238.938
14580	rod	5.05	5	1	2	object	16	4	control	505	238.938
14584	rod	5.05	5	1	2	object	20	2	control	430	238.938
14585	rod	5.05	5	1	2	object	21	2	control	425	238.938
14586	rod	5.05	5	1	2	object	22	1	broom	NA	238.938
14587	rod	5.05	5	1	2	object	23	6	control	648	238.938
14588	rod	5.05	5	1	2	object	24	3	control	550	238.938
14589	rod	5.05	5	1	2	object	25	1	control	467	238.938
14591	rod	5.05	5	1	2	object	27	3	broom	536	238.938
14592	rod	5.05	5	1	2	object	28	5	control	425	238.938
14593	rod	5.05	5	1	2	object	29	5	control	488	238.938
14598	rod	5.05	5	1	2	object	34	4	broom	563	238.938
14604	rod	5.05	5	1	2	object	40	4	control	444	238.938
14606	rod	5.05	5	1	2	object	42	2	control	473	238.938
14608	rod	5.05	5	1	2	object	44	4	control	427	238.938
14612	rod	5.05	5	1	2	object	48	3	control	472	238.938
14613	rod	5.05	5	1	2	object	49	2	glove	618	238.938
14614	rod	5.05	5	1	2	object	50	1	control	568	238.938
14615	rod	5.05	5	1	2	object	51	1	control	431	238.938
14616	rod	5.05	5	1	2	object	52	2	control	435	238.938

14618	rod	5.05	5	1	2	object	54	3	control	560	238.938
14619	rod	5.05	5	1	2	object	55	4	control	435	238.938
14620	rod	5.05	5	1	2	object	56	6	control	1179	238.938
14622	rod	5.05	5	1	2	object	58	2	control	520	238.938
14625	rod	5.05	5	1	2	object	61	4	glove	333	238.938
14626	rod	5.05	5	1	2	object	62	2	control	463	238.938
14628	rod	5.05	5	1	2	object	64	1	control	392	238.938
14629	rod	5.05	5	1	2	object	65	3	control	508	238.938
14630	rod	5.05	5	1	2	object	66	5	broom	561	238.938
14631	rod	5.05	5	1	2	object	67	3	control	411	238.938
14632	rod	5.05	5	1	2	object	68	1	control	452	238.938
14636	rod	5.05	5	1	2	object	72	1	control	513	238.938
14638	rod	5.05	5	1	2	object	74	6	control	456	238.938
14639	rod	5.05	5	1	2	object	75	1	control	521	238.938
14640	rod	5.05	5	1	2	object	76	5	control	433	238.938
14641	rod	5.05	5	1	2	object	77	2	broom	1492	238.938
14642	rod	5.05	5	1	2	object	78	2	control	467	238.938
14643	rod	5.05	5	1	2	object	79	2	control	406	238.938
14645	rod	5.05	5	1	2	object	81	5	control	444	238.938
14646	rod	5.05	5	1	2	object	82	4	control	544	238.938
14647	rod	5.05	5	1	2	object	83	6	control	NA	238.938
14648	rod	5.05	5	1	2	object	84	1	net	NA	238.938
14649	rod	5.05	5	1	2	object	85	2	control	545	238.938
14650	rod	5.05	5	1	2	object	86	6	control	532	238.938
14651	rod	5.05	5	1	2	object	87	2	control	485	238.938
14653	rod	5.05	5	1	2	object	89	3	control	514	238.938
14654	rod	5.05	5	1	2	object	90	1	glove	500	238.938
14655	rod	5.05	5	1	2	object	91	5	control	747	238.938
14656	rod	5.05	5	1	2	object	92	5	glove	418	238.938
14657	rod	5.05	5	1	2	object	93	6	control	355	238.938
14658	rod	5.05	5	1	2	object	94	5	control	386	238.938
14661	rod	5.05	5	1	2	object	97	1	control	484	238.938
14662	rod	5.05	5	1	2	object	98	3	net	1129	238.938
14663	rod	5.05	5	1	2	object	99	3	control	411	238.938
14664	rod	5.05	5	1	2	object	100	2	net	767	238.938
14665	rod	5.05	5	1	2	object	101	5	control	1070	238.938
14667	rod	5.05	5	1	2	object	103	1	control	489	238.938
14668	rod	5.05	5	1	2	object	104	1	control	379	238.938
14670	rod	5.05	5	1	2	object	106	4	control	447	238.938
14671	rod	5.05	5	1	2	object	107	6	glove	920	238.938
14672	rod	5.05	5	1	2	object	108	3	control	NA	238.938
14674	rod	5.05	5	1	2	object	110	6	control	1838	238.938
14675	rod	5.05	5	1	2	object	111	3	control	456	238.938
14676	rod	5.05	5	1	2	object	112	3	glove	728	238.938
14677	rod	5.05	5	1	2	object	113	5	control	434	238.938
14678	rod	5.05	5	1	2	object	114	4	control	439	238.938
14679	rod	5.05	5	1	2	object	115	3	control	455	238.938
14681	rod	5.05	5	1	2	object	117	4	control	511	238.938
14682	rod	5.05	5	1	2	object	118	6	control	471	238.938
14683	rod	5.05	5	1	2	object	119	6	control	467	238.938

14684	rod	5.05	5	1	2	object	120	5	net	481	238.938
14685	rod	5.05	5	1	2	object	121	6	control	368	238.938
14686	rod	5.05	5	1	2	object	122	6	control	679	238.938
14688	rod	5.05	5	1	2	object	124	6	broom	828	238.938
14693	rod	5.05	5	1	2	object	129	5	control	322	238.938
14694	rod	5.05	5	1	2	object	130	5	control	1147	238.938
14695	rod	5.05	5	1	2	object	131	4	control	479	238.938
14696	rod	5.05	5	1	2	object	132	6	net	374	238.938
14697	rod	5.05	5	1	2	object	133	5	control	778	238.938
14699	rod	5.05	5	1	2	object	135	2	control	577	238.938
14701	rod	5.05	5	1	2	object	137	5	control	363	238.938
14702	rod	5.05	5	1	2	object	138	6	control	474	238.938
14703	rod	5.05	5	1	2	object	139	3	control	419	238.938
14705	rod	5.05	5	1	2	object	141	4	net	604	238.938
14706	rod	5.05	5	1	2	object	142	1	control	NA	238.938
14708	rod	5.05	5	1	2	object	144	3	control	524	238.938
14709	rod	5.05	5	1	2	object	145	1	control	410	238.938
14711	rod	5.05	5	1	2	object	147	3	control	435	238.938
14713	rod	5.05	5	1	2	object	149	3	control	503	238.938
14715	rod	5.05	5	1	2	object	151	4	control	1055	238.938
14731	rod	5.05	5	1	2	object	167	5	control	345	238.938
14732	rod	5.05	5	1	2	object	168	4	control	429	238.938
14733	zaz	6.18	1	1	2	mask	1	2	control	628	242.811
14734	zaz	6.18	1	1	2	mask	2	1	control	604	242.811
14735	zaz	6.18	1	1	2	mask	3	1	control	605	242.811
14737	zaz	6.18	1	1	2	mask	5	2	control	NA	242.811
14739	zaz	6.18	1	1	2	mask	7	4	control	601	242.811
14740	zaz	6.18	1	1	2	mask	8	2	control	528	242.811
14741	zaz	6.18	1	1	2	mask	9	6	control	690	242.811
14742	zaz	6.18	1	1	2	mask	10	4	control	526	242.811
14747	zaz	6.18	1	1	2	mask	15	1	control	563	242.811
14748	zaz	6.18	1	1	2	mask	16	2	control	618	242.811
14749	zaz	6.18	1	1	2	mask	17	1	mask_man	2878	242.811
14750	zaz	6.18	1	1	2	mask	18	3	control	NA	242.811
14751	zaz	6.18	1	1	2	mask	19	5	control	NA	242.811
14754	zaz	6.18	1	1	2	mask	22	3	control	2141	242.811
14755	zaz	6.18	1	1	2	mask	23	5	control	607	242.811
14756	zaz	6.18	1	1	2	mask	24	6	control	598	242.811
14758	zaz	6.18	1	1	2	mask	26	6	control	2861	242.811
14759	zaz	6.18	1	1	2	mask	27	2	control	531	242.811
14761	zaz	6.18	1	1	2	mask	29	5	control	605	242.811
14762	zaz	6.18	1	1	2	mask	30	6	control	643	242.811
14763	zaz	6.18	1	1	2	mask	31	4	mask_man	1446	242.811
14764	zaz	6.18	1	1	2	mask	32	4	control	587	242.811
14765	zaz	6.18	1	1	2	mask	33	2	control	897	242.811
14766	zaz	6.18	1	1	2	mask	34	3	control	603	242.811
14767	zaz	6.18	1	1	2	mask	35	5	mask_man	856	242.811
14768	zaz	6.18	1	1	2	mask	36	1	control	NA	242.811
14770	zaz	6.18	1	1	2	mask	38	1	control	509	242.811
14771	zaz	6.18	1	1	2	mask	39	5	control	629	242.811

14773	zaz	6.18	1	1	2	mask	41	1	control	2310		242.811
14774	zaz	6.18	1	1	2	mask	42	4	control	532		242.811
14775	zaz	6.18	1	1	2	mask	43	2	control	1521		242.811
14776	zaz	6.18	1	1	2	mask	44	1	mask_man	864		242.811
14779	zaz	6.18	1	1	2	mask	47	1	control	1067		242.811
14780	zaz	6.18	1	1	2	mask	48	4	control	534		242.811
14781	zaz	6.18	1	1	2	mask	49	3	control	619		242.811
14782	zaz	6.18	1	1	2	mask	50	4	mask_man	697		242.811
14786	zaz	6.18	1	1	2	mask	54	5	control	NA		242.811
14787	zaz	6.18	1	1	2	mask	55	2	control	525		242.811
14788	zaz	6.18	1	1	2	mask	56	3	control	1412		242.811
14794	zaz	6.18	1	1	2	mask	62	6	control	562		242.811
14795	zaz	6.18	1	1	2	mask	63	2	control	NA		242.811
14797	zaz	6.18	1	1	2	mask	65	5	control	NA		242.811
14800	zaz	6.18	1	1	2	mask	68	4	control	1237		242.811
14805	zaz	6.18	1	1	2	mask	73	3	control	601		242.811
14806	zaz	6.18	1	1	2	mask	74	3	control	NA		242.811
14807	zaz	6.18	1	1	2	mask	75	3	mask_man	1331		242.811
14816	zaz	6.18	1	1	2	mask	84	6	control	1550		242.811
14817	zaz	6.18	1	1	2	mask	85	1	control	588		242.811
14821	zaz	6.18	1	1	2	mask	89	3	control	NA		242.811
14822	zaz	6.18	1	1	2	mask	90	4	mask_man	731		242.811
14828	zaz	6.18	1	1	2	mask	96	2	control	1305		242.811
14829	zaz	6.18	1	1	2	mask	97	5	control	1697		242.811
14830	zaz	6.18	1	1	2	mask	98	2	mask_man	688		242.811
14839	zaz	6.18	1	1	2	mask	107	5	control	1613		242.811
14840	zaz	6.18	1	1	2	mask	108	4	control	531		242.811
14841	zaz	6.18	1	1	2	mask	109	4	control	1130		242.811
14842	zaz	6.18	1	1	2	mask	110	3	mask_man	973		242.811
14868	zaz	6.18	1	1	2	mask	136	3	control	1035		242.811
14869	zaz	6.18	1	1	2	mask	137	5	control	NA		242.811
14875	zaz	6.18	1	1	2	mask	143	2	control	NA		242.811
14883	zaz	6.18	1	1	2	mask	151	6	mask_man	1104		242.811
14884	zaz	6.18	1	1	2	mask	152	3	control	NA		242.811
14889	zaz	6.18	1	1	2	mask	157	1	mask_man	716		242.811
14902	zaz	6.18	1	1	2	mask	170	5	control	NA		242.811
14908	zaz	6.18	1	1	2	mask	176	1	control	1879		242.811
14909	zaz	6.18	1	1	2	mask	177	6	control	NA		242.811
14911	zaz	6.18	1	1	2	mask	179	6	control	2279		242.811
14912	zaz	6.18	1	1	2	mask	180	3	control	1750		242.811
14913	zaz	6.18	1	1	2	mask	181	6	control	887		242.811
14915	zaz	6.18	1	1	2	mask	183	5	control	NA		242.811
14919	zaz	6.18	1	1	2	mask	187	2	control	808		242.811
14920	zaz	6.18	1	1	2	mask	188	1	control	649		242.811
14921	zaz	6.18	1	1	2	mask	189	2	mask_man	1109		242.811
14922	zaz	6.18	1	1	2	mask	190	6	control	NA		242.811
14925	zaz	6.18	1	1	2	mask	193	4	control	NA		242.811
14926	zaz	6.18	1	1	2	mask	194	2	control	1005		242.811
14928	zaz	6.18	1	1	2	mask	196	6	control	2625		242.811
14930	zaz	6.18	1	1	2	mask	198	3	control	1668		242.811

14932	zaz	6.18	1	1	2	mask	200	1	control	1147	242.811
14933	zaz	6.18	1	1	2	mask	201	3	mask_man	1142	242.811
14940	zaz	6.18	1	1	2	mask	208	1	control	633	242.811
14941	zaz	6.18	1	1	2	mask	209	6	mask_man	868	242.811
14945	zaz	6.18	1	1	2	mask	213	4	control	627	242.811
14946	zaz	6.18	1	1	2	mask	214	4	control	717	242.811
14947	zaz	6.18	1	1	2	mask	215	5	mask_man	793	242.811
14956	zaz	6.18	1	1	2	mask	224	6	control	569	242.811
14958	zaz	6.18	1	1	2	mask	226	4	control	1149	242.811
14959	zaz	6.18	1	1	2	mask	227	2	mask_man	928	242.811
14967	zaz	6.18	1	1	2	mask	235	1	control	764	242.811
14969	zaz	6.18	1	1	2	mask	237	2	control	534	242.811
14971	zaz	6.18	1	1	2	mask	239	5	control	NA	242.811
14978	zaz	6.18	1	1	2	mask	246	1	control	NA	242.811
14980	zaz	6.18	1	1	2	mask	248	6	mask_man	727	242.811
14981	zaz	6.18	1	1	2	mask	249	4	control	NA	242.811
14984	zaz	6.18	1	1	2	mask	252	5	mask_man	746	242.811
14988	zaz	6.18	1	1	2	mask	256	1	control	NA	242.811
14990	zaz	6.18	1	1	2	mask	258	1	control	695	242.811
14992	zaz	6.18	1	1	2	mask	260	5	control	497	242.811
14993	zaz	6.18	1	1	2	mask	261	3	control	1228	242.811
14994	zaz	6.18	1	1	2	mask	262	3	control	1489	242.811
14995	zaz	6.18	1	1	2	object	1	2	control	540	242.811
14996	zaz	6.18	1	1	2	object	2	6	control	NA	242.811
15003	zaz	6.18	1	1	2	object	9	2	control	426	242.811
15004	zaz	6.18	1	1	2	object	10	5	control	NA	242.811
15013	zaz	6.18	1	1	2	object	19	3	control	593	242.811
15014	zaz	6.18	1	1	2	object	20	5	control	531	242.811
15015	zaz	6.18	1	1	2	object	21	3	control	580	242.811
15016	zaz	6.18	1	1	2	object	22	1	control	752	242.811
15017	zaz	6.18	1	1	2	object	23	2	broom	NA	242.811
15020	zaz	6.18	1	1	2	object	26	6	control	NA	242.811
15021	zaz	6.18	1	1	2	object	27	6	control	1098	242.811
15022	zaz	6.18	1	1	2	object	28	6	broom	NA	242.811
15025	zaz	6.18	1	1	2	object	31	1	control	987	242.811
15027	zaz	6.18	1	1	2	object	33	6	control	493	242.811
15028	zaz	6.18	1	1	2	object	34	4	glove	1414	242.811
15029	zaz	6.18	1	1	2	object	35	3	control	573	242.811
15031	zaz	6.18	1	1	2	object	37	4	control	720	242.811
15032	zaz	6.18	1	1	2	object	38	4	control	506	242.811
15034	zaz	6.18	1	1	2	object	40	4	control	461	242.811
15035	zaz	6.18	1	1	2	object	41	3	broom	NA	242.811
15037	zaz	6.18	1	1	2	object	43	3	control	NA	242.811
15038	zaz	6.18	1	1	2	object	44	1	control	1837	242.811
15039	zaz	6.18	1	1	2	object	45	4	control	511	242.811
15040	zaz	6.18	1	1	2	object	46	1	net	2462	242.811
15041	zaz	6.18	1	1	2	object	47	6	control	556	242.811
15042	zaz	6.18	1	1	2	object	48	6	control	541	242.811
15043	zaz	6.18	1	1	2	object	49	2	control	504	242.811
15046	zaz	6.18	1	1	2	object	52	2	control	1266	242.811

15047	zaz	6.18	1	1	2	object	53	4	control	544	242.811
15048	zaz	6.18	1	1	2	object	54	1	control	754	242.811
15051	zaz	6.18	1	1	2	object	57	5	control	927	242.811
15052	zaz	6.18	1	1	2	object	58	2	control	562	242.811
15057	zaz	6.18	1	1	2	object	63	1	control	1101	242.811
15058	zaz	6.18	1	1	2	object	64	4	control	768	242.811
15060	zaz	6.18	1	1	2	object	66	5	control	556	242.811
15061	zaz	6.18	1	1	2	object	67	6	glove	NA	242.811
15065	zaz	6.18	1	1	2	object	71	1	control	562	242.811
15066	zaz	6.18	1	1	2	object	72	1	glove	NA	242.811
15073	zaz	6.18	1	1	2	object	79	5	control	2403	242.811
15075	zaz	6.18	1	1	2	object	81	1	control	742	242.811
15076	zaz	6.18	1	1	2	object	82	2	net	951	242.811
15077	zaz	6.18	1	1	2	object	83	5	control	NA	242.811
15078	zaz	6.18	1	1	2	object	84	2	control	2806	242.811
15079	zaz	6.18	1	1	2	object	85	4	control	820	242.811
15083	zaz	6.18	1	1	2	object	89	1	control	1207	242.811
15097	zaz	6.18	1	1	2	object	103	4	control	NA	242.811
15098	zaz	6.18	1	1	2	object	104	6	control	1819	242.811
15099	zaz	6.18	1	1	2	object	105	5	control	549	242.811
15100	zaz	6.18	1	1	2	object	106	4	net	880	242.811
15110	zaz	6.18	1	1	2	object	116	3	control	NA	242.811
15111	zaz	6.18	1	1	2	object	117	1	control	NA	242.811
15122	zaz	6.18	1	1	2	object	128	4	control	681	242.811
15123	zaz	6.18	1	1	2	object	129	5	glove	NA	242.811
15137	zaz	6.18	1	1	2	object	143	2	control	2009	242.811
15138	zaz	6.18	1	1	2	object	144	3	control	2598	242.811
15140	zaz	6.18	1	1	2	object	146	1	control	NA	242.811
15141	zaz	6.18	1	1	2	object	147	5	control	2272	242.811
15142	zaz	6.18	1	1	2	object	148	3	control	NA	242.811
15143	zaz	6.18	1	1	2	object	149	6	net	933	242.811
15144	zaz	6.18	1	1	2	object	150	1	control	809	242.811
15149	zaz	6.18	1	1	2	object	155	3	control	NA	242.811
15150	zaz	6.18	1	1	2	object	156	2	control	NA	242.811
15178	zaz	6.18	1	1	2	object	184	5	control	2852	242.811
15179	zaz	6.18	1	1	2	object	185	5	control	565	242.811
15183	zaz	6.18	1	1	2	object	189	4	control	1213	242.811
15184	zaz	6.18	1	1	2	object	190	2	control	1418	242.811
15185	zaz	6.18	1	1	2	object	191	5	broom	NA	242.811
15188	zaz	6.18	1	1	2	object	194	1	control	1219	242.811
15189	zaz	6.18	1	1	2	object	195	3	control	2179	242.811
15190	zaz	6.18	1	1	2	object	196	6	control	2138	242.811
15198	zaz	6.18	1	1	2	object	204	6	control	2078	242.811
15199	zaz	6.18	1	1	2	object	205	4	control	798	242.811
15200	zaz	6.18	1	1	2	object	206	6	control	2473	242.811
15202	zaz	6.18	1	1	2	object	208	2	control	1010	242.811
15203	zaz	6.18	1	1	2	object	209	2	control	608	242.811
15204	zaz	6.18	1	1	2	object	210	4	broom	NA	242.811
15207	zaz	6.18	1	1	2	object	213	6	control	1350	242.811
15209	zaz	6.18	1	1	2	object	215	2	control	NA	242.811

15210	zaz	6.18	1	1	2	object	216	6	control	2183	242.811
15211	zaz	6.18	1	1	2	object	217	6	control	NA	242.811
15212	zaz	6.18	1	1	2	object	218	5	net	1370	242.811
15213	zaz	6.18	1	1	2	object	219	2	control	NA	242.811
15214	zaz	6.18	1	1	2	object	220	5	control	2950	242.811
15215	zaz	6.18	1	1	2	object	221	2	glove	NA	242.811
15217	zaz	6.18	1	1	2	object	223	3	control	1641	242.811
15218	zaz	6.18	1	1	2	object	224	4	control	1433	242.811
15223	zaz	6.18	1	1	2	object	229	6	control	1697	242.811
15224	zaz	6.18	1	1	2	object	230	3	control	NA	242.811
15225	zaz	6.18	1	1	2	object	231	4	control	596	242.811
15500	hum	11.95	6	2	2	object	275	3	control	737	257.6
15501	hum	11.95	6	2	2	object	276	3	control	567	257.6
15502	hum	11.95	6	2	2	object	277	2	control	517	257.6
15503	hum	11.95	6	2	2	object	278	1	control	520	257.6
15505	hum	11.95	6	2	2	object	280	3	control	503	257.6
15553	hum	11.95	6	2	2	object	328	5	control	767	257.6
15554	hum	11.95	6	2	2	object	329	1	control	660	257.6
15555	hum	11.95	6	2	2	object	330	2	control	554	257.6
15557	hum	11.95	6	2	2	object	332	5	control	507	257.6
15558	hum	11.95	6	2	2	object	333	1	glove	613	257.6
15559	hum	11.95	6	2	2	object	334	3	control	488	257.6
15560	hum	11.95	6	2	2	object	335	3	control	475	257.6
15562	hum	11.95	6	2	2	object	337	1	control	519	257.6
15563	hum	11.95	6	2	2	object	338	1	control	465	257.6
15564	hum	11.95	6	2	2	object	339	2	control	545	257.6
15566	hum	11.95	6	2	2	object	341	1	control	454	257.6
15568	hum	11.95	6	2	2	object	343	3	control	473	257.6
15569	hum	11.95	6	2	2	object	344	4	control	449	257.6
15570	hum	11.95	6	2	2	object	345	1	control	444	257.6
15611	hum	11.95	6	2	2	object	386	6	control	570	257.6
15612	hum	11.95	6	2	2	object	387	5	control	444	257.6
15613	hum	11.95	6	2	2	object	388	1	broom	537	257.6
15614	hum	11.95	6	2	2	object	389	1	control	504	257.6
15657	hum	11.95	6	2	2	object	432	3	glove	703	257.6
15658	hum	11.95	6	2	2	object	433	1	control	537	257.6
15659	hum	11.95	6	2	2	object	434	2	net	851	257.6
15660	hum	11.95	6	2	2	object	435	3	control	470	257.6
15661	hum	11.95	6	2	2	object	436	4	control	481	257.6
15662	hum	11.95	6	2	2	object	437	6	broom	490	257.6
15663	hum	11.95	6	2	2	object	438	6	control	420	257.6
15664	hum	11.95	6	2	2	object	439	3	control	423	257.6
15710	hum	11.95	6	2	2	object	485	6	control	535	257.6
15711	hum	11.95	6	2	2	object	486	5	control	471	257.6
15712	hum	11.95	6	2	2	object	487	3	control	499	257.6
15713	hum	11.95	6	2	2	object	488	4	net	552	257.6
15714	hum	11.95	6	2	2	object	489	6	control	481	257.6
15715	hum	11.95	6	2	2	object	490	3	control	551	257.6
15716	hum	11.95	6	2	2	object	491	6	control	436	257.6
15717	hum	11.95	6	2	2	object	492	1	net	552	257.6

15718	hum	11.95	6	2	2	object	493	1	control	499	257.6
15720	hum	11.95	6	2	2	object	495	2	control	444	257.6
15722	hum	11.95	6	2	2	object	497	3	control	434	257.6
15723	hum	11.95	6	2	2	object	498	4	control	437	257.6
15724	hum	11.95	6	2	2	object	499	4	control	449	257.6
15725	hum	11.95	6	2	2	object	500	6	net	405	257.6
15726	hum	11.95	6	2	2	object	501	4	control	480	257.6
15727	hum	11.95	6	2	2	object	502	3	control	443	257.6
15729	hum	11.95	6	2	2	object	504	2	control	431	257.6
15730	hum	11.95	6	2	2	object	505	3	control	526	257.6
15731	hum	11.95	6	2	2	object	506	5	broom	415	257.6
15732	hum	11.95	6	2	2	object	507	2	control	570	257.6
15733	hum	11.95	6	2	2	object	508	6	glove	420	257.6
15734	hum	11.95	6	2	2	object	509	6	control	396	257.6
15735	hum	11.95	6	2	2	object	510	6	control	537	257.6
15736	hum	11.95	6	2	2	object	511	4	control	439	257.6
15738	hum	11.95	6	2	2	object	513	5	control	428	257.6
15740	hum	11.95	6	2	2	object	515	1	control	484	257.6
15741	hum	11.95	6	2	2	object	516	2	control	469	257.6
15742	hum	11.95	6	2	2	object	517	4	control	465	257.6
15744	hum	11.95	6	2	2	object	519	2	control	460	257.6
16153	hum	11.95	6	2	2	object	928	2	control	571	257.6
16155	hum	11.95	6	2	2	object	930	6	control	500	257.6
16156	hum	11.95	6	2	2	object	931	5	control	456	257.6
16157	hum	11.95	6	2	2	object	932	6	control	444	257.6
16158	hum	11.95	6	2	2	object	933	6	glove	549	257.6
16159	hum	11.95	6	2	2	object	934	1	control	618	257.6
16160	hum	11.95	6	2	2	object	935	1	control	682	257.6
16161	hum	11.95	6	2	2	object	936	4	broom	620	257.6
16162	hum	11.95	6	2	2	object	937	4	control	528	257.6
16163	hum	11.95	6	2	2	object	938	1	control	567	257.6
16164	hum	11.95	6	2	2	object	939	5	broom	447	257.6
16165	hum	11.95	6	2	2	object	940	3	control	552	257.6
16166	hum	11.95	6	2	2	object	941	5	control	518	257.6
16167	hum	11.95	6	2	2	object	942	2	control	473	257.6
16169	hum	11.95	6	2	2	object	944	4	control	495	257.6
16170	hum	11.95	6	2	2	object	945	5	glove	543	257.6
16171	hum	11.95	6	2	2	object	946	3	control	523	257.6
16172	hum	11.95	6	2	2	object	947	5	control	469	257.6
16173	hum	11.95	6	2	2	object	948	3	control	565	257.6
16174	hum	11.95	6	2	2	object	949	3	broom	510	257.6
16175	hum	11.95	6	2	2	object	950	4	control	533	257.6
16176	hum	11.95	6	2	2	object	951	6	control	458	257.6
16178	hum	11.95	6	2	2	object	953	4	control	444	257.6
16179	hum	11.95	6	2	2	object	954	5	control	481	257.6
16180	hum	11.95	6	2	2	object	955	1	control	504	257.6
16182	hum	11.95	6	2	2	object	957	4	control	507	257.6
16184	hum	11.95	6	2	2	object	959	1	control	562	257.6
16185	hum	11.95	6	2	2	object	960	2	control	471	257.6
16186	hum	11.95	6	2	2	object	961	6	broom	417	257.6

16187	hum	11.95	6	2	2	object	962	4	control	447	257.6
16189	hum	11.95	6	2	2	object	964	2	control	500	257.6
16190	hum	11.95	6	2	2	object	965	2	control	474	257.6
16191	hum	11.95	6	2	2	object	966	2	control	476	257.6
16193	hum	11.95	6	2	2	object	968	3	control	427	257.6
16255	hum	11.95	6	2	2	object	1030	4	control	999	257.6
16256	hum	11.95	6	2	2	object	1031	2	glove	642	257.6
16257	hum	11.95	6	2	2	object	1032	3	control	560	257.6
16258	hum	11.95	6	2	2	object	1033	5	control	492	257.6
16259	hum	11.95	6	2	2	object	1034	3	glove	573	257.6
16260	hum	11.95	6	2	2	object	1035	5	control	457	257.6
16261	hum	11.95	6	2	2	object	1036	2	control	495	257.6
16263	hum	11.95	6	2	2	object	1038	1	control	475	257.6
16264	hum	11.95	6	2	2	object	1039	5	control	462	257.6
16266	hum	11.95	6	2	2	object	1041	2	control	434	257.6
16267	hum	11.95	6	2	2	object	1042	3	control	565	257.6
16268	hum	11.95	6	2	2	object	1043	2	control	482	257.6
16269	hum	11.95	6	2	2	object	1044	5	net	512	257.6
16270	hum	11.95	6	2	2	object	1045	2	control	557	257.6
16271	hum	11.95	6	2	2	object	1046	5	control	422	257.6
16272	hum	11.95	6	2	2	object	1047	6	control	447	257.6
16274	hum	11.95	6	2	2	object	1049	6	control	460	257.6
16275	hum	11.95	6	2	2	object	1050	3	control	456	257.6
16811	hum	11.95	6	2	2	object	463	6	control	800	257.6
16812	hum	11.95	6	2	2	object	464	2	control	632	257.6
16814	hum	11.95	6	2	2	object	466	5	control	528	257.6
16815	hum	11.95	6	2	2	object	467	5	control	595	257.6
16816	hum	11.95	6	2	2	object	468	1	control	618	257.6
16818	hum	11.95	6	2	2	object	470	3	control	603	257.6
16820	hum	11.95	6	2	2	object	472	1	control	548	257.6
16821	hum	11.95	6	2	2	object	473	4	control	502	257.6
16822	hum	11.95	6	2	2	object	474	2	glove	608	257.6
16823	hum	11.95	6	2	2	object	475	5	control	475	257.6
16824	hum	11.95	6	2	2	object	476	2	control	518	257.6
16825	hum	11.95	6	2	2	object	477	1	control	503	257.6
16827	hum	11.95	6	2	2	object	479	2	control	466	257.6
16828	hum	11.95	6	2	2	object	480	4	glove	469	257.6
16829	hum	11.95	6	2	2	object	481	5	control	455	257.6
16830	hum	11.95	6	2	2	object	482	6	control	445	257.6
16832	hum	11.95	6	2	2	object	484	4	control	465	257.6
16833	hum	11.95	6	2	2	object	485	1	control	480	257.6
16835	hum	11.95	6	2	2	object	487	2	control	826	257.6
16837	hum	11.95	6	2	2	object	489	5	control	400	257.6
16851	hum	11.95	6	2	2	object	503	6	control	524	257.6
16852	hum	11.95	6	2	2	object	504	2	control	542	257.6
16854	hum	11.95	6	2	2	object	506	6	control	459	257.6
16855	hum	11.95	6	2	2	object	507	4	control	479	257.6
16856	hum	11.95	6	2	2	object	508	3	control	482	257.6
16857	hum	11.95	6	2	2	object	509	4	net	508	257.6
16858	hum	11.95	6	2	2	object	510	5	control	507	257.6

16859	hum	11.95	6	2	2	object	511	2	control	525	257.6
16860	hum	11.95	6	2	2	object	512	4	control	455	257.6
16862	hum	11.95	6	2	2	object	514	6	control	405	257.6
16863	hum	11.95	6	2	2	object	515	3	net	512	257.6
16864	hum	11.95	6	2	2	object	516	2	control	584	257.6
16865	hum	11.95	6	2	2	object	517	4	control	472	257.6
16866	hum	11.95	6	2	2	object	518	3	glove	443	257.6
16867	hum	11.95	6	2	2	object	519	3	control	443	257.6
16868	hum	11.95	6	2	2	object	520	4	control	449	257.6
16869	hum	11.95	6	2	2	object	521	2	control	440	257.6
16870	hum	11.95	6	2	2	object	522	6	net	442	257.6
16871	hum	11.95	6	2	2	object	523	4	control	531	257.6
16872	hum	11.95	6	2	2	object	524	1	control	516	257.6
16873	hum	11.95	6	2	2	object	525	4	broom	512	257.6
16874	hum	11.95	6	2	2	object	526	2	control	521	257.6
16875	hum	11.95	6	2	2	object	527	5	glove	430	257.6
16876	hum	11.95	6	2	2	object	528	6	control	434	257.6
16877	hum	11.95	6	2	2	object	529	2	net	481	257.6
16878	hum	11.95	6	2	2	object	530	4	control	441	257.6
16880	hum	11.95	6	2	2	object	532	4	control	459	257.6
16881	hum	11.95	6	2	2	object	533	3	control	598	257.6
16883	hum	11.95	6	2	2	object	535	1	control	493	257.6
16884	hum	11.95	6	2	2	object	536	5	control	451	257.6
16885	hum	11.95	6	2	2	object	537	2	control	474	257.6
16887	hum	11.95	6	2	2	object	539	6	control	461	257.6
16888	hum	11.95	6	2	2	object	540	1	glove	537	257.6
16889	hum	11.95	6	2	2	object	541	6	control	447	257.6
16890	hum	11.95	6	2	2	object	542	4	control	467	257.6
16891	hum	11.95	6	2	2	object	543	1	broom	460	257.6
16892	hum	11.95	6	2	2	object	544	1	control	514	257.6
16950	hum	11.95	6	2	2	object	602	4	control	888	257.6
16951	hum	11.95	6	2	2	object	603	1	control	690	257.6
16953	hum	11.95	6	2	2	object	605	3	control	481	257.6
16955	hum	11.95	6	2	2	object	607	3	control	455	257.6
16956	hum	11.95	6	2	2	object	608	3	control	425	257.6
16957	hum	11.95	6	2	2	object	609	1	control	527	257.6
16959	hum	11.95	6	2	2	object	611	1	control	602	257.6
16960	hum	11.95	6	2	2	object	612	2	control	482	257.6
16961	hum	11.95	6	2	2	object	613	1	control	520	257.6
16962	hum	11.95	6	2	2	object	614	5	net	525	257.6
16963	hum	11.95	6	2	2	object	615	1	control	514	257.6
16964	hum	11.95	6	2	2	object	616	6	glove	466	257.6
16965	hum	11.95	6	2	2	object	617	4	control	457	257.6
16966	hum	11.95	6	2	2	object	618	6	control	443	257.6
16967	hum	11.95	6	2	2	object	619	6	broom	468	257.6
16968	hum	11.95	6	2	2	object	620	3	control	439	257.6
16969	hum	11.95	6	2	2	object	621	3	control	450	257.6
16970	hum	11.95	6	2	2	object	622	5	broom	454	257.6
16971	hum	11.95	6	2	2	object	623	5	control	444	257.6
16972	hum	11.95	6	2	2	object	624	3	control	532	257.6

16973	hum	11.95	6	2	2	object	625	6	control	379	257.6
16974	hum	11.95	6	2	2	object	626	3	broom	551	257.6
16975	hum	11.95	6	2	2	object	627	5	control	419	257.6
16976	hum	11.95	6	2	2	object	628	2	control	431	257.6
16977	hum	11.95	6	2	2	object	629	2	control	461	257.6
16978	hum	11.95	6	2	2	object	630	2	broom	525	257.6
16979	hum	11.95	6	2	2	object	631	5	control	435	257.6
16980	hum	11.95	6	2	2	object	632	5	control	436	257.6
16981	hum	11.95	6	2	2	object	633	6	control	416	257.6
16983	hum	11.95	6	2	2	object	635	3	control	498	257.6
16984	hum	11.95	6	2	2	object	636	6	control	406	257.6
16985	hum	11.95	6	2	2	object	637	6	control	501	257.6
16986	hum	11.95	6	2	2	object	638	1	net	521	257.6
16987	hum	11.95	6	2	2	object	639	5	control	431	257.6
16988	hum	11.95	6	2	2	object	640	5	control	548	257.6
16990	hum	11.95	6	2	2	object	642	6	control	434	257.6
16992	hum	11.95	6	2	2	object	644	3	control	453	257.6
16993	hum	11.95	6	2	2	object	645	3	control	571	257.6
16994	hum	11.95	6	2	2	object	646	3	control	441	257.6
16995	hum	11.95	6	2	2	object	647	3	control	466	257.6
17573	hum	11.95	6	2	2	mask	578	2	control	674	257.6
17574	hum	11.95	6	2	2	mask	579	6	control	459	257.6
17585	hum	11.95	6	2	2	mask	590	4	control	572	257.6
17586	hum	11.95	6	2	2	mask	591	3	control	537	257.6
17587	hum	11.95	6	2	2	mask	592	2	control	679	257.6
17589	hum	11.95	6	2	2	mask	594	4	control	467	257.6
17591	hum	11.95	6	2	2	mask	596	6	control	531	257.6
17592	hum	11.95	6	2	2	mask	597	2	control	503	257.6
17593	hum	11.95	6	2	2	mask	598	4	control	528	257.6
17594	hum	11.95	6	2	2	mask	599	3	mask_man	561	257.6
17595	hum	11.95	6	2	2	mask	600	3	control	540	257.6
17596	hum	11.95	6	2	2	mask	601	3	control	512	257.6
17597	hum	11.95	6	2	2	mask	602	5	control	483	257.6
17598	hum	11.95	6	2	2	mask	603	4	mask_man	539	257.6
17599	hum	11.95	6	2	2	mask	604	2	control	520	257.6
17600	hum	11.95	6	2	2	mask	605	2	control	505	257.6
17601	hum	11.95	6	2	2	mask	606	3	control	473	257.6
17603	hum	11.95	6	2	2	mask	608	6	control	480	257.6
17604	hum	11.95	6	2	2	mask	609	1	control	579	257.6
17605	hum	11.95	6	2	2	mask	610	5	control	459	257.6
17606	hum	11.95	6	2	2	mask	611	6	mask_man	430	257.6
17607	hum	11.95	6	2	2	mask	612	6	control	434	257.6
17608	hum	11.95	6	2	2	mask	613	3	control	508	257.6
17609	hum	11.95	6	2	2	mask	614	5	control	436	257.6
17610	hum	11.95	6	2	2	mask	615	2	mask_man	477	257.6
17611	hum	11.95	6	2	2	mask	616	3	control	439	257.6
17612	hum	11.95	6	2	2	mask	617	1	mask_man	498	257.6
17613	hum	11.95	6	2	2	mask	618	3	control	408	257.6
17614	hum	11.95	6	2	2	mask	619	3	mask_man	560	257.6
17615	hum	11.95	6	2	2	mask	620	4	control	494	257.6

17616	hum	11.95	6	2	2	mask	621	6	mask_man	442	257.6
17617	hum	11.95	6	2	2	mask	622	1	control	503	257.6
17618	hum	11.95	6	2	2	mask	623	1	control	533	257.6
17619	hum	11.95	6	2	2	mask	624	4	control	446	257.6
17620	hum	11.95	6	2	2	mask	625	5	mask_man	503	257.6
17621	hum	11.95	6	2	2	mask	626	2	control	470	257.6
17622	hum	11.95	6	2	2	mask	627	6	control	426	257.6
17624	hum	11.95	6	2	2	mask	629	5	control	411	257.6
17625	hum	11.95	6	2	2	mask	630	4	mask_man	422	257.6
17626	hum	11.95	6	2	2	mask	631	6	control	403	257.6
17627	hum	11.95	6	2	2	mask	632	2	control	449	257.6
17681	hum	11.95	6	2	2	mask	686	3	control	519	257.6
17682	hum	11.95	6	2	2	mask	687	2	mask_man	601	257.6
17683	hum	11.95	6	2	2	mask	688	1	control	636	257.6
17684	hum	11.95	6	2	2	mask	689	3	control	553	257.6
17685	hum	11.95	6	2	2	mask	690	5	mask_man	500	257.6
17686	hum	11.95	6	2	2	mask	691	1	control	536	257.6
17687	hum	11.95	6	2	2	mask	692	1	control	605	257.6
17688	hum	11.95	6	2	2	mask	693	2	control	527	257.6
17690	hum	11.95	6	2	2	mask	695	3	control	469	257.6
17691	hum	11.95	6	2	2	mask	696	2	control	578	257.6
17692	hum	11.95	6	2	2	mask	697	2	control	564	257.6
17694	hum	11.95	6	2	2	mask	699	4	control	541	257.6
17695	hum	11.95	6	2	2	mask	700	5	control	526	257.6
17696	hum	11.95	6	2	2	mask	701	3	mask_man	557	257.6
17697	hum	11.95	6	2	2	mask	702	6	control	468	257.6
17698	hum	11.95	6	2	2	mask	703	4	control	512	257.6
17699	hum	11.95	6	2	2	mask	704	2	mask_man	591	257.6
17700	hum	11.95	6	2	2	mask	705	5	control	484	257.6
17701	hum	11.95	6	2	2	mask	706	3	control	502	257.6
17702	hum	11.95	6	2	2	mask	707	6	control	425	257.6
17704	hum	11.95	6	2	2	mask	709	2	control	464	257.6
17705	hum	11.95	6	2	2	mask	710	5	control	422	257.6
17707	hum	11.95	6	2	2	mask	712	4	control	495	257.6
17708	hum	11.95	6	2	2	mask	713	6	control	471	257.6
17709	hum	11.95	6	2	2	mask	714	4	control	522	257.6
17711	hum	11.95	6	2	2	mask	716	1	control	674	257.6
17712	hum	11.95	6	2	2	mask	717	1	control	602	257.6
17713	hum	11.95	6	2	2	mask	718	4	control	453	257.6
17714	hum	11.95	6	2	2	mask	719	1	mask_man	613	257.6
17715	hum	11.95	6	2	2	mask	720	4	control	517	257.6
17716	hum	11.95	6	2	2	mask	721	3	control	506	257.6
17718	hum	11.95	6	2	2	mask	723	1	control	494	257.6
17720	hum	11.95	6	2	2	mask	725	5	control	473	257.6
17721	hum	11.95	6	2	2	mask	726	1	control	540	257.6
17722	hum	11.95	6	2	2	mask	727	1	mask_man	573	257.6
17723	hum	11.95	6	2	2	mask	728	4	control	566	257.6
17724	hum	11.95	6	2	2	mask	729	6	control	430	257.6
17725	hum	11.95	6	2	2	mask	730	6	mask_man	450	257.6
17726	hum	11.95	6	2	2	mask	731	5	control	511	257.6

17728	hum	11.95	6	2	2	mask	733	2	control	487		257.6
17729	hum	11.95	6	2	2	mask	734	5	control	473		257.6
17730	hum	11.95	6	2	2	mask	735	4	mask_man	508		257.6
17731	hum	11.95	6	2	2	mask	736	6	control	444		257.6
17732	hum	11.95	6	2	2	mask	737	3	control	434		257.6
17734	hum	11.95	6	2	2	mask	739	5	control	524		257.6
17837	hum	11.95	6	2	2	mask	842	4	control	776		257.6
17839	hum	11.95	6	2	2	mask	844	1	control	636		257.6
17840	hum	11.95	6	2	2	mask	845	6	control	461		257.6
17842	hum	11.95	6	2	2	mask	847	5	control	552		257.6
17844	hum	11.95	6	2	2	mask	849	6	control	532		257.6
17845	hum	11.95	6	2	2	mask	850	2	control	650		257.6
17846	hum	11.95	6	2	2	mask	851	1	control	601		257.6
17848	hum	11.95	6	2	2	mask	853	3	control	503		257.6
17849	hum	11.95	6	2	2	mask	854	6	control	484		257.6
17851	hum	11.95	6	2	2	mask	856	1	control	652		257.6
17852	hum	11.95	6	2	2	mask	857	3	control	517		257.6
17853	hum	11.95	6	2	2	mask	858	5	control	472		257.6
17854	nor	7.92	5	2	2	object	1	3	control	971		366.55
17877	nor	7.92	5	2	2	object	24	6	control		NA	366.55
17878	nor	7.92	5	2	2	object	25	6	control	400		366.55
17879	nor	7.92	5	2	2	object	26	1	control	396		366.55
17909	nor	7.92	5	2	2	object	56	3	control	391		366.55
17910	nor	7.92	5	2	2	object	57	4	control	371		366.55
17911	nor	7.92	5	2	2	object	58	4	control	425		366.55
17912	nor	7.92	5	2	2	object	59	3	control	407		366.55
17914	nor	7.92	5	2	2	object	61	5	control	419		366.55
17917	nor	7.92	5	2	2	object	64	4	control	418		366.55
17922	nor	7.92	5	2	2	object	69	6	control	461		366.55
17923	nor	7.92	5	2	2	object	70	4	control	355		366.55
17930	nor	7.92	5	2	2	object	77	3	control	411		366.55
17932	nor	7.92	5	2	2	object	79	4	control	422		366.55
17933	nor	7.92	5	2	2	object	80	5	control	362		366.55
17954	nor	7.92	5	2	2	object	101	6	control	467		366.55
17955	nor	7.92	5	2	2	object	102	2	net	420		366.55
17956	nor	7.92	5	2	2	object	103	5	control	1377		366.55
17963	nor	7.92	5	2	2	object	110	2	control	390		366.55
17964	nor	7.92	5	2	2	object	111	1	broom	910		366.55
17965	nor	7.92	5	2	2	object	112	6	control	866		366.55
17966	nor	7.92	5	2	2	object	113	6	glove	1066		366.55
17967	nor	7.92	5	2	2	object	114	5	control	2629		366.55
17968	nor	7.92	5	2	2	object	115	4	glove	443		366.55
17974	nor	7.92	5	2	2	object	121	1	control	563		366.55
17975	nor	7.92	5	2	2	object	122	3	control	579		366.55
17976	nor	7.92	5	2	2	object	123	1	control	460		366.55
17977	nor	7.92	5	2	2	object	124	1	net	854		366.55
18001	nor	7.92	5	2	2	object	148	5	control	460		366.55
18002	nor	7.92	5	2	2	object	149	3	control	696		366.55
18003	nor	7.92	5	2	2	object	150	4	broom	498		366.55
18028	nor	7.92	5	2	2	object	175	3	control	551		366.55

18029	nor	7.92	5	2	2	object	176	1	control	649	366.55
18030	nor	7.92	5	2	2	object	177	1	control	499	366.55
18032	nor	7.92	5	2	2	object	179	6	control	422	366.55
18037	nor	7.92	5	2	2	object	184	4	control	478	366.55
18039	nor	7.92	5	2	2	object	186	6	control	726	366.55
18040	nor	7.92	5	2	2	object	187	3	net	583	366.55
18041	nor	7.92	5	2	2	object	188	1	control	456	366.55
18055	nor	7.92	5	2	2	object	202	5	control	376	366.55
18069	nor	7.92	5	2	2	object	216	4	control	494	366.55
18070	nor	7.92	5	2	2	object	217	6	control	452	366.55
18071	nor	7.92	5	2	2	object	218	2	control	1497	366.55
18072	nor	7.92	5	2	2	object	219	5	net	527	366.55
18073	nor	7.92	5	2	2	object	220	5	control	473	366.55
18078	nor	7.92	5	2	2	object	225	4	control	770	366.55
18079	nor	7.92	5	2	2	object	226	6	control	461	366.55
18089	nor	7.92	5	2	2	object	236	3	control	417	366.55
18090	nor	7.92	5	2	2	object	237	6	control	349	366.55
18095	nor	7.92	5	2	2	object	242	4	control	463	366.55
18097	nor	7.92	5	2	2	object	244	1	glove	585	366.55
18109	nor	7.92	5	2	2	object	256	2	control	562	366.55
18111	nor	7.92	5	2	2	object	258	1	control	467	366.55
18113	nor	7.92	5	2	2	object	260	2	control	417	366.55
18123	nor	7.92	5	2	2	object	270	2	control	500	366.55
18128	nor	7.92	5	2	2	object	275	4	control	431	366.55
18129	nor	7.92	5	2	2	object	276	2	broom	611	366.55
18137	nor	7.92	5	2	2	object	284	3	control	485	366.55
18138	nor	7.92	5	2	2	object	285	5	control	392	366.55
18139	nor	7.92	5	2	2	object	286	3	control	459	366.55
18160	nor	7.92	5	2	2	object	307	4	net	1081	366.55
18162	nor	7.92	5	2	2	object	309	2	control	381	366.55
18163	nor	7.92	5	2	2	object	310	5	control	591	366.55
18167	nor	7.92	5	2	2	object	314	2	control	418	366.55
18168	nor	7.92	5	2	2	object	315	6	net	505	366.55
18169	nor	7.92	5	2	2	object	316	2	control	427	366.55
18170	nor	7.92	5	2	2	object	317	1	control	379	366.55
18172	nor	7.92	5	2	2	object	319	3	glove	549	366.55
18173	nor	7.92	5	2	2	object	320	2	control	421	366.55
18182	nor	7.92	5	2	2	object	329	1	control	413	366.55
18184	nor	7.92	5	2	2	object	331	4	control	339	366.55
18185	nor	7.92	5	2	2	object	332	3	control	449	366.55
18186	nor	7.92	5	2	2	object	333	2	control	447	366.55
18217	nor	7.92	5	2	2	object	364	3	broom	453	366.55
18218	nor	7.92	5	2	2	object	365	2	control	442	366.55
18219	nor	7.92	5	2	2	object	366	6	control	368	366.55
18220	nor	7.92	5	2	2	object	367	1	control	480	366.55
18222	nor	7.92	5	2	2	object	369	1	control	457	366.55
18227	nor	7.92	5	2	2	object	374	3	control	400	366.55
18228	nor	7.92	5	2	2	object	375	6	control	387	366.55
18230	nor	7.92	5	2	2	object	377	1	control	489	366.55
18233	nor	7.92	5	2	2	object	380	3	control	744	366.55

18242	nor	7.92	5	2	2	object	389	2	glove	519	366.55
18243	nor	7.92	5	2	2	object	390	4	control	448	366.55
18244	nor	7.92	5	2	2	object	391	3	control	579	366.55
18275	nor	7.92	5	2	2	object	422	5	control	419	366.55
18276	nor	7.92	5	2	2	object	423	5	control	434	366.55
18278	nor	7.92	5	2	2	object	425	6	control	439	366.55
18283	nor	7.92	5	2	2	object	430	2	control	443	366.55
18302	nor	7.92	5	2	2	object	449	6	control	494	366.55
18303	nor	7.92	5	2	2	object	450	6	broom	592	366.55
18304	nor	7.92	5	2	2	object	451	1	control	499	366.55
18306	nor	7.92	5	2	2	object	453	1	control	511	366.55
18308	nor	7.92	5	2	2	object	455	3	control	430	366.55
18309	nor	7.92	5	2	2	object	456	5	control	597	366.55
18310	nor	7.92	5	2	2	object	457	3	control	429	366.55
18311	nor	7.92	5	2	2	object	458	5	control	415	366.55
18315	nor	7.92	5	2	2	mask	4	2	control	647	366.55
18316	nor	7.92	5	2	2	mask	5	4	control	407	366.55
18317	nor	7.92	5	2	2	mask	6	4	control	378	366.55
18319	nor	7.92	5	2	2	mask	8	5	control	NA	366.55
18321	nor	7.92	5	2	2	mask	10	4	control	514	366.55
18322	nor	7.92	5	2	2	mask	11	5	control	323	366.55
18323	nor	7.92	5	2	2	mask	12	6	control	345	366.55
18324	nor	7.92	5	2	2	mask	13	1	control	526	366.55
18326	nor	7.92	5	2	2	mask	15	2	control	345	366.55
18327	nor	7.92	5	2	2	mask	16	2	control	364	366.55
18330	nor	7.92	5	2	2	mask	19	1	control	483	366.55
18334	nor	7.92	5	2	2	mask	23	1	mask_man	512	366.55
18335	nor	7.92	5	2	2	mask	24	1	control	367	366.55
18336	nor	7.92	5	2	2	mask	25	6	control	451	366.55
18340	nor	7.92	5	2	2	mask	29	6	control	408	366.55
18341	nor	7.92	5	2	2	mask	30	5	mask_man	411	366.55
18342	nor	7.92	5	2	2	mask	31	6	control	376	366.55
18345	nor	7.92	5	2	2	mask	34	2	control	639	366.55
18348	nor	7.92	5	2	2	mask	37	6	control	622	366.55
18352	nor	7.92	5	2	2	mask	41	2	control	339	366.55
18362	nor	7.92	5	2	2	mask	51	5	control	318	366.55
18363	nor	7.92	5	2	2	mask	52	4	control	368	366.55
18365	nor	7.92	5	2	2	mask	54	4	mask_man	332	366.55
18369	nor	7.92	5	2	2	mask	58	1	control	392	366.55
18370	nor	7.92	5	2	2	mask	59	5	control	363	366.55
18372	nor	7.92	5	2	2	mask	61	3	control	488	366.55
18378	nor	7.92	5	2	2	mask	67	4	control	492	366.55
18379	nor	7.92	5	2	2	mask	68	1	mask_man	441	366.55
18380	nor	7.92	5	2	2	mask	69	1	control	809	366.55
18381	nor	7.92	5	2	2	mask	70	4	control	423	366.55
18391	nor	7.92	5	2	2	mask	80	5	control	347	366.55
18393	nor	7.92	5	2	2	mask	82	3	control	488	366.55
18394	nor	7.92	5	2	2	mask	83	1	control	498	366.55
18407	nor	7.92	5	2	2	mask	96	1	control	456	366.55
18410	nor	7.92	5	2	2	mask	99	6	mask_man	396	366.55

18411	nor	7.92	5	2	2	mask	100	1	control	496	366.55
18426	nor	7.92	5	2	2	mask	115	3	mask_man	359	366.55
18427	nor	7.92	5	2	2	mask	116	3	control	472	366.55
18428	nor	7.92	5	2	2	mask	117	2	control	487	366.55
18430	nor	7.92	5	2	2	mask	119	6	control	440	366.55
18434	nor	7.92	5	2	2	mask	123	3	control	407	366.55
18435	nor	7.92	5	2	2	mask	124	4	control	566	366.55
18438	nor	7.92	5	2	2	mask	127	1	control	341	366.55
18439	nor	7.92	5	2	2	mask	128	3	control	407	366.55
18443	nor	7.92	5	2	2	mask	132	5	mask_man	1212	366.55
18444	nor	7.92	5	2	2	mask	133	2	control	529	366.55
18456	nor	7.92	5	2	2	mask	145	5	control	334	366.55
18457	nor	7.92	5	2	2	mask	146	2	mask_man	623	366.55
18458	nor	7.92	5	2	2	mask	147	4	control	323	366.55
18459	nor	7.92	5	2	2	mask	148	4	control	394	366.55
18460	nor	7.92	5	2	2	mask	149	4	control	382	366.55
18466	nor	7.92	5	2	2	mask	155	4	mask_man	395	366.55
18467	nor	7.92	5	2	2	mask	156	2	control	394	366.55
18469	nor	7.92	5	2	2	mask	158	2	mask_man	759	366.55
18470	nor	7.92	5	2	2	mask	159	6	control	2882	366.55
18471	nor	7.92	5	2	2	mask	160	4	control	464	366.55
18481	nor	7.92	5	2	2	mask	170	1	control	761	366.55
18483	nor	7.92	5	2	2	mask	172	3	control	465	366.55
18484	nor	7.92	5	2	2	mask	173	5	control	642	366.55
18489	nor	7.92	5	2	2	mask	178	3	control	959	366.55
18490	nor	7.92	5	2	2	mask	179	3	control	610	366.55
18491	nor	7.92	5	2	2	mask	180	6	mask_man	458	366.55
18492	nor	7.92	5	2	2	mask	181	3	control	484	366.55
18495	nor	7.92	5	2	2	mask	184	2	control	354	366.55
18498	nor	7.92	5	2	2	mask	187	1	mask_man	584	366.55
18499	nor	7.92	5	2	2	mask	188	3	control	474	366.55
18501	nor	7.92	5	2	2	mask	190	6	control	425	366.55
18502	nor	7.92	5	2	2	mask	191	1	control	421	366.55
18504	nor	7.92	5	2	2	mask	193	6	control	542	366.55
18506	nor	7.92	5	2	2	mask	195	4	control	437	366.55
18531	nor	7.92	5	2	2	mask	220	1	control	804	366.55
18533	nor	7.92	5	2	2	mask	222	6	control	398	366.55
18534	nor	7.92	5	2	2	mask	223	4	control	352	366.55
18536	nor	7.92	5	2	2	mask	225	5	control	369	366.55
18537	nor	7.92	5	2	2	mask	226	5	mask_man	394	366.55
18538	nor	7.92	5	2	2	mask	227	2	control	541	366.55
18547	nor	7.92	5	2	2	mask	236	5	control	398	366.55
18549	nor	7.92	5	2	2	mask	238	5	control	357	366.55
18550	nor	7.92	5	2	2	mask	239	4	control	356	366.55
18551	nor	7.92	5	2	2	mask	240	2	control	341	366.55
18552	nor	7.92	5	2	2	mask	241	6	mask_man	461	366.55
18553	nor	7.92	5	2	2	mask	242	5	control	441	366.55
18555	nor	7.92	5	2	2	mask	244	6	control	393	366.55
18566	nor	7.92	5	2	2	mask	255	2	control	431	366.55
18567	nor	7.92	5	2	2	mask	256	3	control	662	366.55

18584	nor	7.92	5	2	2	mask	273	2	mask_man	1676	366.55
18585	nor	7.92	5	2	2	mask	274	5	control	1126	366.55
18586	nor	7.92	5	2	2	mask	275	2	control	542	366.55
18587	nor	7.92	5	2	2	mask	276	6	control	425	366.55
18609	nor	7.92	5	2	2	mask	298	3	mask_man	466	366.55
18610	nor	7.92	5	2	2	mask	299	4	control	393	366.55
18611	nor	7.92	5	2	2	mask	300	4	mask_man	366	366.55
18612	nor	7.92	5	2	2	mask	301	3	control	414	366.55
18613	nor	7.92	5	2	2	mask	302	5	control	370	366.55
18614	nor	7.92	5	2	2	mask	303	3	mask_man	417	366.55
18630	nor	7.92	5	2	2	mask	319	3	control	NA	366.55
18633	nor	7.92	5	2	2	mask	322	6	control	NA	366.55
18664	nor	7.92	5	2	2	mask	353	5	control	351	366.55
18665	nor	7.92	5	2	2	mask	354	4	control	392	366.55
18666	nor	7.92	5	2	2	mask	355	1	control	421	366.55
19398	osk	11.71	7	2	2	object	26	2	control	424	291.94
19570	osk	11.71	7	2	2	object	198	6	control	660	291.94
19610	osk	11.71	7	2	2	object	238	4	control	535	291.94
19611	osk	11.71	7	2	2	object	239	1	control	511	291.94
19612	osk	11.71	7	2	2	object	240	6	control	451	291.94
19613	osk	11.71	7	2	2	object	241	5	control	439	291.94
19615	osk	11.71	7	2	2	object	243	2	control	474	291.94
19616	osk	11.71	7	2	2	object	244	4	glove	553	291.94
19617	osk	11.71	7	2	2	object	245	3	control	503	291.94
19618	osk	11.71	7	2	2	object	246	6	control	530	291.94
19645	osk	11.71	7	2	2	object	273	3	control	480	291.94
19646	osk	11.71	7	2	2	object	274	1	control	449	291.94
19647	osk	11.71	7	2	2	object	275	1	control	583	291.94
19649	osk	11.71	7	2	2	object	277	6	control	453	291.94
19650	osk	11.71	7	2	2	object	278	1	control	491	291.94
19651	osk	11.71	7	2	2	object	279	2	control	509	291.94
19652	osk	11.71	7	2	2	object	280	6	net	957	291.94
19653	osk	11.71	7	2	2	object	281	6	control	402	291.94
19654	osk	11.71	7	2	2	object	282	5	control	449	291.94
19655	osk	11.71	7	2	2	object	283	1	control	540	291.94
19656	osk	11.71	7	2	2	object	284	3	broom	498	291.94
19657	osk	11.71	7	2	2	object	285	1	control	609	291.94
19658	osk	11.71	7	2	2	object	286	6	control	395	291.94
19659	osk	11.71	7	2	2	object	287	4	broom	608	291.94
19667	osk	11.71	7	2	2	object	295	2	control	521	291.94
19668	osk	11.71	7	2	2	object	296	6	control	487	291.94
19670	osk	11.71	7	2	2	object	298	6	control	398	291.94
19671	osk	11.71	7	2	2	object	299	2	control	477	291.94
19673	osk	11.71	7	2	2	object	301	5	control	403	291.94
19718	osk	11.71	7	2	2	object	346	5	control	420	291.94
19720	osk	11.71	7	2	2	object	348	4	control	519	291.94
19721	osk	11.71	7	2	2	object	349	6	control	411	291.94
19722	osk	11.71	7	2	2	object	350	4	control	531	291.94
19724	osk	11.71	7	2	2	object	352	5	control	436	291.94
19726	osk	11.71	7	2	2	object	354	2	control	472	291.94

19741	osk	11.71	7	2	2	object	369	5	control	428	291.94
19742	osk	11.71	7	2	2	object	370	2	control	442	291.94
19743	osk	11.71	7	2	2	object	371	2	glove	448	291.94
19744	osk	11.71	7	2	2	object	372	1	control	558	291.94
19745	osk	11.71	7	2	2	object	373	3	control	461	291.94
19746	osk	11.71	7	2	2	object	374	2	control	465	291.94
19770	osk	11.71	7	2	2	object	398	1	net	465	291.94
19771	osk	11.71	7	2	2	object	399	3	control	432	291.94
19772	osk	11.71	7	2	2	object	400	1	control	614	291.94
19773	osk	11.71	7	2	2	object	401	2	control	485	291.94
19775	osk	11.71	7	2	2	object	403	3	control	531	291.94
19777	osk	11.71	7	2	2	object	405	4	control	535	291.94
19778	osk	11.71	7	2	2	object	406	2	net	565	291.94
19779	osk	11.71	7	2	2	object	407	3	control	421	291.94
19780	osk	11.71	7	2	2	object	408	3	net	660	291.94
19781	osk	11.71	7	2	2	object	409	1	control	610	291.94
19845	osk	11.71	7	2	2	object	473	6	glove	420	291.94
19846	osk	11.71	7	2	2	object	474	5	control	399	291.94
19847	osk	11.71	7	2	2	object	475	5	control	387	291.94
19848	osk	11.71	7	2	2	object	476	5	control	394	291.94
19849	osk	11.71	7	2	2	object	477	2	broom	453	291.94
19883	osk	11.71	7	2	2	object	511	6	control	400	291.94
19884	osk	11.71	7	2	2	object	512	3	control	495	291.94
19885	osk	11.71	7	2	2	object	513	5	broom	439	291.94
19886	osk	11.71	7	2	2	object	514	2	control	461	291.94
19887	osk	11.71	7	2	2	object	515	3	control	467	291.94
19888	osk	11.71	7	2	2	object	516	4	control	468	291.94
19889	osk	11.71	7	2	2	object	517	5	net	523	291.94
19890	osk	11.71	7	2	2	object	518	5	control	449	291.94
19891	osk	11.71	7	2	2	object	519	4	control	563	291.94
19902	osk	11.71	7	2	2	object	530	4	control	495	291.94
19903	osk	11.71	7	2	2	object	531	5	glove	446	291.94
19904	osk	11.71	7	2	2	object	532	5	control	422	291.94
19905	osk	11.71	7	2	2	object	533	4	control	485	291.94
19906	osk	11.71	7	2	2	object	534	3	glove	488	291.94
19907	osk	11.71	7	2	2	object	535	4	control	527	291.94
19909	osk	11.71	7	2	2	object	537	5	control	464	291.94
19911	osk	11.71	7	2	2	object	539	1	control	498	291.94
19926	osk	11.71	7	2	2	object	554	3	control	505	291.94
19927	osk	11.71	7	2	2	object	555	6	broom	448	291.94
19928	osk	11.71	7	2	2	object	556	2	control	530	291.94
19944	osk	11.71	7	2	2	object	572	1	control	570	291.94
19945	osk	11.71	7	2	2	object	573	4	control	463	291.94
19946	osk	11.71	7	2	2	object	574	4	control	490	291.94
19948	osk	11.71	7	2	2	object	576	6	control	456	291.94
19949	osk	11.71	7	2	2	object	577	3	control	517	291.94
19950	osk	11.71	7	2	2	object	578	3	control	486	291.94
19951	osk	11.71	7	2	2	object	579	4	net	552	291.94
19968	osk	11.71	7	2	2	object	596	4	control	428	291.94
19969	osk	11.71	7	2	2	object	597	3	control	462	291.94

19970	osk	11.71	7	2	2	object	598	1	control	545	291.94
19972	osk	11.71	7	2	2	object	600	6	control	477	291.94
19973	osk	11.71	7	2	2	object	601	2	control	509	291.94
19974	osk	11.71	7	2	2	object	602	6	control	433	291.94
19976	osk	11.71	7	2	2	object	604	2	control	458	291.94
19977	osk	11.71	7	2	2	object	605	1	glove	598	291.94
19978	osk	11.71	7	2	2	object	606	4	control	547	291.94
19991	osk	11.71	7	2	2	object	619	1	control	530	291.94
19992	osk	11.71	7	2	2	object	620	1	broom	510	291.94
19993	osk	11.71	7	2	2	object	621	2	control	588	291.94
20018	osk	11.71	7	2	2	object	646	6	control	400	291.94
20019	osk	11.71	7	2	2	object	647	5	control	416	291.94
20020	osk	11.71	7	2	2	object	648	6	control	398	291.94
20021	osk	11.71	7	2	2	object	649	3	control	467	291.94
20022	osk	11.71	7	2	2	mask	1	2	control	452	291.94
20023	osk	11.71	7	2	2	mask	2	2	control	411	291.94
20037	osk	11.71	7	2	2	mask	16	6	control	394	291.94
20038	osk	11.71	7	2	2	mask	17	5	control	394	291.94
20040	osk	11.71	7	2	2	mask	19	4	control	430	291.94
20041	osk	11.71	7	2	2	mask	20	6	control	401	291.94
20042	osk	11.71	7	2	2	mask	21	1	control	562	291.94
20044	osk	11.71	7	2	2	mask	23	6	control	605	291.94
20068	osk	11.71	7	2	2	mask	47	4	control	427	291.94
20069	osk	11.71	7	2	2	mask	48	5	control	387	291.94
20070	osk	11.71	7	2	2	mask	49	5	mask_man	303	291.94
20072	osk	11.71	7	2	2	mask	51	1	control	591	291.94
20074	osk	11.71	7	2	2	mask	53	6	control	418	291.94
20075	osk	11.71	7	2	2	mask	54	4	control	506	291.94
20076	osk	11.71	7	2	2	mask	55	2	mask_man	821	291.94
20077	osk	11.71	7	2	2	mask	56	4	control	569	291.94
20078	osk	11.71	7	2	2	mask	57	4	control	442	291.94
20079	osk	11.71	7	2	2	mask	58	5	control	401	291.94
20081	osk	11.71	7	2	2	mask	60	2	control	480	291.94
20083	osk	11.71	7	2	2	mask	62	1	control	503	291.94
20085	osk	11.71	7	2	2	mask	64	3	control	500	291.94
20087	osk	11.71	7	2	2	mask	66	2	control	502	291.94
20088	osk	11.71	7	2	2	mask	67	2	control	449	291.94
20089	osk	11.71	7	2	2	mask	68	3	mask_man	520	291.94
20090	osk	11.71	7	2	2	mask	69	2	control	488	291.94
20092	osk	11.71	7	2	2	mask	71	1	control	466	291.94
20093	osk	11.71	7	2	2	mask	72	6	control	397	291.94
20094	osk	11.71	7	2	2	mask	73	2	control	490	291.94
20095	osk	11.71	7	2	2	mask	74	6	mask_man	718	291.94
20096	osk	11.71	7	2	2	mask	75	6	control	392	291.94
20097	osk	11.71	7	2	2	mask	76	5	control	490	291.94
20098	osk	11.71	7	2	2	mask	77	3	control	436	291.94
20099	osk	11.71	7	2	2	mask	78	2	mask_man	472	291.94
20100	osk	11.71	7	2	2	mask	79	3	control	507	291.94
20109	osk	11.71	7	2	2	mask	88	5	mask_man	447	291.94
20110	osk	11.71	7	2	2	mask	89	5	control	374	291.94

20111	osk	11.71	7	2	2	mask	90	4	control	533	291.94
20112	osk	11.71	7	2	2	mask	91	1	control	578	291.94
20113	osk	11.71	7	2	2	mask	92	4	mask_man	418	291.94
20114	osk	11.71	7	2	2	mask	93	4	control	551	291.94
20115	osk	11.71	7	2	2	mask	94	2	control	493	291.94
20116	osk	11.71	7	2	2	mask	95	3	control	437	291.94
20117	osk	11.71	7	2	2	mask	96	3	mask_man	487	291.94
20118	osk	11.71	7	2	2	mask	97	3	control	460	291.94
20119	osk	11.71	7	2	2	mask	98	1	control	613	291.94
20120	osk	11.71	7	2	2	mask	99	2	control	470	291.94
20136	osk	11.71	7	2	2	mask	115	6	control	416	291.94
20137	osk	11.71	7	2	2	mask	116	3	control	471	291.94
20138	osk	11.71	7	2	2	mask	117	5	mask_man	411	291.94
20139	osk	11.71	7	2	2	mask	118	1	control	582	291.94
20141	osk	11.71	7	2	2	mask	120	5	control	413	291.94
20157	osk	11.71	7	2	2	mask	136	3	mask_man	484	291.94
20158	osk	11.71	7	2	2	mask	137	6	control	409	291.94
20159	osk	11.71	7	2	2	mask	138	4	control	553	291.94
20160	osk	11.71	7	2	2	mask	139	2	control	459	291.94
20162	osk	11.71	7	2	2	mask	141	6	control	378	291.94
20163	osk	11.71	7	2	2	mask	142	2	mask_man	480	291.94
20164	osk	11.71	7	2	2	mask	143	4	control	573	291.94
20165	osk	11.71	7	2	2	mask	144	1	control	532	291.94
20166	osk	11.71	7	2	2	mask	145	3	control	473	291.94
20168	osk	11.71	7	2	2	mask	147	2	control	476	291.94
20223	osk	11.71	7	2	2	mask	202	6	mask_man	448	291.94
20224	osk	11.71	7	2	2	mask	203	2	control	494	291.94
20225	osk	11.71	7	2	2	mask	204	5	control	447	291.94
20227	osk	11.71	7	2	2	mask	206	6	control	422	291.94
20228	osk	11.71	7	2	2	mask	207	4	control	554	291.94
20229	osk	11.71	7	2	2	mask	208	5	control	417	291.94
20230	osk	11.71	7	2	2	mask	209	1	mask_man	525	291.94
20269	osk	11.71	7	2	2	mask	248	3	control	511	291.94
20270	osk	11.71	7	2	2	mask	249	6	control	441	291.94
20271	osk	11.71	7	2	2	mask	250	3	control	510	291.94
20273	osk	11.71	7	2	2	mask	252	6	control	419	291.94
20275	osk	11.71	7	2	2	mask	254	4	control	566	291.94
20276	osk	11.71	7	2	2	mask	255	2	control	490	291.94
20277	osk	11.71	7	2	2	mask	256	4	mask_man	467	291.94
20374	osk	11.71	7	2	2	mask	353	4	control	447	291.94
20375	osk	11.71	7	2	2	mask	354	4	mask_man	461	291.94
20376	osk	11.71	7	2	2	mask	355	3	control	481	291.94
20378	osk	11.71	7	2	2	mask	357	1	mask_man	616	291.94
20379	osk	11.71	7	2	2	mask	358	3	control	461	291.94
20380	osk	11.71	7	2	2	mask	359	4	control	455	291.94
20383	osk	11.71	7	2	2	mask	362	1	mask_man	488	291.94
20384	osk	11.71	7	2	2	mask	363	6	control	411	291.94
20385	osk	11.71	7	2	2	mask	364	2	control	505	291.94
20386	osk	11.71	7	2	2	mask	365	5	control	385	291.94
20459	osk	11.71	7	2	2	mask	438	1	control	480	291.94

20460	osk	11.71	7	2	2	mask	439	1	control	493	291.94
20462	osk	11.71	7	2	2	mask	441	5	control	378	291.94
20463	osk	11.71	7	2	2	mask	442	1	control	468	291.94
20465	osk	11.71	7	2	2	mask	444	3	control	418	291.94
20466	osk	11.71	7	2	2	mask	445	6	mask_man	392	291.94
20467	osk	11.71	7	2	2	mask	446	1	control	490	291.94
20469	osk	11.71	7	2	2	mask	448	5	control	399	291.94
20470	osk	11.71	7	2	2	mask	449	3	control	414	291.94
20471	osk	11.71	7	2	2	mask	450	2	control	445	291.94
20472	osk	11.71	7	2	2	mask	451	3	control	472	291.94
20483	osk	11.79	7	3	1	face	11	2	control	572	291.94
20484	osk	11.79	7	3	1	face	12	2	control	443	291.94
20485	osk	11.79	7	3	1	face	13	5	control	426	291.94
20487	osk	11.79	7	3	1	face	15	1	control	484	291.94
20516	osk	11.79	7	3	1	face	44	2	control	501	291.94
20517	osk	11.79	7	3	1	face	45	4	control	556	291.94
20518	osk	11.79	7	3	1	face	46	3	control	466	291.94
20519	osk	11.79	7	3	1	face	47	4	control	558	291.94
20521	osk	11.79	7	3	1	face	49	5	control	414	291.94
20522	osk	11.79	7	3	1	face	50	6	face	475	291.94
20523	osk	11.79	7	3	1	face	51	1	control	518	291.94
20524	osk	11.79	7	3	1	face	52	4	face	455	291.94
20525	osk	11.79	7	3	1	face	53	2	control	491	291.94
20526	osk	11.79	7	3	1	face	54	2	face	596	291.94
20527	osk	11.79	7	3	1	face	55	6	control	482	291.94
20528	osk	11.79	7	3	1	face	56	3	control	543	291.94
20529	osk	11.79	7	3	1	face	57	6	control	536	291.94
20530	osk	11.79	7	3	1	face	58	5	face	438	291.94
20531	osk	11.79	7	3	1	face	59	6	control	480	291.94
20532	osk	11.79	7	3	1	face	60	5	face	562	291.94
20533	osk	11.79	7	3	1	face	61	6	control	451	291.94
20535	osk	11.79	7	3	1	face	63	5	control	451	291.94
20537	osk	11.79	7	3	1	face	65	1	control	677	291.94
20539	osk	11.79	7	3	1	face	67	5	control	575	291.94
20540	osk	11.79	7	3	1	face	68	1	control	530	291.94
20541	osk	11.79	7	3	1	face	69	4	control	627	291.94
20543	osk	11.79	7	3	1	face	71	3	control	545	291.94
20545	osk	11.79	7	3	1	face	73	1	control	533	291.94
20556	osk	11.79	7	3	1	face	84	3	control	487	291.94
20557	osk	11.79	7	3	1	face	85	4	control	545	291.94
20558	osk	11.79	7	3	1	face	86	5	control	406	291.94
20559	osk	11.79	7	3	1	face	87	2	face	421	291.94
20560	osk	11.79	7	3	1	face	88	6	control	449	291.94
20561	osk	11.79	7	3	1	face	89	3	control	448	291.94
20562	osk	11.79	7	3	1	face	90	2	control	529	291.94
20564	osk	11.79	7	3	1	face	92	6	control	439	291.94
20566	osk	11.79	7	3	1	face	94	2	control	530	291.94
20568	osk	11.79	7	3	1	face	96	4	control	556	291.94
20569	osk	11.79	7	3	1	face	97	6	control	576	291.94
20573	osk	11.79	7	3	1	face	101	1	control	610	291.94

20574	osk	11.79	7	3	1 face	102	3 control	496	291.94
20575	osk	11.79	7	3	1 face	103	4 control	511	291.94
20577	osk	11.79	7	3	1 face	105	4 control	616	291.94
20578	osk	11.79	7	3	1 face	106	1 face	749	291.94
20579	osk	11.79	7	3	1 face	107	5 control	455	291.94
20580	osk	11.79	7	3	1 face	108	3 control	508	291.94
20581	osk	11.79	7	3	1 face	109	3 control	525	291.94
20583	osk	11.79	7	3	1 face	111	1 control	697	291.94
20585	osk	11.79	7	3	1 face	113	5 control	493	291.94
20586	osk	11.79	7	3	1 face	114	5 control	469	291.94
20587	osk	11.79	7	3	1 face	115	4 face	562	291.94
20594	osk	11.79	7	3	1 face	122	3 control	516	291.94
20595	osk	11.79	7	3	1 face	123	2 control	455	291.94
20596	osk	11.79	7	3	1 face	124	1 face	547	291.94
20597	osk	11.79	7	3	1 face	125	5 control	451	291.94
20598	osk	11.79	7	3	1 face	126	2 control	485	291.94
20600	osk	11.79	7	3	1 face	128	6 control	442	291.94
20611	osk	11.79	7	3	1 face	139	3 control	493	291.94
20612	osk	11.79	7	3	1 face	140	3 face	532	291.94
20613	osk	11.79	7	3	1 face	141	2 control	527	291.94
20615	osk	11.79	7	3	1 face	143	3 face	484	291.94
20616	osk	11.79	7	3	1 face	144	5 control	450	291.94
20617	osk	11.79	7	3	1 face	145	2 control	511	291.94
20619	osk	11.79	7	3	1 face	147	1 control	672	291.94
20620	osk	11.79	7	3	1 face	148	2 control	575	291.94
20621	osk	11.79	7	3	1 face	149	4 control	506	291.94
20622	osk	11.79	7	3	1 face	150	4 face	551	291.94
20623	osk	11.79	7	3	1 face	151	6 control	414	291.94
20624	osk	11.79	7	3	1 face	152	5 control	445	291.94
20625	osk	11.79	7	3	1 face	153	5 control	437	291.94
20678	osk	11.79	7	3	1 face	206	3 face	487	291.94
20679	osk	11.79	7	3	1 face	207	5 control	417	291.94
20680	osk	11.79	7	3	1 face	208	4 control	558	291.94
20681	osk	11.79	7	3	1 face	209	3 control	459	291.94
20683	osk	11.79	7	3	1 face	211	4 control	517	291.94
20684	osk	11.79	7	3	1 face	212	2 control	480	291.94
20686	osk	11.79	7	3	1 face	214	3 control	469	291.94
20687	osk	11.79	7	3	1 face	215	1 control	524	291.94
20688	osk	11.79	7	3	1 face	216	5 face	421	291.94
20689	osk	11.79	7	3	1 face	217	6 control	402	291.94
20690	osk	11.79	7	3	1 face	218	4 control	481	291.94
20691	osk	11.79	7	3	1 face	219	6 face	433	291.94
20692	osk	11.79	7	3	1 face	220	1 control	518	291.94
20694	osk	11.79	7	3	1 face	222	6 control	389	291.94
20891	osk	11.79	7	3	1 face	419	1 face	748	291.94
20893	osk	11.79	7	3	1 face	421	2 control	486	291.94
20895	osk	11.79	7	3	1 face	423	2 control	476	291.94
20896	osk	11.79	7	3	1 face	424	4 control	529	291.94
20899	osk	11.79	7	3	1 face	427	2 control	485	291.94
20900	osk	11.79	7	3	1 face	428	2 face	535	291.94

20901	osk	11.79	7	3	1	face	429	5	control	451	291.94
20902	osk	11.79	7	3	1	face	430	4	control	538	291.94
21101	osk	11.79	7	3	1	face	629	4	control	469	291.94
21102	osk	11.79	7	3	1	face	630	5	control	516	291.94
21108	osk	11.79	7	3	1	face	636	2	control	473	291.94
21109	bex	7.15	7	3	1	face	1	2	control	549	240.042
21110	bex	7.15	7	3	1	face	2	1	control	579	240.042
21111	bex	7.15	7	3	1	face	3	2	control	499	240.042
21113	bex	7.15	7	3	1	face	5	2	control	441	240.042
21114	bex	7.15	7	3	1	face	6	6	control	413	240.042
21115	bex	7.15	7	3	1	face	7	3	control	426	240.042
21116	bex	7.15	7	3	1	face	8	4	control	451	240.042
21118	bex	7.15	7	3	1	face	10	1	control	439	240.042
21119	bex	7.15	7	3	1	face	11	1	face	NA	240.042
21121	bex	7.15	7	3	1	face	13	6	control	470	240.042
21122	bex	7.15	7	3	1	face	14	5	control	511	240.042
21123	bex	7.15	7	3	1	face	15	6	control	442	240.042
21125	bex	7.15	7	3	1	face	17	1	control	574	240.042
21127	bex	7.15	7	3	1	face	19	1	control	726	240.042
21128	bex	7.15	7	3	1	face	20	3	control	500	240.042
21129	bex	7.15	7	3	1	face	21	4	control	471	240.042
21130	bex	7.15	7	3	1	face	22	3	face	NA	240.042
21132	bex	7.15	7	3	1	face	24	1	control	2127	240.042
21134	bex	7.15	7	3	1	face	26	3	control	462	240.042
21135	bex	7.15	7	3	1	face	27	6	control	446	240.042
21137	bex	7.15	7	3	1	face	29	5	control	440	240.042
21138	bex	7.15	7	3	1	face	30	1	control	559	240.042
21139	bex	7.15	7	3	1	face	31	6	control	422	240.042
21140	bex	7.15	7	3	1	face	32	5	face	NA	240.042
21142	bex	7.15	7	3	1	face	34	6	control	426	240.042
21143	bex	7.15	7	3	1	face	35	5	control	539	240.042
21144	bex	7.15	7	3	1	face	36	1	control	586	240.042
21145	bex	7.15	7	3	1	face	37	4	face	NA	240.042
21148	bex	7.15	7	3	1	face	40	2	control	662	240.042
21149	bex	7.15	7	3	1	face	41	6	face	NA	240.042
21150	bex	7.15	7	3	1	face	42	3	control	498	240.042
21151	bex	7.15	7	3	1	face	43	1	control	570	240.042
21152	bex	7.15	7	3	1	face	44	6	control	474	240.042
21154	bex	7.15	7	3	1	face	46	3	control	497	240.042
21155	bex	7.15	7	3	1	face	47	2	control	529	240.042
21156	bex	7.15	7	3	1	face	48	2	face	NA	240.042
21157	bex	7.15	7	3	1	face	49	6	control	474	240.042
21158	bex	7.15	7	3	1	face	50	2	control	592	240.042
21159	bex	7.15	7	3	1	face	51	4	control	NA	240.042
21161	bex	7.15	7	3	1	face	53	5	control	450	240.042
21162	bex	7.15	7	3	1	face	54	1	control	NA	240.042
21163	bex	7.15	7	3	1	face	55	4	control	605	240.042
21164	bex	7.15	7	3	1	face	56	6	face	NA	240.042
21165	bex	7.15	7	3	1	face	57	1	control	551	240.042
21167	bex	7.15	7	3	1	face	59	5	control	420	240.042

21169	bex	7.15	7	3	1	face	61	3	control	535	240.042
21170	bex	7.15	7	3	1	face	62	6	control	438	240.042
21171	bex	7.15	7	3	1	face	63	6	face	NA	240.042
21172	bex	7.15	7	3	1	face	64	2	control	521	240.042
21173	bex	7.15	7	3	1	face	65	2	control	559	240.042
21175	bex	7.15	7	3	1	face	67	6	control	438	240.042
21176	bex	7.15	7	3	1	face	68	5	face	NA	240.042
21178	bex	7.15	7	3	1	face	70	2	control	534	240.042
21179	bex	7.15	7	3	1	face	71	5	control	534	240.042
21180	bex	7.15	7	3	1	face	72	3	face	NA	240.042
21181	bex	7.15	7	3	1	face	73	6	control	474	240.042
21182	bex	7.15	7	3	1	face	74	5	control	480	240.042
21183	bex	7.15	7	3	1	face	75	2	control	575	240.042
21185	bex	7.15	7	3	1	face	77	4	control	496	240.042
21186	bex	7.15	7	3	1	face	78	2	control	561	240.042
21187	bex	7.15	7	3	1	face	79	4	face	NA	240.042
21191	bex	7.15	7	3	1	face	83	2	control	538	240.042
21192	bex	7.15	7	3	1	face	84	3	face	NA	240.042
21193	bex	7.15	7	3	1	face	85	1	control	552	240.042
21194	bex	7.15	7	3	1	face	86	6	control	446	240.042
21195	bex	7.15	7	3	1	face	87	5	control	487	240.042
21197	bex	7.15	7	3	1	face	89	4	control	550	240.042
21198	bex	7.15	7	3	1	face	90	5	control	492	240.042
21199	bex	7.15	7	3	1	face	91	1	face	NA	240.042
21200	bex	7.15	7	3	1	face	92	5	control	526	240.042
21201	bex	7.15	7	3	1	face	93	4	control	635	240.042
21203	bex	7.15	7	3	1	face	95	3	control	525	240.042
21204	bex	7.15	7	3	1	face	96	5	control	513	240.042
21205	bex	7.15	7	3	1	face	97	4	control	495	240.042
21206	bex	7.15	7	3	1	face	98	1	face	NA	240.042
21209	bex	7.15	7	3	1	face	101	4	control	NA	240.042
21210	bex	7.15	7	3	1	face	102	1	control	NA	240.042
21211	bex	7.15	7	3	1	face	103	3	control	566	240.042
21213	bex	7.15	7	3	1	face	105	3	control	529	240.042
21214	bex	7.15	7	3	1	face	106	3	control	524	240.042
21215	bex	7.15	7	3	1	face	107	4	control	689	240.042
21216	bex	7.15	7	3	1	face	108	2	face	NA	240.042
21217	bex	7.15	7	3	1	face	109	3	control	535	240.042
21218	bex	7.15	7	3	1	face	110	4	face	NA	240.042
21222	bex	7.15	7	3	1	face	114	2	control	670	240.042
21224	bex	7.15	7	3	1	face	116	2	control	576	240.042
21226	bex	7.15	7	3	1	face	118	2	control	517	240.042
21227	bex	7.15	7	3	1	face	119	5	control	538	240.042
21228	bex	7.15	7	3	1	face	120	5	face	NA	240.042
21229	bex	7.15	7	3	1	face	121	6	control	447	240.042
21230	bex	7.15	7	3	1	face	122	2	face	NA	240.042
21231	bex	7.15	7	3	1	face	123	3	control	554	240.042
21232	bex	7.15	7	3	1	face	124	4	control	481	240.042
21234	bex	7.15	7	3	1	face	126	4	control	518	240.042
21235	bex	7.15	7	3	1	face	127	1	control	566	240.042

21237	bex	7.15	7	3	1	face	129	6	control	407	240.042
21239	bex	7.15	7	3	1	face	131	5	control	445	240.042
21240	bex	7.15	7	3	1	face	132	2	control	617	240.042
21241	bex	7.15	7	3	1	face	133	2	control	538	240.042
21242	bex	7.15	7	3	1	face	134	3	control	545	240.042
21317	cas	7.72	1	3	1	face	75	4	control	1274	430.331
21318	cas	7.72	1	3	1	face	76	1	control	604	430.331
21356	cas	7.72	1	3	1	face	114	6	control	518	430.331
21358	cas	7.72	1	3	1	face	116	3	control	627	430.331
21469	cas	7.72	1	3	1	face	227	5	control	NA	430.331
21471	cas	7.72	1	3	1	face	229	5	control	783	430.331
21563	cas	7.72	1	3	1	face	321	4	control	576	430.331
21564	cas	7.72	1	3	1	face	322	4	control	886	430.331
21578	cas	7.72	1	3	1	face	336	2	control	524	430.331
21612	cas	7.72	1	3	1	face	370	4	control	912	430.331
21615	cas	7.72	1	3	1	face	373	5	control	941	430.331
21616	cas	7.72	1	3	1	face	374	5	control	638	430.331
21630	cas	7.72	1	3	1	face	388	1	control	596	430.331
21632	cas	7.72	1	3	1	face	390	6	control	1056	430.331
21633	cas	7.72	1	3	1	face	391	3	control	779	430.331
21636	cas	7.72	1	3	1	face	394	5	control	639	430.331
21639	cas	7.72	1	3	1	face	397	2	face	NA	430.331
21641	cas	7.72	1	3	1	face	399	5	control	1209	430.331
21650	cas	7.72	1	3	1	face	408	5	face	NA	430.331
21654	cas	7.72	1	3	1	face	412	6	control	1224	430.331
21655	cas	7.72	1	3	1	face	413	3	control	1013	430.331
21656	cas	7.72	1	3	1	face	414	2	control	701	430.331
21657	cas	7.72	1	3	1	face	415	1	face	NA	430.331
21660	cas	7.72	1	3	1	face	418	6	control	758	430.331
21663	cas	7.72	1	3	1	face	421	3	face	685	430.331
21665	cas	7.72	1	3	1	face	423	6	control	836	430.331
21666	cas	7.72	1	3	1	face	424	5	control	654	430.331
21668	cas	7.72	1	3	1	face	426	3	face	698	430.331
21675	cas	7.72	1	3	1	face	433	1	control	1125	430.331
21676	cas	7.72	1	3	1	face	434	4	control	694	430.331
21678	cas	7.72	1	3	1	face	436	4	face	556	430.331
21680	cas	7.72	1	3	1	face	438	5	control	718	430.331
21681	cas	7.72	1	3	1	face	439	3	control	665	430.331
21682	cas	7.72	1	3	1	face	440	2	control	825	430.331
21683	cas	7.72	1	3	1	face	441	3	face	NA	430.331
21689	cas	7.72	1	3	1	face	447	5	control	646	430.331
21690	cas	7.72	1	3	1	face	448	3	control	754	430.331
21692	cas	7.72	1	3	1	face	450	2	control	504	430.331
21694	cas	7.72	1	3	1	face	452	5	face	500	430.331
21697	cas	7.72	1	3	1	face	455	2	control	599	430.331
21705	cas	7.72	1	3	1	face	463	1	control	827	430.331
21706	cas	7.72	1	3	1	face	464	4	control	798	430.331
21708	cas	7.72	1	3	1	face	466	5	face	NA	430.331
21711	cas	7.72	1	3	1	face	469	6	control	830	430.331
21712	cas	7.72	1	3	1	face	470	4	face	1002	430.331

21714	cas	7.72	1	3	1 face	472	4	control	540	430.331
21720	cas	7.72	1	3	1 face	478	6	control	720	430.331
21728	cas	7.72	1	3	1 face	486	2	face	699	430.331
21730	cas	7.72	1	3	1 face	488	6	control	555	430.331
21737	cas	7.72	1	3	1 face	495	5	control	570	430.331
21739	cas	7.72	1	3	1 face	497	4	control	882	430.331
21740	cas	7.72	1	3	1 face	498	2	control	634	430.331
21742	cas	7.72	1	3	1 face	500	1	control	807	430.331
21743	cas	7.72	1	3	1 face	501	1	control	494	430.331
21744	cas	7.72	1	3	1 face	502	1	control	735	430.331
21751	cas	7.72	1	3	1 face	509	4	control	608	430.331
21754	cas	7.72	1	3	1 face	512	2	control	NA	430.331
21771	cas	7.72	1	3	1 face	529	1	control	711	430.331
21774	cas	7.72	1	3	1 face	532	4	control	527	430.331
21781	cas	7.72	1	3	1 face	539	6	face	624	430.331
21786	cas	7.72	1	3	1 face	544	3	control	1887	430.331
21787	cas	7.72	1	3	1 face	545	6	face	781	430.331
21788	cas	7.72	1	3	1 face	546	5	control	651	430.331
21808	cas	7.72	1	3	1 face	566	6	control	612	430.331
21818	cas	7.72	1	3	1 face	576	6	control	594	430.331
21834	cas	7.72	1	3	1 face	592	4	face	653	430.331
21838	cas	7.72	1	3	1 face	596	4	control	642	430.331
21850	cas	7.72	1	3	1 face	608	2	control	579	430.331
21879	cas	7.72	1	3	1 face	637	2	control	NA	430.331
21884	cas	7.72	1	3	1 face	642	3	control	580	430.331
21886	cas	7.72	1	3	1 face	644	6	control	664	430.331
21900	cas	7.72	1	3	1 face	658	5	control	831	430.331
21902	cas	7.72	1	3	1 face	660	5	control	629	430.331
21904	cas	7.72	1	3	1 face	662	2	control	705	430.331
21905	cas	7.72	1	3	1 face	663	6	control	596	430.331
21906	cas	7.72	1	3	1 face	664	3	control	535	430.331
21907	cas	7.72	1	3	1 face	665	2	face	NA	430.331
21920	cas	7.72	1	3	1 face	678	3	control	665	430.331
21921	cas	7.72	1	3	1 face	679	1	control	658	430.331
21924	cas	7.72	1	3	1 face	682	6	control	699	430.331
21931	cas	7.72	1	3	1 face	689	3	control	611	430.331
21936	cas	7.72	1	3	1 face	694	2	control	838	430.331
21937	cas	7.72	1	3	1 face	695	1	face	898	430.331
21938	cas	7.72	1	3	1 face	696	1	control	638	430.331
21941	cas	7.72	1	3	1 face	699	3	control	NA	430.331
21943	cas	7.72	1	3	1 face	701	1	control	715	430.331
21945	cas	7.72	1	3	1 face	703	4	control	586	430.331
21946	cas	7.72	1	3	1 face	704	6	face	635	430.331
21949	cas	7.72	1	3	1 face	707	3	control	1015	430.331
21950	cas	7.72	1	3	1 face	708	1	control	588	430.331
21953	cas	7.72	1	3	1 face	711	1	face	1057	430.331
21954	cas	7.72	1	3	1 face	712	3	control	1005	430.331
21956	cas	7.72	1	3	1 face	714	1	control	760	430.331
21960	cas	7.72	1	3	1 face	718	2	control	474	430.331
21961	cas	7.72	1	3	1 face	719	4	control	NA	430.331

21964	cas	7.72	1	3	1	face	722	2	control	644	430.331
21965	cas	7.72	1	3	1	face	723	5	control	760	430.331
21968	cas	7.72	1	3	1	face	726	3	control	1245	430.331
21969	cas	7.72	1	3	1	face	727	4	control	1148	430.331
21970	cas	7.72	1	3	1	face	728	4	control	507	430.331
23117	der	7.15	7	3	1	face	1	3	control	1604	479.861
23118	der	7.15	7	3	1	face	2	2	control	796	479.861
23119	der	7.15	7	3	1	face	3	6	control	614	479.861
23120	der	7.15	7	3	1	face	4	3	control	563	479.861
23122	der	7.15	7	3	1	face	6	6	control	421	479.861
23123	der	7.15	7	3	1	face	7	5	control	398	479.861
23125	der	7.15	7	3	1	face	9	3	control	451	479.861
23126	der	7.15	7	3	1	face	10	2	control	490	479.861
23128	der	7.15	7	3	1	face	12	3	control	493	479.861
23130	der	7.15	7	3	1	face	14	5	control	546	479.861
23131	der	7.15	7	3	1	face	15	6	control	593	479.861
23133	der	7.15	7	3	1	face	17	6	control	593	479.861
23135	der	7.15	7	3	1	face	19	6	control	967	479.861
23136	der	7.15	7	3	1	face	20	1	control	465	479.861
23137	der	7.15	7	3	1	face	21	3	control	646	479.861
23139	der	7.15	7	3	1	face	23	4	control	451	479.861
23140	der	7.15	7	3	1	face	24	2	control	513	479.861
23141	der	7.15	7	3	1	face	25	2	face	NA	479.861
23145	der	7.15	7	3	1	face	29	6	control	621	479.861
23146	der	7.15	7	3	1	face	30	5	control	668	479.861
23147	der	7.15	7	3	1	face	31	5	face	NA	479.861
23148	der	7.15	7	3	1	face	32	1	control	819	479.861
23150	der	7.15	7	3	1	face	34	1	control	562	479.861
23151	der	7.15	7	3	1	face	35	5	face	NA	479.861
23152	der	7.15	7	3	1	face	36	5	control	570	479.861
23153	der	7.15	7	3	1	face	37	1	control	612	479.861
23154	der	7.15	7	3	1	face	38	6	control	934	479.861
23155	der	7.15	7	3	1	face	39	3	face	NA	479.861
23156	der	7.15	7	3	1	face	40	3	control	685	479.861
23157	der	7.15	7	3	1	face	41	1	face	NA	479.861
23158	der	7.15	7	3	1	face	42	5	control	509	479.861
23159	der	7.15	7	3	1	face	43	3	control	2491	479.861
23160	der	7.15	7	3	1	face	44	1	control	2178	479.861
23161	der	7.15	7	3	1	face	45	1	face	2329	479.861
23162	der	7.15	7	3	1	face	46	2	control	1139	479.861
23163	der	7.15	7	3	1	face	47	4	control	485	479.861
23164	der	7.15	7	3	1	face	48	4	control	482	479.861
23165	der	7.15	7	3	1	face	49	6	face	1723	479.861
23166	der	7.15	7	3	1	face	50	3	control	2311	479.861
23167	der	7.15	7	3	1	face	51	2	control	646	479.861
23168	der	7.15	7	3	1	face	52	1	face	NA	479.861
23169	der	7.15	7	3	1	face	53	4	control	523	479.861
23170	der	7.15	7	3	1	face	54	6	control	1682	479.861
23171	der	7.15	7	3	1	face	55	3	face	NA	479.861
23172	der	7.15	7	3	1	face	56	5	control	878	479.861

23173 der	7.15	7	3	1 face	57	3 control	723	479.861
23175 der	7.15	7	3	1 face	59	2 control	626	479.861
23176 der	7.15	7	3	1 face	60	1 control	580	479.861
23177 der	7.15	7	3	1 face	61	1 control	666	479.861
23179 der	7.15	7	3	1 face	63	4 control	452	479.861
23180 der	7.15	7	3	1 face	64	2 face	NA	479.861
23181 der	7.15	7	3	1 face	65	6 control	479	479.861
23183 der	7.15	7	3	1 face	67	6 control	802	479.861
23185 der	7.15	7	3	1 face	69	5 control	643	479.861
23186 der	7.15	7	3	1 face	70	3 control	625	479.861
23187 der	7.15	7	3	1 face	71	4 face	661	479.861
23188 der	7.15	7	3	1 face	72	2 control	611	479.861
23189 der	7.15	7	3	1 face	73	5 control	944	479.861
23191 der	7.15	7	3	1 face	75	5 control	572	479.861
23192 der	7.15	7	3	1 face	76	3 control	635	479.861
23193 der	7.15	7	3	1 face	77	4 control	556	479.861
23194 der	7.15	7	3	1 face	78	2 face	1546	479.861
23196 der	7.15	7	3	1 face	80	1 control	522	479.861
23197 der	7.15	7	3	1 face	81	4 face	542	479.861
23198 der	7.15	7	3	1 face	82	6 control	559	479.861
23199 der	7.15	7	3	1 face	83	2 control	531	479.861
23201 der	7.15	7	3	1 face	85	6 control	2576	479.861
23202 der	7.15	7	3	1 face	86	6 control	905	479.861
23203 der	7.15	7	3	1 face	87	4 control	525	479.861
23204 der	7.15	7	3	1 face	88	4 face	525	479.861
23205 der	7.15	7	3	1 face	89	4 control	550	479.861
23207 der	7.15	7	3	1 face	91	6 control	626	479.861
23208 der	7.15	7	3	1 face	92	4 control	586	479.861
23210 der	7.15	7	3	1 face	94	4 control	488	479.861
23211 der	7.15	7	3	1 face	95	1 control	608	479.861
23212 der	7.15	7	3	1 face	96	2 control	577	479.861
23214 der	7.15	7	3	1 face	98	2 control	619	479.861
23215 der	7.15	7	3	1 face	99	1 control	510	479.861
23216 der	7.15	7	3	1 face	100	2 control	583	479.861
23218 der	7.15	7	3	1 face	102	5 control	541	479.861
23220 der	7.15	7	3	1 face	104	2 control	494	479.861
23221 der	7.15	7	3	1 face	105	2 control	696	479.861
23223 der	7.15	7	3	1 face	107	5 control	469	479.861
23224 der	7.15	7	3	1 face	108	1 control	562	479.861
23226 der	7.15	7	3	1 face	110	1 control	648	479.861
23227 der	7.15	7	3	1 face	111	6 face	1030	479.861
23228 der	7.15	7	3	1 face	112	4 control	640	479.861
23229 der	7.15	7	3	1 face	113	5 control	464	479.861
23230 der	7.15	7	3	1 face	114	3 control	486	479.861
23231 der	7.15	7	3	1 face	115	5 face	415	479.861
23232 der	7.15	7	3	1 face	116	3 control	680	479.861
23233 der	7.15	7	3	1 face	117	4 control	523	479.861
23234 der	7.15	7	3	1 face	118	6 face	505	479.861
23235 der	7.15	7	3	1 face	119	3 control	523	479.861
23236 der	7.15	7	3	1 face	120	3 face	476	479.861

23237	der	7.15	7	3	1	face	121	4	control	503	479.861
23240	der	7.15	7	3	1	face	124	2	control	595	479.861
23241	der	7.15	7	3	1	face	125	3	control	512	479.861
23245	der	7.15	7	3	1	face	129	1	control	429	479.861
23247	der	7.15	7	3	1	face	131	2	control	1013	479.861
23248	elm	7.12	7	3	1	face	1	2	control	636	290.101
23249	elm	7.12	7	3	1	face	2	5	control	390	290.101
23250	elm	7.12	7	3	1	face	3	2	control	497	290.101
23252	elm	7.12	7	3	1	face	5	1	control	561	290.101
23253	elm	7.12	7	3	1	face	6	6	control	514	290.101
23268	elm	7.12	7	3	1	face	21	5	control	NA	290.101
23269	elm	7.12	7	3	1	face	22	6	control	525	290.101
23270	elm	7.12	7	3	1	face	23	1	control	2103	290.101
23271	elm	7.12	7	3	1	face	24	4	face	1952	290.101
23272	elm	7.12	7	3	1	face	25	4	control	805	290.101
23274	elm	7.12	7	3	1	face	27	4	control	629	290.101
23275	elm	7.12	7	3	1	face	28	5	control	610	290.101
23276	elm	7.12	7	3	1	face	29	6	control	581	290.101
23278	elm	7.12	7	3	1	face	31	1	control	563	290.101
23279	elm	7.12	7	3	1	face	32	4	control	513	290.101
23280	elm	7.12	7	3	1	face	33	2	control	563	290.101
23281	elm	7.12	7	3	1	face	34	1	face	795	290.101
23282	elm	7.12	7	3	1	face	35	5	control	565	290.101
23283	elm	7.12	7	3	1	face	36	2	face	690	290.101
23284	elm	7.12	7	3	1	face	37	1	control	721	290.101
23285	elm	7.12	7	3	1	face	38	1	face	634	290.101
23286	elm	7.12	7	3	1	face	39	3	control	NA	290.101
23287	elm	7.12	7	3	1	face	40	4	control	601	290.101
23296	elm	7.12	7	3	1	face	49	3	control	729	290.101
23299	elm	7.12	7	3	1	face	52	6	control	543	290.101
23300	elm	7.12	7	3	1	face	53	2	control	520	290.101
23302	elm	7.12	7	3	1	face	55	5	control	735	290.101
23314	elm	7.12	7	3	1	face	67	6	control	491	290.101
23315	elm	7.12	7	3	1	face	68	2	control	499	290.101
23316	elm	7.12	7	3	1	face	69	6	face	515	290.101
23317	elm	7.12	7	3	1	face	70	4	control	590	290.101
23318	elm	7.12	7	3	1	face	71	3	control	531	290.101
23319	elm	7.12	7	3	1	face	72	6	control	476	290.101
23321	elm	7.12	7	3	1	face	74	6	control	475	290.101
23327	elm	7.12	7	3	1	face	80	2	control	1760	290.101
23328	elm	7.12	7	3	1	face	81	1	control	577	290.101
23330	elm	7.12	7	3	1	face	83	4	control	496	290.101
23331	elm	7.12	7	3	1	face	84	5	control	653	290.101
23336	elm	7.12	7	3	1	face	89	3	face	569	290.101
23337	elm	7.12	7	3	1	face	90	4	control	536	290.101
23338	elm	7.12	7	3	1	face	91	5	face	657	290.101
23340	elm	7.12	7	3	1	face	93	4	face	519	290.101
23341	elm	7.12	7	3	1	face	94	3	control	543	290.101
23342	elm	7.12	7	3	1	face	95	1	control	759	290.101
23344	elm	7.12	7	3	1	face	97	1	control	523	290.101

23353	elm	7.12	7	3	1	face	106	2	control	NA	290.101
23354	elm	7.12	7	3	1	face	107	4	control	509	290.101
23355	elm	7.12	7	3	1	face	108	3	face	673	290.101
23356	elm	7.12	7	3	1	face	109	5	control	518	290.101
23358	elm	7.12	7	3	1	face	111	2	control	571	290.101
23359	elm	7.12	7	3	1	face	112	6	control	472	290.101
23361	elm	7.12	7	3	1	face	114	1	control	576	290.101
23363	elm	7.12	7	3	1	face	116	3	control	505	290.101
23371	elm	7.12	7	3	1	face	124	1	control	605	290.101
23372	elm	7.12	7	3	1	face	125	5	control	442	290.101
23373	elm	7.12	7	3	1	face	126	5	face	475	290.101
23374	elm	7.12	7	3	1	face	127	2	control	479	290.101
23375	elm	7.12	7	3	1	face	128	3	control	532	290.101
23376	elm	7.12	7	3	1	face	129	5	face	367	290.101
23377	elm	7.12	7	3	1	face	130	3	control	463	290.101
23378	elm	7.12	7	3	1	face	131	5	control	455	290.101
23379	elm	7.12	7	3	1	face	132	3	control	507	290.101
23381	elm	7.12	7	3	1	face	134	4	control	577	290.101
23382	elm	7.12	7	3	1	face	135	1	control	581	290.101
23383	elm	7.12	7	3	1	face	136	3	control	566	290.101
23384	elm	7.12	7	3	1	face	137	2	face	634	290.101
23385	elm	7.12	7	3	1	face	138	6	control	479	290.101
23390	elm	7.12	7	3	1	face	143	5	control	497	290.101
23391	elm	7.12	7	3	1	face	144	2	face	592	290.101
23392	elm	7.12	7	3	1	face	145	6	control	741	290.101
23393	elm	7.12	7	3	1	face	146	2	control	448	290.101
23395	elm	7.12	7	3	1	face	148	1	control	488	290.101
23396	elm	7.12	7	3	1	face	149	2	control	491	290.101
23405	elm	7.12	7	3	1	face	158	6	face	368	290.101
23406	elm	7.12	7	3	1	face	159	3	control	1678	290.101
23407	elm	7.12	7	3	1	face	160	3	control	490	290.101
23409	elm	7.12	7	3	1	face	162	4	control	767	290.101
23417	elm	7.12	7	3	1	face	170	2	control	2256	290.101
23418	elm	7.12	7	3	1	face	171	4	face	552	290.101
23419	elm	7.12	7	3	1	face	172	2	control	424	290.101
23421	elm	7.12	7	3	1	face	174	5	control	560	290.101
23423	elm	7.12	7	3	1	face	176	6	control	433	290.101
23424	elm	7.12	7	3	1	face	177	3	face	512	290.101
23425	elm	7.12	7	3	1	face	178	6	control	452	290.101
23426	elm	7.12	7	3	1	face	179	3	control	520	290.101
23437	elm	7.12	7	3	1	face	190	1	face	542	290.101
23438	elm	7.12	7	3	1	face	191	1	control	495	290.101
23439	elm	7.12	7	3	1	face	192	2	control	501	290.101
23441	elm	7.12	7	3	1	face	194	6	control	546	290.101
23443	elm	7.12	7	3	1	face	196	4	control	437	290.101
23444	elm	7.12	7	3	1	face	197	4	control	483	290.101
23446	elm	7.12	7	3	1	face	199	1	control	484	290.101
23447	elm	7.12	7	3	1	face	200	6	face	525	290.101
23448	elm	7.12	7	3	1	face	201	3	control	574	290.101
23449	elm	7.12	7	3	1	face	202	3	control	493	290.101

23451	elm	7.12	7	3	1	face	204	1	control	587	290.101
23452	elm	7.12	7	3	1	face	205	2	control	509	290.101
23453	elm	7.12	7	3	1	face	206	6	control	549	290.101
23460	fla	8.84	1	3	1	face	7	2	control	641	138.603
23461	fla	8.84	1	3	1	face	8	5	control	488	138.603
23462	fla	8.84	1	3	1	face	9	5	control	1048	138.603
23494	fla	8.84	1	3	1	face	41	4	control	681	138.603
23495	fla	8.84	1	3	1	face	42	6	control	707	138.603
23496	fla	8.84	1	3	1	face	43	5	control	597	138.603
23510	fla	8.84	1	3	1	face	57	1	control	621	138.603
23511	fla	8.84	1	3	1	face	58	1	control	665	138.603
23513	fla	8.84	1	3	1	face	60	3	control	675	138.603
23519	fla	8.84	1	3	1	face	66	1	control	590	138.603
23520	fla	8.84	1	3	1	face	67	3	control	634	138.603
23521	fla	8.84	1	3	1	face	68	2	face	1256	138.603
23522	fla	8.84	1	3	1	face	69	1	control	662	138.603
23524	fla	8.84	1	3	1	face	71	3	control	649	138.603
23525	fla	8.84	1	3	1	face	72	5	control	528	138.603
23526	fla	8.84	1	3	1	face	73	6	control	583	138.603
23527	fla	8.84	1	3	1	face	74	5	face	1193	138.603
23528	fla	8.84	1	3	1	face	75	3	control	626	138.603
23529	fla	8.84	1	3	1	face	76	5	control	538	138.603
23530	fla	8.84	1	3	1	face	77	6	control	647	138.603
23531	fla	8.84	1	3	1	face	78	3	face	818	138.603
23539	fla	8.84	1	3	1	face	86	3	control	626	138.603
23540	fla	8.84	1	3	1	face	87	2	control	714	138.603
23541	fla	8.84	1	3	1	face	88	1	face	651	138.603
23542	fla	8.84	1	3	1	face	89	1	control	613	138.603
23543	fla	8.84	1	3	1	face	90	3	control	625	138.603
23552	fla	8.84	1	3	1	face	99	1	control	601	138.603
23554	fla	8.84	1	3	1	face	101	4	control	575	138.603
23555	fla	8.84	1	3	1	face	102	3	control	574	138.603
23571	fla	8.84	1	3	1	face	118	2	control	627	138.603
23573	fla	8.84	1	3	1	face	120	4	control	623	138.603
23574	fla	8.84	1	3	1	face	121	1	face	665	138.603
23575	fla	8.84	1	3	1	face	122	2	control	606	138.603
23577	fla	8.84	1	3	1	face	124	1	control	594	138.603
23578	fla	8.84	1	3	1	face	125	1	control	589	138.603
23579	fla	8.84	1	3	1	face	126	4	control	528	138.603
23581	fla	8.84	1	3	1	face	128	2	control	586	138.603
23595	fla	8.84	1	3	1	face	142	5	control	572	138.603
23597	fla	8.84	1	3	1	face	144	6	control	578	138.603
23598	fla	8.84	1	3	1	face	145	4	control	605	138.603
23611	fla	8.84	1	3	1	face	158	2	control	576	138.603
23613	fla	8.84	1	3	1	face	160	6	control	516	138.603
23622	fla	8.84	1	3	1	face	169	5	face	595	138.603
23623	fla	8.84	1	3	1	face	170	5	control	628	138.603
23637	fla	8.84	1	3	1	face	184	3	face	660	138.603
23638	fla	8.84	1	3	1	face	185	4	control	574	138.603
23639	fla	8.84	1	3	1	face	186	4	control	532	138.603

23663 fla	8.84	1	3	1 face	210	3 control	558	138.603
23665 fla	8.84	1	3	1 face	212	6 control	560	138.603
23666 fla	8.84	1	3	1 face	213	6 control	494	138.603
23667 fla	8.84	1	3	1 face	214	2 face	665	138.603
23668 fla	8.84	1	3	1 face	215	2 control	564	138.603
23696 fla	8.84	1	3	1 face	243	1 control	739	138.603
23697 fla	8.84	1	3	1 face	244	6 control	540	138.603
23705 fla	8.84	1	3	1 face	252	5 control	520	138.603
23712 fla	8.84	1	3	1 face	259	2 face	560	138.603
23713 fla	8.84	1	3	1 face	260	6 control	529	138.603
23714 fla	8.84	1	3	1 face	261	3 control	600	138.603
23715 fla	8.84	1	3	1 face	262	5 control	525	138.603
23726 fla	8.84	1	3	1 face	273	6 face	559	138.603
23727 fla	8.84	1	3	1 face	274	1 control	573	138.603
23728 fla	8.84	1	3	1 face	275	2 control	523	138.603
23730 fla	8.84	1	3	1 face	277	5 control	503	138.603
23743 fla	8.84	1	3	1 face	290	2 control	516	138.603
23744 fla	8.84	1	3	1 face	291	5 face	1365	138.603
23745 fla	8.84	1	3	1 face	292	6 control	573	138.603
23754 fla	8.84	1	3	1 face	301	4 control	493	138.603
23755 fla	8.84	1	3	1 face	302	6 face	636	138.603
23756 fla	8.84	1	3	1 face	303	4 control	525	138.603
23757 fla	8.84	1	3	1 face	304	1 control	536	138.603
23759 fla	8.84	1	3	1 face	306	5 control	510	138.603
23761 fla	8.84	1	3	1 face	308	2 control	841	138.603
23766 fla	8.84	1	3	1 face	313	6 control	503	138.603
23767 fla	8.84	1	3	1 face	314	3 face	590	138.603
23768 fla	8.84	1	3	1 face	315	4 control	515	138.603
23774 fla	8.84	1	3	1 face	321	2 control	522	138.603
23776 fla	8.84	1	3	1 face	323	2 control	575	138.603
23777 fla	8.84	1	3	1 face	324	6 control	558	138.603
23778 fla	8.84	1	3	1 face	325	5 control	527	138.603
23779 fla	8.84	1	3	1 face	326	1 face	710	138.603
23780 fla	8.84	1	3	1 face	327	4 control	678	138.603
23781 fla	8.84	1	3	1 face	328	3 control	656	138.603
23782 fla	8.84	1	3	1 face	329	3 control	549	138.603
23783 fla	8.84	1	3	1 face	330	4 face	510	138.603
23784 fla	8.84	1	3	1 face	331	6 control	523	138.603
23785 fla	8.84	1	3	1 face	332	2 control	641	138.603
23786 fla	8.84	1	3	1 face	333	6 face	662	138.603
23787 fla	8.84	1	3	1 face	334	3 control	540	138.603
23788 fla	8.84	1	3	1 face	335	5 control	680	138.603
23796 fla	8.84	1	3	1 face	343	1 control	574	138.603
23798 fla	8.84	1	3	1 face	345	4 control	880	138.603
23799 fla	8.84	1	3	1 face	346	4 control	568	138.603
23800 fla	8.84	1	3	1 face	347	4 face	613	138.603
23801 fla	8.84	1	3	1 face	348	5 control	499	138.603
23802 fla	8.84	1	3	1 face	349	4 face	555	138.603
23803 fla	8.84	1	3	1 face	350	2 control	586	138.603
23808 fla	8.84	1	3	1 face	355	4 control	556	138.603

23812	fla	8.84	1	3	1	face	359	4	control	561	138.603
23813	fla	8.84	1	3	1	face	360	1	control	696	138.603
23814	fla	8.84	1	3	1	face	361	3	control	720	138.603
23816	gro	8.58	7	3	1	face	2	6	control	470	169.929
23817	gro	8.58	7	3	1	face	3	5	control	488	169.929
23818	gro	8.58	7	3	1	face	4	6	control	474	169.929
23819	gro	8.58	7	3	1	face	5	6	control	497	169.929
23821	gro	8.58	7	3	1	face	7	1	control	1041	169.929
23822	gro	8.58	7	3	1	face	8	5	control	732	169.929
23823	gro	8.58	7	3	1	face	9	1	control	716	169.929
23824	gro	8.58	7	3	1	face	10	2	face	NA	169.929
23825	gro	8.58	7	3	1	face	11	1	control	681	169.929
23826	gro	8.58	7	3	1	face	12	3	control	605	169.929
23827	gro	8.58	7	3	1	face	13	5	control	840	169.929
23829	gro	8.58	7	3	1	face	15	4	control	957	169.929
23830	gro	8.58	7	3	1	face	16	6	face	568	169.929
23831	gro	8.58	7	3	1	face	17	3	control	2044	169.929
23832	gro	8.58	7	3	1	face	18	5	face	1257	169.929
23833	gro	8.58	7	3	1	face	19	6	control	575	169.929
23834	gro	8.58	7	3	1	face	20	1	control	599	169.929
23835	gro	8.58	7	3	1	face	21	3	face	678	169.929
23836	gro	8.58	7	3	1	face	22	4	control	758	169.929
23838	gro	8.58	7	3	1	face	24	2	control	626	169.929
23839	gro	8.58	7	3	1	face	25	6	control	2346	169.929
23840	gro	8.58	7	3	1	face	26	4	face	744	169.929
23841	gro	8.58	7	3	1	face	27	3	control	613	169.929
23843	gro	8.58	7	3	1	face	29	1	control	637	169.929
23844	gro	8.58	7	3	1	face	30	2	face	930	169.929
23845	gro	8.58	7	3	1	face	31	2	control	643	169.929
23846	gro	8.58	7	3	1	face	32	3	control	1678	169.929
23847	gro	8.58	7	3	1	face	33	6	control	2034	169.929
23849	gro	8.58	7	3	1	face	35	6	control	764	169.929
23850	gro	8.58	7	3	1	face	36	3	control	587	169.929
23851	gro	8.58	7	3	1	face	37	5	control	663	169.929
23852	gro	8.58	7	3	1	face	38	5	face	578	169.929
23854	gro	8.58	7	3	1	face	40	1	control	2666	169.929
23855	gro	8.58	7	3	1	face	41	4	control	690	169.929
23856	gro	8.58	7	3	1	face	42	1	face	1000	169.929
23857	gro	8.58	7	3	1	face	43	6	control	1342	169.929
23858	gro	8.58	7	3	1	face	44	2	control	498	169.929
23859	gro	8.58	7	3	1	face	45	5	control	592	169.929
23860	gro	8.58	7	3	1	face	46	3	face	896	169.929
23861	gro	8.58	7	3	1	face	47	5	control	625	169.929
23862	gro	8.58	7	3	1	face	48	5	control	861	169.929
23863	gro	8.58	7	3	1	face	49	1	control	762	169.929
23865	gro	8.58	7	3	1	face	51	3	control	1014	169.929
23866	gro	8.58	7	3	1	face	52	5	control	614	169.929
23867	gro	8.58	7	3	1	face	53	6	control	1063	169.929
23869	gro	8.58	7	3	1	face	55	6	control	1137	169.929
23870	gro	8.58	7	3	1	face	56	3	control	597	169.929

23872 gro	8.58	7	3	1 face	58	2 control	681	169.929
23873 gro	8.58	7	3	1 face	59	6 control	558	169.929
23874 gro	8.58	7	3	1 face	60	2 control	758	169.929
23875 gro	8.58	7	3	1 face	61	1 face	1015	169.929
23876 gro	8.58	7	3	1 face	62	5 control	1425	169.929
23879 gro	8.58	7	3	1 face	65	2 control	NA	169.929
23881 gro	8.58	7	3	1 face	67	4 control	736	169.929
23882 gro	8.58	7	3	1 face	68	4 control	1131	169.929
23883 gro	8.58	7	3	1 face	69	1 control	897	169.929
23885 gro	8.58	7	3	1 face	71	2 control	609	169.929
23886 gro	8.58	7	3	1 face	72	2 control	736	169.929
23887 gro	8.58	7	3	1 face	73	6 face	1568	169.929
23888 gro	8.58	7	3	1 face	74	6 control	1449	169.929
23889 gro	8.58	7	3	1 face	75	1 control	587	169.929
23890 gro	8.58	7	3	1 face	76	5 control	722	169.929
23891 gro	8.58	7	3	1 face	77	4 face	1384	169.929
23892 gro	8.58	7	3	1 face	78	4 control	728	169.929
23894 gro	8.58	7	3	1 face	80	1 control	1073	169.929
23895 gro	8.58	7	3	1 face	81	4 control	578	169.929
23897 gro	8.58	7	3	1 face	83	4 control	963	169.929
23898 gro	8.58	7	3	1 face	84	3 control	836	169.929
23900 gro	8.58	7	3	1 face	86	3 control	563	169.929
23901 gro	8.58	7	3	1 face	87	4 control	737	169.929
23903 gro	8.58	7	3	1 face	89	5 control	1034	169.929
23905 gro	8.58	7	3	1 face	91	2 control	1269	169.929
23906 gro	8.58	7	3	1 face	92	1 control	NA	169.929
23907 gro	8.58	7	3	1 face	93	1 control	1061	169.929
23908 gro	8.58	7	3	1 face	94	6 face	1358	169.929
23909 gro	8.58	7	3	1 face	95	3 control	898	169.929
23910 gro	8.58	7	3	1 face	96	2 control	609	169.929
23912 gro	8.58	7	3	1 face	98	5 control	866	169.929
23914 gro	8.58	7	3	1 face	100	5 control	978	169.929
23915 gro	8.58	7	3	1 face	101	1 face	1183	169.929
23916 gro	8.58	7	3	1 face	102	2 control	895	169.929
23922 gro	8.58	7	3	1 face	108	6 control	1464	169.929
23923 gro	8.58	7	3	1 face	109	5 face	826	169.929
23924 gro	8.58	7	3	1 face	110	6 control	547	169.929
23925 gro	8.58	7	3	1 face	111	3 control	672	169.929
23926 gro	8.58	7	3	1 face	112	4 control	652	169.929
23934 gro	8.58	7	3	1 face	120	6 control	1863	169.929
23935 gro	8.58	7	3	1 face	121	4 control	1326	169.929
23936 gro	8.58	7	3	1 face	122	3 control	839	169.929
23937 gro	8.58	7	3	1 face	123	2 face	1081	169.929
23938 gro	8.58	7	3	1 face	124	1 control	656	169.929
23952 gro	8.58	7	3	1 face	138	2 control	1401	169.929
23953 gro	8.58	7	3	1 face	139	3 face	1015	169.929
23954 gro	8.58	7	3	1 face	140	4 control	1139	169.929
23955 gro	8.58	7	3	1 face	141	4 face	646	169.929
23956 gro	8.58	7	3	1 face	142	4 control	1318	169.929
23958 gro	8.58	7	3	1 face	144	6 control	533	169.929

23959	gro	8.58	7	3	1 face	145	6 control	641	169.929
23960	gro	8.58	7	3	1 face	146	3 control	670	169.929
23961	gro	8.58	7	3	1 face	147	3 control	689	169.929
23976	han	8.05	7	3	1 face	15	4 control	511	256.872
24006	han	8.05	7	3	1 face	45	5 control	395	256.872
24020	han	8.05	7	3	1 face	59	1 control	376	256.872
24022	han	8.05	7	3	1 face	61	4 control	388	256.872
24023	han	8.05	7	3	1 face	62	5 control	421	256.872
24024	han	8.05	7	3	1 face	63	1 control	345	256.872
24025	han	8.05	7	3	1 face	64	2 control	499	256.872
24026	han	8.05	7	3	1 face	65	5 control	555	256.872
24040	han	8.05	7	3	1 face	79	6 control	421	256.872
24045	han	8.05	7	3	1 face	84	4 face	388	256.872
24046	han	8.05	7	3	1 face	85	1 control	442	256.872
24047	han	8.05	7	3	1 face	86	3 control	527	256.872
24057	han	8.05	7	3	1 face	96	2 face	368	256.872
24058	han	8.05	7	3	1 face	97	5 control	445	256.872
24088	han	8.05	7	3	1 face	127	2 control	327	256.872
24114	han	8.05	7	3	1 face	153	5 face	940	256.872
24115	han	8.05	7	3	1 face	154	4 control	475	256.872
24117	han	8.05	7	3	1 face	156	6 face	415	256.872
24118	han	8.05	7	3	1 face	157	4 control	458	256.872
24119	han	8.05	7	3	1 face	158	6 control	444	256.872
24125	han	8.05	7	3	1 face	164	5 control	480	256.872
24128	han	8.05	7	3	1 face	167	6 face	715	256.872
24132	han	8.05	7	3	1 face	171	4 control	536	256.872
24133	han	8.05	7	3	1 face	172	4 control	372	256.872
24140	han	8.05	7	3	1 face	179	4 control	482	256.872
24141	han	8.05	7	3	1 face	180	3 face	543	256.872
24149	han	8.05	7	3	1 face	188	4 control	969	256.872
24150	han	8.05	7	3	1 face	189	5 control	491	256.872
24152	han	8.05	7	3	1 face	191	1 control	382	256.872
24153	han	8.05	7	3	1 face	192	3 face	632	256.872
24160	han	8.05	7	3	1 face	199	3 control	621	256.872
24161	han	8.05	7	3	1 face	200	1 face	591	256.872
24162	han	8.05	7	3	1 face	201	5 control	481	256.872
24163	han	8.05	7	3	1 face	202	2 control	363	256.872
24164	han	8.05	7	3	1 face	203	6 control	403	256.872
24165	han	8.05	7	3	1 face	204	6 face	613	256.872
24172	han	8.05	7	3	1 face	211	6 control	523	256.872
24173	han	8.05	7	3	1 face	212	3 control	926	256.872
24175	han	8.05	7	3	1 face	214	6 control	304	256.872
24180	han	8.05	7	3	1 face	219	3 control	855	256.872
24181	han	8.05	7	3	1 face	220	5 control	464	256.872
24192	han	8.05	7	3	1 face	231	6 control	459	256.872
24193	han	8.05	7	3	1 face	232	2 face	455	256.872
24194	han	8.05	7	3	1 face	233	2 control	394	256.872
24195	han	8.05	7	3	1 face	234	1 control	437	256.872
24212	han	8.05	7	3	1 face	251	6 control	357	256.872
24214	han	8.05	7	3	1 face	253	4 face	386	256.872

24215	han	8.05	7	3	1	face	254	5	control	345	256.872
24216	han	8.05	7	3	1	face	255	4	control	452	256.872
24217	han	8.05	7	3	1	face	256	6	control	447	256.872
24248	han	8.05	7	3	1	face	287	1	control	371	256.872
24333	han	8.05	7	3	1	face	372	3	control	419	256.872
24334	han	8.05	7	3	1	face	373	4	face	454	256.872
24359	han	8.05	7	3	1	face	398	3	control	513	256.872
24391	han	8.05	7	3	1	face	430	4	control	1884	256.872
24392	han	8.05	7	3	1	face	431	5	face	417	256.872
24393	han	8.05	7	3	1	face	432	1	control	518	256.872
24395	han	8.05	7	3	1	face	434	1	control	418	256.872
24454	han	8.05	7	3	1	face	493	5	control	312	256.872
24487	han	8.05	7	3	1	face	526	5	control	309	256.872
24489	han	8.05	7	3	1	face	528	3	control	417	256.872
24493	han	8.05	7	3	1	face	532	2	face	469	256.872
24494	han	8.05	7	3	1	face	533	6	control	459	256.872
24495	han	8.05	7	3	1	face	534	1	control	727	256.872
24496	han	8.05	7	3	1	face	535	2	control	446	256.872
24498	han	8.05	7	3	1	face	537	4	control	422	256.872
24499	han	8.05	7	3	1	face	538	2	control	381	256.872
24500	han	8.05	7	3	1	face	539	6	control	545	256.872
24502	han	8.05	7	3	1	face	541	1	control	379	256.872
24503	han	8.05	7	3	1	face	542	3	control	402	256.872
24505	han	8.05	7	3	1	face	544	3	control	373	256.872
24506	han	8.05	7	3	1	face	545	2	control	455	256.872
24507	han	8.05	7	3	1	face	546	1	face	476	256.872
24510	han	8.05	7	3	1	face	549	6	control	419	256.872
24512	han	8.05	7	3	1	face	551	5	control	397	256.872
24521	han	8.05	7	3	1	face	560	5	control	473	256.872
24525	han	8.05	7	3	1	face	564	2	control	362	256.872
24530	han	8.05	7	3	1	face	569	6	control	498	256.872
24546	han	8.05	7	3	1	face	585	2	control	472	256.872
24563	han	8.05	7	3	1	face	602	3	control	461	256.872
24574	han	8.05	7	3	1	face	613	2	control	433	256.872
24698	han	8.05	7	3	1	face	737	1	face	508	256.872
24809	han	8.05	7	3	1	face	848	1	control	459	256.872
24810	han	8.05	7	3	1	face	849	2	control	442	256.872
24811	han	8.05	7	3	1	face	850	3	face	362	256.872
24823	han	8.05	7	3	1	face	862	4	control	504	256.872
24859	han	8.05	7	3	1	face	898	1	control	405	256.872
24861	han	8.05	7	3	1	face	900	3	control	493	256.872
24863	han	8.05	7	3	1	face	902	3	control	509	256.872
24864	han	8.05	7	3	1	face	903	4	control	385	256.872
24866	han	8.05	7	3	1	face	905	1	control	420	256.872
24867	han	8.05	7	3	1	face	906	2	control	471	256.872
24868	han	8.05	7	3	1	face	907	4	control	452	256.872
24869	han	8.05	7	3	1	face	908	5	face	347	256.872
24870	han	8.05	7	3	1	face	909	1	control	407	256.872
24871	han	8.05	7	3	1	face	910	2	control	400	256.872
24873	han	8.05	7	3	1	face	912	2	control	387	256.872

24874	han	8.05	7	3	1	face	913	3	control	374	256.872
24875	han	8.05	7	3	1	face	914	2	control	350	256.872
25080	hum	12.03	6	3	1	face	205	6	control	999	257.6
25081	hum	12.03	6	3	1	face	206	4	control	537	257.6
25082	hum	12.03	6	3	1	face	207	3	control	550	257.6
25084	hum	12.03	6	3	1	face	209	6	control	429	257.6
25085	hum	12.03	6	3	1	face	210	1	control	514	257.6
25086	hum	12.03	6	3	1	face	211	2	control	451	257.6
25087	hum	12.03	6	3	1	face	212	6	control	440	257.6
25088	hum	12.03	6	3	1	face	213	3	control	476	257.6
25089	hum	12.03	6	3	1	face	214	2	face	501	257.6
25090	hum	12.03	6	3	1	face	215	3	control	495	257.6
25091	hum	12.03	6	3	1	face	216	5	control	530	257.6
25092	hum	12.03	6	3	1	face	217	6	control	436	257.6
25094	hum	12.03	6	3	1	face	219	6	control	481	257.6
25233	hum	12.03	6	3	1	face	358	2	control	NA	257.6
25234	hum	12.03	6	3	1	face	359	2	control	NA	257.6
25236	hum	12.03	6	3	1	face	361	6	control	466	257.6
25238	hum	12.03	6	3	1	face	363	5	control	726	257.6
25239	hum	12.03	6	3	1	face	364	1	control	604	257.6
25241	hum	12.03	6	3	1	face	366	6	control	415	257.6
25242	hum	12.03	6	3	1	face	367	6	control	478	257.6
25243	hum	12.03	6	3	1	face	368	1	control	503	257.6
25244	hum	12.03	6	3	1	face	369	3	face	551	257.6
25245	hum	12.03	6	3	1	face	370	6	control	438	257.6
25247	hum	12.03	6	3	1	face	372	5	control	NA	257.6
25248	hum	12.03	6	3	1	face	373	3	face	585	257.6
25249	hum	12.03	6	3	1	face	374	1	control	490	257.6
25250	hum	12.03	6	3	1	face	375	5	control	461	257.6
25251	hum	12.03	6	3	1	face	376	5	control	554	257.6
25253	hum	12.03	6	3	1	face	378	3	control	510	257.6
25254	hum	12.03	6	3	1	face	379	4	face	449	257.6
25255	hum	12.03	6	3	1	face	380	1	control	583	257.6
25256	hum	12.03	6	3	1	face	381	6	control	483	257.6
25257	hum	12.03	6	3	1	face	382	5	face	585	257.6
25258	hum	12.03	6	3	1	face	383	1	control	506	257.6
25259	hum	12.03	6	3	1	face	384	2	face	522	257.6
25260	hum	12.03	6	3	1	face	385	5	control	444	257.6
25271	hum	12.03	6	3	1	face	396	4	control	NA	257.6
25272	hum	12.03	6	3	1	face	397	2	control	954	257.6
25273	hum	12.03	6	3	1	face	398	3	control	398	257.6
25274	hum	12.03	6	3	1	face	399	6	face	413	257.6
25279	hum	12.03	6	3	1	face	404	4	control	722	257.6
25281	hum	12.03	6	3	1	face	406	2	control	466	257.6
25282	hum	12.03	6	3	1	face	407	6	face	417	257.6
25283	hum	12.03	6	3	1	face	408	4	control	476	257.6
25284	hum	12.03	6	3	1	face	409	2	control	516	257.6
25286	hum	12.03	6	3	1	face	411	4	control	498	257.6
25287	hum	12.03	6	3	1	face	412	1	control	461	257.6
25288	hum	12.03	6	3	1	face	413	2	control	486	257.6

25290	hum	12.03	6	3	1	face	415	6 control	459	257.6
25291	hum	12.03	6	3	1	face	416	4 control	517	257.6
25293	hum	12.03	6	3	1	face	418	4 control	469	257.6
25294	hum	12.03	6	3	1	face	419	3 control	481	257.6
25296	hum	12.03	6	3	1	face	421	3 control	469	257.6
25297	hum	12.03	6	3	1	face	422	4 control	545	257.6
25298	hum	12.03	6	3	1	face	423	4 control	513	257.6
25299	hum	12.03	6	3	1	face	424	5 face	452	257.6
25300	hum	12.03	6	3	1	face	425	1 control	473	257.6
25301	hum	12.03	6	3	1	face	426	4 control	514	257.6
25302	hum	12.03	6	3	1	face	427	6 face	424	257.6
25303	hum	12.03	6	3	1	face	428	5 control	461	257.6
25304	hum	12.03	6	3	1	face	429	1 control	494	257.6
25305	hum	12.03	6	3	1	face	430	4 face	471	257.6
25306	hum	12.03	6	3	1	face	431	6 control	412	257.6
25307	hum	12.03	6	3	1	face	432	3 control	464	257.6
25309	hum	12.03	6	3	1	face	434	1 control	508	257.6
25310	hum	12.03	6	3	1	face	435	5 control	390	257.6
25311	hum	12.03	6	3	1	face	436	1 face	452	257.6
25312	hum	12.03	6	3	1	face	437	2 control	471	257.6
25313	hum	12.03	6	3	1	face	438	4 control	448	257.6
25314	hum	12.03	6	3	1	face	439	5 control	469	257.6
25315	hum	12.03	6	3	1	face	440	1 face	638	257.6
25324	hum	12.03	6	3	1	face	449	5 control	801	257.6
25325	hum	12.03	6	3	1	face	450	4 control	487	257.6
25326	hum	12.03	6	3	1	face	451	3 control	492	257.6
25327	hum	12.03	6	3	1	face	452	4 face	486	257.6
25328	hum	12.03	6	3	1	face	453	5 control	515	257.6
25330	hum	12.03	6	3	1	face	455	1 control	492	257.6
25332	hum	12.03	6	3	1	face	457	2 control	442	257.6
25348	hum	12.03	6	3	1	face	473	1 face	900	257.6
25349	hum	12.03	6	3	1	face	474	2 control	706	257.6
25350	hum	12.03	6	3	1	face	475	5 control	434	257.6
25351	hum	12.03	6	3	1	face	476	2 face	553	257.6
25352	hum	12.03	6	3	1	face	477	3 control	480	257.6
25353	hum	12.03	6	3	1	face	478	5 face	436	257.6
25354	hum	12.03	6	3	1	face	479	6 control	442	257.6
25355	hum	12.03	6	3	1	face	480	6 control	447	257.6
25356	hum	12.03	6	3	1	face	481	3 control	477	257.6
25358	hum	12.03	6	3	1	face	483	3 control	476	257.6
25360	hum	12.03	6	3	1	face	485	2 control	498	257.6
25380	hum	12.03	6	3	1	face	505	1 control	824	257.6
25382	hum	12.03	6	3	1	face	507	1 control	502	257.6
25384	hum	12.03	6	3	1	face	509	2 control	450	257.6
25385	hum	12.03	6	3	1	face	510	4 control	423	257.6
25387	hum	12.03	6	3	1	face	512	3 control	410	257.6
25388	hum	12.03	6	3	1	face	513	3 face	478	257.6
25389	hum	12.03	6	3	1	face	514	1 control	455	257.6
25391	hum	12.03	6	3	1	face	516	5 control	404	257.6
25392	hum	12.03	6	3	1	face	517	1 control	509	257.6

25393	hum	12.03	6	3	1	face	518	5	control	398	257.6
25394	hum	12.03	6	3	1	face	519	3	control	453	257.6
25395	kas	12.18	7	3	1	face	1	3	control	585	479.6
25396	kas	12.18	7	3	1	face	2	1	control	737	479.6
25404	kas	12.18	7	3	1	face	10	3	control	922	479.6
25406	kas	12.18	7	3	1	face	12	3	control	568	479.6
25420	kas	12.18	7	3	1	face	26	1	control	1659	479.6
25462	kas	12.18	7	3	1	face	68	5	control	NA	479.6
25463	kas	12.18	7	3	1	face	69	2	control	670	479.6
25481	kas	12.18	7	3	1	face	87	1	control	713	479.6
25491	kas	12.18	7	3	1	face	97	4	control	576	479.6
25555	kas	12.18	7	3	1	face	161	3	face	NA	479.6
25556	kas	12.18	7	3	1	face	162	4	control	547	479.6
25557	kas	12.18	7	3	1	face	163	6	control	574	479.6
25574	kas	12.18	7	3	1	face	180	2	control	580	479.6
25605	kas	12.18	7	3	1	face	211	6	face	691	479.6
25606	kas	12.18	7	3	1	face	212	3	control	665	479.6
25670	kas	12.18	7	3	1	face	276	3	control	NA	479.6
25755	kas	12.18	7	3	1	face	361	5	control	579	479.6
25764	kas	12.18	7	3	1	face	370	1	control	1180	479.6
25780	kas	12.18	7	3	1	face	386	3	control	715	479.6
25782	kas	12.18	7	3	1	face	388	1	control	753	479.6
25783	kas	12.18	7	3	1	face	389	1	control	637	479.6
25784	kas	12.18	7	3	1	face	390	2	control	644	479.6
25786	kas	12.18	7	3	1	face	392	6	control	657	479.6
25787	kas	12.18	7	3	1	face	393	4	face	641	479.6
25788	kas	12.18	7	3	1	face	394	2	control	1173	479.6
25789	kas	12.18	7	3	1	face	395	4	control	698	479.6
25881	kas	12.18	7	3	1	face	487	3	control	651	479.6
25882	kas	12.18	7	3	1	face	488	5	face	577	479.6
25883	kas	12.18	7	3	1	face	489	5	control	548	479.6
25884	kas	12.18	7	3	1	face	490	6	control	624	479.6
25885	kas	12.18	7	3	1	face	491	6	control	556	479.6
25887	kas	12.18	7	3	1	face	493	3	control	725	479.6
25888	kas	12.18	7	3	1	face	494	4	control	599	479.6
25889	kas	12.18	7	3	1	face	495	6	control	495	479.6
25890	kas	12.18	7	3	1	face	496	1	face	633	479.6
25891	kas	12.18	7	3	1	face	497	6	control	559	479.6
25893	kas	12.18	7	3	1	face	499	4	control	564	479.6
25894	kas	12.18	7	3	1	face	500	5	control	514	479.6
25895	kas	12.18	7	3	1	face	501	1	face	600	479.6
25896	kas	12.18	7	3	1	face	502	1	control	650	479.6
25897	kas	12.18	7	3	1	face	503	2	control	595	479.6
25899	kas	12.18	7	3	1	face	505	5	control	501	479.6
25900	kas	12.18	7	3	1	face	506	3	control	659	479.6
25901	kas	12.18	7	3	1	face	507	4	control	520	479.6
25903	kas	12.18	7	3	1	face	509	1	control	620	479.6
25904	kas	12.18	7	3	1	face	510	6	control	552	479.6
25905	kas	12.18	7	3	1	face	511	5	control	505	479.6
25914	kas	12.18	7	3	1	face	520	5	control	535	479.6

26030	kas	12.18	7	3	1 face	636	2 control	499	479.6
26032	kas	12.18	7	3	1 face	638	6 control	525	479.6
26033	kas	12.18	7	3	1 face	639	5 control	474	479.6
26034	kas	12.18	7	3	1 face	640	5 face	482	479.6
26035	kas	12.18	7	3	1 face	641	2 control	1226	479.6
26036	kas	12.18	7	3	1 face	642	2 control	522	479.6
26037	kas	12.18	7	3	1 face	643	1 control	616	479.6
26038	kas	12.18	7	3	1 face	644	3 face	1064	479.6
26039	kas	12.18	7	3	1 face	645	4 control	552	479.6
26040	kas	12.18	7	3	1 face	646	5 control	452	479.6
26041	kas	12.18	7	3	1 face	647	2 face	605	479.6
26042	kas	12.18	7	3	1 face	648	5 control	494	479.6
26065	kas	12.18	7	3	1 face	671	1 control	548	479.6
26066	kas	12.18	7	3	1 face	672	4 control	501	479.6
26079	kas	12.18	7	3	1 face	685	1 control	625	479.6
26080	kas	12.18	7	3	1 face	686	1 control	502	479.6
26081	kas	12.18	7	3	1 face	687	6 face	432	479.6
26082	kas	12.18	7	3	1 face	688	1 control	546	479.6
26083	kas	12.18	7	3	1 face	689	1 face	554	479.6
26084	kas	12.18	7	3	1 face	690	6 control	468	479.6
26085	kas	12.18	7	3	1 face	691	3 face	536	479.6
26086	kas	12.18	7	3	1 face	692	3 control	542	479.6
26088	kas	12.18	7	3	1 face	694	4 control	532	479.6
26089	kas	12.18	7	3	1 face	695	5 control	561	479.6
26090	kas	12.18	7	3	1 face	696	4 face	621	479.6
26103	kas	12.18	7	3	1 face	709	3 control	699	479.6
26104	kas	12.18	7	3	1 face	710	3 control	537	479.6
26106	kas	12.18	7	3	1 face	712	4 control	562	479.6
26107	kas	12.18	7	3	1 face	713	6 face	514	479.6
26108	kas	12.18	7	3	1 face	714	2 control	566	479.6
26109	kas	12.18	7	3	1 face	715	3 control	669	479.6
26110	kas	12.18	7	3	1 face	716	1 control	602	479.6
26157	kas	12.18	7	3	1 face	763	5 control	NA	479.6
26158	kas	12.18	7	3	1 face	764	2 control	509	479.6
26159	kas	12.18	7	3	1 face	765	4 control	520	479.6
26164	kas	12.18	7	3	1 face	770	4 face	467	479.6
26165	kas	12.18	7	3	1 face	771	3 control	558	479.6
26249	kas	12.18	7	3	1 face	855	6 control	513	479.6
26314	kas	12.18	7	3	1 face	920	4 control	530	479.6
26315	kas	12.18	7	3	1 face	921	2 face	719	479.6
26316	kas	12.18	7	3	1 face	922	3 control	1025	479.6
26317	kas	12.18	7	3	1 face	923	2 face	460	479.6
26318	kas	12.18	7	3	1 face	924	6 control	422	479.6
26320	kas	12.18	7	3	1 face	926	2 control	484	479.6
26321	kas	12.18	7	3	1 face	927	6 control	737	479.6
26322	kas	12.18	7	3	1 face	928	2 control	528	479.6
26323	kas	12.18	7	3	1 face	929	5 face	439	479.6
26396	kas	12.18	7	3	1 face	1002	4 control	456	479.6
26398	kas	12.18	7	3	1 face	1004	1 control	617	479.6
26552	kas	12.18	7	3	1 face	1158	1 control	537	479.6

26553	kas	12.18	7	3	1	face	1159	2	control	455	479.6
26554	kas	12.18	7	3	1	face	1160	6	control	432	479.6
26590	lou	7.01	2	3	1	face	36	5	control	NA	357.091
26591	lou	7.01	2	3	1	face	37	2	control	741	357.091
26592	lou	7.01	2	3	1	face	38	3	control	528	357.091
26653	lou	7.01	2	3	1	face	99	6	control	NA	357.091
26670	lou	7.01	2	3	1	face	116	6	control	582	357.091
26688	lou	7.01	2	3	1	face	134	3	control	NA	357.091
26722	lou	7.01	2	3	1	face	168	1	control	561	357.091
26723	lou	7.01	2	3	1	face	169	1	face	NA	357.091
26724	lou	7.01	2	3	1	face	170	1	control	577	357.091
26725	lou	7.01	2	3	1	face	171	2	control	477	357.091
26726	lou	7.01	2	3	1	face	172	4	control	547	357.091
26728	lou	7.01	2	3	1	face	174	5	control	489	357.091
26740	lou	7.01	2	3	1	face	186	4	control	703	357.091
26751	lou	7.01	2	3	1	face	197	3	control	529	357.091
26753	lou	7.01	2	3	1	face	199	1	control	614	357.091
26754	lou	7.01	2	3	1	face	200	2	control	473	357.091
26770	lou	7.01	2	3	1	face	216	2	face	NA	357.091
26771	lou	7.01	2	3	1	face	217	3	control	696	357.091
26772	lou	7.01	2	3	1	face	218	3	control	477	357.091
26774	lou	7.01	2	3	1	face	220	5	control	457	357.091
26784	lou	7.01	2	3	1	face	230	5	control	463	357.091
26785	lou	7.01	2	3	1	face	231	6	control	511	357.091
26795	lou	7.01	2	3	1	face	241	6	control	610	357.091
26803	lou	7.01	2	3	1	face	249	5	control	NA	357.091
26813	lou	7.01	2	3	1	face	259	2	control	834	357.091
26821	lou	7.01	2	3	1	face	267	3	face	NA	357.091
26822	lou	7.01	2	3	1	face	268	1	control	NA	357.091
26843	lou	7.01	2	3	1	face	289	5	control	599	357.091
26855	lou	7.01	2	3	1	face	301	5	control	2199	357.091
26856	lou	7.01	2	3	1	face	302	1	control	775	357.091
26876	lou	7.01	2	3	1	face	322	4	control	593	357.091
26933	lou	7.01	2	3	1	face	379	6	control	605	357.091
26955	lou	7.01	2	3	1	face	401	6	control	NA	357.091
26978	lou	7.01	2	3	1	face	424	2	control	547	357.091
26979	lou	7.01	2	3	1	face	425	4	face	843	357.091
26980	lou	7.01	2	3	1	face	426	4	control	631	357.091
26992	lou	7.01	2	3	1	face	438	2	control	620	357.091
26993	lou	7.01	2	3	1	face	439	3	face	627	357.091
26994	lou	7.01	2	3	1	face	440	6	control	453	357.091
26996	lou	7.01	2	3	1	face	442	2	control	550	357.091
27007	lou	7.01	2	3	1	face	453	4	control	510	357.091
27008	lou	7.01	2	3	1	face	454	4	control	558	357.091
27022	lou	7.01	2	3	1	face	468	6	control	529	357.091
27023	lou	7.01	2	3	1	face	469	3	control	491	357.091
27035	lou	7.01	2	3	1	face	481	6	control	457	357.091
27036	lou	7.01	2	3	1	face	482	6	face	647	357.091
27037	lou	7.01	2	3	1	face	483	2	control	639	357.091
27038	lou	7.01	2	3	1	face	484	5	control	481	357.091

27050	lou	7.01	2	3	1	face	496	6	face	624	357.091
27074	lou	7.01	2	3	1	face	520	6	control	445	357.091
27093	lou	7.01	2	3	1	face	539	2	control	564	357.091
27110	lou	7.01	2	3	1	face	556	3	control	546	357.091
27138	lou	7.01	2	3	1	face	584	3	control	462	357.091
27142	lou	7.01	2	3	1	face	588	4	control	694	357.091
27147	lou	7.01	2	3	1	face	593	2	control	479	357.091
27154	lou	7.01	2	3	1	face	600	4	control	463	357.091
27179	lou	7.01	2	3	1	face	625	1	face	607	357.091
27180	lou	7.01	2	3	1	face	626	3	control	537	357.091
27181	lou	7.01	2	3	1	face	627	6	control	532	357.091
27182	lou	7.01	2	3	1	face	628	1	control	NA	357.091
27183	lou	7.01	2	3	1	face	629	3	face	868	357.091
27184	lou	7.01	2	3	1	face	630	1	control	548	357.091
27192	lou	7.01	2	3	1	face	638	6	face	621	357.091
27204	lou	7.01	2	3	1	face	650	6	control	993	357.091
27226	lou	7.01	2	3	1	face	672	6	control	487	357.091
27271	lou	7.01	2	3	1	face	717	5	control	465	357.091
27293	lou	7.01	2	3	1	face	739	3	control	1123	357.091
27294	lou	7.01	2	3	1	face	740	2	face	NA	357.091
27295	lou	7.01	2	3	1	face	741	5	control	515	357.091
27306	lou	7.01	2	3	1	face	752	6	control	493	357.091
27307	lou	7.01	2	3	1	face	753	1	control	590	357.091
27334	lou	7.01	2	3	1	face	780	3	control	NA	357.091
27374	lou	7.01	2	3	1	face	820	4	face	NA	357.091
27375	lou	7.01	2	3	1	face	821	4	control	589	357.091
27376	lou	7.01	2	3	1	face	822	1	control	1336	357.091
27387	lou	7.01	2	3	1	face	833	4	control	578	357.091
27408	lou	7.01	2	3	1	face	854	2	face	NA	357.091
27418	lou	7.01	2	3	1	face	864	4	control	616	357.091
27420	lou	7.01	2	3	1	face	866	3	control	545	357.091
27434	lou	7.01	2	3	1	face	880	2	control	505	357.091
27446	lou	7.01	2	3	1	face	892	5	face	617	357.091
27456	lou	7.01	2	3	1	face	902	5	control	481	357.091
27457	lou	7.01	2	3	1	face	903	2	control	667	357.091
27462	lou	7.01	2	3	1	face	908	5	control	553	357.091
27463	lou	7.01	2	3	1	face	909	5	control	523	357.091
27506	lou	7.01	2	3	1	face	952	5	face	732	357.091
27524	lou	7.01	2	3	1	face	970	3	control	697	357.091
27526	lou	7.01	2	3	1	face	972	1	face	1397	357.091
27527	lou	7.01	2	3	1	face	973	1	control	525	357.091
27535	lou	7.01	2	3	1	face	981	1	control	577	357.091
27536	lou	7.01	2	3	1	face	982	4	face	793	357.091
27537	lou	7.01	2	3	1	face	983	2	control	457	357.091
27538	lou	7.01	2	3	1	face	984	4	control	578	357.091
27543	lou	7.01	2	3	1	face	989	1	control	NA	357.091
27544	lou	7.01	2	3	1	face	990	5	face	576	357.091
27545	lou	7.01	2	3	1	face	991	3	control	491	357.091
27547	lou	7.01	2	3	1	face	993	5	control	511	357.091
27548	lou	7.01	2	3	1	face	994	5	control	420	357.091

27549	lou	7.01	2	3	1 face	995	3 control	485	357.091
27552	lou	7.01	2	3	1 face	998	5 control	494	357.091
27554	nor	7.95	5	3	1 face	2	5 control	401	366.55
27563	nor	7.95	5	3	1 face	11	1 control	459	366.55
27573	nor	7.95	5	3	1 face	21	4 control	360	366.55
27583	nor	7.95	5	3	1 face	31	5 control	878	366.55
27595	nor	7.95	5	3	1 face	43	1 control	432	366.55
27596	nor	7.95	5	3	1 face	44	1 control	452	366.55
27612	nor	7.95	5	3	1 face	60	3 control	545	366.55
27655	nor	7.95	5	3	1 face	103	5 control	385	366.55
27657	nor	7.95	5	3	1 face	105	6 control	NA	366.55
27658	nor	7.95	5	3	1 face	106	5 control	312	366.55
27694	nor	7.95	5	3	1 face	142	4 control	330	366.55
27696	nor	7.95	5	3	1 face	144	3 control	478	366.55
27697	nor	7.95	5	3	1 face	145	3 face	524	366.55
27698	nor	7.95	5	3	1 face	146	1 control	467	366.55
27699	nor	7.95	5	3	1 face	147	3 control	408	366.55
27700	nor	7.95	5	3	1 face	148	4 face	430	366.55
27713	nor	7.95	5	3	1 face	161	5 face	NA	366.55
27714	nor	7.95	5	3	1 face	162	1 control	351	366.55
27715	nor	7.95	5	3	1 face	163	1 face	568	366.55
27747	nor	7.95	5	3	1 face	195	1 control	565	366.55
27748	nor	7.95	5	3	1 face	196	6 control	371	366.55
27752	nor	7.95	5	3	1 face	200	5 control	345	366.55
27753	nor	7.95	5	3	1 face	201	2 control	381	366.55
27777	nor	7.95	5	3	1 face	225	6 control	394	366.55
27785	nor	7.95	5	3	1 face	233	5 control	368	366.55
27786	nor	7.95	5	3	1 face	234	6 control	578	366.55
27788	nor	7.95	5	3	1 face	236	1 control	374	366.55
27792	nor	7.95	5	3	1 face	240	2 control	911	366.55
27798	nor	7.95	5	3	1 face	246	3 control	460	366.55
27801	nor	7.95	5	3	1 face	249	3 control	NA	366.55
27805	nor	7.95	5	3	1 face	253	2 face	905	366.55
27806	nor	7.95	5	3	1 face	254	3 control	425	366.55
27808	nor	7.95	5	3	1 face	256	2 control	442	366.55
27809	nor	7.95	5	3	1 face	257	4 face	438	366.55
27831	nor	7.95	5	3	1 face	279	1 control	397	366.55
27833	nor	7.95	5	3	1 face	281	1 control	399	366.55
27851	nor	7.95	5	3	1 face	299	4 control	461	366.55
27870	nor	7.95	5	3	1 face	318	1 control	328	366.55
27884	nor	7.95	5	3	1 face	332	1 face	533	366.55
27885	nor	7.95	5	3	1 face	333	6 control	561	366.55
27886	nor	7.95	5	3	1 face	334	4 control	417	366.55
27887	nor	7.95	5	3	1 face	335	2 face	400	366.55
27888	nor	7.95	5	3	1 face	336	3 control	1098	366.55
27889	nor	7.95	5	3	1 face	337	5 control	423	366.55
27891	nor	7.95	5	3	1 face	339	2 control	383	366.55
27892	nor	7.95	5	3	1 face	340	6 control	374	366.55
27897	nor	7.95	5	3	1 face	345	5 control	328	366.55
27904	nor	7.95	5	3	1 face	352	3 control	375	366.55

27905	nor	7.95	5	3	1	face	353	1	control	381	366.55
27906	nor	7.95	5	3	1	face	354	3	face	498	366.55
27914	nor	7.95	5	3	1	face	362	6	control	639	366.55
27915	nor	7.95	5	3	1	face	363	6	control	416	366.55
27934	nor	7.95	5	3	1	face	382	2	control	391	366.55
27935	nor	7.95	5	3	1	face	383	3	control	576	366.55
27936	nor	7.95	5	3	1	face	384	5	control	1290	366.55
27938	nor	7.95	5	3	1	face	386	2	control	437	366.55
27939	nor	7.95	5	3	1	face	387	2	control	414	366.55
27947	nor	7.95	5	3	1	face	395	2	control	357	366.55
27948	nor	7.95	5	3	1	face	396	6	face	465	366.55
27949	nor	7.95	5	3	1	face	397	2	control	495	366.55
27965	nor	7.95	5	3	1	face	413	6	face	404	366.55
27966	nor	7.95	5	3	1	face	414	4	control	353	366.55
27967	nor	7.95	5	3	1	face	415	5	control	370	366.55
27995	nor	7.95	5	3	1	face	443	6	control	965	366.55
27996	nor	7.95	5	3	1	face	444	5	control	418	366.55
27997	nor	7.95	5	3	1	face	445	5	control	399	366.55
27998	nor	7.95	5	3	1	face	446	1	face	479	366.55
27999	nor	7.95	5	3	1	face	447	4	control	353	366.55
28008	nor	7.95	5	3	1	face	456	3	control	414	366.55
28009	nor	7.95	5	3	1	face	457	2	face	1251	366.55
28010	nor	7.95	5	3	1	face	458	2	control	431	366.55
28011	nor	7.95	5	3	1	face	459	1	control	442	366.55
28012	nor	7.95	5	3	1	face	460	6	face	378	366.55
28013	nor	7.95	5	3	1	face	461	2	control	358	366.55
28014	nor	7.95	5	3	1	face	462	5	control	367	366.55
28021	nor	7.95	5	3	1	face	469	6	control	537	366.55
28038	nor	7.95	5	3	1	face	486	4	face	398	366.55
28061	nor	7.95	5	3	1	face	509	4	control	308	366.55
28062	nor	7.95	5	3	1	face	510	4	control	314	366.55
28063	nor	7.95	5	3	1	face	511	4	control	338	366.55
28065	nor	7.95	5	3	1	face	513	6	control	479	366.55
28066	nor	7.95	5	3	1	face	514	4	control	370	366.55
28068	nor	7.95	5	3	1	face	516	6	control	472	366.55
28091	nor	7.95	5	3	1	face	539	1	control	877	366.55
28092	nor	7.95	5	3	1	face	540	5	control	330	366.55
28093	nor	7.95	5	3	1	face	541	5	face	369	366.55
28098	nor	7.95	5	3	1	face	546	3	control	380	366.55
28099	nor	7.95	5	3	1	face	547	1	control	405	366.55
28100	nor	7.95	5	3	1	face	548	5	face	401	366.55
28101	nor	7.95	5	3	1	face	549	4	control	404	366.55
28102	nor	7.95	5	3	1	face	550	2	control	386	366.55
28103	nor	7.95	5	3	1	face	551	3	control	858	366.55
28104	nor	7.95	5	3	1	face	552	3	face	471	366.55
28113	nor	7.95	5	3	1	face	561	4	control	373	366.55
28117	nor	7.95	5	3	1	face	565	5	control	438	366.55
28141	nor	7.95	5	3	1	face	589	2	control	423	366.55
28142	nor	7.95	5	3	1	face	590	5	control	361	366.55
28143	nor	7.95	5	3	1	face	591	6	control	379	366.55

28144	pac	6.92	1	3	1	face	1	4	control	NA	309.77
28146	pac	6.92	1	3	1	face	3	4	control	351	309.77
28147	pac	6.92	1	3	1	face	4	4	control	335	309.77
28163	pac	6.92	1	3	1	face	20	5	control	536	309.77
28164	pac	6.92	1	3	1	face	21	3	control	431	309.77
28165	pac	6.92	1	3	1	face	22	3	control	596	309.77
28170	pac	6.92	1	3	1	face	27	3	face	376	309.77
28171	pac	6.92	1	3	1	face	28	6	control	398	309.77
28172	pac	6.92	1	3	1	face	29	2	face	428	309.77
28173	pac	6.92	1	3	1	face	30	6	control	359	309.77
28174	pac	6.92	1	3	1	face	31	2	control	434	309.77
28176	pac	6.92	1	3	1	face	33	6	control	346	309.77
28178	pac	6.92	1	3	1	face	35	2	control	398	309.77
28179	pac	6.92	1	3	1	face	36	4	control	399	309.77
28184	pac	6.92	1	3	1	face	41	3	control	1846	309.77
28185	pac	6.92	1	3	1	face	42	1	face	493	309.77
28186	pac	6.92	1	3	1	face	43	3	control	599	309.77
28187	pac	6.92	1	3	1	face	44	4	control	383	309.77
28188	pac	6.92	1	3	1	face	45	4	control	460	309.77
28189	pac	6.92	1	3	1	face	46	4	face	536	309.77
28190	pac	6.92	1	3	1	face	47	2	control	652	309.77
28191	pac	6.92	1	3	1	face	48	2	control	354	309.77
28192	pac	6.92	1	3	1	face	49	4	control	1318	309.77
28194	pac	6.92	1	3	1	face	51	6	control	364	309.77
28199	pac	6.92	1	3	1	face	56	5	control	1435	309.77
28200	pac	6.92	1	3	1	face	57	1	control	879	309.77
28201	pac	6.92	1	3	1	face	58	1	face	381	309.77
28202	pac	6.92	1	3	1	face	59	2	control	431	309.77
28203	pac	6.92	1	3	1	face	60	2	control	352	309.77
28204	pac	6.92	1	3	1	face	61	4	face	386	309.77
28205	pac	6.92	1	3	1	face	62	2	control	439	309.77
28206	pac	6.92	1	3	1	face	63	6	control	531	309.77
28208	pac	6.92	1	3	1	face	65	5	control	457	309.77
28209	pac	6.92	1	3	1	face	66	5	control	797	309.77
28216	pac	6.92	1	3	1	face	73	1	control	469	309.77
28217	pac	6.92	1	3	1	face	74	2	control	471	309.77
28218	pac	6.92	1	3	1	face	75	1	control	440	309.77
28219	pac	6.92	1	3	1	face	76	3	face	402	309.77
28220	pac	6.92	1	3	1	face	77	5	control	1415	309.77
28221	pac	6.92	1	3	1	face	78	4	control	445	309.77
28222	pac	6.92	1	3	1	face	79	3	control	410	309.77
28227	pac	6.92	1	3	1	face	84	3	control	510	309.77
28229	pac	6.92	1	3	1	face	86	3	control	379	309.77
28230	pac	6.92	1	3	1	face	87	6	control	478	309.77
28232	pac	6.92	1	3	1	face	89	1	control	379	309.77
28233	pac	6.92	1	3	1	face	90	4	control	312	309.77
28234	pac	6.92	1	3	1	face	91	5	control	381	309.77
28235	pac	6.92	1	3	1	face	92	6	face	436	309.77
28236	pac	6.92	1	3	1	face	93	4	control	370	309.77
28244	pac	6.92	1	3	1	face	101	5	face	413	309.77

28245	pac	6.92	1	3	1	face	102	4	control	428	309.77
28246	pac	6.92	1	3	1	face	103	1	face	475	309.77
28247	pac	6.92	1	3	1	face	104	5	control	371	309.77
28248	pac	6.92	1	3	1	face	105	6	face	702	309.77
28249	pac	6.92	1	3	1	face	106	4	control	337	309.77
28262	pac	6.92	1	3	1	face	119	6	control	NA	309.77
28263	pac	6.92	1	3	1	face	120	3	face	528	309.77
28264	pac	6.92	1	3	1	face	121	1	control	537	309.77
28265	pac	6.92	1	3	1	face	122	4	face	771	309.77
28266	pac	6.92	1	3	1	face	123	1	control	400	309.77
28267	pac	6.92	1	3	1	face	124	3	control	358	309.77
28268	pac	6.92	1	3	1	face	125	5	face	1295	309.77
28269	pac	6.92	1	3	1	face	126	5	control	639	309.77
28270	pac	6.92	1	3	1	face	127	1	control	637	309.77
28277	pac	6.92	1	3	1	face	134	3	control	390	309.77
28305	pac	6.92	1	3	1	face	162	6	control	354	309.77
28306	pac	6.92	1	3	1	face	163	1	control	539	309.77
28323	pac	6.92	1	3	1	face	180	1	control	321	309.77
28324	pac	6.92	1	3	1	face	181	2	face	322	309.77
28325	pac	6.92	1	3	1	face	182	5	control	480	309.77
28327	pac	6.92	1	3	1	face	184	3	control	558	309.77
28342	pac	6.92	1	3	1	face	199	6	control	413	309.77
28343	pac	6.92	1	3	1	face	200	2	control	340	309.77
28352	pac	6.92	1	3	1	face	209	2	control	400	309.77
28354	pac	6.92	1	3	1	face	211	4	control	352	309.77
28355	pac	6.92	1	3	1	face	212	1	control	377	309.77
28356	pac	6.92	1	3	1	face	213	6	control	494	309.77
28378	pac	6.92	1	3	1	face	235	3	control	841	309.77
28379	pac	6.92	1	3	1	face	236	5	control	423	309.77
28381	pac	6.92	1	3	1	face	238	3	control	720	309.77
28382	pac	6.92	1	3	1	face	239	2	control	852	309.77
28384	pac	6.92	1	3	1	face	241	3	control	364	309.77
28385	pac	6.92	1	3	1	face	242	5	control	322	309.77
28386	pac	6.92	1	3	1	face	243	6	face	326	309.77
28387	pac	6.92	1	3	1	face	244	4	control	351	309.77
28394	pac	6.92	1	3	1	face	251	4	control	380	309.77
28395	pac	6.92	1	3	1	face	252	5	control	328	309.77
28396	pac	6.92	1	3	1	face	253	2	control	367	309.77
28397	pac	6.92	1	3	1	face	254	5	face	309	309.77
28426	pac	6.92	1	3	1	face	283	5	control	411	309.77
28427	pac	6.92	1	3	1	face	284	6	control	503	309.77
28428	pac	6.92	1	3	1	face	285	2	face	672	309.77
28429	pac	6.92	1	3	1	face	286	1	control	389	309.77
28430	pac	6.92	1	3	1	face	287	1	control	385	309.77
28431	pac	6.92	1	3	1	face	288	6	control	359	309.77
28433	pac	6.92	1	3	1	face	290	4	control	433	309.77
28435	pac	6.92	1	3	1	face	292	2	control	365	309.77
28437	pac	6.92	1	3	1	face	294	2	control	333	309.77
28445	pac	6.92	1	3	1	face	302	5	control	NA	309.77
28447	rio	6.21	2	3	1	face	2	1	control	443	275.339

28450	rio	6.21	2	3	1	face	5	2	control	377	275.339
28451	rio	6.21	2	3	1	face	6	3	control	354	275.339
28452	rio	6.21	2	3	1	face	7	3	control	343	275.339
28457	rio	6.21	2	3	1	face	12	3	control	320	275.339
28460	rio	6.21	2	3	1	face	15	2	control	351	275.339
28461	rio	6.21	2	3	1	face	16	3	face	467	275.339
28467	rio	6.21	2	3	1	face	22	4	control	458	275.339
28468	rio	6.21	2	3	1	face	23	3	control	516	275.339
28469	rio	6.21	2	3	1	face	24	6	control	359	275.339
28471	rio	6.21	2	3	1	face	26	5	control	351	275.339
28472	rio	6.21	2	3	1	face	27	5	control	318	275.339
28479	rio	6.21	2	3	1	face	34	5	control	512	275.339
28487	rio	6.21	2	3	1	face	42	2	face	520	275.339
28490	rio	6.21	2	3	1	face	45	1	control	484	275.339
28492	rio	6.21	2	3	1	face	47	4	control	352	275.339
28493	rio	6.21	2	3	1	face	48	4	control	351	275.339
28505	rio	6.21	2	3	1	face	60	6	control	545	275.339
28506	rio	6.21	2	3	1	face	61	6	face	406	275.339
28507	rio	6.21	2	3	1	face	62	6	control	379	275.339
28508	rio	6.21	2	3	1	face	63	2	control	375	275.339
28509	rio	6.21	2	3	1	face	64	2	face	849	275.339
28516	rio	6.21	2	3	1	face	71	3	control	375	275.339
28517	rio	6.21	2	3	1	face	72	2	control	381	275.339
28518	rio	6.21	2	3	1	face	73	3	control	442	275.339
28527	rio	6.21	2	3	1	face	82	3	face	NA	275.339
28533	rio	6.21	2	3	1	face	88	1	control	587	275.339
28535	rio	6.21	2	3	1	face	90	5	control	405	275.339
28536	rio	6.21	2	3	1	face	91	3	control	515	275.339
28538	rio	6.21	2	3	1	face	93	1	control	424	275.339
28540	rio	6.21	2	3	1	face	95	4	control	374	275.339
28541	rio	6.21	2	3	1	face	96	6	control	374	275.339
28542	rio	6.21	2	3	1	face	97	3	face	1251	275.339
28543	rio	6.21	2	3	1	face	98	3	control	407	275.339
28544	rio	6.21	2	3	1	face	99	3	control	385	275.339
28546	rio	6.21	2	3	1	face	101	6	control	350	275.339
28549	rio	6.21	2	3	1	face	104	1	control	NA	275.339
28550	rio	6.21	2	3	1	face	105	5	control	360	275.339
28558	rio	6.21	2	3	1	face	113	6	face	779	275.339
28559	rio	6.21	2	3	1	face	114	5	control	363	275.339
28564	rio	6.21	2	3	1	face	119	2	face	549	275.339
28565	rio	6.21	2	3	1	face	120	3	control	445	275.339
28566	rio	6.21	2	3	1	face	121	3	control	411	275.339
28567	rio	6.21	2	3	1	face	122	2	control	433	275.339
28569	rio	6.21	2	3	1	face	124	5	control	409	275.339
28570	rio	6.21	2	3	1	face	125	1	control	477	275.339
28573	rio	6.21	2	3	1	face	128	1	control	494	275.339
28574	rio	6.21	2	3	1	face	129	2	control	405	275.339
28601	rio	6.21	2	3	1	face	156	3	control	435	275.339
28603	rio	6.21	2	3	1	face	158	1	control	477	275.339
28604	rio	6.21	2	3	1	face	159	1	control	621	275.339

28605	rio	6.21	2	3	1	face	160	5	face	1285	275.339
28606	rio	6.21	2	3	1	face	161	6	control	450	275.339
28608	rio	6.21	2	3	1	face	163	2	control	NA	275.339
28609	rio	6.21	2	3	1	face	164	5	control	442	275.339
28610	rio	6.21	2	3	1	face	165	1	control	762	275.339
28612	rio	6.21	2	3	1	face	167	1	control	446	275.339
28613	rio	6.21	2	3	1	face	168	6	control	485	275.339
28614	rio	6.21	2	3	1	face	169	1	control	527	275.339
28615	rio	6.21	2	3	1	face	170	5	face	1152	275.339
28617	rio	6.21	2	3	1	face	172	5	control	574	275.339
28618	rio	6.21	2	3	1	face	173	4	control	477	275.339
28624	rio	6.21	2	3	1	face	179	2	control	386	275.339
28625	rio	6.21	2	3	1	face	180	4	control	461	275.339
28626	rio	6.21	2	3	1	face	181	6	face	1622	275.339
28627	rio	6.21	2	3	1	face	182	2	control	1275	275.339
28629	rio	6.21	2	3	1	face	184	5	control	456	275.339
28639	rio	6.21	2	3	1	face	194	1	face	464	275.339
28640	rio	6.21	2	3	1	face	195	6	control	408	275.339
28641	rio	6.21	2	3	1	face	196	4	control	409	275.339
28642	rio	6.21	2	3	1	face	197	6	control	379	275.339
28644	rio	6.21	2	3	1	face	199	4	control	488	275.339
28645	rio	6.21	2	3	1	face	200	3	control	416	275.339
28646	rio	6.21	2	3	1	face	201	1	face	781	275.339
28647	rio	6.21	2	3	1	face	202	6	control	411	275.339
28648	rio	6.21	2	3	1	face	203	4	face	442	275.339
28649	rio	6.21	2	3	1	face	204	1	control	520	275.339
28650	rio	6.21	2	3	1	face	205	3	control	449	275.339
28651	rio	6.21	2	3	1	face	206	2	control	479	275.339
28652	rio	6.21	2	3	1	face	207	1	face	575	275.339
28653	rio	6.21	2	3	1	face	208	4	control	399	275.339
28654	rio	6.21	2	3	1	face	209	5	face	523	275.339
28655	rio	6.21	2	3	1	face	210	2	control	NA	275.339
28657	rio	6.21	2	3	1	face	212	2	control	436	275.339
28658	rio	6.21	2	3	1	face	213	4	face	384	275.339
28659	rio	6.21	2	3	1	face	214	6	control	1000	275.339
28660	rio	6.21	2	3	1	face	215	4	control	382	275.339
28661	rio	6.21	2	3	1	face	216	4	control	479	275.339
28663	rio	6.21	2	3	1	face	218	2	control	440	275.339
28664	rio	6.21	2	3	1	face	219	5	control	328	275.339
28671	rio	6.21	2	3	1	face	226	4	control	354	275.339
28672	rio	6.21	2	3	1	face	227	5	control	347	275.339
28673	rio	6.21	2	3	1	face	228	6	control	360	275.339
28674	rio	6.21	2	3	1	face	229	4	face	431	275.339
28675	rio	6.21	2	3	1	face	230	3	control	535	275.339
28677	rio	6.21	2	3	1	face	232	1	control	509	275.339
28679	rio	6.21	2	3	1	face	234	5	control	384	275.339
28680	rio	6.21	2	3	1	face	235	5	control	366	275.339
28683	rio	6.21	2	3	1	face	238	4	control	456	275.339
28684	rio	6.21	2	3	1	face	239	6	control	NA	275.339
28686	rod	5.96	5	3	1	face	2	6	control	408	238.938

28687	rod	5.96	5	3	1	face	3	4	control	402	238.938
28688	rod	5.96	5	3	1	face	4	1	control	454	238.938
28689	rod	5.96	5	3	1	face	5	4	control	516	238.938
28691	rod	5.96	5	3	1	face	7	6	control	534	238.938
28692	rod	5.96	5	3	1	face	8	4	control	427	238.938
28696	rod	5.96	5	3	1	face	12	2	control	546	238.938
28697	rod	5.96	5	3	1	face	13	3	control	553	238.938
28698	rod	5.96	5	3	1	face	14	2	control	514	238.938
28700	rod	5.96	5	3	1	face	16	3	control	450	238.938
28701	rod	5.96	5	3	1	face	17	6	control	494	238.938
28703	rod	5.96	5	3	1	face	19	2	control	494	238.938
28704	rod	5.96	5	3	1	face	20	5	control	427	238.938
28705	rod	5.96	5	3	1	face	21	1	control	406	238.938
28706	rod	5.96	5	3	1	face	22	3	face	NA	238.938
28707	rod	5.96	5	3	1	face	23	4	control	466	238.938
28708	rod	5.96	5	3	1	face	24	2	control	953	238.938
28709	rod	5.96	5	3	1	face	25	5	face	565	238.938
28710	rod	5.96	5	3	1	face	26	4	control	497	238.938
28712	rod	5.96	5	3	1	face	28	4	control	474	238.938
28713	rod	5.96	5	3	1	face	29	1	control	507	238.938
28715	rod	5.96	5	3	1	face	31	4	control	479	238.938
28716	rod	5.96	5	3	1	face	32	4	control	382	238.938
28717	rod	5.96	5	3	1	face	33	1	face	996	238.938
28718	rod	5.96	5	3	1	face	34	3	control	526	238.938
28719	rod	5.96	5	3	1	face	35	1	control	515	238.938
28720	rod	5.96	5	3	1	face	36	6	face	580	238.938
28721	rod	5.96	5	3	1	face	37	5	control	546	238.938
28722	rod	5.96	5	3	1	face	38	6	control	480	238.938
28724	rod	5.96	5	3	1	face	40	1	control	520	238.938
28725	rod	5.96	5	3	1	face	41	6	control	358	238.938
28727	rod	5.96	5	3	1	face	43	5	control	415	238.938
28728	rod	5.96	5	3	1	face	44	3	control	590	238.938
28730	rod	5.96	5	3	1	face	46	3	control	491	238.938
28731	rod	5.96	5	3	1	face	47	6	control	415	238.938
28732	rod	5.96	5	3	1	face	48	3	control	501	238.938
28734	rod	5.96	5	3	1	face	50	3	control	484	238.938
28735	rod	5.96	5	3	1	face	51	1	control	422	238.938
28736	rod	5.96	5	3	1	face	52	5	control	409	238.938
28737	rod	5.96	5	3	1	face	53	4	face	468	238.938
28738	rod	5.96	5	3	1	face	54	2	control	520	238.938
28739	rod	5.96	5	3	1	face	55	5	face	515	238.938
28740	rod	5.96	5	3	1	face	56	1	control	465	238.938
28742	rod	5.96	5	3	1	face	58	6	control	406	238.938
28743	rod	5.96	5	3	1	face	59	4	control	430	238.938
28744	rod	5.96	5	3	1	face	60	4	control	431	238.938
28745	rod	5.96	5	3	1	face	61	5	face	NA	238.938
28746	rod	5.96	5	3	1	face	62	6	control	481	238.938
28747	rod	5.96	5	3	1	face	63	6	control	419	238.938
28748	rod	5.96	5	3	1	face	64	4	control	519	238.938
28750	rod	5.96	5	3	1	face	66	4	control	506	238.938

28751	rod	5.96	5	3	1	face	67	2	control	491	238.938
28752	rod	5.96	5	3	1	face	68	2	control	1002	238.938
28754	rod	5.96	5	3	1	face	70	5	control	486	238.938
28755	rod	5.96	5	3	1	face	71	1	control	451	238.938
28756	rod	5.96	5	3	1	face	72	2	control	418	238.938
28757	rod	5.96	5	3	1	face	73	2	face	521	238.938
28758	rod	5.96	5	3	1	face	74	6	control	395	238.938
28759	rod	5.96	5	3	1	face	75	3	control	463	238.938
28760	rod	5.96	5	3	1	face	76	2	control	513	238.938
28762	rod	5.96	5	3	1	face	78	4	control	364	238.938
28763	rod	5.96	5	3	1	face	79	2	face	743	238.938
28764	rod	5.96	5	3	1	face	80	3	control	354	238.938
28765	rod	5.96	5	3	1	face	81	3	face	533	238.938
28766	rod	5.96	5	3	1	face	82	2	control	503	238.938
28767	rod	5.96	5	3	1	face	83	5	control	367	238.938
28768	rod	5.96	5	3	1	face	84	1	control	458	238.938
28770	rod	5.96	5	3	1	face	86	6	control	303	238.938
28771	rod	5.96	5	3	1	face	87	4	control	449	238.938
28772	rod	5.96	5	3	1	face	88	5	control	405	238.938
28773	rod	5.96	5	3	1	face	89	6	face	1233	238.938
28774	rod	5.96	5	3	1	face	90	5	control	1255	238.938
28775	rod	5.96	5	3	1	face	91	6	face	541	238.938
28776	rod	5.96	5	3	1	face	92	2	control	520	238.938
28777	rod	5.96	5	3	1	face	93	3	control	480	238.938
28778	rod	5.96	5	3	1	face	94	3	face	561	238.938
28782	rod	5.96	5	3	1	face	98	6	control	623	238.938
28785	rod	5.96	5	3	1	face	101	5	control	NA	238.938
28786	rod	5.96	5	3	1	face	102	6	control	461	238.938
28787	rod	5.96	5	3	1	face	103	4	face	466	238.938
28788	rod	5.96	5	3	1	face	104	5	control	845	238.938
28790	rod	5.96	5	3	1	face	106	3	control	427	238.938
28791	rod	5.96	5	3	1	face	107	5	control	678	238.938
28792	rod	5.96	5	3	1	face	108	6	control	406	238.938
28793	rod	5.96	5	3	1	face	109	1	face	492	238.938
28794	rod	5.96	5	3	1	face	110	1	control	906	238.938
28795	rod	5.96	5	3	1	face	111	1	control	382	238.938
28797	rod	5.96	5	3	1	face	113	5	control	344	238.938
28799	rod	5.96	5	3	1	face	115	1	control	427	238.938
28800	rod	5.96	5	3	1	face	116	4	face	511	238.938
28801	rod	5.96	5	3	1	face	117	3	control	393	238.938
28802	rod	5.96	5	3	1	face	118	1	face	427	238.938
28803	rod	5.96	5	3	1	face	119	2	control	506	238.938
28804	rod	5.96	5	3	1	face	120	1	control	484	238.938
28805	rod	5.96	5	3	1	face	121	2	face	439	238.938
28806	rod	5.96	5	3	1	face	122	6	control	525	238.938
28807	rod	5.96	5	3	1	face	123	2	control	363	238.938
28809	rod	5.96	5	3	1	face	125	3	control	375	238.938
28810	rod	5.96	5	3	1	face	126	3	control	425	238.938
28811	rod	5.96	5	3	1	face	127	5	control	543	238.938
28812	zaz	7.02	1	3	1	face	1	3	control	669	242.811

28813	zaz	7.02	1	3	1	face	2	2	control	529	242.811
28814	zaz	7.02	1	3	1	face	3	1	control	596	242.811
28815	zaz	7.02	1	3	1	face	4	6	control	548	242.811
28817	zaz	7.02	1	3	1	face	6	4	control	496	242.811
28818	zaz	7.02	1	3	1	face	7	5	control	528	242.811
28820	zaz	7.02	1	3	1	face	9	3	control	546	242.811
28821	zaz	7.02	1	3	1	face	10	4	control	584	242.811
28822	zaz	7.02	1	3	1	face	11	2	face	NA	242.811
28823	zaz	7.02	1	3	1	face	12	1	control	2182	242.811
28827	zaz	7.02	1	3	1	face	16	4	control	857	242.811
28828	zaz	7.02	1	3	1	face	17	2	control	606	242.811
28829	zaz	7.02	1	3	1	face	18	1	control	542	242.811
28830	zaz	7.02	1	3	1	face	19	3	face	NA	242.811
28831	zaz	7.02	1	3	1	face	20	1	control	578	242.811
28832	zaz	7.02	1	3	1	face	21	6	control	608	242.811
28833	zaz	7.02	1	3	1	face	22	2	control	546	242.811
28834	zaz	7.02	1	3	1	face	23	6	face	NA	242.811
28837	zaz	7.02	1	3	1	face	26	2	control	659	242.811
28838	zaz	7.02	1	3	1	face	27	5	control	NA	242.811
28841	zaz	7.02	1	3	1	face	30	4	control	752	242.811
28842	zaz	7.02	1	3	1	face	31	4	face	NA	242.811
28844	zaz	7.02	1	3	1	face	33	6	control	517	242.811
28845	zaz	7.02	1	3	1	face	34	6	control	662	242.811
28846	zaz	7.02	1	3	1	face	35	3	control	543	242.811
28848	zaz	7.02	1	3	1	face	37	4	control	NA	242.811
28849	zaz	7.02	1	3	1	face	38	1	face	NA	242.811
28851	zaz	7.02	1	3	1	face	40	1	control	771	242.811
28852	zaz	7.02	1	3	1	face	41	5	control	518	242.811
28853	zaz	7.02	1	3	1	face	42	1	face	NA	242.811
28854	zaz	7.02	1	3	1	face	43	2	control	644	242.811
28857	zaz	7.02	1	3	1	face	46	3	control	977	242.811
28858	zaz	7.02	1	3	1	face	47	5	control	678	242.811
28859	zaz	7.02	1	3	1	face	48	6	control	500	242.811
28861	zaz	7.02	1	3	1	face	50	6	control	490	242.811
28862	zaz	7.02	1	3	1	face	51	1	control	597	242.811
28863	zaz	7.02	1	3	1	face	52	3	face	NA	242.811
28864	zaz	7.02	1	3	1	face	53	5	control	470	242.811
28865	zaz	7.02	1	3	1	face	54	4	control	512	242.811
28866	zaz	7.02	1	3	1	face	55	6	control	522	242.811
28868	zaz	7.02	1	3	1	face	57	3	control	513	242.811
28869	zaz	7.02	1	3	1	face	58	6	face	1528	242.811
28870	zaz	7.02	1	3	1	face	59	2	control	688	242.811
28871	zaz	7.02	1	3	1	face	60	2	control	621	242.811
28872	zaz	7.02	1	3	1	face	61	6	face	NA	242.811
28873	zaz	7.02	1	3	1	face	62	1	control	572	242.811
28874	zaz	7.02	1	3	1	face	63	4	control	610	242.811
28875	zaz	7.02	1	3	1	face	64	5	control	623	242.811
28878	zaz	7.02	1	3	1	face	67	2	control	1274	242.811
28879	zaz	7.02	1	3	1	face	68	5	control	473	242.811
28880	zaz	7.02	1	3	1	face	69	4	face	NA	242.811

28881	zaz	7.02	1	3	1	face	70	4	control	NA	242.811
28883	zaz	7.02	1	3	1	face	72	2	control	594	242.811
28885	zaz	7.02	1	3	1	face	74	3	control	576	242.811
28887	zaz	7.02	1	3	1	face	76	5	control	595	242.811
28888	zaz	7.02	1	3	1	face	77	2	control	641	242.811
28889	zaz	7.02	1	3	1	face	78	4	control	645	242.811
28891	zaz	7.02	1	3	1	face	80	1	control	604	242.811
28893	zaz	7.02	1	3	1	face	82	5	control	520	242.811
28894	zaz	7.02	1	3	1	face	83	6	control	548	242.811
28895	zaz	7.02	1	3	1	face	84	6	control	510	242.811
28896	zaz	7.02	1	3	1	face	85	2	face	NA	242.811
28899	zaz	7.02	1	3	1	face	88	3	control	1136	242.811
28900	zaz	7.02	1	3	1	face	89	1	control	618	242.811
28902	zaz	7.02	1	3	1	face	91	1	control	543	242.811
28903	zaz	7.02	1	3	1	face	92	4	control	541	242.811
28905	zaz	7.02	1	3	1	face	94	3	control	576	242.811
28906	zaz	7.02	1	3	1	face	95	2	control	521	242.811
28908	zaz	7.02	1	3	1	face	97	3	control	NA	242.811
28909	zaz	7.02	1	3	1	face	98	4	control	515	242.811
28910	zaz	7.02	1	3	1	face	99	5	face	NA	242.811
28911	zaz	7.02	1	3	1	face	100	6	control	NA	242.811
28914	zaz	7.02	1	3	1	face	103	6	control	517	242.811
28916	zaz	7.02	1	3	1	face	105	1	control	579	242.811
28918	zaz	7.02	1	3	1	face	107	3	control	725	242.811
28919	zaz	7.02	1	3	1	face	108	2	control	537	242.811
28925	zaz	7.02	1	3	1	face	114	1	control	628	242.811
28926	zaz	7.02	1	3	1	face	115	3	control	744	242.811
28928	zaz	7.02	1	3	1	face	117	4	control	604	242.811
28929	zaz	7.02	1	3	1	face	118	6	control	547	242.811
28930	zaz	7.02	1	3	1	face	119	1	control	553	242.811
28931	zaz	7.02	1	3	1	face	120	1	face	NA	242.811
28934	zaz	7.02	1	3	1	face	123	4	control	935	242.811
28935	zaz	7.02	1	3	1	face	124	5	control	567	242.811
28936	zaz	7.02	1	3	1	face	125	3	control	649	242.811
28938	zaz	7.02	1	3	1	face	127	3	control	538	242.811
28939	zaz	7.02	1	3	1	face	128	3	face	NA	242.811
28944	zaz	7.02	1	3	1	face	133	6	control	467	242.811
28945	zaz	7.02	1	3	1	face	134	2	control	538	242.811
28946	zaz	7.02	1	3	1	face	135	5	control	474	242.811
28947	zaz	7.02	1	3	1	face	136	5	face	NA	242.811
28949	zaz	7.02	1	3	1	face	138	5	control	491	242.811
28950	zaz	7.02	1	3	1	face	139	2	face	NA	242.811
28951	zaz	7.02	1	3	1	face	140	2	control	489	242.811
28953	zaz	7.02	1	3	1	face	142	1	control	557	242.811
28954	zaz	7.02	1	3	1	face	143	2	control	552	242.811
28955	zaz	7.02	1	3	1	face	144	5	control	527	242.811
28957	zaz	7.02	1	3	1	face	146	5	control	680	242.811
